# Excess Met1-linked ubiquitination leads to solid aggregate formation

**DOI:** 10.1038/s44318-026-00909-7

**Published:** 2026-09-28

**Authors:** Stephanie Kaypee, Minori Miyasaka, Takuto Nakajima, Hiromi Nishimura, Jun-ichi Sakamaki, Masaaki Komatsu, Fumiyo Ikeda

**Affiliations:** 1 Graduate School of Frontier Biosciences, The University of Osaka, Suita, Osaka 565-0871 Japan; 2 Department of Physiology, Juntendo University Graduate School of Medicine, Tokyo, 113-8421 Japan

**Keywords:** Post-translational Modifications & Proteolysis, Translation & Protein Quality

## Abstract

The ubiquitin ligase HOIL-1 regulates the formation of Met1-linked (linear) ubiquitin chains through its coordination with the E3 ligase HOIP within the Linear Ubiquitin Chain Assembly Complex (LUBAC). While HOIP-dependent Met1-linked ubiquitination is well established in inflammation and immunity, the physiological importance of its quantitative control remains unclear. Here, we show that cells expressing catalytically inactive HOIL-1 accumulate increased α-synuclein, tau, and amyloid-β aggregates. This is associated with defective late-stage autophagic flux, characterized by impaired delivery of p62-positive aggregates to lysosomes. In parallel, p62 bodies undergo a biophysical transition from dynamic, liquid-like condensates to rigid, solid-like structures. Elevation of Met1-linked ubiquitin chains, either through HOIL-1 inactivation or depletion of the Met1-specific deubiquitinase OTULIN, phenocopies these defects. Together, our findings identify HOIL-1 as a key regulator of aggregate clearance and proteostasis through quantitative control of Met1-linked ubiquitination.

## Synopsis

LUBAC-mediated linear ubiquitination is important in immunity and inflammation, but its role in protein quality control is less established. Here, catalytic activity of its HOIL-1 subunit is found to restrain Met1-linked ubiquitination, to maintain the dynamic properties of p62 condensates and promote aggregate clearance.

- HOIL-1 catalytic activity prevents accumulation of neurodegenerative protein aggregates.
- Inactivation of HOIL-1 impairs late-stage autophagic clearance of protein aggregates.
- Inactivation of HOIL-1 shifts aggregates into rigid, solid-like states.
- Excess Met1-ubiquitin chains drive aggregate solidification.

## Abstract (web summary)

LUBAC subunit HOIL-1 restrains linear ubiquitination to maintain dynamic properties of p62 condensates and promote aggregate clearance.

## Graphical abstract

**Figure:**
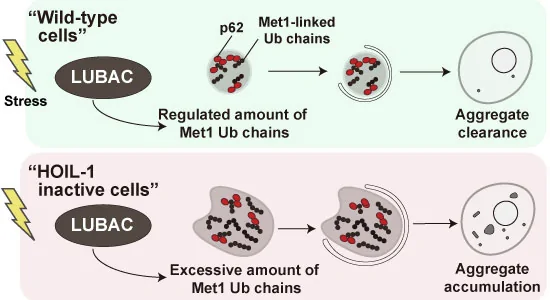

## Introduction

Proteostasis, the maintenance of protein homeostasis, is crucial for ensuring proper protein synthesis, folding, trafficking and degradation (Bauer et al, 2023; Sala et al, 2017). Failure of proteostasis results in protein misfolding and aggregation (Ciechanover and Kwon, 2015; Sala et al, 2017). Protein aggregation is a characteristic feature of neurodegenerative disorders such as Alzheimer’s disease (AD), Parkinson’s disease (PD), and amyotrophic lateral sclerosis (ALS) (Moda et al, 2023). Accumulation of misfolded proteins in neurodegenerative diseases, including α-synuclein, tau, amyloid-β (Aβ), and TAR DNA-binding protein 43 (TDP-43), disrupts cellular homeostasis and leads to neuronal dysfunction (Ciechanover and Kwon, 2015; Moda et al, 2023). Cells employ specialized pathways to manage protein aggregates, such as degradation via the ubiquitin-proteasome system (UPS) and autophagic clearance (Kocaturk and Gozuacik, 2018). The UPS targets misfolded or damaged ubiquitin-tagged proteins for degradation through the 26S proteasome (Hipp et al, 2019); however, its ability to degrade large aggregates is limited (Kocaturk and Gozuacik, 2018). Autophagy complements the UPS by engulfing protein aggregates into autophagosomes, which fuse with lysosomes for degradation (Dikic, 2017).

While ubiquitination is a key signal for both the UPS and autophagy, the specific types and topologies of ubiquitin chains play distinct roles in these processes (Agrata and Komander, 2025; Akizuki et al, 2024). Of particular interest in proteostasis and aggregate clearance are Met1-linked ubiquitin chains, whose critical yet underexplored involvement in cellular quality control and neurodegenerative contexts has recently been highlighted (Bellenguez et al, 2022; Furthmann et al, 2023; Tokunaga and Ikeda, 2022; van Well et al, 2019). For example, a recent genetic study on the etiology of AD and related dementias identified several key genes, including “Heme-oxidized IRP2 ubiquitin ligase 1 (HOIL-1)”, “Shank-associated RH domain-interacting protein (SHARPIN)”, “OTU domain-containing deubiquitinase with linear linkage specificity (OTULIN)”, and “TNFAIP3-interacting protein 1 (TNIP1)/ABIN1” (Bellenguez et al, 2022), all of which play roles in the Met1-linked ubiquitination system (Rittinger and Ikeda, 2017; Wagner et al, 2008). Met1-linked ubiquitin chains have been implicated in distinct signaling pathways, including inflammation (Fiil and Gyrd-Hansen, 2021; Jahan et al, 2021) and, more recently, cellular quality control (Tokunaga and Ikeda, 2022), although their precise roles in modulating aggregate dynamics and clearance remain poorly understood. A comprehensive understanding of how Met1-linked ubiquitination—particularly through its intricate regulation by Linear Ubiquitin Chain Assembly Complex (LUBAC) components such as HOIL-1—influences aggregate dynamics and clearance is crucial for the development of targeted therapeutic strategies against neurodegenerative proteinopathies.

**Figure 1:**
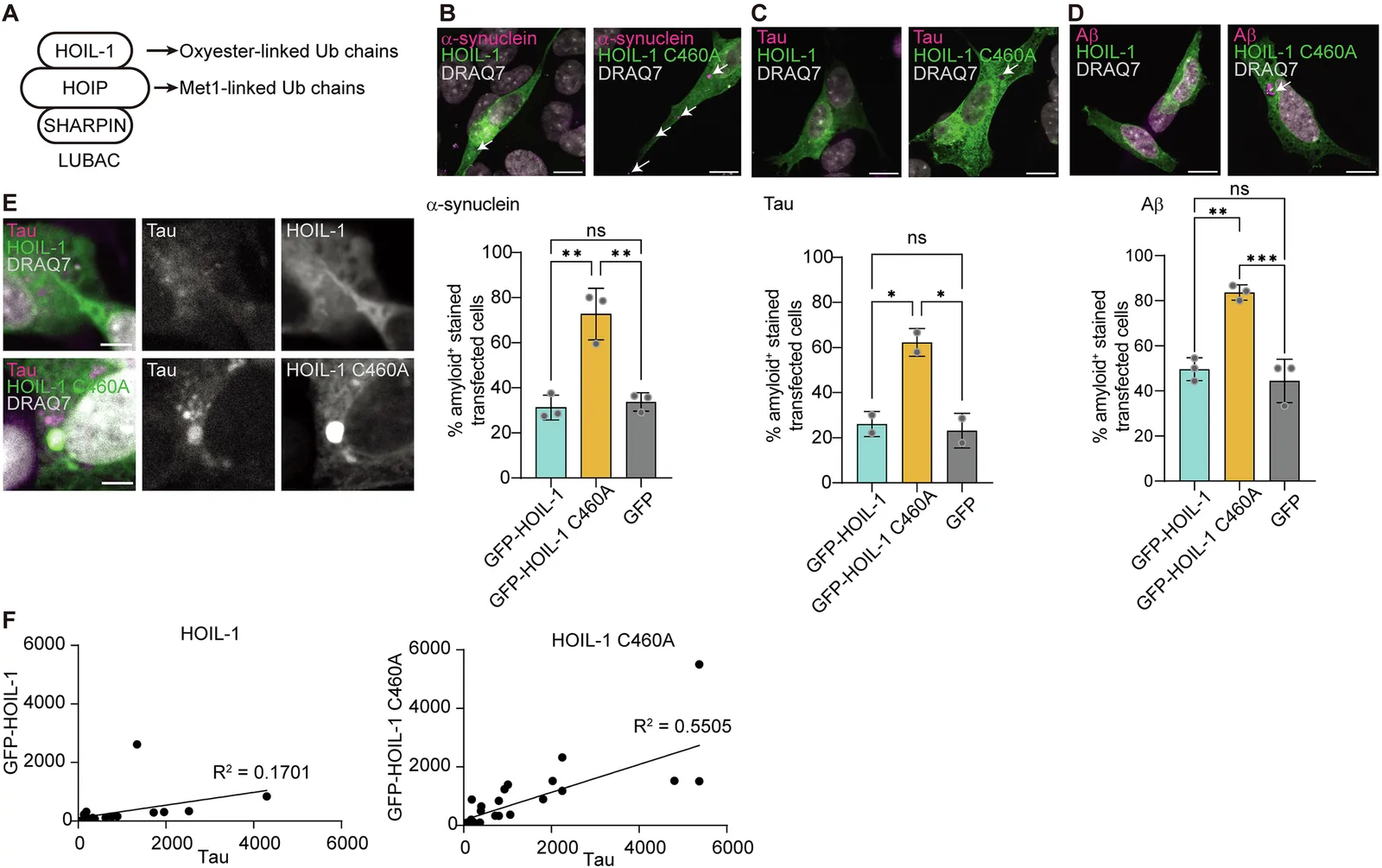
Aggregate-prone proteins accumulate in cells expressing the catalytically inactive HOIL-1 C460A mutant. (**A**) Schematic representation of the LUBAC subunits and their distinct catalytic activities. (**B**–**D**) Comparison of α-synuclein (**B**), tau (**C**), and Aβ (**D**) aggregates (indicated by arrows) in SH-SY5Y cells transiently expressing GFP-HOIL-1, GFP-HOIL-1 C460A or GFP-alone (GFP). Scale bar = 10 μm. The graphs below show the percentage of GFP-positive cells containing α-synuclein, tau, or Aβ aggregates, respectively. Mean ± SD was plotted, and one-way ANOVA followed by Tukey’s multiple-comparison test was performed; (**B**) **P = 0.002, **P = 0.0014, (**C**) *P = 0.0189, *0.0234 (**D**) ***P = 0.0008, **P = 0.0018. (**B**) *n* ≥ 100 cells, *N* = 3, (**C**) *n* ≥ 97 cells, *N* = 2, (**D**) *n* ≥ 23 cells, *N* = 3 independent biological repeats. (**E**) Representative microscopy images showing colocalization of tau aggregates and GFP-tagged HOIL-1 or HOIL-1 C460A in SH-SY5Y cells. Scale bar, 5 μm. *N* = 2 independent biological repeats. (**F**) Linear regression analysis of tau aggregate and GFP signal intensities. Regions of interest (ROIs) were defined based on tau aggregate fluorescence, and GFP fluorescence intensity within the corresponding regions was quantified. For GFP-HOIL-1, *R*² = 0.1701, *P* = 0.0505 (ns); for GFP-HOIL-1 C460A, *R*² = 0.5505, *P* < 0.0001 (****). Source data are available online for this figure.

LUBAC is responsible for forming these critical Met1-linked ubiquitin chains (Kirisako et al, 2006). LUBAC comprises the E3 ligase “HOIL-1-interacting protein (HOIP)”, which has a catalytic center for Met1 ubiquitination, another E3 ligase, HOIL-1, and the regulatory subunit SHARPIN (Gerlach et al, 2011; Ikeda et al, 2011; Tokunaga et al, 2011) (Fig. 1A). HOIL-1 plays a unique regulatory role in this complex, which is crucial for Met1-linked ubiquitination (Kelsall et al, 2019). Its catalytic activity, which is involved in the formation of specific heterotypic ubiquitin chains, is essential for fine-tuning the overall levels of Met1-linked ubiquitin chains (Rodriguez Carvajal et al, 2021). Indeed, its catalytic inactivation leads to an increase in these chains, suggesting a suppressive or balancing function of HOIL-1 on Met1-linked ubiquitination activity of HOIP. A growing body of evidence highlights the diverse functions of LUBAC, extending from immune signal regulation to cellular quality control mechanisms, particularly the regulation of protein aggregation (Tokunaga and Ikeda, 2022). Recent findings suggest that Met1-linked ubiquitination is involved in marking misfolded or aggregated proteins and modulating stress responses (Asaoka et al, 2016; van Well et al, 2019). LUBAC components have been observed to localize to protein aggregates, and their activity appears to influence the clearance of aggregates through autophagy or proteasomal degradation (Furthmann et al, 2023; van Well et al, 2019). However, despite these observations, the specific contribution of HOIL-1 catalytic activity to these processes, particularly its impact on the material properties of aggregates and efficiency of autophagic flux, remains poorly defined. Specifically, three critical questions persist: (1) How does HOIL-1 catalytic activity specifically regulate aggregate clearance? (2) What is the relationship between the Met1-linked chain levels and the biophysical properties of aggregates? (3) How does this regulation affect autophagic flux?

To address these critical questions, we hypothesized that the catalytic activity of HOIL-1 within LUBAC is crucial for fine-tuning Met1-linked ubiquitination and that dysregulation of these chains directly affects the biophysical properties of aggregates, thereby preventing the accumulation of toxic protein aggregates and maintaining their dynamic liquid-like state to facilitate efficient autophagic clearance. To test this hypothesis, we systematically investigated the role of HOIL-1 catalytic activity in aggregate management using cellular models of neurodegeneration and in vitro biochemical reconstitution, with a focus on its impact on aggregate biophysical properties and autophagic flux.

## Results

### Aggregate-prone proteins accumulate amyloid in neuroblastoma cells expressing a catalytically inactive HOIL-1 mutant

**Figure EV1:**
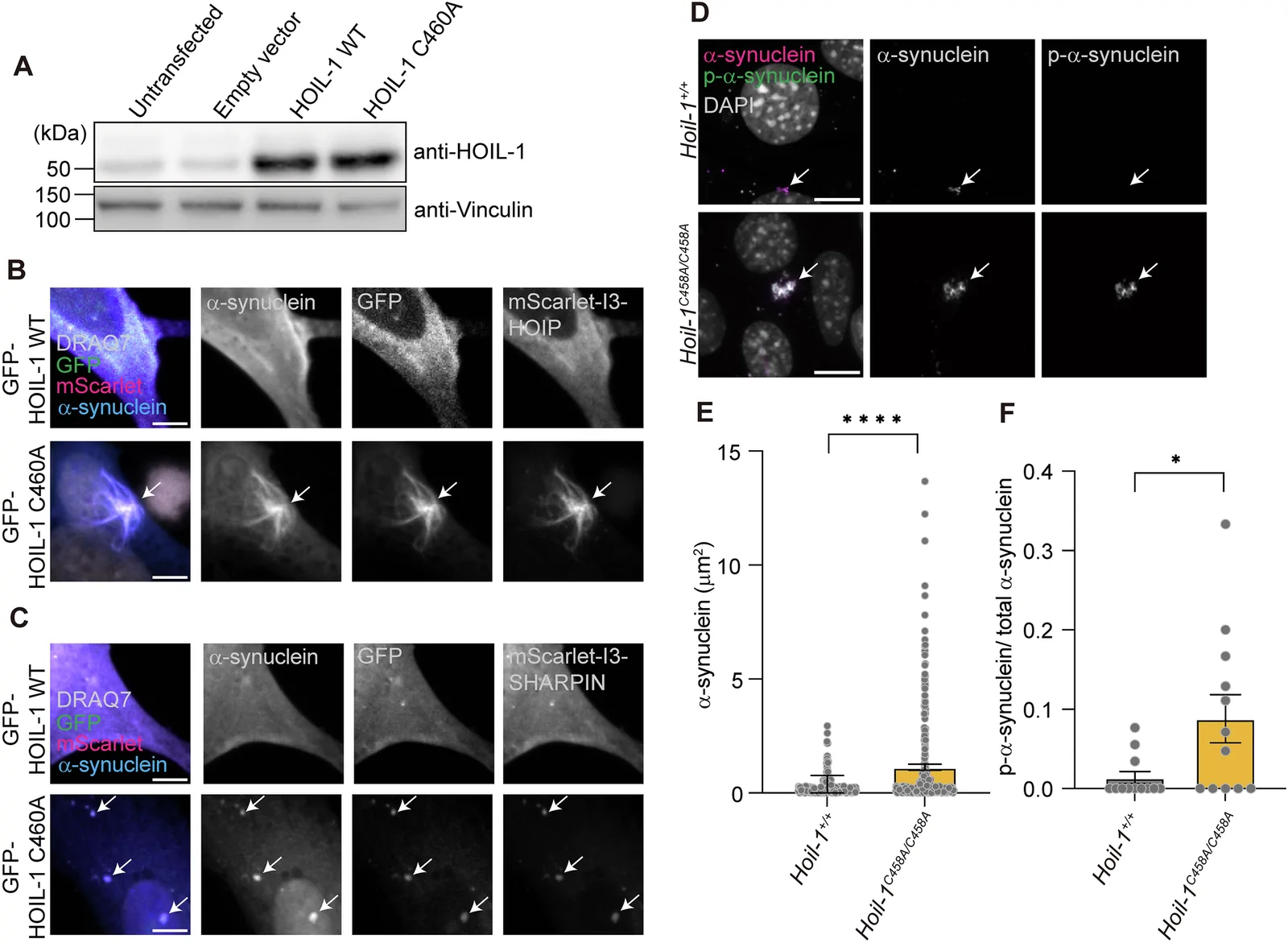
LUBAC components localize to protein aggregates, which accumulate in cells expressing catalytically inactive HOIL-1. (**A**) Immunoblot analysis of HOIL-1 expression in SH-SY5Y cells 48 h after transfection with HOIL-1 or HOIL-1 C460A. Untransfected and empty vector (EV)-transfected cells served as controls. Vinculin was used as a loading control. Representative data from one experiment. (**B**,** C**) Representative fluorescence microscopy images showing colocalization of α-synuclein aggregates with GFP-HOIL-1 or GFP-HOIL-1 C460A and either (**B**) mScarlet-I3-HOIP or (**C**) mScarlet-I3-SHARPIN in SH-SY5Y cells. Representative images from three independent biological experiments are shown. Scale bar, 5 μm. Arrows indicate the position of α-synuclein-positive puncta across each channel. (**D**) Representative immunofluorescence images showing colocalization of α-synuclein aggregates and phosphorylated α-synuclein (p-α-synuclein) in *Hoil-1*^+/+^ and *Hoil-1*^*C458A/C458A*^ MEFs. Representative images from three independent biological experiments are shown. Scale bar, 10 μm. Arrows indicate the position of α-synuclein-positive puncta across each channel. (**E**) Quantification of α-synuclein aggregate size from (**D**). Data were presented as mean ± SEM from more than 100 cells per condition across three independent biological experiments. Statistical significance was determined using a Mann–Whitney test (****P = 8.9 × 10^−5^). (**F**) Quantification of the ratio of p-α-synuclein-positive puncta to total α-synuclein puncta from (**D**). Data were presented as mean ± SEM from *n* = 12 fields across three independent biological experiments. Statistical significance was determined using an unpaired *t*-test (*P = 0.0268).

To determine whether HOIL-1 catalytic activity influences protein aggregation, we used SH-SY5Y (human neuroblastoma) cells, a widely recognized cellular model for studying neurodegenerative protein aggregation. We examined three well-established aggregation-prone proteins in these cells, α-synuclein, tau, and Aβ. SH-SY5Y cells expressing the catalytically inactive HOIL-1 C460A mutant (where the ubiquitin-loading Cys is mutated to Ala) (Fig. EV1A) showed a significantly higher number of amyloid-positive aggregates for α-synuclein, tau, and Aβ compared to cells expressing wild-type HOIL-1 (Fig. 1B–D). These α-synuclein aggregates in HOIL-1 C460A-expressing cells colocalized with all the LUBAC components, namely HOIP, SHARPIN and HOIL-1 (Fig. EV1B,C). Notably, HOIL-1 C460A was detected within these structures, as demonstrated by its colocalization with tau aggregates (Fig. 1E,F).

Next, we investigated whether the loss of HOIL-1 catalytic activity in an endogenous protein produces similar effects. Mouse embryonic fibroblasts (MEFs) derived from *Hoil-1*^*C458A/C458A*^ mice generated by CRISPR/Cas9 (Rodriguez Carvajal et al, 2021) or *Hoil-1*^*+/+*^ littermate controls were used to assess the physiological relevance of HOIL-1 catalytic activity in an endogenous, non-neuronal context and to confirm that the observed phenotypes were not cell-type specific. We examined the total α-synuclein and its disease-associated phosphorylated form (pS129) in MEFs (Oueslati, 2016). *Hoil-1*^*C458A/C458A*^ MEFs accumulated larger α-synuclein-positive amyloid than *Hoil-1*^*+/+*^ MEFs (Fig. EV1D,E). Consistent with the pathological aggregation, *Hoil-1*^*C458A/C458A*^ MEFs exhibited prominent pS129-α-synuclein aggregates, whereas *Hoil-1*^*+/+*^ MEFs displayed minimal staining (Fig. EV1D,F). These findings demonstrate that the loss of HOIL-1 catalytic activity drives the buildup of both total and pathologically modified α-synuclein aggregates.

### Aggregate clearance is defective in MEFs expressing a catalytically inactive HOIL-1 mutant

**Figure EV2:**
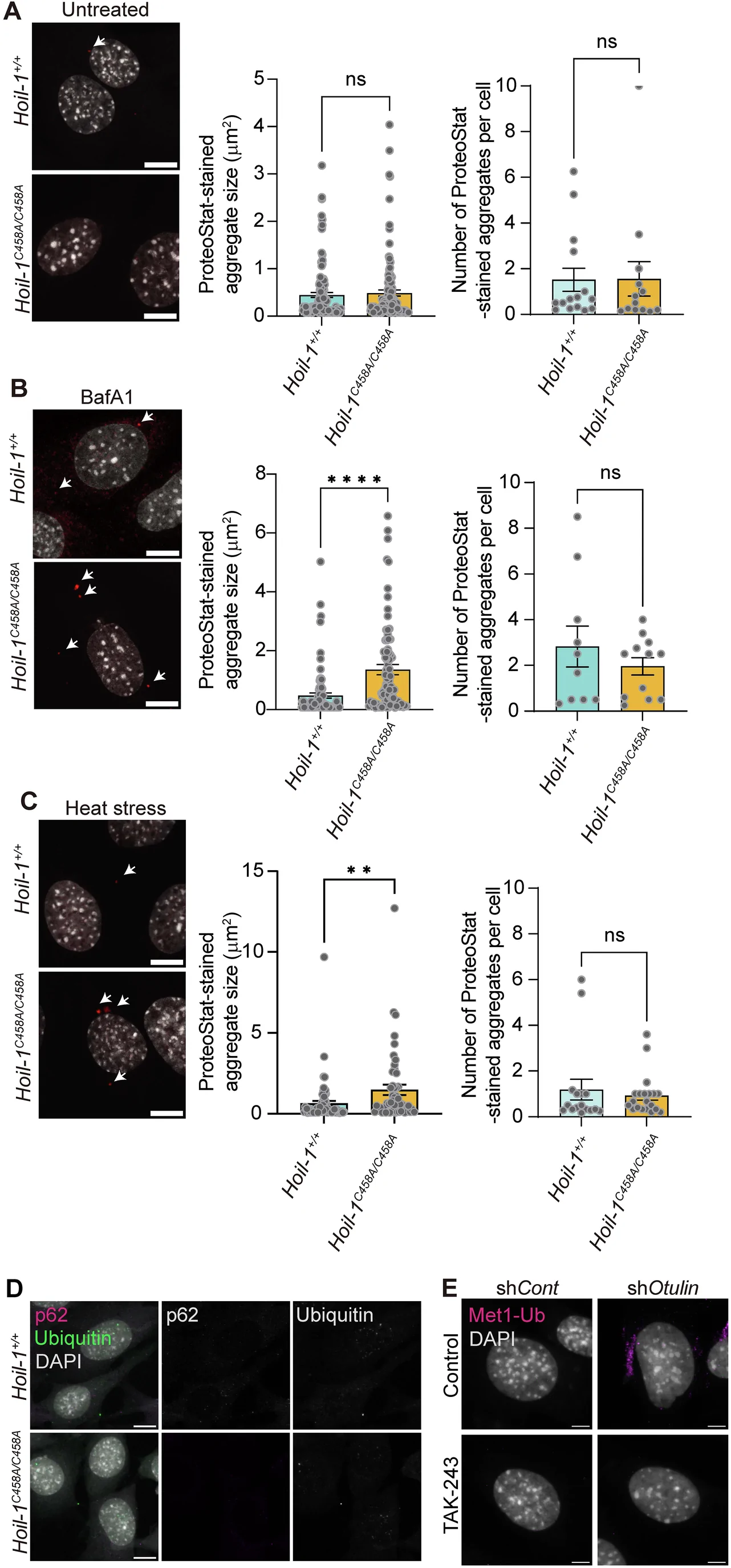
Characterization of proteotoxic stress-induced aggregates in *Hoil-1*^*C458A/C458A*^ cells. (**A**–**C**) Representative images of ProteoStat-stained aggregates (arrows) in *Hoil-1*^+/+^ and *Hoil-1*^*C458A/C458A*^ MEFs under (**A**) untreated and proteotoxic stress conditions, (**B**) treatment with 100 nM bafilomycin A1 (BafA1), and (**C**) heat stress (42 °C). Scale bar, 5 μm. Quantification of aggregate size and aggregate number per cell is shown alongside the representative images. Data were presented as mean ± SEM from two independent biological experiments (untreated, *n* > 75 cells; BafA1, *n* > 30 cells; heat stress, *n* > 55 cells per condition). Statistical significance was determined using a Mann–Whitney test ((**A**) size: ns, *P* = 0.5399; number: ns, *P* = 0.0146; (**B**) size: ****P = 2.8 × 10^−8^; number: ns, *P* = 0.5343; (**C**) size: ** *P* = 0.0029; number: ns, *P* = 0.0280). (**D**) Representative immunofluorescence images showing p62 and ubiquitin in untreated *Hoil-1*^+/+^ and *Hoil-1*^C458A/C458A^ MEFs. Representative images from two independent biological experiments are shown. Scale bar, 5 μm. (**E**) Representative fluorescence microscopy images showing Met1-linked ubiquitin chains in sh*Control* and sh*Otulin* MEFs treated with or without the E1 ubiquitin-activating enzyme inhibitor TAK243 (1 μM, 4 h). Scale bar, 5 μm.

To examine how HOIL-1 catalytic activity contributes to proteostasis, we challenged *Hoil-1*^*+/+*^ and *Hoil-1*^*C458A/C458A*^ MEFs with either heat stress (42 °C) or lysosomal inhibition using a specific inhibitor of V-ATPase, bafilomycin A1. The aggregates were visualized using ProteoStat, a dye that selectively labels misfolded protein aggregates. Under both stress conditions, *Hoil-1*^*C458A/C458A*^ MEFs accumulated similar numbers yet significantly larger aggregates than *Hoil-1*^*+/+*^ MEFs (Fig. EV2A–C), suggesting that the loss of HOIL-1 catalytic activity sensitizes cells to proteotoxic stress.

**Figure 2:**
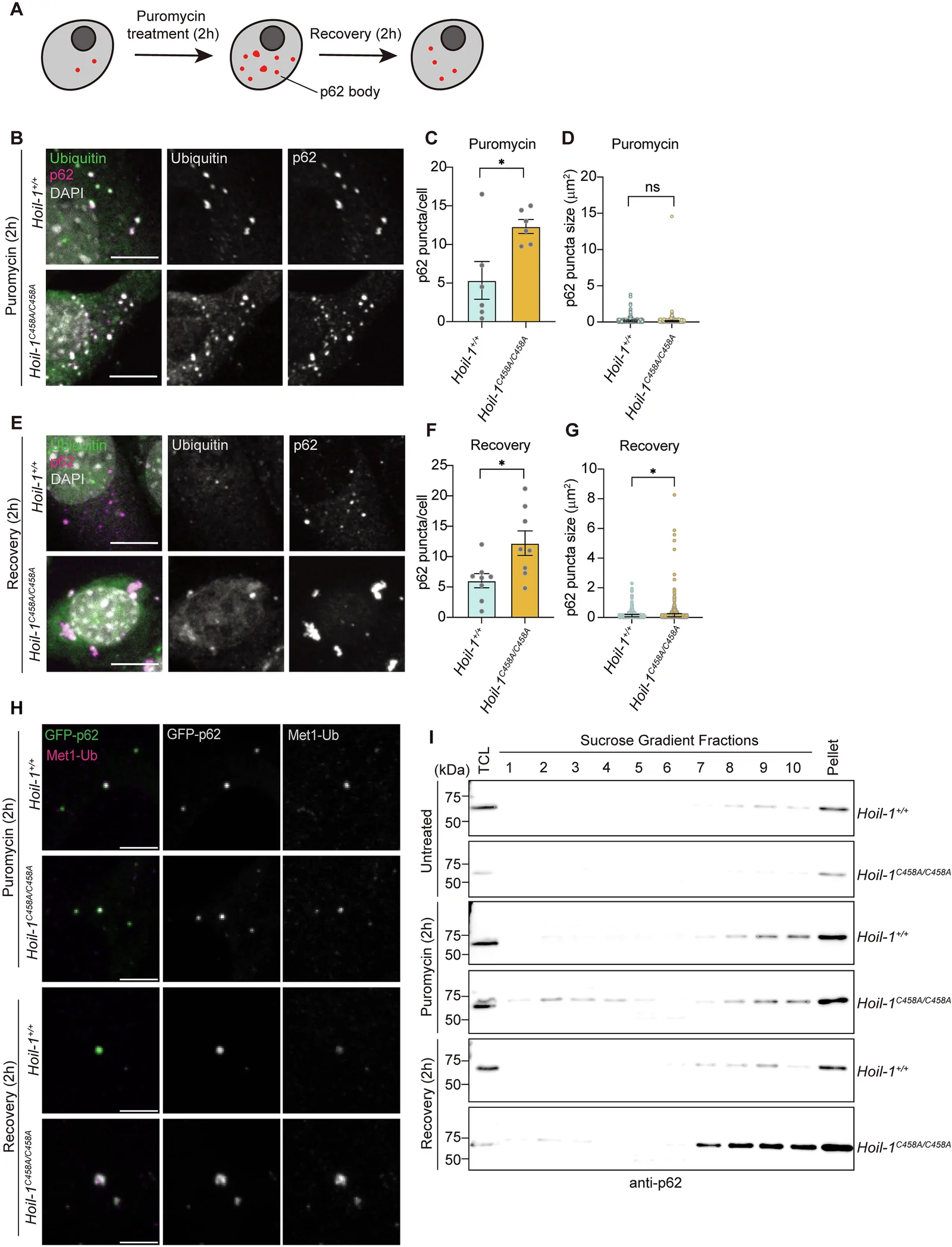
Defect in p62 body clearance in *Hoil-1*^*C458A/C458A*^ cells. (**A**) Schematic of the puromycin chase assay. (**B**) *Hoil-1*^*+/+*^ and *Hoil-1*^*C458A/C458A*^ MEFs were treated with puromycin (5 μg/ml) for 2 h. Representative immunofluorescence images showing p62 and ubiquitin staining following puromycin treatment. Scale bar, 5 μm. Representative images from three independent biological experiments. (**C**,** D**) Quantification of (**C**) the number of p62 puncta per cell and (**D**) p62 puncta size following puromycin treatment. In (**C**), data were presented as mean ± SEM and were analyzed using an unpaired *t*-test (*P = 0.0235). Data were from three independent biological experiments (*Hoil-1*^*+/+*^: *n* = 68 cells; *Hoil-1*^*C458A/C458A*^: *n* = 38 cells). In (**D**), data were presented as median with interquartile range and were analyzed using a Mann–Whitney test (ns, *P* = 0.4380). Data were from three independent biological experiments (*Hoil-1*^*+/+*^: *n* = 403 puncta; *Hoil-1*^*C458A/C458A*^: *n* = 473 puncta). (**E**) *Hoil-1*^*+/+*^ and *Hoil-1*^*C458A/C458A*^ MEFs were treated with puromycin (5 μg/ml) for 2 h, followed by a 2 h recovery period in puromycin-free medium. Representative immunofluorescence images showing p62 and ubiquitin staining after the recovery period. Scale bar, 5 μm. Representative images from three independent biological experiments. (**F**,** G**) Quantification of (**F**) the number of p62 puncta per cell and (**G**) p62 puncta size following the 2 h recovery period. In (**F**), data were presented as mean ± SEM and were analyzed using an unpaired *t*-test (*P = 0.019). Data were from three independent biological experiments (*Hoil-1*^*+/+*^: *n* = 48 cells; *Hoil-1*^*C458A/C458A*^: *n* = 60 cells). In (**G**), data were presented as median with interquartile range and were analyzed using a Mann–Whitney test (*P = 0.0315). Data were from three independent biological experiments (*Hoil-1*^*+/+*^: *n* = 333 puncta; *Hoil-1*^*C458A/C458A*^: *n* = 687 puncta). (**H**) *Hoil-1*^*+/+*^ and *Hoil-1*^*C458A/C458A*^ MEFs expressing GFP-tagged p62 were treated with puromycin (5 μg/ml) for 2 h and placed in puromycin-free media for 2 h for recovery. Representative microscopy images to show the colocalization of GFP-tagged p62 and Met1-linked ubiquitin chain. Representative images from two biological repeats. Scale bar, 5 μm. (**I**) Sucrose gradient fractionation analysis of p62 distribution in *Hoil-1*^*+/+*^ and *Hoil-1*^*C458A/C458A*^ MEFs under untreated conditions, following puromycin treatment (5 μg/ml, 2 h), or after a 2 h recovery period in puromycin-free medium, as indicated. Representative data from three independent biological experiments. Source data are available online for this figure.

To determine whether HOIL-1 regulates aggregate formation or clearance, we performed a puromycin chase assay. Puromycin, a tRNA mimic, induces the premature termination of nascent polypeptides, generating misfolded proteins that rapidly coalesce into amorphous aggregates (Lelouard et al, 2004; Zaffagnini et al, 2018). MEFs were treated with puromycin for 2 h to induce aggregate formation, followed by a 2 h recovery period in puromycin-free medium to allow aggregate clearance (Fig. 2A). Aggregates were monitored by immunocytochemistry staining for endogenous p62, a ubiquitin-dependent receptor for misfolded proteins, and ubiquitin (Figs. EV2D and 2B–G). Following puromycin treatment, *Hoil-1*^*C458A/C458A*^ MEFs displayed an increased number of p62-positive puncta (p62 bodies) per cell relative to *Hoil-1*^*+/+*^ MEFs, although their sizes were comparable at this stage (Fig. 2B–D). In contrast, differences were observed during the recovery phase. After recovery from puromycin treatment, *Hoil-1*^*+/+*^ MEFs exhibited fewer large p62 bodies compared with *Hoil-1*^*C458A/C458A*^ MEFs (Fig. 2E–G). These p62 bodies colocalized with Met1-linked ubiquitin signals, as determined using a specific antibody against Met1-linked ubiquitin chains, suggesting a role for these chains in the formation or maintenance of p62 bodies (Figs. EV2E and 2H).

To verify that p62 bodies corresponded to insoluble aggregates, we performed sucrose gradient fractionation of lysates from MEFs subjected to puromycin treatment and recovery. Consistent with microscopic observations, denser fractions from *Hoil-1*^*C458A/C458A*^ MEFs contained increased signals of p62 specifically during the recovery phase (Fig. 2I). Together, these results demonstrate that the loss of HOIL-1 catalytic activity impairs the efficient clearance of misfolded protein aggregates positive for p62 and ubiquitin, leading to their aberrant accumulation.

### Late-stage aggrephagy is impaired in *Hoil-1*^*C458A/C458A*^ MEFs

**Figure 3:**
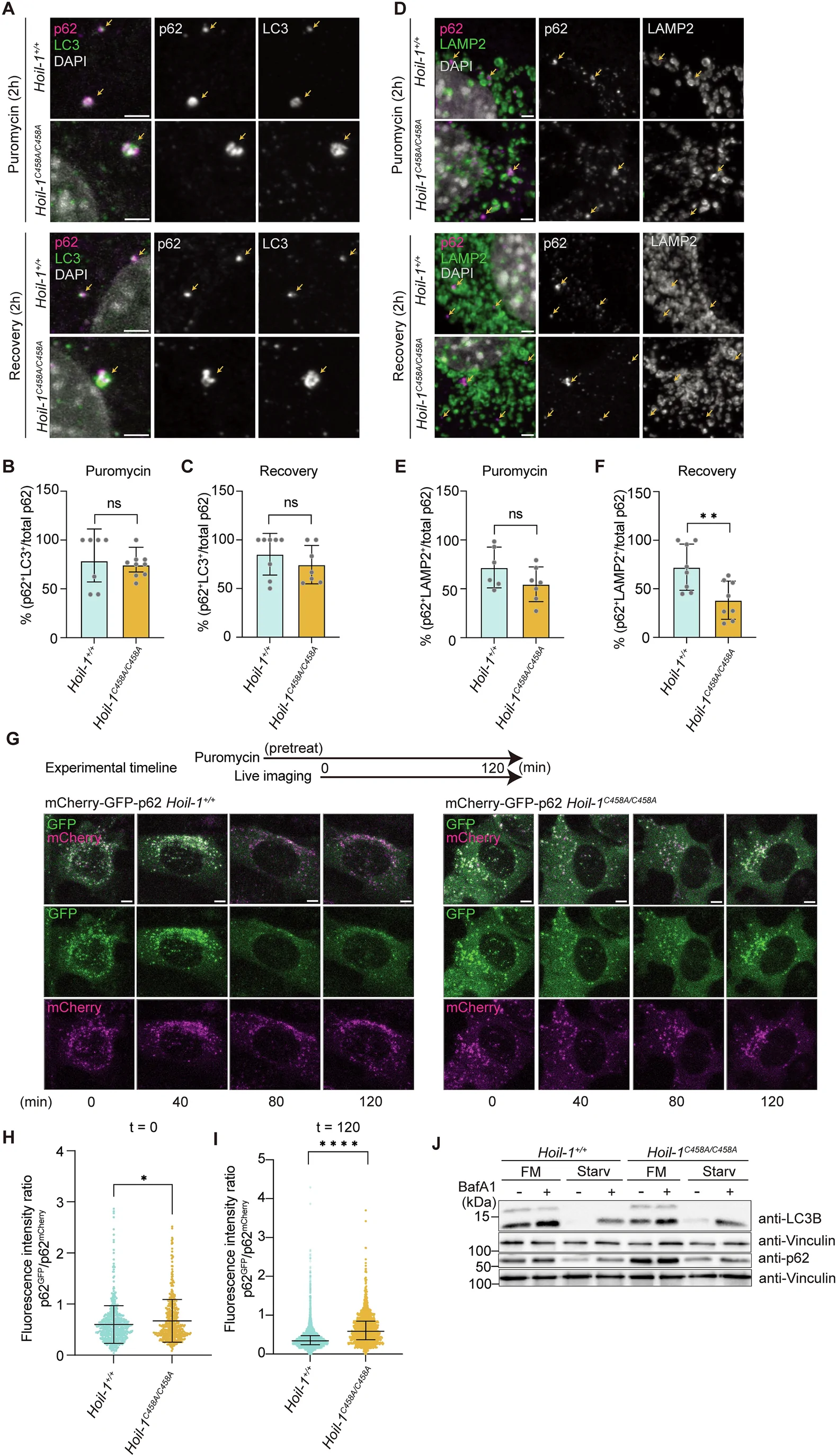
Aggrephagy-mediated clearance of aggregates is compromised in *Hoil-1*^*C458A/C458A*^ MEFs. (**A**) Representative immunofluorescence images showing colocalization of p62 and LC3 in *Hoil-1*^*+/+*^ and *Hoil-1*^*C458A/C458A*^ MEFs following puromycin treatment (5 μg/ml) and after the recovery period, as indicated. Scale bar, 5 μm. Arrows indicate the position of p62-positive puncta across each channel. (**B**, **C**) Quantification of the percentage of p62 puncta colocalized with LC3 following puromycin treatment (**B**) and after recovery (**C**). Data were presented as mean ± SEM from two independent biological experiments (puromycin: *Hoil-1*^*+/+*^, *n* = 7 fields; *Hoil-1*^*C458A/C458A*^, *n* = 8 fields; recovery: *Hoil-1*^*+/+*^, *n* = 8 fields; *Hoil-1*^*C458A/C458A*^, *n* = 8 fields). Statistical significance was determined using an unpaired *t*-test (ns, *P* > 0.05). (**D**) Representative immunofluorescence images showing colocalization of p62 and LAMP2 in *Hoil-1*^*+/+*^ and *Hoil-1*^*C458A/C458A*^ MEFs following puromycin treatment (5 μg/ml, 2 h) and after a 2 h recovery period, as indicated. Scale bar, 5 μm. Arrows indicate the position of p62-positive puncta across each channel. (**E**,** F**). Quantification of the percentage of p62 puncta colocalized with LAMP2 following puromycin treatment (**E**) and after recovery (**F**). Data were presented as mean ± SEM from three independent biological experiments (puromycin: *Hoil-1*^*+/+*^, *n* = 6 fields; *Hoil-1*^*C458A/C458A*^, *n* = 7 fields; recovery: *n* = 7 fields for each genotype). Statistical significance was determined using an unpaired *t*-test (ns, *P* > 0.05; **P = 0.0078). (**G**) Experimental timeline and representative time-lapse images of the mCherry-EGFP-p62 reporter in *Hoil-1*^*+/+*^ and *Hoil-1*^*C458A/C458A*^ MEFs (*n* = 40 cells from three independent biological experiments). MEFs were pretreated with puromycin (5 μg/ml), and p62 puncta formation was confirmed prior to the start of live-cell imaging (*t* = 0). Scale bar, 5 μm. (**H**,** I**) Quantification of the GFP/mCherry fluorescence intensity ratio of the mCherry-EGFP-p62 reporter in *Hoil-1*^*+/+*^ and *Hoil-1*^*C458A/C458A*^ MEFs. The GFP/mCherry ratio was measured at the first frame (*t* = 0 min; (**H**)) and the final frame (*t* = 120 min, 60th frame; (**I**)). Data were presented as median values with interquartile ranges from *n* = 40 cells obtained from three independent biological experiments. Statistical significance was determined using a Mann–Whitney test (*P = 0.0269, ****P < 1.0 × 10^−15^. (**J**) Protein levels of autophagy markers in *Hoil-1*^*+/+*^ and *Hoil-1*^*C458A/C458A*^ MEFs under basal (full medium, FM) and amino acid starvation (Starv, 4 h) conditions, in the presence or absence of bafilomycin A1. Representative immunoblot data from two independent biological experiments. Source data are available online for this figure.

Protein aggregates are removed primarily through selective autophagy called aggrephagy (Bauer et al, 2023; Ichimura et al, 2008). Because *Hoil-1*^*C458A/C458A*^ MEFs accumulated p62^+^/ubiquitin^+^ puncta (Fig. 2B,E) and ProteoStat-stained aggregates (Fig. EV2A–C) under proteotoxic stress, we investigated whether impaired aggrephagy underlies this defect. We first examined the recruitment of key autophagy machinery to p62 bodies under proteotoxic stress using puromycin treatment and subsequent recovery. In both *Hoil-1*^*+/+*^ and *Hoil-1*^*C458A/C458A*^ MEFs, p62 puncta colocalized with LC3, indicating that autophagy initiation and autophagosome assembly at p62 bodies remained intact (Fig. 3A–C). Consistent with earlier observations, *Hoil-1*^*C458A/C458A*^ MEFs displayed larger p62 puncta, but the overall percentage of p62-LC3 overlap was comparable between the genotypes in both the puromycin and recovery phases (Fig. 3A–C).

**Figure EV3:**
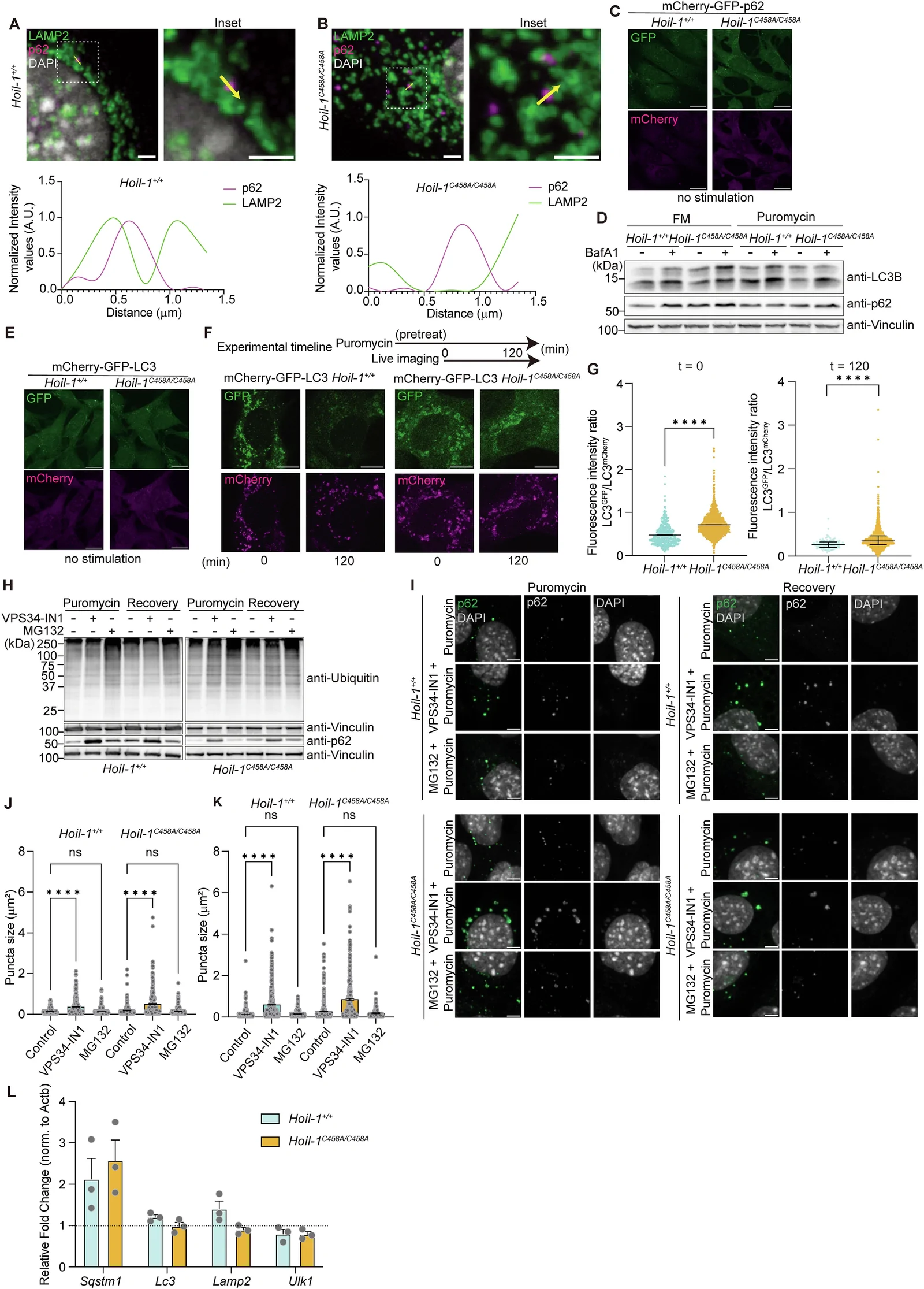
Aggrephagy is selectively impaired in *Hoil-1*^*C458A/C458A*^ MEFs. (**A**,** B**) Fluorescence intensity profiles of p62 and LAMP2 along the line indicated by the arrow in (A) *Hoil-1*^+/+^ and (B) *Hoil-1*^*C458A/C458A*^ MEFs, illustrating the spatial relationship between p62 and LAMP2 signals. Representative images from three independent biological experiments are shown. Scale bar, 2 μm. White dashed boxes indicate the region magnified in the inset. (**C**) Representative fluorescence microscopy images showing mCherry-EGFP-p62 in untreated *Hoil-1*^+/+^ and *Hoil-1*^*C458A/C458A*^ MEFs. Representative images from two independent biological experiments are shown. Scale bar, 20 μm. (**D**) Immunoblot analysis of autophagy markers in *Hoil-1*^+/+^ and *Hoil-1*^*C458A/C458A*^ MEFs cultured in full medium (FM) or following puromycin treatment (2 h), in the presence or absence of bafilomycin A1 (BafA1). Representative data from two independent biological experiments. (**E**) Representative fluorescence microscopy images of *Hoil-1*^+/+^ and *Hoil-1*^*C458A/C458A*^ MEFs expressing mCherry-EGFP-LC3B under basal (unstimulated) conditions. Scale bar, 20 μm. (**F**) Representative time-lapse imaging of the mCherry-EGFP-LC3 reporter in *Hoil-1*^+/+^ and *Hoil-1*^*C458A/C458A*^ MEFs following puromycin treatment (5 μg/ml). Cells were pretreated with puromycin prior to live-cell imaging to ensure the formation of LC3-positive puncta before image acquisition (*n* = 40 cells from three independent biological experiments). Scale bar, 10 μm. (**G**) Quantification of the GFP/mCherry fluorescence intensity ratio of the mCherry-EGFP-LC3 reporter shown in (**F**). Ratios were measured at the start of imaging (*t* = 0 min) and at the final time point (*t* = 120 min). Data were presented as mean ± SEM from three independent biological experiments. Statistical significance was determined using a Mann–Whitney test (*t* = 0, ****P < 1.0×10^-15^, *t* = 120, ****P = 1.5 × 10^−12^). (**H**) *Hoil-1*^+/+^ and *Hoil-1*^*C458A/C458A*^ MEFs were pretreated with VPS34-IN1 (5 μM, 16 h) or MG132 (5 μM, 2 h), followed by puromycin treatment (5 μg/ml, 2 h) and a 2 h recovery period in puromycin-free medium. VPS34-IN1 or MG132 was maintained throughout both the puromycin treatment and recovery phases. Cell lysates were subsequently analyzed by immunoblotting using antibodies against p62 and ubiquitin. Vinculin was used as a loading control. Representative data from two independent biological experiments. (**I**) *Hoil-1*^+/+^ and *Hoil-1*^*C458A/C458A*^ MEFs were pretreated with VPS34-IN1 (5 μM, 16 h) or MG132 (5 μM, 2 h), followed by puromycin treatment (5 μg/ml, 2 h) and a 2 h recovery period in puromycin-free medium. VPS34-IN1 or MG132 was maintained throughout both the puromycin treatment and recovery phases. Cells were subsequently analyzed by immunofluorescence staining for p62. Representative data from two independent biological experiments. Scale bar, 5 μm. (**J**,** K**). Quantification of p62 body size in puromycin-treated (**J**) and recovery (**K**) conditions in *Hoil-1*^+/+^ and *Hoil-1*^*C458A/C458A*^ MEFs. Analyses were performed using samples treated under the same conditions as those shown in (**I**). Data were presented as mean ± SEM. Statistical significance was assessed using a Kruskal–Wallis test followed by Dunn’s multiple-comparison test ((**J**) ****P = 4.0 × 10^−8^, (**K**) ****P < 1.0 × 10^−15^). Approximately 30 cells per condition were analyzed across two independent biological experiments. (**L**) qRT-PCR analysis of autophagy-related gene expression in *Hoil-1*^+/+^ and *Hoil-1*^*C458A/C458A*^ MEFs following puromycin treatment (5 μg/ml). Gene expression levels were normalized to *Actb* and are shown relative to untreated controls. Data were presented as mean relative fold change ± SEM from three independent biological experiments. No significant differences were observed between genotypes.

To evaluate whether p62 bodies are degraded by lysosomes, we examined the colocalization of p62 bodies with LAMP2, a lysosomal membrane marker. Following puromycin treatment, p62 bodies in both *Hoil-1*^*+/+*^ and *Hoil-1*^*C458A/C458A*^ MEFs were surrounded by LAMP2-positive structures (Figs. 3D,E and EV3A,B). In Fig. EV3A,B, representative p62 puncta from *Hoil-1*^*+/+*^ and *Hoil-1*^*C458A/C458A*^ MEFs during the recovery phase were selected for line-scan fluorescence analysis of p62 and LAMP2 signals. These analyses show that, in *Hoil-1*^*+/+*^ MEFs, the residual p62 puncta were typically enclosed within or surrounded by LAMP2-positive vesicles. In contrast, in *Hoil-1*^*C458A/C458A*^ MEFs, p62 puncta are rarely associated with LAMP2-positive compartments, suggesting impaired lysosomal targeting or degradation. During recovery, *Hoil-1*^*+/+*^ MEFs showed efficient recruitment of LAMP2 to p62 puncta and marked reduction in p62-body size (Fig. 3F). In contrast, *Hoil-1*^*C458A/C458A*^ MEFs accumulated enlarged p62 bodies that lacked adjacent LAMP2-positive structures, indicating failure of autophagosome–lysosome fusion or autolysosome maturation.

To directly monitor the p62-dependent autophagic flux, we generated reporter MEF lines stably expressing mCherry-EGFP–p62 (Pankiv et al, 2007). In this system, GFP fluorescence is quenched under acidic conditions, such as in lysosomes, whereas mCherry fluorescence remains stable, allowing distinction between autophagosomes (GFP^+^ mCherry^+^) and autolysosomes (GFP^–^ mCherry^+^). In the *Hoil-1*^*+/+*^ reporter line, puromycin treatment triggered a progressive loss of GFP signal, indicating that p62 bodies were delivered to lysosomes (Figs. EV3C and 3G). In contrast, *Hoil-1*^*C458A/C458A*^ MEFs accumulated GFP^+^ mCherry^+^ p62 puncta, indicating that lysosomal degradation was blocked (Figs. EV3C and 3G). Quantification of the GFP/mCherry ratios confirmed significantly elevated levels of GFP retention in *Hoil-1*^*C458A/C458A*^ reporter MEFs (Fig. 3H,I). Although these reporter-based observations were clear differences between *Hoil-1*^*C458A/C458A*^ MEFs and *Hoil-1*^*+/+*^ MEFs, assessment of autophagic flux by immunoblotting analysis of total cell lysates showed little difference between the two genotypes. These findings suggest that the observed effects occur within specific subcellular compartments and are therefore not readily detected by bulk biochemical analyses (Fig. EV3D).

To further validate these findings, we generated mCherry-EGFP–LC3 reporter MEFs (Ebner et al, 2018; Kimura et al, 2007). Although the difference was milder than p62-reporter-based data, *Hoil-1*^*C458A/C458A*^ MEFs treated with puromycin accumulated dual-positive (GFP^+^ mCherry^+^) LC3 vesicles, indicating defective autophagic flux and impaired autophagosome maturation specifically during aggregate clearance (Fig. EV3E–G). Of note, bulk autophagic flux induced by amino acid starvation was not significantly altered in *Hoil-1*^*C458A/C458A*^ MEFs in comparison to *Hoil-1*^*+/+*^ MEFs, as determined by immunoblotting analysis (Fig. 3J). We next analyzed the effects of a macroautophagy inhibitor (a VPS34 inhibitor) and a proteasome inhibitor, MG132, on p62-body clearance in MEFs. The efficacy of these inhibitors was first validated by monitoring p62 and ubiquitin levels by immunoblotting (Fig. EV3H). Microscopic analysis revealed that the VPS34 inhibition further increased the size of p62 bodies in both *Hoil-1*^*+/+*^ and *Hoil-1*^*C458A/C458A*^ MEFs (Fig. EV3I–K). In contrast, MG132 treatment had little effect, suggesting that p62-body clearance is largely dependent on the autophagy pathway rather than the proteasome (Fig. EV3I–K). Collectively, these findings indicate that loss of HOIL-1 catalytic activity results in a partial impairment of the autophagy-dependent clearance of large p62 bodies in MEFs.

Together, these data suggest that although early autophagy components, such as LC3, are effectively recruited to p62 bodies, the late stages of aggrephagy, particularly autolysosome formation at the site, are compromised in *Hoil-1*^*C458A/C458A*^ MEFs. Importantly, the mRNA level of autophagy-related genes was comparable between *Hoil-1*^*+/+*^ and *Hoil-1*^*C458A/C458A*^ MEFs (Fig. EV3L). Thus, HOIL-1 catalytic activity appears to be dispensable for general autophagy but is specifically required for efficient aggregate-selective autophagy.

### Transition in condensate properties in *Hoil-1*^*C458A/C458A*^ MEFs

**Figure EV4:**
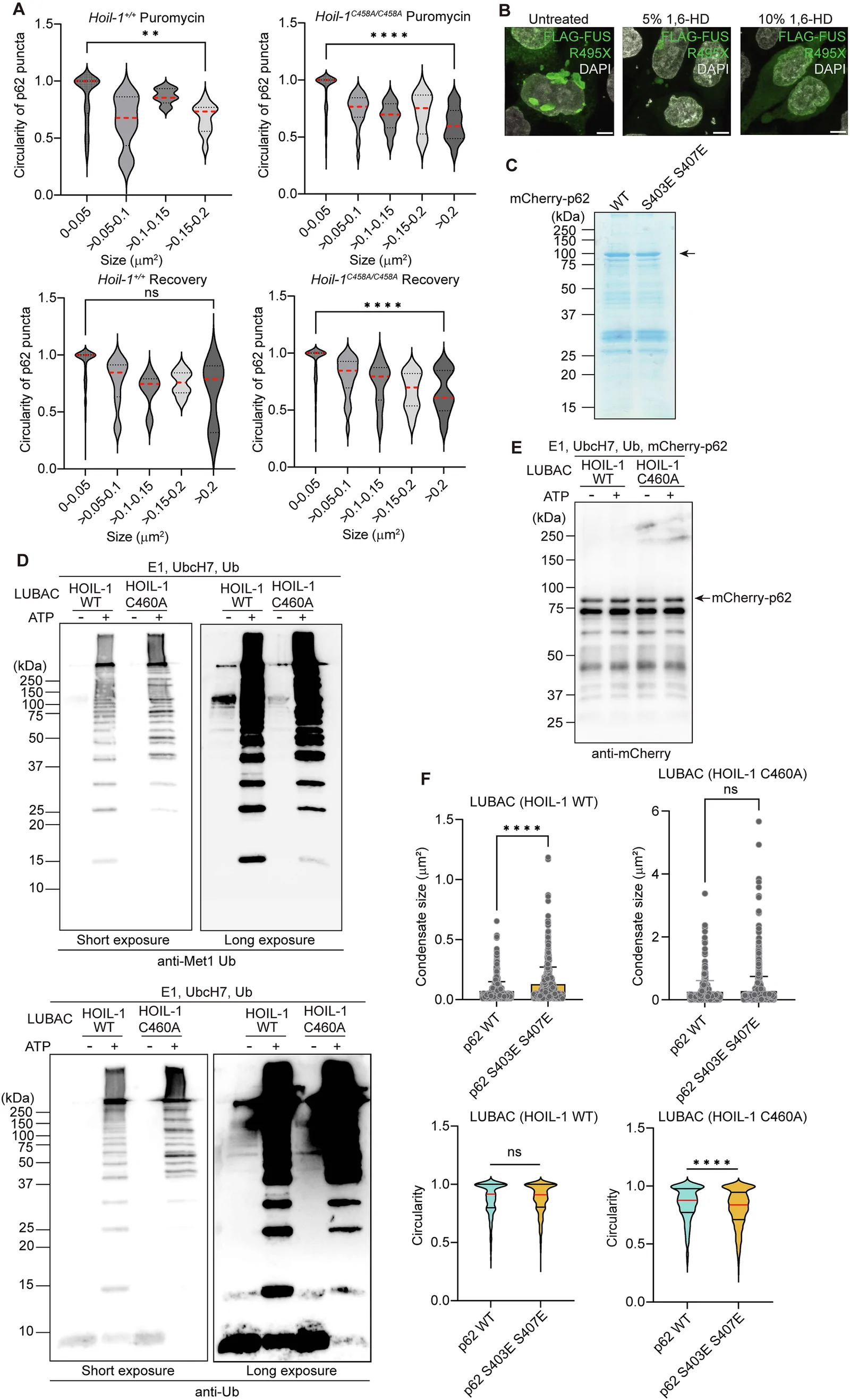
Met1-linked ubiquitin chains alter the material properties of p62 condensates. (**A**) Quantification of p62 body circularity in *Hoil-1*^+/+^ and *Hoil-1*^*C458A/C458A*^ MEFs following puromycin treatment and after the recovery period. Data were presented from three independent biological experiments. Statistical significance was assessed using a Kruskal–Wallis test (*Hoil-1*^+/+^ Puromycin: **, *P* = 0.0097, *Hoil-1*^*C458A/C458A*^ Puromycin: ****P = 6.9 × 10^−7^, *Hoil-1*^+/+^ Recovery: ns, *P* = 0.1467, *Hoil-1*^*C458A/C458A*^ Recovery: ****P = 2.3 × 10^−7^). (**B**) Representative images demonstrating the dissolution of FUS R495X condensates in HEK293T cells following treatment with 5% or 10% 1,6-hexanediol (1,6-HD). Scale bar, 10 μm. (**C**) SDS-PAGE analysis of purified recombinant mCherry-p62 proteins visualized by InstantBlue staining. Arrows indicate mCherry-p62 WT and the phosphomimetic mCherry-p62 S403E/S407E mutant. (**D**) Immunoblot analysis of in vitro ubiquitination reactions comparing ubiquitin chains assembled by wild-type LUBAC and LUBAC containing HOIL-1 C460A. Met1-linked and total ubiquitin chains were detected using anti-Met1 ubiquitin and anti-ubiquitin antibodies, respectively. (**E**) Immunoblot analysis of mCherry-p62 in parallel reaction mixtures corresponding to the in vitro LLPS assays shown in Fig. 4F, demonstrating the absence of detectable p62 ubiquitination under the assay conditions used. (**F**) Direct comparison of condensate size and circularity between mCherry-p62 WT and the phosphomimetic mCherry-p62 S403E/S407E mutant using the datasets shown in Fig. 4F–K. Condensate size is presented as mean ± SEM, whereas circularity is presented as median (red line) with interquartile range. Statistical significance was assessed using a Mann–Whitney test (HOIL-1 WT Condensate size: ****P < 1.0 × 10^−15^; HOIL-1 C460A Condensate size: ns, *P* = 0.6951; HOIL-1 WT Circularity: ns, *P* = 0.1619; HOIL-1 C460A Circularity: ****P = 5.1 × 10^−5^).

To determine whether the altered biophysical states of p62-positive aggregates contribute to clearance defects in *Hoil-1*^*C458A/C458A*^ MEFs, we first assessed the circularity of endogenous p62 puncta (Ray et al, 2020; Tomaszewski et al, 2023). Soluble condensates (also termed liquid-like biomolecular condensates) typically display near-perfect circularity (close to 1), whereas solid aggregates exhibit irregular and non-circular shapes. Stratification of p62 puncta by size in the recovery samples of *Hoil-1*^*C458A/C458A*^ MEFs revealed a progressive decrease in circularity with increasing puncta size, consistent with a shift toward more rigid and solid-like structures (Fig. EV4A).

**Figure 4:**
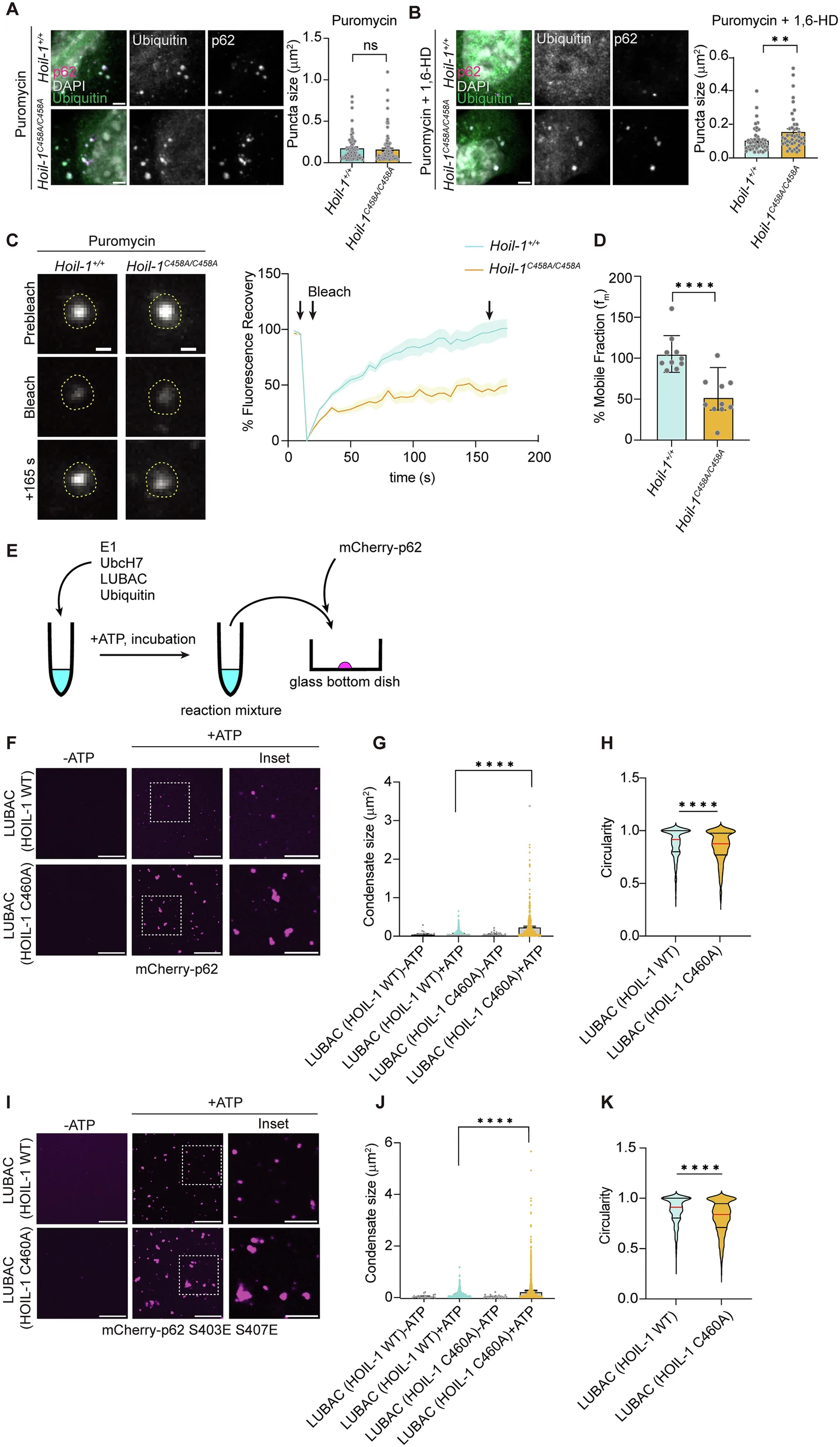
Transition of condensate property by catalytically inactive mutations in HOIL-1 in MEFs and in vitro. (**A**,**B**) Representative immunofluorescence images of p62 and ubiquitin in *Hoil-1*^*+/+*^ and *Hoil-1*^*C458A/C458A*^ MEFs following (**A**) puromycin treatment (5 μg/ml, 2 h) or (**B**) puromycin treatment followed by 5% 1,6-hexanediol (1,6-HD). These experiments were performed to assess the dissolution of puromycin-induced p62 condensates upon 1,6-HD treatment. Representative images from three independent biological experiments are shown. Scale bar, 2 μm. Quantification of p62 condensate size under puromycin treatment alone or following puromycin plus 5% 1,6-HD treatment in *Hoil-1*^*+/+*^ and *Hoil-1*^*C458A/C458A*^ MEFs. Data are presented as mean ± SEM from three independent biological experiments (puromycin: *Hoil-1*^*+/+*^, *n* = 70 cells; *Hoil-1*^*C458A/C458A*^, *n* = 97 cells; puromycin + 1,6-HD: *Hoil-1*^*+/+*^, *n* = 51 cells; *Hoil-1*^*C458A/C458A*^, *n* = 44 cells). Statistical significance was determined using a Mann–Whitney test (ns, *P* > 0.05; **P = 0.0071). (**C**) Representative fluorescence recovery after photobleaching (FRAP) analysis of GFP-p62 condensates in *Hoil-1*^*+/+*^ and *Hoil-1*^*C458A/C458A*^ MEFs. Representative images are shown before photobleaching, immediately after photobleaching (*t* = 15 s), and during recovery (*t* = 165 s). Graphs show the mean fluorescence recovery (%) ±SEM over time. Data were obtained from *n* = 9 bleaching events from three independent biological experiments. Scale bar, 0.5 μm. Arrows indicate the time points corresponding to the representative images (*t* = 5 s, *t *= 15 s, and *t* = 165 s). Yellow dashed borders indicate the location of GFP-p62 puncta. (**D**) Quantification of the mobile fraction (f_m_) of GFP-p62 condensates in *Hoil-1*^*+/+*^ and *Hoil-1*^*C458A/C458A*^ MEFs determined by FRAP analysis. Data were presented as mean ± SD from three independent biological experiments. Statistical significance was assessed using an unpaired *t*-test (****P = 1.2 × 10^−5^). (**E**) Experimental scheme of the in vitro LLPS assay. mCherry-p62 was incubated with products generated by the in vitro ubiquitination reaction, and condensate formation was analyzed by confocal microscopy. (**F**) Representative fluorescence images from the in vitro LLPS assay using mCherry-p62 WT incubated with Met1-linked ubiquitin chains generated by either wild-type LUBAC [LUBAC (HOIL-1 WT)] or LUBAC containing the catalytically inactive HOIL-1 mutant [LUBAC (HOIL-1 C460A)]. Scale bar, 10 μm; inset, 5 μm. White dashed boxes indicate the region magnified in the inset. (**G**,** H**) Quantification of (**G**) condensate size and (**H**) condensate circularity of mCherry-p62 assemblies formed in the in vitro LLPS assay shown in (**F**). For (**G**), data were presented as mean ± SEM and were analyzed using a Kruskal–Wallis test with Dunn’s multiple-comparison test (****P < 1.0 × 10^−15^). For (**H**), data were presented as median (red line) with interquartile range and were analyzed using a Mann–Whitney test (****P = 5.5 × 10^−6^). Data were from three independent experiments. (**I**) Representative fluorescence images from the in vitro LLPS assay using the phosphomimetic mCherry-p62 S403E/S407E mutant incubated with Met1-linked ubiquitin chains generated by either wild-type LUBAC [LUBAC (HOIL-1 WT)] or LUBAC containing the catalytically inactive HOIL-1 mutant [LUBAC (HOIL-1 C460A)]. Scale bar, 10 μm; inset, 5 μm. White dashed boxes indicate the region magnified in the inset. (**J**,** K**) Quantification of (**J**) condensate size and (**K**) condensate circularity of mCherry-p62 S403E/S407E assemblies formed in the in vitro LLPS assay shown in (**I**). For (**J**), data were presented as mean ± SEM and were analyzed using a Kruskal–Wallis test with Dunn’s multiple-comparison test (****P = 3.0 × 10^−9^). For (**K**), data were presented as median (red line) with interquartile range and were analyzed using a Mann–Whitney test (****P < 1.0 × 10^−15^). Data were from three independent experiments. Source data are available online for this figure.

To evaluate whether these structures represent solid aggregates, we examined their sensitivity to 1,6-hexanediol (1,6-HD), a chemical disruptor of weak hydrophobic interactions. As a positive control, FUS R495X, which forms 1,6-HD -sensitive condensates (Yamazaki et al, 2018) was readily dispersed by 1,6-HD in human embryonic kidney 293 T (HEK293T) cells (Fig. EV4B). In *Hoil-1*^*+/+*^ MEFs, puromycin-induced p62^+^ ubiquitin^+^ puncta were efficiently dissolved by 1,6-HD (Fig. 4A,B). However, in *Hoil-1*^*C458A/C458A*^ MEFs, a substantial fraction of p62 puncta persisted after treatment, indicating reduced 1,6-HD sensitivity and supporting a transition to more solid-like structures (Fig. 4A,B). Circularity and 1,6-HD sensitivity capture distinct and complementary properties, and aggregate shape alone should not be interpreted as a direct measure of liquidity.

Next, we evaluated the dynamic properties of these condensates using fluorescence recovery after photobleaching (FRAP). In puromycin-treated MEFs, in which GFP-p62 was stably expressed, GFP^+^ puncta showed robust fluorescence recovery in *Hoil-1*^*+/+*^ MEFs, consistent with rapid internal molecular exchange. In contrast, GFP-p62 puncta in *Hoil-1*^*C458A/C458A*^ MEFs showed markedly slower and incomplete recovery, indicative of reduced molecular mobility and a shift toward a less dynamic, solid state (Fig. 4C,D).

We and others demonstrated that, in vitro, LUBAC containing a point mutation at HOIL-1 C460 catalytic site (Fuseya et al, 2020; McCrory et al, 2022) produces Met1-linked ubiquitin chains more efficiently than wild-type LUBAC (Rodriguez Carvajal et al, 2021). This altered activity leads to the depletion of short ubiquitin chains and the absence of oxyester-linked ubiquitin species, which are typically generated by wild-type LUBAC. Given that LUBAC containing the HOIL-1 C460A mutant produces aberrant Met1-linked ubiquitin chains in vitro, we investigated how these chains influence the aggregate properties. We utilized Met1-linked chains produced in vitro by either HOIL-1 wild-type or HOIL-1 C460A-containing LUBAC in liquid-liquid phase separation (LLPS) reconstitution assays using recombinant mCherry-p62 (Figs. 4E and EV4C–E). Under these conditions, ubiquitination of mCherry-p62 was not detected, ruling out a contribution of p62 ubiquitination to the observed effects (Fig. EV4E). Met1-linked chains synthesized by HOIL-1 C460A-containing LUBAC promoted the formation of large, irregular, non-spherical p62 condensates, in contrast to the smaller, more rounded condensates formed by LUBAC wild-type-derived ubiquitin chains (Fig. 4F–H). Interestingly, p62 with phosphomimetic mutations (S403E/S407E) in its UBA domain, known for its enhanced ubiquitin affinity (Lim et al, 2015), formed large, irregular condensates in the presence of ubiquitin chains generated by LUBAC containing HOIL-1 C460A (Figs. 4I–K and EV4F). Comparison of condensate size and circularity between wild-type p62 and the phosphomimetic p62 mutant using the same dataset revealed that p62 condensates formed in the presence of HOIL-1 C460A-generated ubiquitin chains were less circular (Fig. EV4F). These findings suggest that interactions between p62 and ubiquitin chains contribute to the regulation of condensate morphology. Although we did not observe LUBAC-dependent ubiquitination of p62 in an identical condition of these assays, it would be critical to identify target substrates in the control of aggregate clearance in the future.

Collectively, these findings indicate that HOIL-1 catalytic activity plays a crucial role in maintaining the proper physical state of the Met1-linked ubiquitin-dependent p62 condensates.

### Elevated Met1-linked ubiquitin chains contribute to solid-like aggregate formation

**Figure 5:**
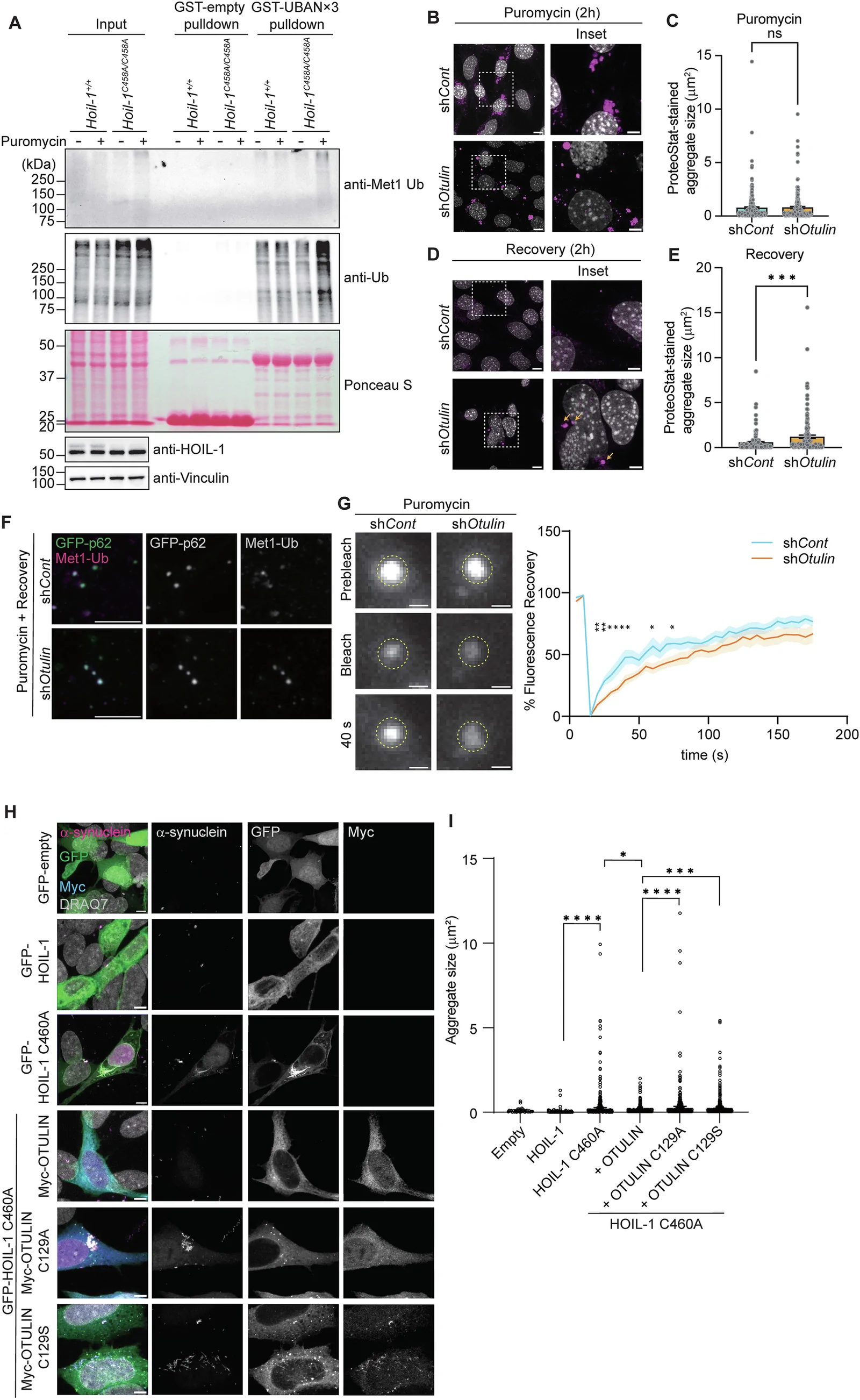
Accumulation of Met1-linked ubiquitin chains upon proteotoxic stress in *Hoil-1*^*C458A/C458A*^ MEFs. (**A**) GST-empty and GST-UBAN×3 pull-downs were performed using lysates from *Hoil-1*^*+/+*^ and *Hoil-1*^*C458A/C458A*^ MEFs, followed by immunoblot analysis as indicated. Ponceau S staining (bottom panel) is shown as a loading control. Representative data from three independent biological experiments. (**B**) Representative images of ProteoStat-stained aggregates in sh*Control* and sh*Otulin* MEFs following puromycin treatment (5 μg/ml). Representative images from three independent biological experiments are shown. Scale bar, 5 μm. White dashed boxes indicate the region magnified in the inset. (**C**) Quantification of aggregate size in sh*Control* and sh*Otulin* MEFs following puromycin treatment. Data were presented as mean ± SEM and were analyzed using a Mann–Whitney test (ns, *P* > 0.05). More than 60 cells per condition were analyzed from two independent biological experiments (sh*Control*: *n* = 211 puncta; sh*Otulin*: *n* = 166 puncta). (**D**) Representative images of ProteoStat-stained aggregates in sh*Control* and sh*Otulin* MEFs following puromycin treatment and a subsequent recovery period, as indicated. Representative images from three independent biological experiments are shown (sh*Control*: *n* = 116 puncta; sh*Otulin*: *n* = 130 puncta). Scale bar, 5 μm. Arrows indicate the ProteoStat-positive puncta in sh*Otulin* MEFs. White dashed boxes indicate the region magnified in the inset. (**E**) Quantification of aggregate size in sh*Control* and sh*Otulin* MEFs under recovery conditions. Data were presented as mean ± SEM and were analyzed using a Mann–Whitney test (***P = 0.0002). More than 65 cells per condition were analyzed from two independent biological experiments. (**F**) sh*Control* and sh*Otulin* MEFs expressing GFP-p62 were treated with puromycin (5 μg/ml) for 2 h, followed by a 2 h recovery period in puromycin-free medium. Representative immunofluorescence images showing colocalization of GFP-p62 and Met1-linked ubiquitin chains are shown. Representative images from two independent biological experiments. Scale bar, 5 μm. (**G**) Representative fluorescence recovery after photobleaching (FRAP) analysis of GFP-p62 condensates in sh*Control* and sh*Otulin* MEFs. Representative images are shown before photobleaching (*t* = 5 s), immediately after photobleaching (*t* = 15 s), and during recovery (*t* = 40 s). Graphs show the mean fluorescence recovery (%) ±SEM over time. Scale bar, 0.5 μm. Statistical significance at each time point was assessed using a two-sided Welch’s *t*-test as previously described (Kageyama et al, 2021): **P = 0.0009254 (*t* = 20 s); **P = 0.004062 (*t* = 25 s); *P = 0.032709 (*t* = 30 s); *P = 0.020494 (*t* = 35 s); *P = 0.028078 (*t* = 40 s); *P = 0.023970 (*t* = 45 s); *P* = 0.042569 (*t* = 60 s); and, *P* = 0.029833 (*t* = 75 s). Data were obtained from *n* = 9 bleaching events across five independent biological experiments. Yellow dashed borders indicate the location of GFP-p62 puncta. (**H**) Representative immunofluorescence images showing α-synuclein aggregates and GFP-HOIL-1 or GFP-HOIL-1 C460A co-expressed with Myc-tagged OTULIN, OTULIN C129A, or OTULIN C129S, as indicated, in SH-SY5Y cells. Representative images from two independent biological experiments are shown. Scale bar, 5 μm. (**I**) Quantification of α-synuclein aggregate size from the conditions shown in (**H**). Data were presented as mean ± SEM. Statistical significance was determined using a Kruskal–Wallis test followed by Dunn’s multiple-comparison test (HOIL-1 vs HOIL-1 C460A: ****P < 1.0 × 10^−15^; HOIL-1 C460 vs HOIL-1 C460A + OTULIN: *P = 0.0311; HOIL-1 C460A + OTULIN vs HOIL-1 C460A + OTULIN C129A: ****P = 8.0 × 10^−10^, HOIL-1 C460A + OTULIN vs HOIL-1 C460A + OTULIN C129S: ***P = 0.00025). More than 250 cells per condition were analyzed from two independent biological experiments. Source data are available online for this figure.

**Figure EV5:**
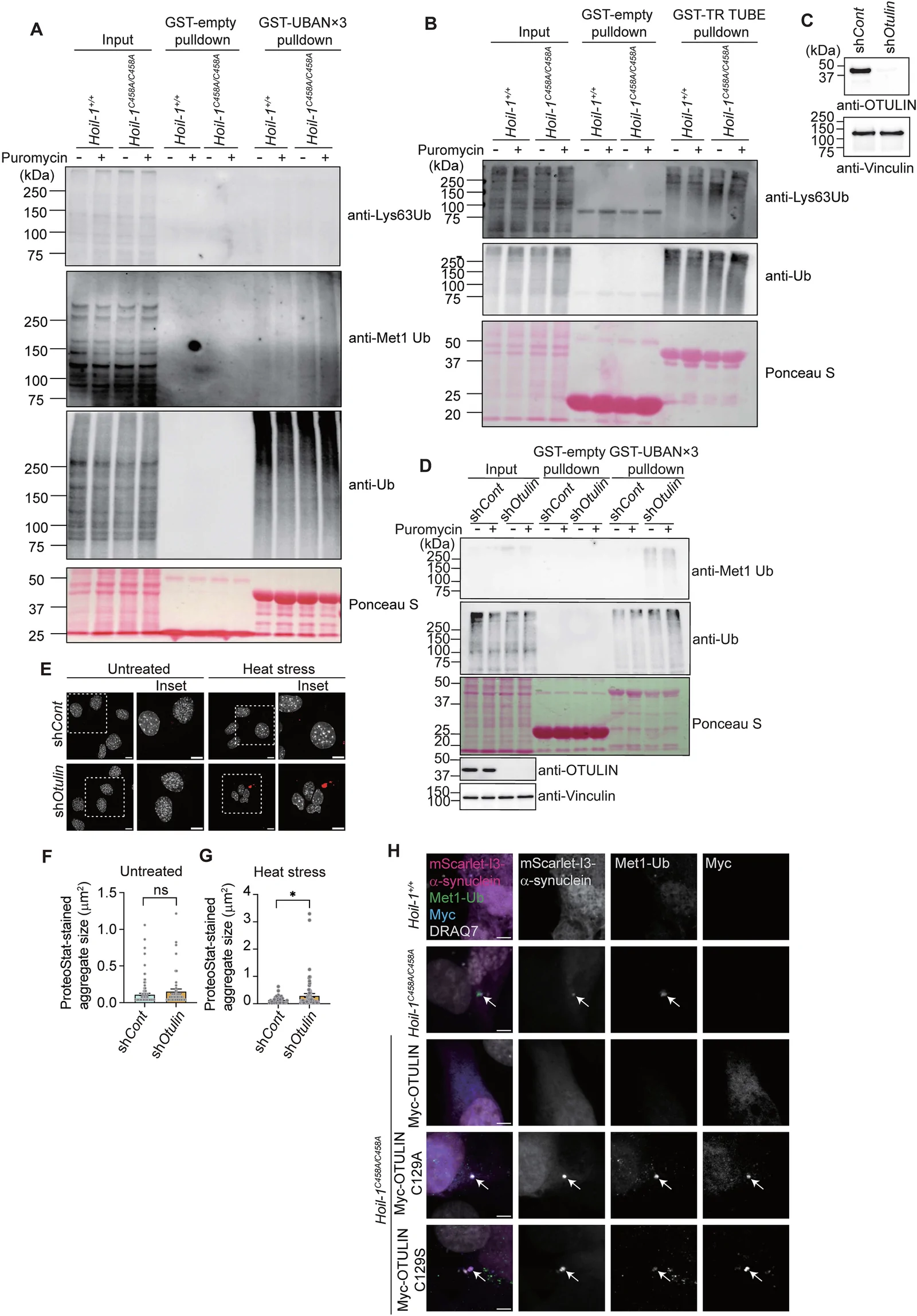
Elevated Met1-linked ubiquitination promotes persistent aggregate accumulation. (**A**) GST and GST-UBAN×3 pull-down assays were performed using lysates from *Hoil-1*^+/+^ and *Hoil-1*^*C458A/C458A*^ MEFs with or without puromycin treatment, followed by immunoblot analysis. Lys63-linked ubiquitin chains, Met1-linked ubiquitin chains, and total ubiquitin were detected using linkage-specific or pan-ubiquitin antibodies, as indicated. Ponceau S staining (bottom panel) is shown as a loading control. Representative data from two independent biological experiments. (**B**) GST and GST-TR-TUBE pull-down assays were performed using lysates from *Hoil-1*^+/+^ and *Hoil-1*^*C458A/C458A*^ MEFs, followed by immunoblot analysis of the indicated proteins. Ponceau S staining (bottom panel) is shown as a loading control. Representative data from three independent biological experiments. (**C**) Immunoblot analysis confirming OTULIN knockdown in sh*Otulin* MEFs compared with sh*Control* MEFs. Vinculin was used as a loading control. (**D**) GST and GST-UBAN×3 pull-down assays were performed using lysates from sh*Control* and sh*Otulin* MEFs, followed by immunoblot analysis. Met1-linked ubiquitin chains (top panel) and total ubiquitin (middle panel) were examined in the presence or absence of puromycin treatment (5 μg/ml, 2 h). Ponceau S staining (bottom panel) is shown as a loading control for pull-down samples. OTULIN knockdown was confirmed using an anti-OTULIN antibody, and vinculin was used as a loading control for input samples. Representative data from three independent biological experiments. (**E**) Representative images of ProteoStat-stained aggregates in sh*Control* and sh*Otulin* MEFs subjected to heat stress (42 °C). Representative images from three independent biological experiments are shown. Scale bar, 5 μm. White dashed boxes indicate the region magnified in the inset. (**F**,** G**) Quantification of aggregate size in sh*Control* and sh*Otulin* MEFs under (**F**) untreated and (**G**) heat stress conditions. Data were presented as mean ± SEM from three independent biological experiments (*N* = 3). Statistical significance was assessed using the Mann–Whitney test (ns, *P* > 0.05; *P = 0.0354). (**H**) Representative fluorescence microscopy images showing α-synuclein aggregates and Met1-linked ubiquitin chains in *Hoil-1*^+/+^ and *Hoil-1*^*C458A/C458A*^ MEFs transiently expressing Myc-tagged OTULIN, OTULIN C129A, or OTULIN C129S, as indicated. Representative images from three independent biological experiments are shown. Scale bar, 5 μm. Arrows indicate the position of α-synuclein-positive puncta across each channel.

Our previous findings indicated that the levels of Met1-linked ubiquitin chains were elevated in *Hoil-1*^*C458A/C458A*^ MEFs upon TNF stimulation (Rodriguez Carvajal et al, 2021). To determine whether increased levels of Met1-linked ubiquitin chains contribute to aggregate formation, we enriched Met1-linked chains from *Hoil-1*^*+/+*^ and *Hoil-1*^*C458A/C458A*^ MEFs treated with puromycin using GST-tagged triplicates of the UBAN domain of TNIP1/ABIN1 (GST-UBAN×3), which specifically binds to Met1-linked ubiquitin chains (Fennell et al, 2020). *Hoil-1*^*C458A/C458A*^ MEFs accumulated more Met1-linked chains upon puromycin treatment than *Hoil-1*^*+/+*^ MEFs, indicating that Met1-linked ubiquitin chains were more efficiently generated in HOIL-1 catalytic mutant cells under proteotoxic stress (Fig. 5A). Although distinct ubiquitin chain linkage types are often associated with aggregate formation, we did not detect any differences in Lys63-linked ubiquitin chains under these conditions. Specifically, analysis of cellular lysates enriched using either GST-UBAN×3 or the pan-ubiquitin enrichment reagent GST-TR-TUBE revealed comparable levels of Lys63-linked ubiquitin chains (Fig. EV5A,B).

Next, we stably knocked down OTULIN, a deubiquitinase specific for Met1-linked ubiquitin chains, in MEFs (Fig. EV5C) and performed GST pulldown following puromycin treatment. Although basal Met1-linked chains were already elevated in sh*Otulin* MEFs, we observed little difference in the signal of Met1-linked ubiquitin chains after puromycin treatment; nevertheless, the enriched Met1-linked chains were clearly higher than those in control MEFs (Fig. EV5D). Supporting our hypothesis that elevated Met1-linked ubiquitin chains sensitize cells to form larger aggregates, sh*Otulin* MEFs developed significantly larger p62-positive puncta during the puromycin recovery phase and under heat stress, mirroring the phenotype observed in *Hoil-1*^*C458A/C458A*^ MEFs (Figs. 5B–E and EV5E–G). Puromycin-induced p62 bodies in these cells colocalized with Met1-linked ubiquitin chains (Fig. 5F) and exhibited slower fluorescence recovery in FRAP assays, particularly during the early phase of recovery (Fig. 5G). These findings support an important role for Met1-linked ubiquitin chains in regulating the properties of protein aggregates and condensates.

To further test whether excessive Met1-linked chains are a major driver of aggregate accumulation, we examined whether OTULIN overexpression could ameliorate the effects induced by the HOIL-1 catalytically inactive mutant (Fig. 5H,I). To this end, we co-expressed GFP, GFP-HOIL-1, or GFP-HOIL-1 C460A with α-synuclein in SH-SY5Y cells. Consistent with our earlier observations, α-synuclein aggregates in HOIL-1 C460A-expressing cells were significantly larger than those in cells expressing wild-type HOIL-1 (Fig. 5H,I). In the HOIL-1 C460A background, we overexpressed wild-type OTULIN or its catalytically inactive mutants (OTULIN C129A or C129S). This allowed us to assess whether reducing the excess Met1-linked chains could diminish the size of the aggregates formed in these cells. We observed a significant reduction in α-synuclein aggregate size upon wild-type OTULIN overexpression, emphasizing the contribution of Met1-linked chains to driving aggregate accumulation. Similar trends were observed in *Hoil-1*^*+/+*^ and *Hoil-1*^*C458A/C458A*^ MEFs (Fig. EV5H).

**Figure 6:**
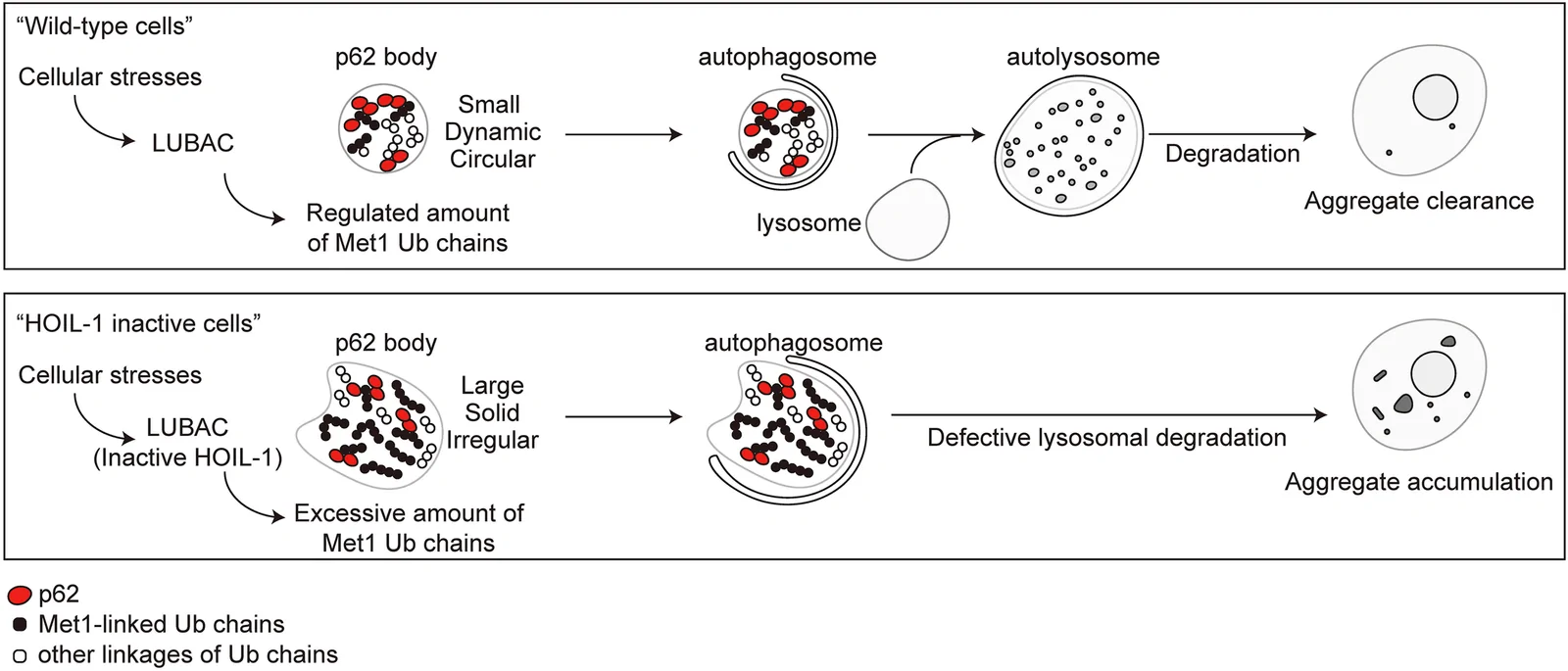
Model for impaired aggregate clearance in cells expressing catalytically inactive HOIL-1. Under proteotoxic stress, misfolded proteins are decorated with ubiquitin chains, including HOIL-1-dependent oxyester-branched Met1-linked ubiquitin chains generated by LUBAC. These ubiquitinated substrates are concentrated within p62 bodies, which form dynamic, liquid-like condensates. p62 condensates are subsequently cleared through aggrephagy, involving autophagosome recruitment, lysosomal fusion, and degradation within autolysosomes, thereby maintaining cellular proteostasis. In cells expressing the catalytically inactive HOIL-1 mutant (*Hoil-1*^*C458A/C458A*^), HOIP-mediated Met1-linked ubiquitination becomes excessive. The resulting accumulation of Met1-linked ubiquitin chains promotes condensation and reduces the dynamic properties of p62 bodies, driving their transition toward a more solid-like state. These rigid condensates are inefficiently processed by aggrephagy, leading to the persistence of insoluble protein aggregates and impaired proteostasis.

In summary, these results support a model in which the excessive expression of Met1-linked ubiquitin chains in cells promotes the formation of more solid and less dynamic protein assemblies, potentially contributing to impaired aggregate clearance (Fig. 6).

## Discussion

Our findings underscore the importance of a precise balance between ubiquitination and deubiquitination of Met1-linked ubiquitin chains in cellular proteostasis. Specifically, the catalytic activity of HOIL-1 is critical for fine-tuning the function of HOIP within LUBAC, thereby preventing overproduction of Met1-linked ubiquitin chains. This regulatory mechanism ensures that in the presence of functional HOIL-1, the overall quantity and potentially the architecture of Met1-linked ubiquitin chains are tightly controlled. We found that elevation of Met1-linked chains, either through HOIL-1 catalytic inactivation or knockdown of the Met1-linked chain-specific deubiquitinase OTULIN, led to defects in aggregate clearance. This suggests that excess Met1-linked ubiquitin may actively contribute to aggregate stabilization and hinder their resolution. Consistent with these cellular observations, our in vitro LLPS assay suggests that excessive Met1-linked chains may promote the formation of rigid, solid-like p62-positive aggregates, a phenomenon directly supported by our cellular biophysical analyses (e.g., 1,6-HD sensitivity and FRAP). These observations were consistent across neuronal cells and MEFs, suggesting a conserved role of HOIL-1 in aggregate regulation. Consistent with previous studies (Furthmann et al, 2023; van Well et al, 2019; Zhang et al, 2022), we found that LUBAC components were sequestered in aggregates, as evidenced by microscopy, confirming the direct involvement of LUBAC in aggregate processing.

Our study implicates selective autophagy, namely aggrephagy, as the primary pathway affected by HOIL-1 loss-of-function. While early autophagy steps remained intact, as evidenced by p62-LC3 colocalization, late-stage autophagy was critically compromised in *Hoil-1*^*C458A/C458A*^ MEFs, highlighting a defect in autolysosome formation and, thus, aggrephagy-mediated degradation of protein aggregates. This impaired clearance is likely exacerbated by the observed transition of p62 bodies from dynamic, liquid-like condensates to more rigid, solid-like structures in the absence of functional HOIL-1. Given the importance of liquidity in early aggregate sequestration and efficient autophagic clearance (Komatsu, 2022; Kurusu et al, 2024; Turco et al, 2021; Yamasaki et al, 2020), our observations suggest that HOIL-1 catalytic activity is essential for maintaining the proper biophysical properties of aggregates, thereby facilitating their efficient turnover.

The precise molecular mechanism by which excessive Met1-linked ubiquitin chains shape the biophysical properties of aggregate materials remains unclear. One possibility is that HOIP directly extends Met1-linked chains on pre-existing ubiquitinated substrates within aggregates, altering their surface properties or internal structures. Another possibility is that free Met1-linked chains are incorporated into growing p62 bodies, thereby directly influencing their physical behavior and promoting solidification. Indeed, p62 and all three components of LUBAC (HOIL-1, HOIP and SHARPIN) have ubiquitin interaction domains, raising the possibility of interaction between ubiquitin chains and these molecules within p62 bodies. However, these mechanisms are not mutually exclusive and require further investigation. We did not observe LUBAC-dependent p62 ubiquitination at least under the conditions we analyzed, further suggesting that interaction between these ubiquitin chains may play a role. Our findings suggest that excess Met1-linked ubiquitin chains lower the threshold for phase transitions, promoting more rigid aggregate states when HOIL-1-mediated catalytic restraint is lost. Although our study provides compelling evidence for the role of HOIL-1 catalytic activity in proteostasis, several important future directions should be considered. The current study primarily relied on in vitro and in cellulo models; therefore, future studies should aim to validate these findings in relevant in vivo neurodegeneration models to confirm the physiological impact of HOIL-1 deficiency on aggregate clearance and disease progression. In a study by Kelsall et al, substitution of the catalytic Cys residue in HOIL-1 with Ser resulted in the accumulation of polyglucosan bodies in the brains, hearts, and livers of aged mice, suggesting a role for HOIL-1 in the clearance of diverse types of protein aggregates (Kelsall et al, 2022). Furthermore, identifying the specific aggregate-associated substrates targeted by LUBAC and characterizing the precise topology of Met1-linked ubiquitin chains, including their length and branching architecture, in HOIL-1 mutant cells will be crucial for fully elucidating the mechanisms by which these chains promote aggregate solidification. Finally, investigation of the potential for therapeutic intervention by modulating HOIL-1 activity or Met1-linked chain levels represents a promising avenue for developing novel treatments for neurodegenerative proteinopathies.

## Methods

**Table:** Reagents and tools table

Reagent/resource	Reference or source	Identifier or catalog number
**Experimental models**		
SH-SY5Y	ECACC	94030304
*Hoil-1* ^*+/+*^ MEFs	Rodriguez Carvajal et al, 2021	N/A
*Hoil-1*^*C458A/C458A*^ MEFs	Rodriguez Carvajal et al, 2021	N/A
HEK293T	ATCC	CRL-3216
Packaging cell line Platinum-E	Ebner et al, 2018	N/A
**Recombinant DNA**		
pcDNA 3.1 human HOIL-1	Gomez-Diaz et al, 2021	N/A
pcDNA 3.1 human HOIL-1 C460A	Gomez-Diaz et al, 2021	N/A
pEGFP-C1 human HOIL-1	Gomez-Diaz et al, 2021	N/A
pEGFP-C1 human HOIL-1 C460A	Gomez-Diaz et al, 2021	N/A
pBABE-puro-mCherry-EGFP-p62	This study	N/A
pGex4T1-mouse ABIN1-UBAN×3	Fennell et al, 2020	N/A
pRK-Myc-human OTULIN	Fennell et al, 2020	N/A
pRK-Myc-human OTULIN C129A	Fennell et al, 2020	N/A
pRK-Myc-human OTULIN C129S	This study	N/A
pcDNA5/FRT/TO/Flag X3-FUS R495X	The Hirose Lab (The University of Osaka) (Yamazaki et al, 2012)	N/A
pMXs-puro GFP-mouse p62	Ebner et al, 2018	N/A
pBABE-puro-mCherry-EGFP-human LC3B	Ebner et al, 2018	N/A
pGEX6P-mCherry-human p62	Ikeda et al, 2023	N/A
pGEX6P-mCherry-human p62 S403E S407E	Ikeda et al, 2023	N/A
pGEX6P1-TR-TUBE	Asaoka et al, 2016	N/A
mScarlet-I3-HOIP	This study	N/A
mScarlet-I3- SHARPIN	This study	N/A
mScarlet-I3- α-synuclein	This study	N/A
**Antibodies**		
Mouse monoclonal anti-HOIL-1 (2E2)	Millipore	MABC576
Rabbit polyclonanl anti-RNF31/HOIP	Aviva Systems Biology	ARP43241_P050
Rabbit polyclonal anti-SHARPIN	Novus	NBP2-04116
Mouse monoclonal anti-Vinculin	Sigma	V9131
Mouse monoclonal anti-LC3	Nanotools	0260-100/LC3-2G6
Rat monoclonal anti-LAMP2	Abcam	ab13524
Rabbit polyclonal anti-p62	MBL	PM045
Rabbit monoclonal anti-α−synuclein (MJFR1)	Abcam	ab138501
Mouse monoclonal anti-phosphorylated α−synuclein (pSyn#64)	Wako	015-25191
Mouse monoclonal anti-ubiquitin (FK2)	Enzo	BML-PW8810-0500
Rabbit polyclonal anti-OTULIN	Abcam	ab151117
Mouse monoclonal anti-c-MYC (9E10)	Covance	MMS-150P
Mouse monoclonal anti-ubiquitin (P4D1)	Santa Cruz Biotechnology	sc-9017
Mouse monoclonal anti-Met1 ubiquitin (LUB9)	Millipore	MABS451
Rabbit monoclonal anti-Met1 ubiquitin (1E3)	Sigma	ZRB2114-25UL
Mouse monoclonal anti-mCherry	Clontech	632543
Anti-Ubiquitin Antibody, Lys63-Specific, clone Apu3, rabbit monoclonal	Sigma	05-1308
HRP-conjugated goat polyclonal anti-mouse IgG	Bio-Rad	170-6516
HRP-conjugated horse polyclonal anti-mouse IgG	Cell Signalling Technologies	7076S
HRP-conjugated goat polyclonal anti-rabbit IgG	Dako	P0448
Alexa Fluor™ 568 goat polyclonal anti-mouse IgG	Invitrogen	A11031
Alexa Fluor™ 488 goat polyclonal anti-rabbit IgG	Invitrogen	A11008
Alexa Fluor™ 568 goat polyclonal anti-rabbit IgG	Invitrogen	A11036
Alexa Fluor™ 488 goat polyclonal anti-mouse IgG	Invitrogen	A11001
Alexa Fluor™ 488 goat polyclonal anti-rat IgG	Invitrogen	A11006
Alexa Fluor™ 405 goat polyclonal anti-mouse IgG	Invitrogen	A31553
**Oligonucleotides and other sequence-based reagents**		
pLKO.1-shRNA-mOtulin	This study	5’-GCTCATACTGTTACCAGAGAACTCGAGTTCTCTGGTAACAGTATGAGCT-3'
pLKO.1-shRNA-control	This study	5’-CCTAAGGTTAAGTCGCCCTCGCTCGAGCGAGGGCGACTTAACCTTAGG-3'
**Chemicals, enzymes and other reagents**		
Ham’s F12	Wako	087-08335
EBSS-MEM	Wako	055-08975
Fetal bovine serum (FBS)	Biowest	S1810-500
Penicillin-streptomycin	Nacalai Tesque	09367-34
Dulbecco’s modified Eagle’s medium high glucose	Wako	044-29765
Polybrene	Sigma-Aldrich	H9268
Puromycin	ROTH	0240.3
cOmplete Mini EDTA-free protease inhibitor cocktail	Roche	11836170001
Western blotting Luminol Reagent	Santa Cruz	sc-2048
α-synuclein Aggregation Assay Kit)	Cosmo Bio	CSR-SYN01
Tau Aggregation Assay Kit	Cosmo Bio	TAU01
Amyloid β-Protein (1-42)	Bachem	4014447
Amyloid Fluorescent Staining Kit	Cosmo Bio	CSR-SYN02
DRAQ7	Abcam	D15106
Vectashield mounting reagent	Vector Laboratories	VECH-1000, without DAPI
ProteoStat ®	Enzo Life Sciences	ENZ-51023
Vectashield mounting reagent	Vector Laboratories	VECH-1200, containing DAPI
BL21 (DE3)	Agilent	200131
cOmplete EDTA-free protease inhibitor cocktail	Roche	11873580001
COSMOGEL ® GST-Accept resin	Nalacai Tesque	09277-72
Glutathione Sepharose™ beads	Cytiva	17513201
ESF 921 Insect Cell Culture Medium Protein Free	Expression Systems	96-001-01
Streptactin Superflow cartridge	IBA Lifesciences	2-1238-001
ATP	Roche	10519979000
ε-Poly-L-lysine coating solution	Cosmo Bio	SPL01
1,6-Hexanediol	Wako	087-00432
GeneJuice ® Transfection Reagent	Millipore	70967
VPS34-IN1	Selleck	S7980
MG132	Enzo Life Sciences	BML-PI102
TAK243 (MLN7243)	Selleck	S8341
InstantBlue ® Coomassie Protein Stain (ISB1L)	Abcam	ab119211
**Software**		
Fiji software	Schindelin et al, 2012	https://imagej.net/software/fiji/downloads
GraphPad Prism 10.4.2	GraphPad software	https://www.graphpad.com/
**Other**		
0.45 μm syringe PVDF filter	Millipore	SLHVR33RS
Nitrocellulose (0.45 μm pore size)	Cytiva	10600002
Nitrocellulose (0.22 μm pore size)	Cytiva	10600011
Polyvinylidene difluoride membrane	Millipore	ISEQ00010
FUSION SOLO S chemiluminescence detection system	Vilber	N/A
LSM900 confocal microscope	Zeiss	N/A
SONIFIER 250	BRANSON	N/A
Chromatography column	Bio-Rad	7321010
VIVASPIN 20 (MWCO 30,000)	Sartorius	VS2022
TOMY Ultrasonic Disruptor	TOMY	UD-100
Centriprep MWCO 50,000	Merck	4311
Micro Ultracentrifuge	HIMAC Eppendorf Group	CS100FNX
Glass-bottom 35 mm dish	Matsunami	D11130H

### Subcloning

pRK-Myc-human OTULIN, pRK-Myc-human OTULIN C129A, and pRK-Myc-human OTULIN C129S, pLKO.1-shRNA-m*Otulin* (shRNA hairpin seq: GCTCATACTGTTACCAGAGAACTCGAGTTCTCTGGTAACAGTATGAGCT) and pLKO.1-shRNA-*control* scramble shRNA (shRNA hairpin seq: CCTAAGGTTAAGTCGCCCTCGCTCGAGCGAGGGCGACTTAACCTTAGG) were generated by standard subcloning methods. mScarlet-I3-tagged expression plasmids were generated by replacing the EGFP coding sequence in the corresponding pEGFP constructs with the mScarlet-I3 coding sequence.

### Cell culture

SH-SY5Y cells (ECACC, 94030304) were maintained at 37 °C in 5% CO_2_ and grown in a 1:1 ratio of Ham’s F12 medium (Wako, 087-08335) and EBSS-MEM (Wako, 055-08975) supplemented with 15% fetal bovine serum (FBS) and 100 U/ml penicillin-streptomycin (Nacalai Tesque, 09367-34). Immortalized MEFs derived from *Hoil-1*^*+/+*^ and *Hoil-1*^*C458A/C458A*^ mice were established as described previously (Rodriguez Carvajal et al, 2021). Human embryonic kidney 293 T (HEK293T) cells (ATCC) and the packaging cell line Platinum-E (Plat-E) (Markowitz et al, 1990; Morita et al, 2000) were maintained at 37 °C in 5% CO_2_ in Dulbecco’s modified Eagle’s medium high glucose (Wako, 044-29765) supplemented with 10% fetal calf serum (Biowest, S1810-500) and 100 U/ml penicillin-streptomycin (Nacalai Tesque, 09367-34). Cells were routinely tested for mycoplasma contamination using the Venor®GeM Classic assay (Minerva Biolabs, 11-1025), and all cultures tested negative.

### Retroviral and lentiviral infection

Retrovirus production was performed as described previously (Ikeda et al, 2007). Briefly, the retroviral plasmids pBABE-puro-mCherry-EGFP-p62, pMXs-puro GFP-p62, and pBABE-puro-mCherry-EGFP-LC3B were transfected into Plat-E cells. At 48 h post-transfection, MEFs were transduced with filtered conditioned media containing retroviral particles using a 0.45 μm syringe PVDF filter unit (Millipore, SLHVR33RS) supplemented with 4 µg/ml polybrene (Sigma-Aldrich, H9268). To obtain stable cell lines, cells were selected using 4 µg/ml puromycin (ROTH, 0240.3). Lentiviral production was performed as described previously (Kumari et al, 2014). Briefly, lentiviral plasmids (pLKO.1-shOtulin or pLKO.1-shControl) were transfected into HEK293T cells along with the psPAX2 packaging plasmid and the pMD2.G envelope plasmid. At 36 h post-transfection, MEFs were transduced with filtered conditioned media containing lentiviral particles supplemented with 4 µg/ml polybrene (Sigma-Aldrich, H9268). To obtain stable cell lines, cells were selected using 4 µg/ml puromycin (ROTH, 0240.3).

### Immunoblotting analysis

This method has been previously described (Ikeda et al, 2011). Cells were lysed in chilled lysis buffer (50 mM HEPES, 150 mM NaCl, 1 mM EDTA, 1 mM EGTA, 1% Triton X-100, 10% glycerol, 25 mM NaF, 10 mM ZnCl2, 10 mM NEM, 1 mM PMSF, 5 mM Na3VO4, pH 7.4 supplemented with cOmplete Mini EDTA-free protease inhibitor cocktail (Roche, 11836170001)), centrifuged at 10,000 RCF to remove cell debris, and total cell lysates were resolved by SDS-PAGE, and transferred onto a nitrocellulose (Cytiva, 10600002 (0.45 µm pore size), 10600011 (0.2 µm pore size)) or polyvinylidene difluoride membrane (Millipore, ISEQ00010). For lysing aggregate formation cells, lysis buffer containing 2% SDS was used as described previously (Sarraf et al, 2020). Membranes were blocked in 5% bovine serum albumin (BSA)-TBS and blotted with indicated antibodies in blocking solution at 4 °C overnight. HRP-conjugated secondary antibodies were used: goat anti-mouse HRP (Bio-Rad, 170-6516), horse anti-mouse, Cell Signalling Technologies, 7076S) and goat anti-rabbit HRP (Dako, P0448). Western blotting Luminol Reagent (Santa Cruz, sc-2048) and FUSION SOLO S chemiluminescence detection system (Vilber) were used for capturing the chemiluminescent signals. Anti-ubiquitin (P4D1) (Santa Cruz Biotechnology, sc-8017) was used for detecting ubiquitin signal.

### Aggregate formation in cells

Aggregates were formed in SH-SY5Y cells grown on coverslips following the manufacturer’s instructions to either create α-synuclein aggregates (α-synuclein Aggregation Assay Kit, Cosmo Bio, CSR-SYN01) or tau aggregates (Tau Aggregation Assay Kit, Cosmo Bio, TAU01). Cells were simultaneously transfected with pEGFP-C1 human HOIL-1 or pEGFP-C1 human HOIL-1 C460A plasmids (Gomez-Diaz et al, 2021) using GeneJuice (Millipore, 70967) transfection reagent according to the manufacturer’s instructions. The cells were grown for 72 h after transfection. Aβ (1-42) (Bachem, 4014447) peptide was solubilized as described previously (Stine et al, 2003). Oligomeric Aβ was prepared by dissolving Aβ in DMSO and phenol-free F12 to a final concentration of 100 μM. The mixture was vortexed and stored at 4 °C for 24 h. The next day, SH-SY5Y cells were transfected with pEGFP-C1 human HOIL-1 or pEGFP-C1 human HOIL-1 C460A and treated with 20 μM Aβ for 24 h. Aggregates were detected using the Amyloid Fluorescent Staining Kit (Cosmo Bio, CSR-SYN02) according to the manufacturer’s instructions, except that the nuclei were counterstained with DRAQ7 (Abcam, D15106). Coverslips were mounted using the Vectashield mounting reagent (Vector Laboratories, VECH-1000, without DAPI).

### Protein aggregate staining using ProteoStat

MEFs were grown on coverslips and treated with 5 μg/ml puromycin for 2 h, 100 nM bafilomycin A1 for 24 h, or subjected to heat stress at 42 °C for 1 h. Cells were fixed in 4% paraformaldehyde (PFA) for 10 min at 37 °C, permeabilized in 0.5% Triton X-100 solution for 5 min, and blocked with 5% FBS-PBS at 37 °C for 1 h. ProteoStat (Enzo Life Sciences, ENZ-51023) was prepared according to the manufacturer’s instructions. Cells were stained using ProteoStat at a 1:2000 dilution in the 1x assay buffer provided in the kit for 20 min. The cells were mounted using Vectashield mounting reagent (Vector Laboratories, VECH-1200, containing DAPI) and imaged using confocal microscopy or Airyscan on an LSM900 confocal system.

### Recombinant protein purification of mCherry-p62 and mCherry-p62 S403E S407E

Purification of mCherry-p62 and mCherry-p62 S403E S407E was performed as described previously (Ikeda et al, 2023). Briefly, pGEX6P-mCherry-p62 and pGEX6P-mCherry-p62 S403E S407E plasmids were transformed into *E. coli* BL21(DE3) cells (Agilent, 200131). Transformed bacteria were grown in 2xYT media containing 100 μg/ml ampicillin. Protein expression was induced with 0.1 mM IPTG and cultured overnight at 18°C. Cells were harvested and lysed by sonication with SONIFIER 250 (BRANSON) in Buffer A (50 mM HEPES pH 8.0, 500 mM NaCl) supplemented with 1 mM TCEP, 1 mM PMSF, and cOmplete EDTA-free protease inhibitor cocktail (Roche, 11873580001). Cell debris was separated by centrifugation, and the supernatant was applied to COSMOGEL® GST-Accept resin (Nacalai Tesque, 09277-72) in a chromatography column (Bio-Rad, 7321010). The resin was washed with Buffer A, followed by on-column cleavage with GST-tagged PreScission protease for ~16 h at 4 °C. The cleaved proteins were collected and concentrated to ~35 μM in Buffer A supplemented with 10% glycerol using VIVASPIN 20, MWCO 30,000 (Sartorius, VS2022). The proteins were flash-frozen in liquid nitrogen and stored at -80°C until use.

### Recombinant protein purification of GST-empty and GST-UBAN×3

Purification of GST-empty and GST-UBAN×3 was performed as described previously (Fennell et al, 2020). Briefly, pGEX4T1 and pGex4T1-ABIN1-UBAN×3 plasmids were transformed into *E. coli* BL21(DE3) cells (Agilent, 200131). Transformed bacteria were grown in LB medium containing 100 μg/ml ampicillin. Protein expression was induced with 0.5 mM IPTG and cultured overnight at 18 °C. Cells were harvested, resuspended in GST Buffer (20 mM Tris-HCl, pH 7.5, 10 mM EDTA, pH 8.0, 5 mM EGTA, 150 mM NaCl, 1 mM PMSF, and 0.1% β-Mercaptoethanol), and lysed by sonication using TOMY Ultrasonic Disruptor (UD-100). 0.5% Triton X-100 was added to the lysate, and the cell debris was removed by centrifugation. Equilibrated Glutathione Sepharose™ beads (Cytiva, 17513201) were added to the supernatant and rotated for 1 h at 4°C. The beads were washed with GST buffer containing 0.5% Triton X-100. Bead-immobilized GST-empty or GST- UBAN×3 was washed and resuspended in 20 mM Tris-HCl, pH 7.5, containing 0.1% NaN_3_. The bead-immobilized GST proteins were stored at 4°C until use.

### Recombinant protein purification of LUBAC from insect cells

LUBAC purification was performed as previously described (Rodriguez Carvajal et al, 2021). Briefly, Sf9 cells were grown in ESF 921 Insect Cell Culture Medium Protein Free (Expression Systems 96-001-01) at 27 °C and infected with baculovirus encoding for human HOIP, His6-human HOIL-1, and Strep (II)-human SHARPIN at a density of 2 × 10^6^ cells/ml. At 72 h post-infection, the cells were harvested, resuspended, and lysed in 100 mM HEPES, 100 mM NaCl, 100 mM KCl, 100 mM Arg, 10 mM MgSO4, and 20 mM imidazole (pH 8.0) supplemented with cOmplete Mini EDTA-free protease inhibitor cocktail (Roche, 11836170001) using the Constant Systems Cell Disruptor at 1.4 kBar. The lysates were then cleared by centrifugation after the addition of 100 mM benzonase and 10 mM PMSF. Cleared lysates were loaded onto a HisTrap FF cartridge (GE Healthcare), proteins were eluted with buffer (100 mM HEPES, 100 mM NaCl, 100 mM KCl, 50 mM arginine, 500 mM imidazole, pH 8.0), and loaded onto a Streptactin Superflow cartridge (IBA Lifesciences 2-1238-001), followed by washes with washing buffer (100 mM HEPES, 100 mM NaCl, 100 mM KCl, pH 8.0), and elution with elution buffer (100 mM HEPES, 100 mM NaCl, 100 mM KCl, 5 mM D-desthiobiotin, pH 8.0). The eluted proteins were concentrated using Centriprep MWCO 50,000 (Merck, 4311). The proteins were flash-frozen in liquid nitrogen and stored at -80°C until use.

### In vitro ubiquitination assay

In vitro ubiquitination assays were performed as described previously (Rodriguez Carvajal et al, 2021). Briefly, reactions were prepared in assay buffer (50 mM HEPES, 150 mM NaCl, 0.5 mM MgSO4, pH 7.5) containing 0.338 μM Ube1 (ubiquitin-activating enzyme 1), 0.338 μM UbcH7 (ubiquitin-conjugating enzyme H7), 0.338 μM LUBAC with HOIL-1 WT or HOIL-1 C460A, 29.0 µM ubiquitin, and with or without 2 mM ATP (Roche, 10519979000). The reaction was carried out at 37 °C for 3 h. For immunoblotting analysis, the reactions were terminated in SDS buffer and boiled at 95 °C. For in vitro LLPS assays, the reaction mixture was flash-frozen and stored at −80 °C until use.

### In vitro LLPS assay

mCherry-p62 or mCherry-p62 S403E S407E (20 μM) was mixed with 20% volume of the in vitro ubiquitination assay reaction mixture in LLPS buffer containing 20 mM HEPES pH 7.5 and 150 mM NaCl (Ikeda et al, 2023). The LLPS reaction was performed on a glass-bottom dish coated with 3% BSA. The reaction was incubated at ~23 °C for 1 h before imaging using a Zeiss LSM900 confocal microscope.

### GST pull-down assay

This method has been previously described (Rahighi et al, 2009). MEFs were grown in a 10 cm dish to 90% confluence and treated with 5 μg/ml puromycin for 2 h. Following the treatment, cells were lysed in chilled lysis buffer (50 mM HEPES, 150 mM NaCl, 1 mM EDTA, 1 mM EGTA, 25 mM NaF, 10 mM ZnCl2, 10% glycerol, 1% Triton X-100, 20 mM NEM, and cOmplete EDTA-free protease inhibitor cocktail (Roche, 11873580001)). Cell debris was removed at 10,000 rpm, at 4 °C for 10 min. 10% of the supernatant was kept aside as input, and the remaining was divided for GST-empty or GST-UBAN×3 pulldown. GST-empty or GST-UBAN×3 coupled beads were incubated with clarified lysates overnight at 4 °C. The beads were then washed three times with chilled lysis buffer. The precipitated proteins were analyzed by immunoblotting.

### Immunofluorescence staining

SH-SY5Y cells transfected with α-synuclein and pEGFP-C1 human HOIL-1 or pEGFP-C1 human HOIL-1 C460A, as described earlier, were fixed in 4% PFA after 72 h transfection. Cells were permeabilized in 0.5% Triton X-100-PBS for 5 min, blocked with 5% FBS-PBS, and stained with primary antibody for 1 h at 37 °C. Samples were washed in PBS and incubated with secondary antibody, Alexa Fluor 568 goat anti-mouse IgG (H + L) (Invitrogen, A11031), or Alexa Fluor 488 goat anti-rabbit IgG (H + L) (Invitrogen, A11008), Alexa Fluor 568 goat anti-rabbit IgG (H + L) (Invitrogen, A11036), Alexa Fluor 488 goat anti-mouse IgG (H + L) (Invitrogen, A11001) Alexa Fluor 488 goat anti-rat IgG (H + L) (Invitrogen, A11006) and Alexa Fluor 405 goat anti-mouse IgG (H + L) (Invitrogen, A31553) for 1 h at 37 °C. Nuclei were counterstained with DRAQ7 (Abcam, D15106), as indicated in the figures. Coverslips were mounted using the Vectashield mounting reagent (Vector Laboratories, VECH-1200, containing DAPI, or VECH-1000, without DAPI) and imaged using confocal microscopy or Airyscan on an LSM900 confocal microscope (Zeiss). MEFs were seeded onto cover slips. For staining endogenous proteins, cells were fixed in cold methanol for 10 min at −20 °C, rehydrated in cold PBS on ice, and washed three times. Coverslips were blocked in 5%BSA-PBS incubated with the indicated primary antibodies (anti-LC3 (Nanotools, 0260-100/LC3-2G6), anti-LAMP2 (Abcam, ab13524) anti-p62 (MBL, PM045), anti-α-synuclein [MJFR1] (Abcam, ab138501), anti-phosphorylated α-synuclein (Wako, 015-25191), anti-ubiquitin (FK2) (Enzo, BML-PW8810-0500), or anti-Met1 ubiquitin (1E3)(Sigma, ZRB2114-25UL)) overnight. Samples were stained with the secondary antibody as described above and visualized using an LSM900 confocal microscope (Zeiss).

### 1,6-Hexanediol disruption of condensates in cells

Cells (0.2 × 10^6^) were seeded onto poly-L-lysine (Cosmo Bio, SPL01)-coated coverslips in six-well plates. The following day, cells were treated with 0, 5, and 10% 1,6-Hexanediol (Wako, 087-00432) for 1 min at room temperature after treatment with 5 μg/ml puromycin, as stated in the figure legend (Yu et al, 2015). After removing 1,6-Hexanediol, the cells were immediately fixed with 4% PFA for 10 min at 37 °C. The cells were washed with PBS, permeabilized in 0.5% Triton X-100-PBS for 5 min, blocked with 5% FBS-PBS at 37 °C for 1 h, and subjected to immunofluorescence as described above.

### Image analysis

Image analysis was performed using FIJI image analysis software (Schindelin et al, 2012). To determine the number, size, and properties (e.g., circularity) of the p62 puncta, a threshold was first applied to the images to highlight only the p62-stained particles. Subsequently, the “Analyze Particle” function was applied. To determine α-synuclein-phospho-α-synuclein, LC3-p62, or LAMP2-p62 double-positive puncta, a threshold was first applied to the 16-bit images stained with α-synuclein or p62 to highlight positively stained particles. Regions of interest (ROI) and signal intensities were measured. The same ROI was applied to the 16-bit images stained with the corresponding antibodies (phospho-α-synuclein, LC3, or LAMP2). The signal intensity in the ROI was measured. Particles with signal intensities greater than the threshold cutoff were defined as double positive. Cells were counted using a threshold value. Watershed was used to separate the overlapping nuclei.

### Sucrose gradient fractionation

MEFs were treated with 5 μg/ml puromycin for 2 h, or after recovery for 2 h, control cells were left untreated. Cells were harvested after the indicated treatments and lysed in lysis buffer containing 50 mM HEPES, 150 mM NaCl, 1 mM EDTA, 1 mM EGTA, 1% Triton X-100, 10% glycerol, 25 mM NaF, 10 mM ZnCl_2_, 10 mM NEM, 1 mM PMSF, 5 mM Na_3_VO_4_, pH 7.4, supplemented with cOmplete Mini EDTA-free protease inhibitor cocktail (Roche, 11836170001). The lysates were fractionated in a sucrose gradient from 5 to 25% sucrose through ultracentrifugation at 55,000 rpm, 4 °C, for 1.5 h in the Micro Ultracentrifuge (HIMAC Eppendorf Group, CS100FNX).

### Autophagy flux assay using mCherry-EGFP-p62 and LC3

For the autophagy flux assay (Kimura et al, 2007; Mizushima & Murphy, 2020), mCherry-EGFP-p62/LC3 stable MEF lines (*Hoil-1*^*+/+*^ and *Hoil-1*^*C458A/C458A*^) were grown in a glass-bottom 35 mm dish (Matsunami) to a confluency of 80-90%. The cells were pretreated with 5 μg/ml puromycin, and 60 frames were imaged with a 2 min interval between frames in the LSM900 confocal system. During imaging, the dishes were kept in a heated hydration chamber with CO_2_ levels maintained at 5%.

### Fluorescence recovery after photobleaching (FRAP)

For Fluorescence recovery after photobleaching (FRAP) analysis, GFP-p62 was stably expressed in *Hoil-1*^*+/+*^ and *Hoil-1*^*C458A/C458A*^ MEFs or transiently expressed in sh*Control* and sh*Otulin* MEFs with GeneJuice Transfection Reagent (Millipore, 70967). The cells were treated with 5 μg/ml puromycin for 2 h before live-cell imaging. Photobleaching was performed with one pulse using a 488 nm laser at 75% intensity within the region of interest (ROI), as indicated in the figure. Live imaging was performed at 5 s intervals for 3 min 20 s. FRAP analysis was performed as described previously (Zheng et al, 2011). Fluorescence intensity was measured within the ROI and normalized against the unbleached control region. Mobile fractions (f_m_) and immobile fractions (f_i_) were calculated using the equations f_m_ = F_∞_/F_0_, where F_∞_ is the fluorescence at full recovery and F_0_ is the fluorescence before photobleaching, and f_i_ = 1- f_m_. The mobile fractions were compared between the HOIL-1 wild-type and mutant samples using an unpaired t-test for statistical analysis. The percentage recovery was then plotted.

### Statistics and data reproducibility

Statistical tests between the two groups were performed using a two-tailed unpaired *t*-test. For non-parametric datasets, a Mann–Whitney test was performed between two sample sets, and a Kruskal–Wallis test was performed for multiple sample sets. Graphical representations and statistical analyses were performed using GraphPad Prism 10.4.2. The number of independent experiments is indicated in the figure legends.

## Supplementary information

Peer Review File

Source data Fig. 1

Source data Fig. 2

Source data Fig. 3

Source data Fig. 4

Source data Fig. 5

Expanded View Figures

## Data Availability

Data corresponding to the figures are made available. Any other raw data or unique reagents related to this study are available upon reasonable request. The source data of this paper are collected in the following database record: biostudies:S-SCDT-10_1038-S44318-026-00909-7.
