# Supplementary material for: Excess Met1-linked ubiquitination leads to solid aggregate formation
Source: EMBO J. 2026 Sep 28;45(20):7246–73. doi: 10.1038/s44318-026-00909-7 (PMC13672799; doi:10.1038/s44318-026-00909-7)
Supplement: Supplementary file 1 [file 44318_2026_909_MOESM1_ESM.pdf]

THE EMBO JOURNAL

Excess Met1-linked ubiquitination leads to solid aggregate formation

Stephanie Kaypee, Minori Miyasaka, Takuto Nakajima, Hiromi Nishimura, Jun-ichi Sakamaki, Masaaki Komatsu, and Fumiyo Ikeda

Corresponding author(s): Fumiyo Ikeda (ikeda.fumiyo.fbs@osaka-u.ac.jp)

Review Timeline:

Transfer from Review Commons:	13th Apr 26
Editorial Decision:	23rd Apr 26
Revision Received:	24th Jul 26
Editorial Decision:	27th Aug 26
Revision Received:	31st Aug 26
Accepted:	1st Sep 26

Review Commons

Editor: Hartmut Vodermaier

Transaction Report: This manuscript was transferred to The EMBO JOURNAL following peer review at Review Commons.

Review #1

1. Evidence, reproducibility and clarity:

Evidence, reproducibility and clarity (Required)

Background

GWAS analyses carried out several years ago identified over 40 genetic loci associated with increased risk of developing Alzheimers and other types of dementia. They included loci encoding the E3 ubiquitin ligase HOIL-1 and the protein SHARPIN, two of the three components of the Linear Ubiquitin Assembly Complex (LUBAC) (Bellenguez et al, 2022, cited in the paper). The third component of LUBAC, HOIP, is the only E3 ubiquitin ligase known to catalyse the formation of Met1-linked ubiquitin (also known as linear ubiquitin). HOIL-1 is one of the few E3 ubiquitin ligases that attaches ubiquitin to serine and threonine residues in proteins forming ester bonds (Kelsall et al. 2019) and has been reported to restrict the HOIP-catalysed formation of Met1-linked ubiquitin (Kelsall et al. 2019; Fuseya et al. 2020; Rodriguez Carvajal et al. 2021).

Summary of Paper's findings:

In this study the authors report that HOIL-1 catalytic activity prevents neurodegenerative protein aggregation (synuclein, tau, Aβ) in the human SH-SY5Y neuroblastoma cell line or in mouse embryonic fibroblasts (MEFs) expressing a catalytically inactive mutant of HOIL-1. They argue that this is achieved by maintaining the dynamic, liquid-like properties of protein condensates through regulation of Met1-linked ubiquitin chain levels, thereby facilitating efficient clearance via the aggrephagy pathway. They report that loss of HOIL-1 activity leads to excess Met1-ubiquitylation that drives the transition to rigid, solid-like aggregates resistant to autophagic degradation. In support of this conclusion, they also report that the siRNA knock-down of Otulin, a deubiquitylase that hydrolyses Met1-linked ubiquitin specifically, produces the same effect .
The reframing of HOIL-1 as a key factor for fine-tuning ubiquitylation to maintain cellular protein homeostasis is an interesting development and the paper is generally well-written, focused and concise. Further work is required however, to fully convince these reviewers that the effects observed are entirely attributable to excess Met1-linked ubiquitylation, as claimed.

Major comments:

1. The causal link between elevated Met1-linked chains and solid-like aggregates in cells is the central claim of the paper. Throughout the study the authors use inactive HOIL-1 to enhance aggregate formation, which they attribute to increased Met1-linked ubiquitylation, something observed by themselves and others previously (Kelsall et al. 2019; Fuseya et al. 2020; Rodriguez Carvajal et al. 2021). However, the immunoblot for Met1-linked ubiquitin (Fig 5A) is not very convincing. In addition, the authors have not excluded the possibility that the loss of HOIL-1 enzyme activity has other effects on ubiquitylation, such as a change in the architecture of the ubiquitin chains caused by the absence of HOIL-1 catalysed formation of oxyester linkages. Many/most ubiquitin chains formed in cells contain more than one ubiquitin linkage type. It is therefore important for the authors to perform immunoblots for other ubiquitin linkage types, such as Lys63-linked ubiquitin, and to include these results in Fig 5.
2. The reviewers also think that the authors' claims that the transition of condensate property is linked to elevated Met1-linked ubiquitin chains would be strengthened by performing the biophysical assays (FRAP and 1,6-hexanediol resistance) after Otulin knockdown/knockout (and ideally also with Otulin rescue). This will provide direct biophysical evidence linking Met-1 linked chain elevation to condensate liquidity and 1,6-HD sensitivity.
3. The authors have not shown any evidence that Met1-linked chains are more enriched at the sites of protein aggregation. Would the authors be able to demonstrate direct spatial colocalization of Met1-Ub with the analysed aggregates?
4. Do the authors know if the effects that they are seeing are general effects on autophagy? For example, is starvation-induced autophagy similarly impaired in the cells studied? A simple flux-style experiment looking at LC3-II levels and p62 with starvations vs puromycin (-/+ bafilomycin) would be informative here.

Minor comments:

5. The authors show that loss of HOIL-1 catalytic activity causes p62 bodies to transition from dynamic liquid-like states to rigid solid-like states and claim this as a more general effect on protein aggregates. But the study does not directly demonstrate a liquid-to-solid transition for the disease-relevant α-synuclein, tau, or Aβ aggregates, limiting the generalisation of the claim beyond p62 bodies. Perhaps the authors should modify the text to better reflect this (or, even better, consider treating α-synuclein/tau/Aβ aggregates with 1,6-hexanediol to measure the response). [optional]
6. Given that the blots presented in Fig S1A appear to come from different membranes, and high-molecular-weight species of α-synuclein seem to exist in the insoluble pellet fraction of both WT and C460A expressing cells, the reviewers would caution against concluding

anything about differences, which can only be assessed if the samples are run side-by-side on the same gel.
7. The Methods section says that two different total ubiquitin antibodies were purchased, but which one was used in Figure 5 and other figures are not stated. Please clarify.
8. On page 10 ABIN1 is mentioned but it is not mentioned that it is the protein product of the TNIP1 gene that is mentioned in the Introduction. This will confuse to many readers.
9. 1st paragraph of Discussion line 5 from bottom:- change "oof" to "of".

2. Significance:

Significance (Required)

Prior research has established that the components of LUBAC are recruited to, and are components of, protein aggregates. A link between LUBAC and selective autophagy has also been established previously. The significance of this paper is that it identifies the catalytic function of HOIL-1 as a brake on the activity of LUBAC in proteostasis. The reviewer and co-reviewer are not experts in autophagy or aggregate formation in dementia but, if those reviewers who are find the data presented in these areas to be convincing, then this paper may be the first to suggest a molecular mechanism by which polymorphism/mutation of HOIL-1 leads to increased formation of the aggregates observed in Alzheimer's and other dementias. The results presented in the paper also suggested that initial autophagosome recruitment to aggregates is intact but subsequent late-stage autophagy is impaired. Hence, the study begins to identify the specific step that fails. However, as the authors themselves acknowledge, validation of these potentially exciting findings using in vivo models of neurodegeneration should be the aim of future studies.
The paper combines the molecular dissection of ubiquitin and autophagy pathways to understand the causes of neurodegenerative disease. The paper will therefore be of interest to a broad audience, encompassing both the basic research and clinical research communities.

Reviewers field of expertise: Biochemists and cell biologists with an interest in ubiquitin and cell signalling.

3. How much time do you estimate the authors will need to complete the suggested revisions:

Estimated time to Complete Revisions (Required)

(Decision Recommendation)

Between 1 and 3 months

4. *Review Commons* values the work of reviewers and encourages them to get credit for their work. Select 'Yes' below to register your reviewing activity at [Web of Science Reviewer Recognition Service]() (formerly Publons); note that the content of your review will not be visible on Web of Science.

Web of Science Reviewer Recognition

Yes

Review #2

1. Evidence, reproducibility and clarity:

Evidence, reproducibility and clarity (Required)

Summary:

In this manuscript, the authors show that the branching activity of the E3 ligase HOIL-1, a component of the LUBAC complex, contributes to the autophagic clearance of p62 bodies and protein aggregates. This activity is attributed to enhanced linear, unbranched ubiquitin chain formation by the second E3 ligase of LUBAC, called HOIP. The model systems employed are cell lines including MEFs expressing a catalytically dead version of HOIL-1. In addition, the authors perform in vitro reconstitution experiments with purified ubiquitin chains, the LUBAC complex and p62. The main message is that solid p62 bodies are poor substrates for autophagy and that linear, non-branched ubiquitin chains promote solidification. The mechanism remains unclear and some of the effects sizes are rather modest.

Major comments:

The key observations mentioned above are convincingly shown. Since the authors don't claim any detailed molecular mechanisms, the number of conclusions in this study are limited.

Overall, the authors are quite careful regarding their conclusion, and therefore the ones that are made in this manuscript are generally well supported. The data regarding the clearance of the p62 bodies presented in Figure 3 should be backed up with additional data. The authors could add a macroautophagy inhibitors such as VPS34 IN1 and/or

perform the clearance experiments in a ATG KO/KD cell line to corroborate the contribution of macroautophagy to the clearance. In addition, a proteasome inhibitor should be used for comparison.

The expertise and resources for the experiments mentioned above are expected to be well within the authors' capacity and should be doable within a few weeks.

Some of the effects sizes (e.g. Fig. 5 and S5) are very small and it is possible that some of them are below statistical significance if the number of replicates are increased.

Minor comments:

Figure 1D should be quantified, for example using PCC, Pearson correlation coefficient.
Figure S1 should be quantified.
Figure S3: It should be explained how the region for the profiles are shown were selected.

It is suggested to include a scheme of the LUBAC complex and its E3 ligase activities in Figure 1A. This will make it more accessible for readers, who are not so familiar with this complex, in particular as HOIP and HOIL can be easily confused. The authors may also want to clarify this in the abstract.

2. Significance:

Significance (Required)

As mentioned in the summary. The authors report the observation that an excess of linear ubiquitin chains produced by HOIP in the absence of HOIL-1 activity results in the solidification of p62 bodies and reduced clearance by autophagy. This observation is novel and will be interesting for the proteostasis field.

This reviewer is expert in autophagy and protein degradation, but less so in the LUBAC complex.

3. How much time do you estimate the authors will need to complete the suggested revisions:

Estimated time to Complete Revisions (Required)

(Decision Recommendation)

Between 1 and 3 months

4. *Review Commons* values the work of reviewers and encourages them to get credit for their work. Select 'Yes' below to register your reviewing activity at [Web of Science Reviewer Recognition Service]() (formerly Publons); note that the content of your review will not be visible on Web of Science.

Web of Science Reviewer Recognition

Yes

Review #3

1. Evidence, reproducibility and clarity:

Evidence, reproducibility and clarity (Required)

Summary:

The study 'Excess Met1-ubiquitination leads to solid aggregate formation' by Kaypee et. al suggests a previously unrecognised role for the E3 ligase HOIL-1 in clearing protein aggregates via autophagy (e.g. aggrephagy). In their model, toxic protein aggregates in cells are modified with ubiquitin chains, including M1-linked Ub-chains catalysed by LUBAC (of which HOIL-1 is a component). The HOIL-1 ubiquitin signal is posited to induce trafficking of aggregates to lysosomes for subsequent clearance. However, when HOIL-1 is inactive (catalytic C460A mutation), the pathway is interrupted. As a result, protein aggregates fail to clear, they increase in size and shift their biophysical properties from liquid-like to more rigid, insoluble aggregates. The authors explain their observations by an increasing amount of M1-linked chains on protein aggregates, which occur as a result of 'unrestrained HOIP activity' due to HOIL-1 inactivity (based on previous work). Increasing amounts of M1-chains are posited to promote aggregate formation, aggregate growth, and prevent clearance.

The major claims made in this manuscript are the following:

1. Following the induction of protein aggregate formation (e.g. alpha-synuclein, tau, beta-amyloid, p62 bodies), cells that express catalytically inactive HOIL-1 fail to clear protein aggregates and end up with more, larger and more rigid protein aggregates compared to cells that express WT HOIL-1.
2. The observations made in 1. are due to disruptions in late-stage autophagic flux. While

aggregates in cells that express WT HOIL-1 co-localise with autophagy and lysosomal markers, aggregates in cells that express mutant HOIL-1 show autophagic, but not lysosomal markers.
3. An increase in M1-chains (either due to HOIL-1 inactivity or due to OTULIN knockout) is believed to be the cause for claims 1. and 2.

Main methodologies used:

The authors use two cellular systems. The first one is SH-SY5Y cells in which either WT or mutant HOIL-1 are transiently overexpressed (via the pcDNA3.1 plasmid), and physiologically important aggregates (Tau, Abeta, asyn) are induced. The second cellular system is MEF cells in which either WT or mutant HOIL-1 are endogenously expressed; in these cells aggregates are formed crudely through disruption of ribosomal translation. It is questionable if both systems can be compared. Aggregate formation is mainly monitored and quantified via fluorescent microscopy in both fixed and live cells, or via sucrose gradient fractionation to separate soluble and insoluble (=aggregate) fractions. The rigidity of protein aggregates is analysed in cells via FRAP, size and circularity measurements and 1,6-HD treatment, or in vitro after aggregate formation assays via size and circularity measurements.
The observations are on the whole interesting, though the authors fail to discuss their data in light of previously published work. For example, HOIL-1 KO and KI animals were shown to feature polyglucososan bodies in brain, which is not mentioned. Also, McCrory et al on HOIL-1 chain types is not cited but seems relevant (Figure S4). Yet, the manuscript reports a number of interesting findings, more or less coherently, most useful for scientists embedded in current ubiquitin, autophagy, and LLPS fields. These reviewers believe that this manuscript will make a lot of sense in due course, and be well cited for a first description of the role of HOIL-1 in cellular quality control processes.
A number of improvements seem required to consolidate the findings, and improve readability and impact.

Major:

1. Figure 1A-C: The authors transiently overexpress either WT HOIL-1L or catalytically inactive (C460A) HOIL-1 in SH-SY5Y cells, then induce and compare the formation of protein aggregates (alpha-synuclein, tau, amyloid-beta) in those cells over 72 h. More cells with aggregates were found in cells that overexpressed mutant HOIL-1L. While these findings are interesting, the cellular system used is artificial due to the transient overexpression of HOIL-1 (in presence of endogenous HOIL-1). Crucial controls are

missing:

a. Adding a condition in which no protein is overexpressed, for example via an empty pcDNA3.1 or GFP only vector. This would help ruling out secondary effects due to the transient overexpression. It would also allow to monitor whether the same amount of aggregates form in the empty ctr compared to when WT HOIL-1 is overexpressed.

b. Figure legends and raw data points (?) in graphs do not match. The graphs show dubious statistics from 2-3 grey dots, while the figure legend refers to n=100 cells etc. This needs to be fixed.

c. Showing Western Blots of HOIL-1, to better understand the levels of endogenous HOIL-1 vs overexpressed HOIL-1 in these cells, and to compare overexpression levels between WT and mutant HOIL-1.

d. The study would also improve by western blotting and IF staining for other LUBAC components such as HOIP and SHARPIN. Do alpha-synuclein aggregates in both WT and mutant conditions co-localise with the other LUBAC components, and are there any differences between WT and mutants. This would further help strengthening the claims made in Figure S1A: '…suggesting that LUBAC is recruited to or retained within α-Synuclein aggregates.' And in the discussion: 'we found that LUBAC components were sequestered in aggregates, as evidenced by microscopy and gradient fractionation of soluble and insoluble proteins, confirming the direct involvement of LUBAC in aggregate processing.'

2. Figure 2A-F: The authors change to a genetic-derived system (comparing endogenously expressed HOIL-1 WT with mutant HOIL-1 based on MEF cells from their mouse models). However, they use puromycin to produce aggregates from random protein homeostasis defects, which yes leads to aggregates, but is not as nice as the induced generation of neiurodegeneration-relevant aggregates. It was observed that after 2 h of puromycin treatment, cells accumulate p62-positive protein aggregates, and in during recovery (2 h washout), the aggregates in the HOIL1 mutant cells outgrow the aggregates in the WT HOIL1.

a. However, the authors claim that: 'While Hoil-1+/+ MEFs efficiently cleared puromycin-induced p62 bodies,…', which is not supported by the data shown here. When comparing WT in panel C with WT in panel E, it becomes evident that the average number of p62 puncta before and after recovery is the same (around 5 puncta/cell in both pre and post washout conditions). A similar observation can be made for the mutant (around 12 puncta/cell in both pre and post washout conditions). Can the authors please amend their

claims, or comment and perform a direct statistical comparison between the pre and post recovery conditions to test for clearance of p62 puncta in the WT after puromycin washout.

b. The authors state that: 'These findings indicate that although HOIL-1 catalytic activity is dispensable for the initial formation of puromycin-induced aggregates, it is essential for their subsequent clearance.'

As long as clearance of p62 bodies in the WT is not clearly shown, the second part of this sentence should be amended/removed.

c. The experiments shown would improve by adding a t = 0 condition. How many p62 granules are present before puromycin treatment? Is there already a basal difference between WT and mutant HOIL-1L cells?

3. Figure S1A: The authors claim that other LUBAC components co-localise to protein aggregates, based on sucrose gradient fractionation and the presence of the respective proteins in the insoluble fractions. Could the authors perform IF and stain for other LUBAC components (SHARPIN and/or HOIP) in their MEF cell system to directly validate this claim?

4. Figure 3G-H: The authors created a GFP-mCherry-p62 reporter system in both their WT and mutant HOIL-1 MEF cells and performed live cell imaging following puromycin treatment, which allows monitoring of both aggregate formation and loss of GFP signal due to the acidic lysosomal localisation. Excitingly, the ratio of GFP/mCherry in the later timepoints is reduced in the WT compared to mutant HOIL-1, indicating that HOIL-1 activity is required to traffic p62 bodies to lysosomes.

a. In panel G, a surprisingly large amount of p62 granules are present at t0, which (according to the relevant method section) is the time of puromycin treatment. This observation can be made for both WT and mutant cells. After 80 min of puromycin treatment in the WT, the majority of these puncta are cleared. Can the authors please comment on this high amount of p62 granules at t0 (before the effects of puromycin? And also on the observation that after 80 min there are now less granules than before puromycin? In case that t0 indicates the time of puromycin washout rather than puromycin addition, could this please be clarified in the methods or figure legend?

b. Panel H would improve by adding the quantifications for t=0 (or ideally for all the time points).

c. Fig S3C-D: Same comments as before but for GFP-mCherry-LC3.

5. Fig. S4B and Fig 4A-B: The authors state that circular aggregates are more soluble and have more LLP characteristics, whereas non-circular aggregates are less soluble and have more aggregate-like characteristics. However, the aggregates shown in Fig. S4B are un-circular but easily dissolve in response to 1,6-HD treatment, which seems contradictory. On the other hand, the aggregates shown in Fig 4B in HOIL-1 mutant cells appear much rounder than the ones in S4B, but do not dissolve in response to 1,6-HD treatment. Can the authors please comment on these discrepancies?
6. Fig. 4E-G. Here the authors suddenly switch to an in vitro aggregate-formation assay using mCherry-p62. In-vitro M1-chain reactions with either WT LUBAC or LUBAC with mutant HOIL-1L, together with the respective M1-chain reaction product, are added. This is not clear from the figure, and a schematic, as well as a gel (Coomassie) should be included to show component purity and indicate the biochemical in vitro nature of the experiment. It is good to have this breadth of methods, but does not help in the presentation if all figures look alike.

a. The key difference to the cellular situation is p62 aggregates are not directly ubiquitinated here, and instead ubiquitin chains are (non-covalently) added to samples. Can the authors please make this important difference clearer in their text? Why not directly ubiquitinate mCherry-p62 via LUBAC (WT vs mutant HOIL-1) and then perform an aggregation assay on the reaction product?

b. Can the authors please clarify whether the reaction was inactivated prior to addition to the aggregate-formation assay? If not, the enzymes might still be active at the point of aggregate formation, and the observed effects might be influenced by enzymatic activities and not only the presence of different M1-chain architectures.
7. Fig. 5B-C: The M1-specific DUB OTULIN is knocked down (again, cells) to increase the overall amount of M1-linked Ub-chains present in cells. P62 aggregate formation is induced and the authors claim that the increase in M1-chains influences aggregate size. This claim would be strengthened if it was directly shown that M1-chains form on p62 aggregates in this assay, for example via IF using an M1-antibody (and potentially a total ubiquitin antibody). This would also enable to directly compare the abundance of M1-chains between conditions (ctr vs Otulin, WT vs HOIL-1L mutant).

Minor:

1. Figure 1 D: The authors state that 'Notably, HOIL-1 C460A was detected within these structures, as demonstrated by its colocalization with tau aggregates' and show a co-localisation comparison between WT and mutant HOIL-1L. This sentence implies that WT

HOIL-1 was not detected in aggregates, however the chosen image of the WT cells does not show any obvious tau aggregate, even though aggregates were induced in this condition according to 1A-C. The better comparison would be to pick an image that includes a tau aggregate. Moreover, this experiment would benefit from quantification, calculating the percentage of total aggregates that co-localise with WT HOIL-1L vs with mutant HOIL-1L.

2. Figure S1A: The authors state that 'Cells expressing HOIL-1 C460A displayed a pronounced accumulation of high-molecular-weight α-Synuclein species in the insoluble pellet fraction.' While the difference seen by Western Blot is apparent and seems to match Fig 1 A-C, it is not very strong. Moreover, the relevant comparison (WT vs mutant) is made between two different blots/membranes, and it is difficult to assess equal input solely based on the TCL lane. This experiment could be improved by normalising samples (for example via BCA) and by loading and imaging the two conditions (or at least the TCL and pellet samples) on the same membrane.

The authors also state that: 'LUBAC subunits, HOIP, HOIL-1, and SHARPIN, were also enriched in this fraction, suggesting that LUBAC is recruited to or retained within α-Synuclein aggregates.' Both Sharpin and HOIL-1 seem to be present in similar levels in the pellet fractions of WT and mutant HOIL-1. Overall HOIP levels seem to be significantly increased in the mutant over the WT (see TCL lane), and to a similar level in the pellet fraction. It would be great if the authors could include these observations in their interpretation.

3. Figure 2G: Similar to before, this experiment would improve if the authors could find a way to normalise samples between conditions prior to sucrose gradient fractionation or have the most relevant samples on the same blot. It is challenging to properly interpret the results while the bands in the total cell lysate (TCL) lane do not have similar intensities between samples. A blot in which only the TCL and the pellet samples of all conditions were loaded onto the same gel would solve this, allowing for a better comparison between conditions.

Based on what is shown in panel G, the authors should amend their claim: 'Consistent with microscopic observations, denser fractions from Hoil-1C458A/C458A MEFs contained increased signals of p62 specifically during the recovery phase (Figure 2G).' It is not apparent that more p62 is present in the insoluble fractions of mutant HOIL-1 cells after puromycin treatment. The band intensities look very similar (This is different for the recovery condition, which shows a strong difference, as stated).

4. Figure S2: Similar to before, the authors induce protein aggregate formation and compare cells endogenously expressing WT vs mutant HOIL-1L. The size of aggregates increases in mutant cells under proteotoxic stress. What happens to the number of aggregates per cell in these conditions? Does it also increase, or is it just the size?

5. Fig. S4A: Here the authors analyse the circularity of p62 aggregates in HOIL-1L mutant

cells after recovery from puromycin treatment. This experiment would improve if the same analysis could be performed for the WT cells and for the pre-recovery condition (under the condition that large enough granules are present), allowing to make a comparison between WT and mutant, as well as between pre- and post-recovery.

6. Fig. 4H-J: The authors use a p62 mutant that is known for its enhanced ubiquitin affinity, repeat Fig. 4E-G and state: 'These aberrant condensates were similar to those observed in a reaction using wild-type p62.' Can the authors please comment on what they conclude from this similarity and why this experiment was performed? A quantitative comparison (condensate size and circularity) between WT p62 and mutant p62 may further be useful here.

7. Fig. 5A: Here the authors pulldown M1-linked Ub-chains in WT vs mutant HOIL-1 cells, with or without puromycin-induced aggregate formation. More M1-chains are observed in mutant HOIL-1 cells under puromycin treatment, but the difference is very subtle. The conclusions drawn from this experiment could be strengthened by including alternative methods, for example (if available) Ub-AQUA to measure the abundance of M1-chains, or using the M1-antibody for IF analysis.

8. Fig. S5B: The described differences between puromycin treatment and untreated conditions are extremely subtle on the anti-Ub blot, and absent in the anti-Met1 blot. I recommend that the authors remove this sentence, based on the shown data: 'we observed a modest increase in the signal of Met1-linked ubiquitin chains after puromycin treatment'.

9. Fig. 5F-G: The authors went back to their more-artificial system from Fig 1, in which HOIL-1L was transiently overexpressed (WT or inactive mutant) in SH-SY5Y cells, alongside alpha-synuclein aggregate formation.

a. The claims made from this experiment would be stronger in the other cell system. Could OTULIN be transiently overexpressed in the MEF cells, to monitor the effect of aggregate formation and clearance. Again, staining aggregates with the M1-antibody would improve this experiment.

b. The authors claim that HOIL-1 activity fine-tunes the function of HOIP within LUBAC (from discussion: 'This regulatory mechanism ensures that in the presence of functional HOIL-1, the overall quantity and potentially the architecture of Met1-linked ubiquitin chains are tightly controlled'). What is the quantity and architecture of M1-chains catalysed by LUBAC when HOIL-1 is very highly abundant, as it would be the case in this cellular overexpression system?

10. Sentence structure: 'A comprehensive understanding of how Met1-linked ubiquitination, particularly through intricate regulation by Linear Ubiquitin Chain Assembly

Complex (LUBAC) components, such as HOIL-1, influences aggregate dynamics and clearance; therefore, it is crucial to develop targeted therapeutic strategies against neurodegenerative proteinopathies'.

2. Significance:

Significance (Required)

The conclusions drawn from this study are very intriguing and give LUBAC (and HOIL-1) a so far unrecognised role in the clearance of protein aggregates, which are a hallmark of several neurodegenerative diseases for which there are currently no cures. Some of the findings described in this manuscript have the potential to be of very high impact and interest to a broader community, in particular researchers interested in protein homeostasis, autophagy, ubiquitin biology and neurodegeneration. In fact, those findings might even expand to autophagic pathways that target other cargo than protein aggregates. Both the novelty aspect and the potential for translational/therapeutic applications comprise the major strength of this manuscript. However, multiple of the presented experiments are currently lacking crucial controls, show weak effect sizes or were performed in artificial settings that likely do not represent relevant in vivo conditions, overall weakening or not fully supporting the claims made. Consequently, further experiments, data re-analyses and validations were recommended to fully support all the claims made here.

This review was written from the perspective of a researcher in the ubiquitin field.

3. How much time do you estimate the authors will need to complete the suggested revisions:

Estimated time to Complete Revisions (Required)

(Decision Recommendation)

Between 3 and 6 months

4. *Review Commons* values the work of reviewers and encourages them to get credit for their work. Select 'Yes' below to register your reviewing activity at [Web of Science Reviewer Recognition Service]() (formerly Publons); note that the content of your review will not be visible on Web of Science.

Web of Science Reviewer Recognition

Yes

Revision Plan

Manuscript number: RC-2026-03404
Corresponding author(s): Fumiyo, Ikeda

1. General Statements [optional]

This section is optional. Insert here any general statements you wish to make about the goal of the study or about the reviews.

We are grateful for the thorough and constructive referee reports, which collectively recognize both the novelty and the potential impact of our study. The reviewers consistently identify the central finding—that HOIL-1 catalytic activity fine-tunes LUBAC-dependent Met1-linked ubiquitination to regulate condensate material properties and late-stage aggrephagy—as conceptually interesting and potentially foundational for the fields of ubiquitin biology, autophagy, LLPS, and neurodegeneration.

At the same time, the reviewers appropriately highlight areas where additional controls, clarifications, and contextualization are required to consolidate the conclusions and improve clarity and impact. We view these comments as strengthening rather than refuting the study. Importantly, the requested revisions are focused on rigor, quantification, and mechanistic support, rather than on questioning the core observations or overall framework.

As detailed in the revision plan, many of the suggested experiments are already underway or build directly on established systems and reagents used in the current study. Preliminary data support the feasibility of these approaches, and we are confident in our ability to carry out the remaining experiments and analyses within a focused revision. We will also integrate relevant prior literature more explicitly and refine the text to ensure that conclusions are precise, balanced, and well supported.

Overall, we believe this work represents an exciting first description of a regulatory role for HOIL-1 in cellular quality control processes and provides a solid framework for future *in vivo* and translational studies. We are fully committed to completing the required revisions and delivering a strengthened manuscript that fully realizes the potential highlighted by the reviewers.

2. Description of the planned revisions

Insert here a point-by-point reply that explains what revisions, additional experimentations and analyses are <u>planned</u> to address the points raised by the referees.

We have begun the experiments required for the revision; however, several need to be repeated to ensure reproducibility. Accordingly, we outline here our planned approaches and present preliminary observations that support the feasibility of completing these revisions.

Reviewer #1
Evidence, reproducibility and clarity

Background
GWAS analyses carried out several years ago identified over 40 genetic loci associated with increased risk of developing Alzheimers and other types of dementia. They included loci encoding the E3 ubiquitin ligase HOIL-1 and the protein SHARPIN, two of the three components of the Linear Ubiquitin Assembly Complex (LUBAC) (Bellenguez et al, 2022, cited in the paper). The third component of LUBAC, HOIP, is the only E3 ubiquitin ligase known to catalyse the formation of Met1-linked ubiquitin (also known as linear ubiquitin). HOIL-1 is one of the few E3 ubiquitin ligases that attaches ubiquitin to serine and threonine residues in proteins forming ester bonds (Kelsall et al. 2019) and has been reported to restrict the HOIP-catalysed formation of Met1-linked ubiquitin (Kelsall et al. 2019; Fuseya et al. 2020; Rodriguez Carvajal et al. 2021).

Summary of Paper's findings:
In this study the authors report that HOIL-1 catalytic activity prevents neurodegenerative protein aggregation (synuclein, tau, Aβ) in the human SH-SY5Y neuroblastoma cell line or in mouse embryonic fibroblasts (MEFs) expressing a catalytically inactive mutant of HOIL-1. They argue that this is achieved by maintaining the dynamic, liquid-like properties of protein condensates through regulation of Met1-linked ubiquitin chain levels, thereby facilitating efficient clearance via the aggrephagy pathway. They report that loss of HOIL-1 activity leads to excess Met1-ubiquitylation that drives the transition to rigid, solid-like aggregates resistant to autophagic degradation. In support of this conclusion, they also report that the siRNA knock-down of Otulin, a deubiquitylase that hydrolyses Met1-linked ubiquitin specifically, produces the same effect . The reframing of HOIL-1 as a key factor for fine-tuning ubiquitylation to maintain cellular protein homeostasis is an interesting development and the paper is generally well-written, focused and concise. Further work is required however, to fully convince these reviewers that the effects observed are entirely attributable to excess Met1-linked ubiquitylation, as claimed.

We thank the reviewer for the careful assessment and positive evaluation of our study. We appreciate the recognition of the conceptual advance in reframing HOIL-1 as a regulator of protein homeostasis, as well as the clarity, focus, and concision of our presentation. Below are the detailed plans for the revision.

Major comments:
1. The causal link between elevated Met1-linked chains and solid-like aggregates in cells is the central claim of the paper. Throughout the study the authors use inactive HOIL-1 to enhance aggregate formation, which they attribute to increased Met1-linked ubiquitylation, something observed by themselves and others previously (Kelsall et al. 2019; Fuseya et al. 2020; Rodriguez Carvajal et al. 2021). However, the immunoblot for Met1-linked ubiquitin (Fig 5A) is not very convincing.

We thank the reviewer for raising this important point. We will repeat the experiments using larger amounts of starting material to improve the detection of Met1-linked ubiquitin by immunoblotting. Because Met1-linked chains are enriched at specific cellular sites such as condensates and aggregates, total lysates may not optimally reflect their abundance. Therefore, we will additionally assess Met1-linked ubiquitin using immunocytochemistry with a chain-specific antibody.

In addition, the authors have not excluded the possibility that the loss of HOIL-1 enzyme activity has other effects on ubiquitylation, such as a change in the architecture of the ubiquitin chains caused by the absence of HOIL-1 catalysed formation of oxyester linkages.

Indeed, HOIL-1 has been shown to generate hybrid Met1/oxyester ubiquitin chains, as demonstrated in our previous work (Rodriguez Carvajal et al., 2021) and by others (Kelsall et al., 2019; Fuseya et al., 2020). It is therefore of particular interest to examine the functional impact of this specific ubiquitin code. To address this, we will take advantage of the deubiquitinase Cezanne, which we have previously shown to hydrolyze oxyester linkages within Met1-linked chains (Rodriguez Carvajal et al., 2021). We outline the planned experimental set-up below; construction and expression validation of mScarlet-Cezanne have already been completed.

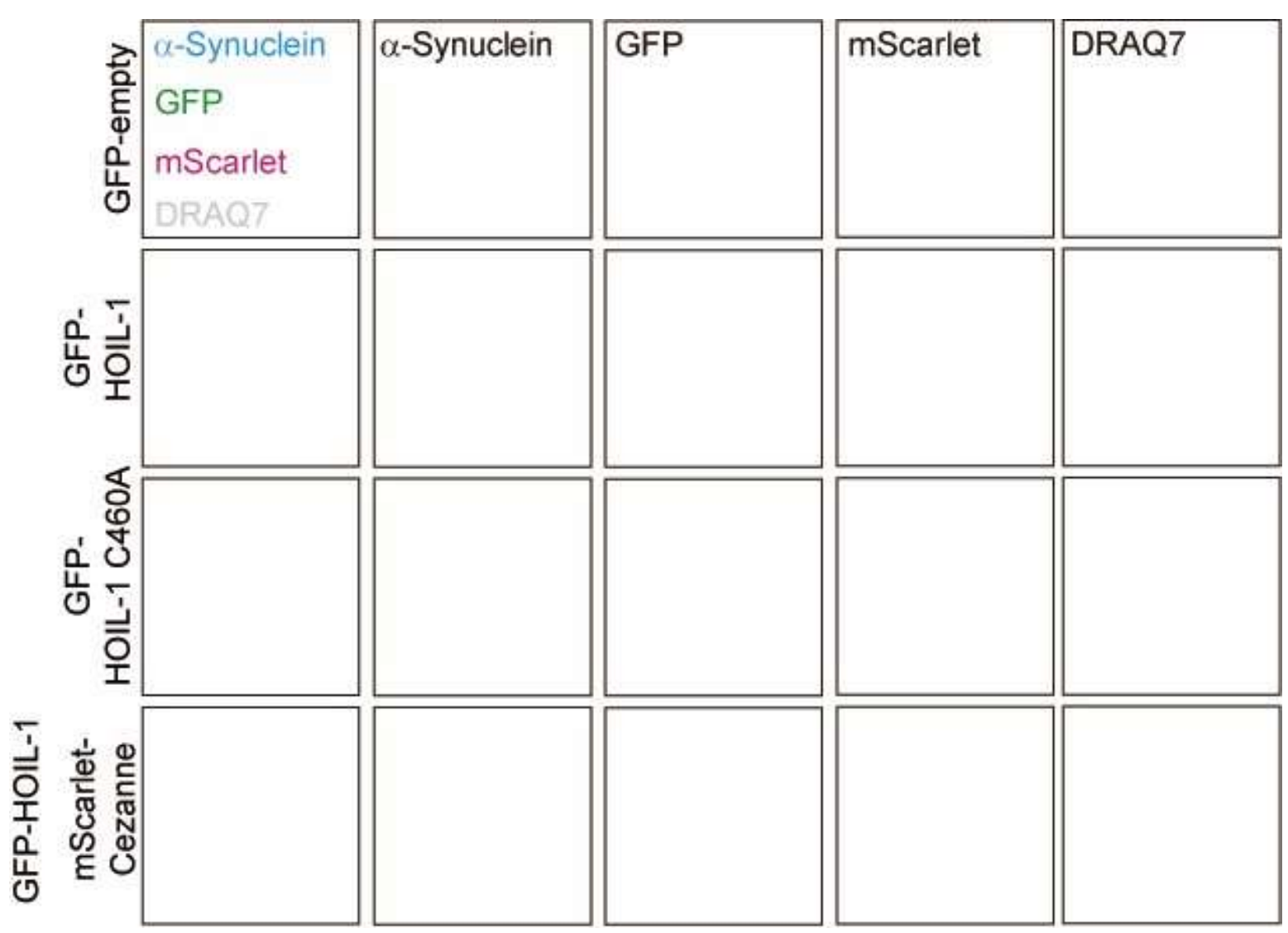

Plan of Cezanne-based ICC analysis

Many/most ubiquitin chains formed in cells contain more than one ubiquitin linkage type. It is therefore important for the authors to perform immunoblots for other ubiquitin linkage types, such as Lys63-linked ubiquitin, and to include these results in Fig 5.

To assess whether Lys63-linked ubiquitin chains are involved, we analyzed the UBAN-enriched fractions for the presence of this linkage type. To date, we have not detected appreciable differences between the conditions examined in Figure 5. Preliminary data supporting this conclusion are shown below.

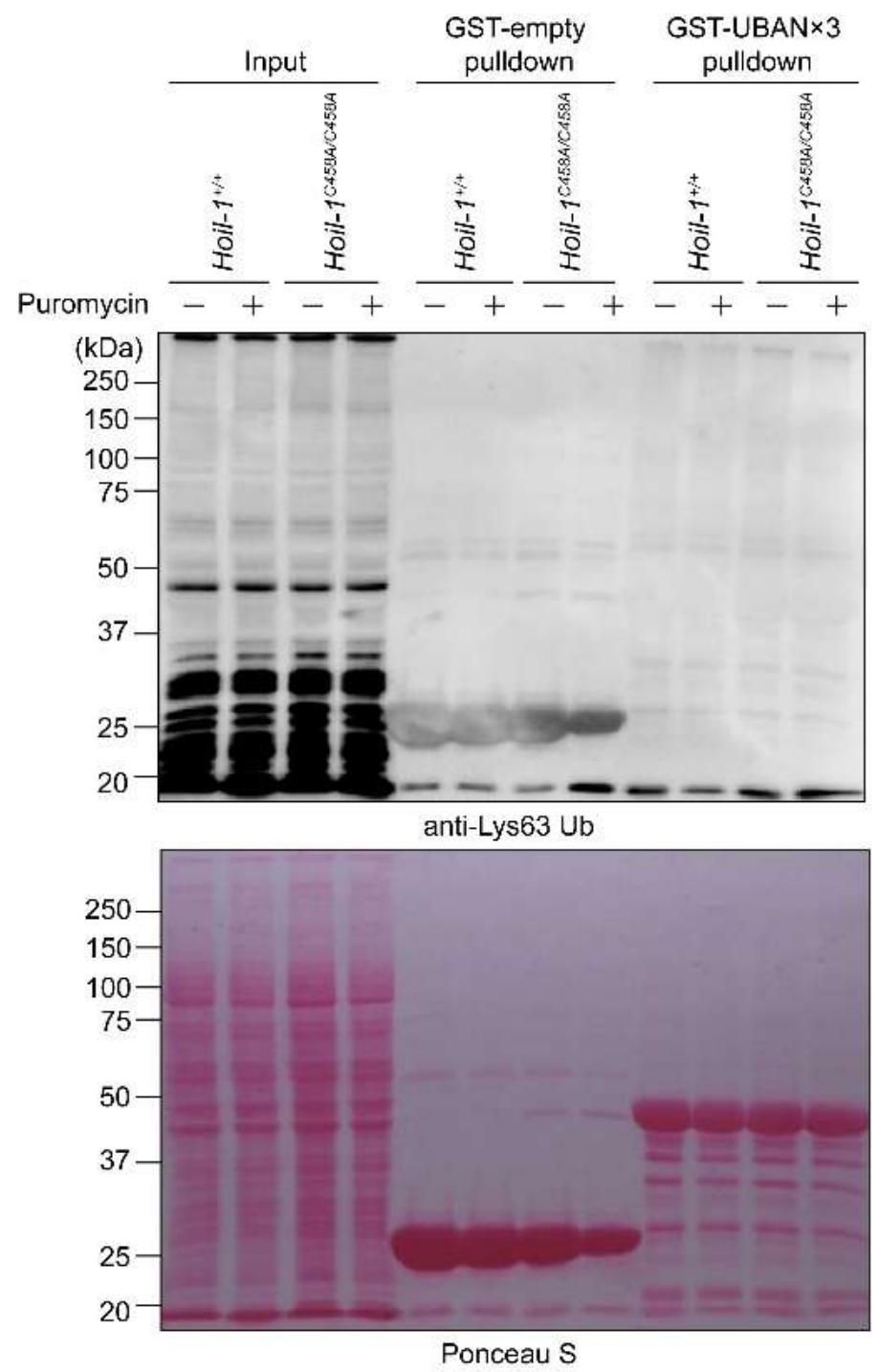

K63-linked ubiquitin chains in MEFs after puromycin treatment

2. The reviewers also think that the authors' claims that the transition of condensate property is linked to elevated Met1-linked ubiquitin chains would be strengthened by performing the biophysical assays (FRAP and 1,6-hexanediol resistance) after Otulin knockdown/knockout (*and ideally also with Otulin rescue*). This will provide direct biophysical evidence linking Met-1 linked chain elevation to condensate liquidity and 1,6-HD sensitivity.

We appreciate these constructive comments. As suggested, we will perform FRAP and 1,6-hexanediol resistance assays in OTULIN knockdown cells. Importantly, our preliminary analyses already show promising trends that support the central conclusions of the study and demonstrate the feasibility of these experiments.

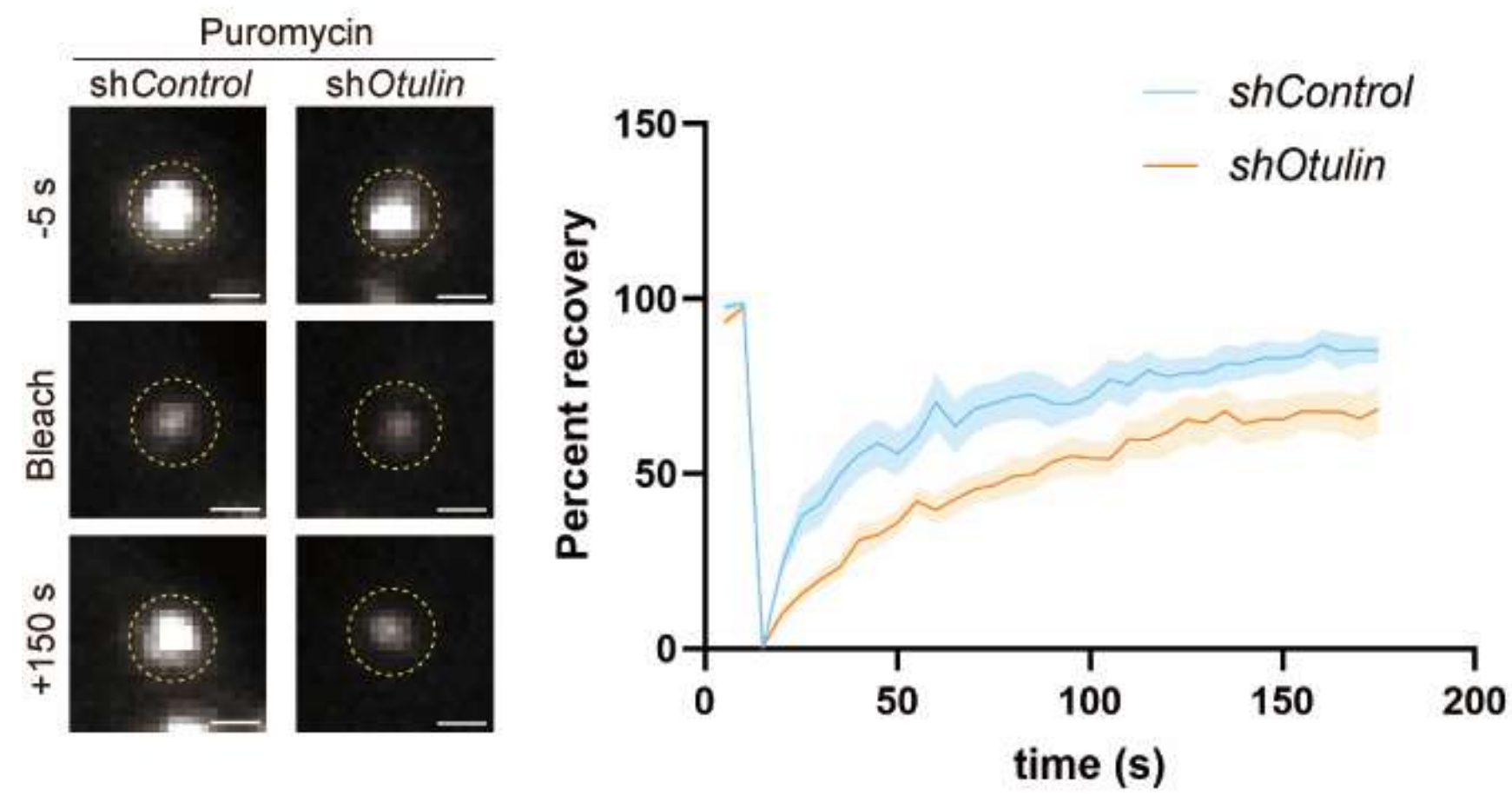

FRAP assay in *shControl* and *shOtulin* MEFs monitoring stress-induced GFP-p62 bodies

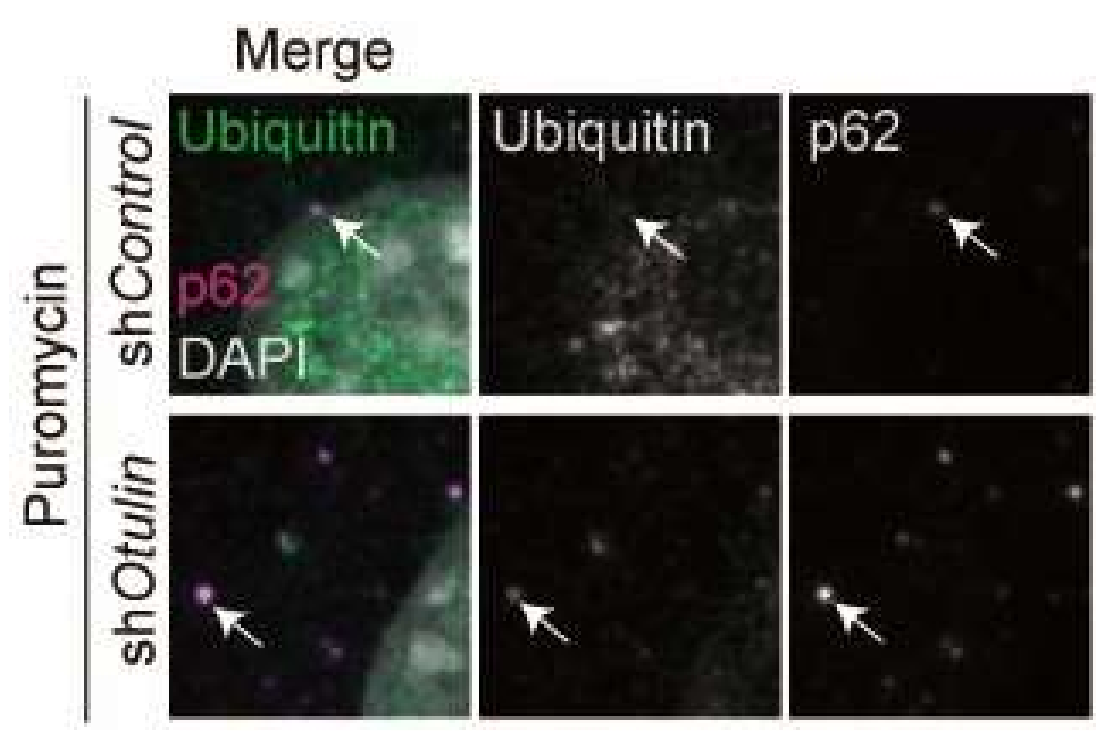

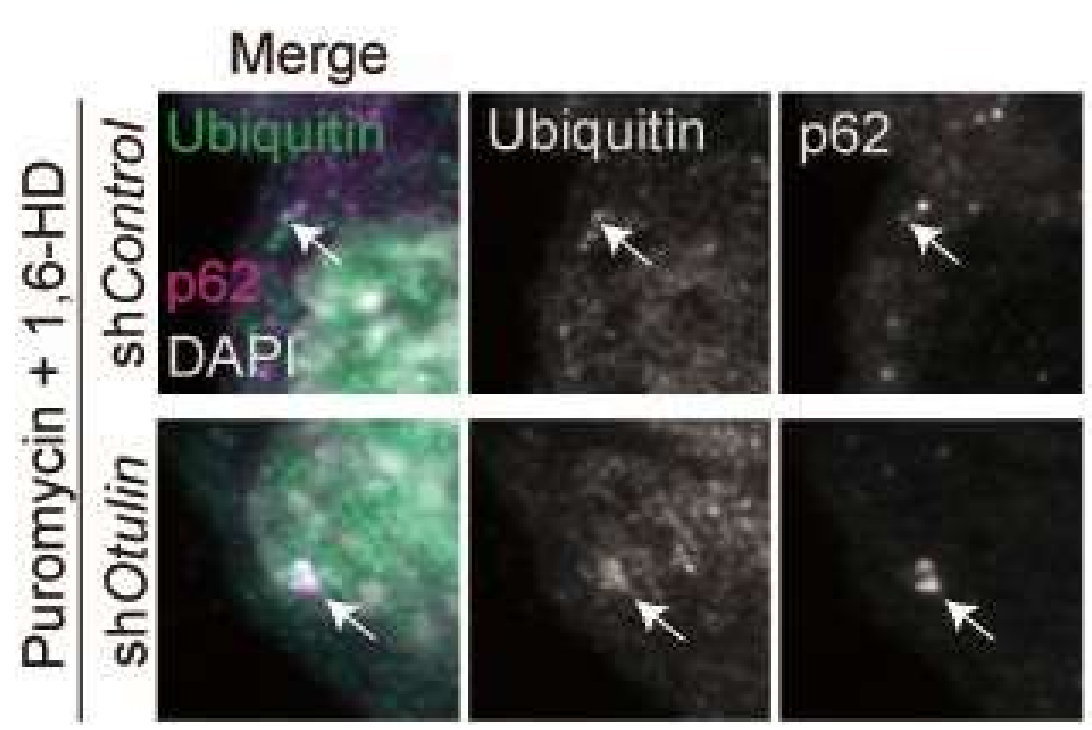

1,6-HD assay in *shControl* and *shOtulin* MEFs

3. The authors have not shown any evidence that Met1-linked chains are more enriched at the sites of protein aggregation. Would the authors be able to demonstrate direct spatial colocalization of Met1-Ub with the analysed aggregates?

We appreciate this important suggestion. We first validated a Met1-linked ubiquitin–specific antibody in sh*Control* and sh*Otulin* MEFs, with and without the E1 inhibitor TAK-243, confirming a specific signal only in sh*Otulin* cells in the absence of E1 inhibition. Using this validated antibody, we will perform the proposed analyses of Met1-linked ubiquitin localization. Our preliminary data already show promising results, as shown below.

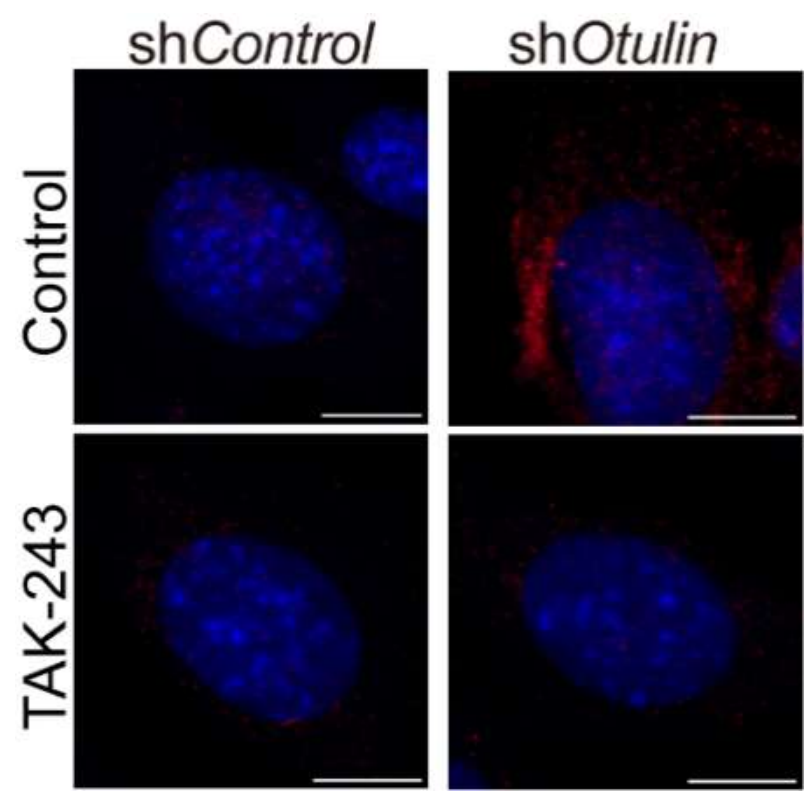

Validation of specificity of the anti-Met1 antibody

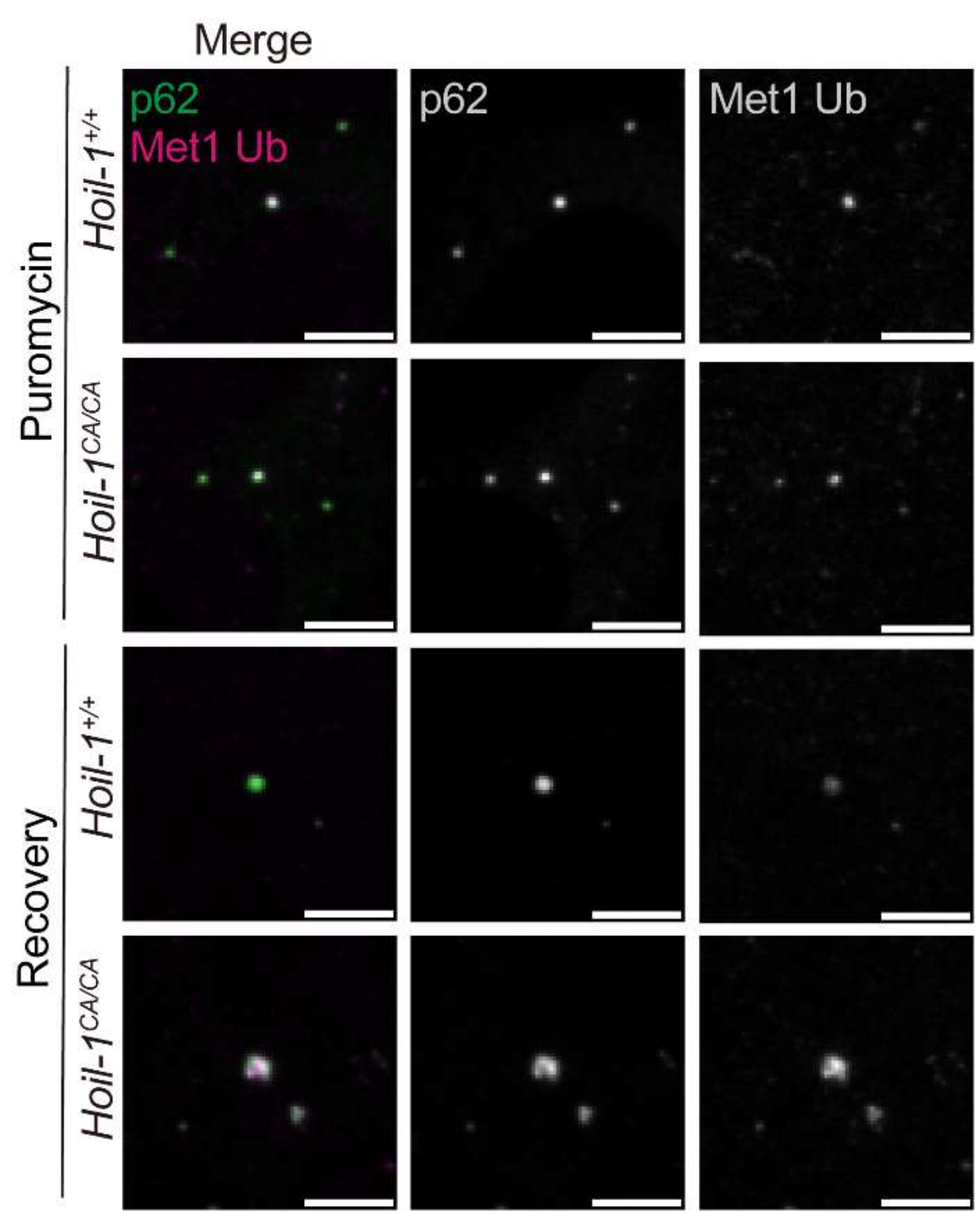

Results of ICC (Met1 and p62)

4. Do the authors know if the effects that they are seeing are general effects on autophagy? For example, is starvation-induced autophagy similarly impaired in the cells studied? A simple flux-style experiment looking at LC3-II levels and p62 with starvations vs puromycin (-/+ bafilomycin) would be informative here.

We will examine autophagic flux under the suggested conditions. Our preliminary data indicate that starvation-induced autophagy is largely comparable between wild-type and HOIL-1 mutant cells, suggesting that the defects we observe are specific to aggregate-targeted degradation rather than reflecting a general impairment of bulk autophagy.

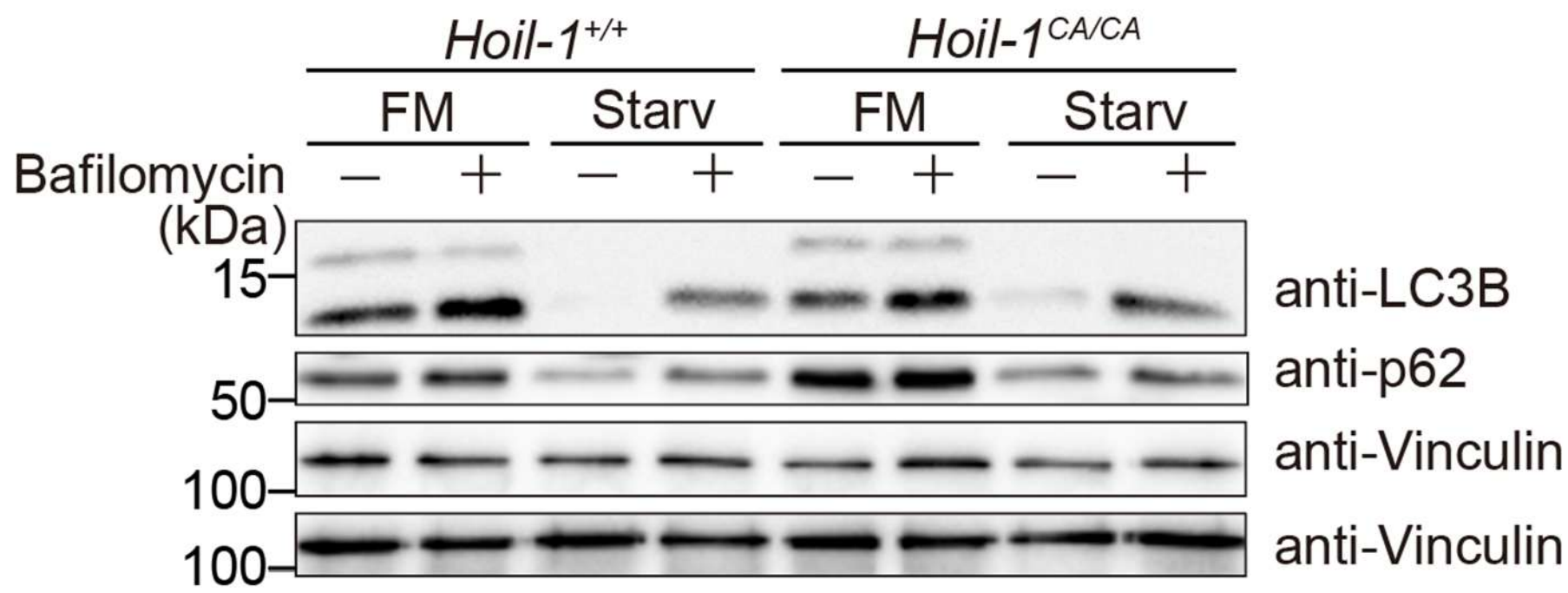

Starvation-induced autophagy flux monitored by LC3B and p62

Minor comments:

5. The authors show that loss of HOIL-1 catalytic activity causes p62 bodies to transition from dynamic liquid-like states to rigid solid-like states and claim this as a more general effect on protein aggregates. But the study does not directly demonstrate a liquid-to-solid transition for the disease-relevant α-synuclein, tau, or Aβ aggregates, limiting the generalisation of the claim beyond p62 bodies. Perhaps the authors should modify the text to better reflect this (or, even better, consider treating α-synuclein/tau/Aβ aggregates with 1,6-hexanediol to measure the response). [optional]

We will revise the manuscript to describe these observations more precisely and clearly, ensuring that the interpretation accurately reflects the data, as suggested.

6. Given that the blots presented in Fig S1A appear to come from different membranes, and high-molecular-weight species of α-synuclein seem to exist in the insoluble pellet fraction of both WT and C460A expressing cells, the reviewers would caution against concluding anything about differences, which can only be assessed if the samples are run side-by-side on the same gel.

We agree with the reviewer that this dataset is not the most appropriate for addressing the point raised. Accordingly, we have decided to omit these data from the revised manuscript to avoid overinterpretation and to maintain analytical rigor.

7. The Methods section says that two different total ubiquitin antibodies were purchased, but which one was used in Figure 5 and other figures are not stated. Please clarify.

We will clarify the antibodies used as suggested in the revised manuscript.

anti-ubiquitin (FK2) (Enzo, BML-PW8810-0500) was used for ICC and anti-ubiquitin (P4D1) (Santa Cruz Biotechnology, sc-8017) was used for WB.

8. On page 10 ABIN1 is mentioned but it is not mentioned that it is the protein product of the TNIP1 gene that is mentioned in the Introduction. This will confuse to many readers.

Thank you for pointing this out. We agree that this could be confusing for readers. We will clarify in the revised manuscript that ABIN1 is the protein product of the *TNIP1* gene when it is first mentioned, ensuring consistency and improving clarity between the Introduction and subsequent sections.

9. 1st paragraph of Discussion line 5 from bottom:- change "oof" to "of".

Thank you for pointing out this typographical error. We have corrected "oof" to "of" in the revised manuscript.

Significance

Prior research has established that the components of LUBAC are recruited to, and are components of, protein aggregates. A link between LUBAC and selective autophagy has also been established previously. The significance of this paper is that it identifies the catalytic function of HOIL-1 as a brake on the activity of LUBAC in proteostasis. The reviewer and co-reviewer are not experts in autophagy or aggregate formation in dementia but, if those reviewers who are find the data presented in these areas to be convincing, then this paper may be the first to suggest a molecular mechanism by which polymorphism/mutation of HOIL-1 leads to increased formation of the aggregates observed in Alzheimer's and other dementias. The results presented in the paper also suggested that initial autophagosome recruitment to aggregates is intact but subsequent late-stage autophagy is impaired. Hence, the study begins to identify the specific step that fails. However, as the authors themselves acknowledge, validation of these potentially exciting findings using in vivo models of neurodegeneration should be the aim of future studies.

The paper combines the molecular dissection of ubiquitin and autophagy pathways to understand the causes of neurodegenerative disease. The paper will therefore be of interest to a broad audience, encompassing both the basic research and clinical research communities.

Reviewers field of expertise: Biochemists and cell biologists with an interest in ubiquitin and cell signalling.

Reviewer #2

Evidence, reproducibility and clarity

Summary:

In this manuscript, the authors show that the branching activity of the E3 ligase HOIL-1, a component of the LUBAC complex, contributes to the autophagic clearance of p62 bodies and

protein aggregates. This activity is attributed to enhanced linear, unbranched ubiquitin chain formation by the second E3 ligase of LUBAC, called HOIP. The model systems employed are cell lines including MEFs expressing a catalytically dead version of HOIL-1. In addition, the authors perform in vitro reconstitution experiments with purified ubiquitin chains, the LUBAC complex and p62. The main message is that solid p62 bodies are poor substrates for autophagy and that linear, non-branched ubiquitin chains promote solidification. The mechanism remains unclear and some of the effects sizes are rather modest.

We appreciate the positive assessment of our integrated cellular and in vitro approaches and the recognition of the key message that HOIL-1–dependent regulation of ubiquitin architecture critically influences p62 body properties and autophagic clearance.

Major comments:

The key observations mentioned above are convincingly shown. Since the authors don't claim any detailed molecular mechanisms, the number of conclusions in this study are limited.

Overall, the authors are quite careful regarding their conclusion, and therefore the ones that are made in this manuscript are generally well supported. The data regarding the clearance of the p62 bodies presented in Figure 3 should be backed up with additional data. The authors could add a macroautophagy inhibitors such as VPS34 IN1 and/or perform the clearance experiments in a ATG KO/KD cell line to corroborate the contribution of macroautophagy to the clearance.
In addition, a proteasome inhibitor should be used for comparison.
The expertise and resources for the experiments mentioned above are expected to be well within the authors' capacity and should be doable within a few weeks.

Thank you for raising this point. We will perform p62-body clearance assays using either a VPS34 inhibitor or MG132, as suggested. We have already acquired the corresponding microscopy images and are currently in the process of analyzing these data.

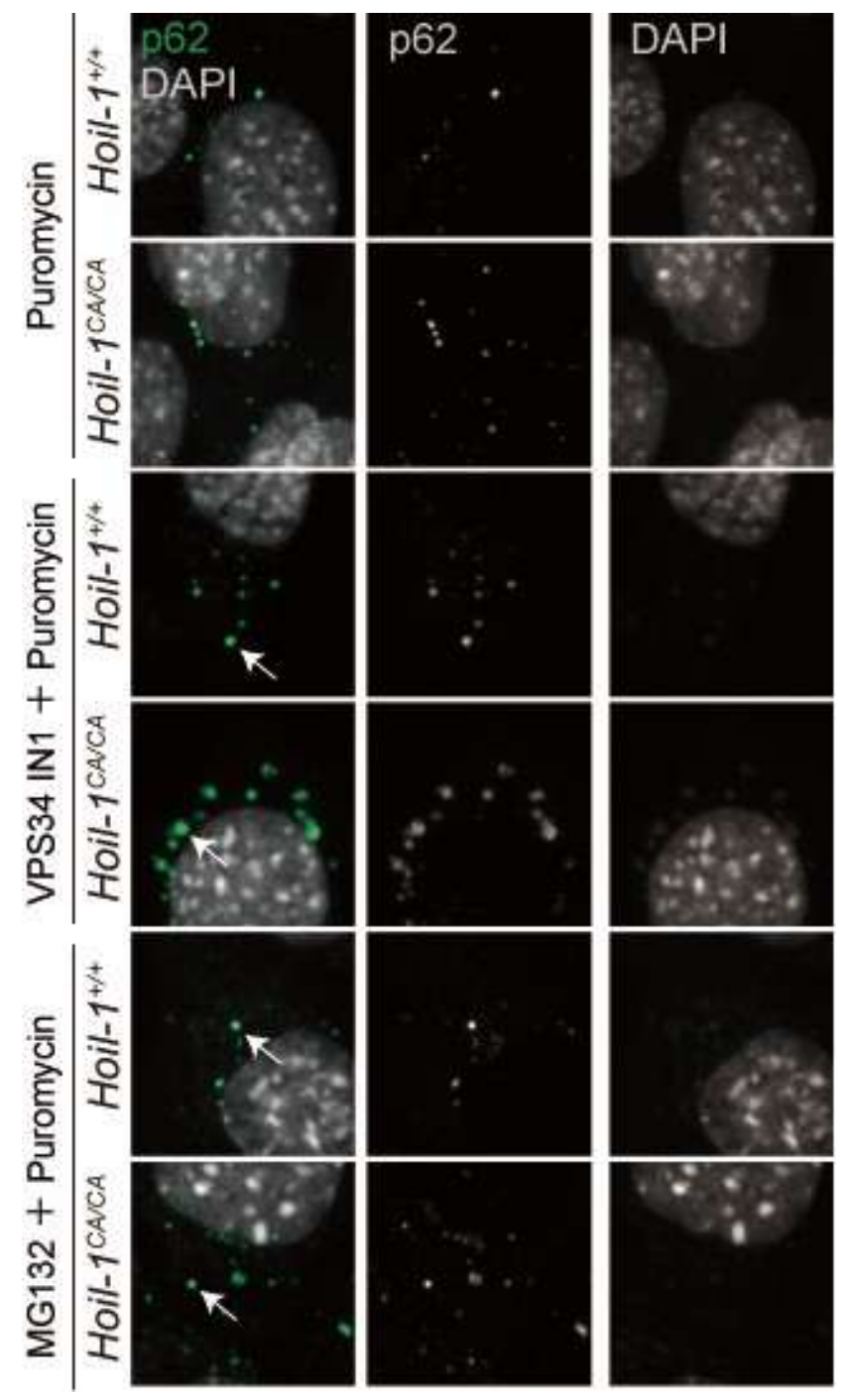

HOIL-1 mutant cells may show bigger puncta under VPS34-IN1 treatment.

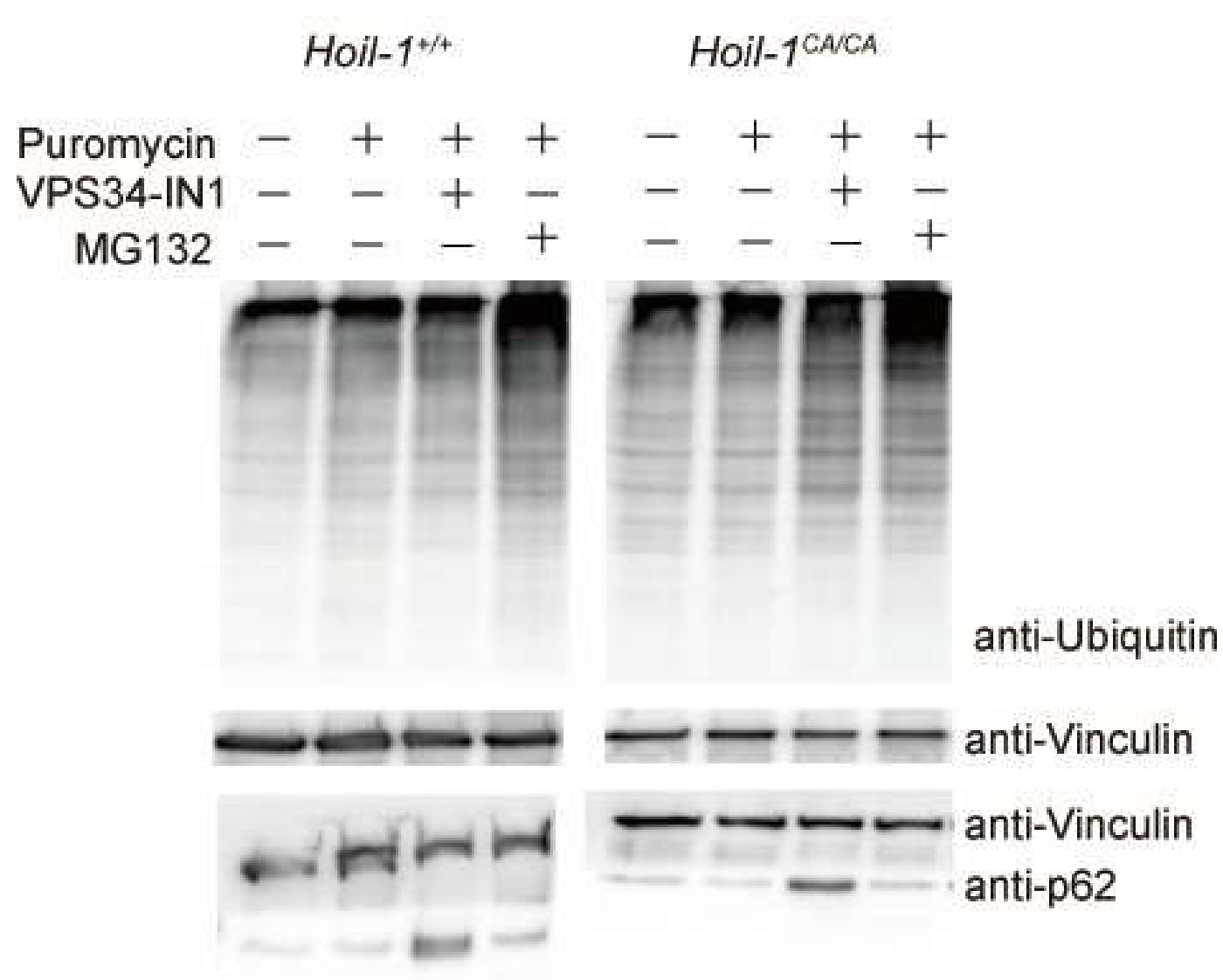

Validation of VPS34-IN1 and MG132 treatment

Some of the effects sizes (e.g. Fig. 5 and S5) are very small and it is possible that some of them are below statistical significance if the number of replicates are increased.

We are currently reanalyzing the data, and with the inclusion of additional data points, we observe increased effect sizes (see below), indicating more robust trends. These reanalyzed datasets appear promising and will replace the previous versions in the revised manuscript.

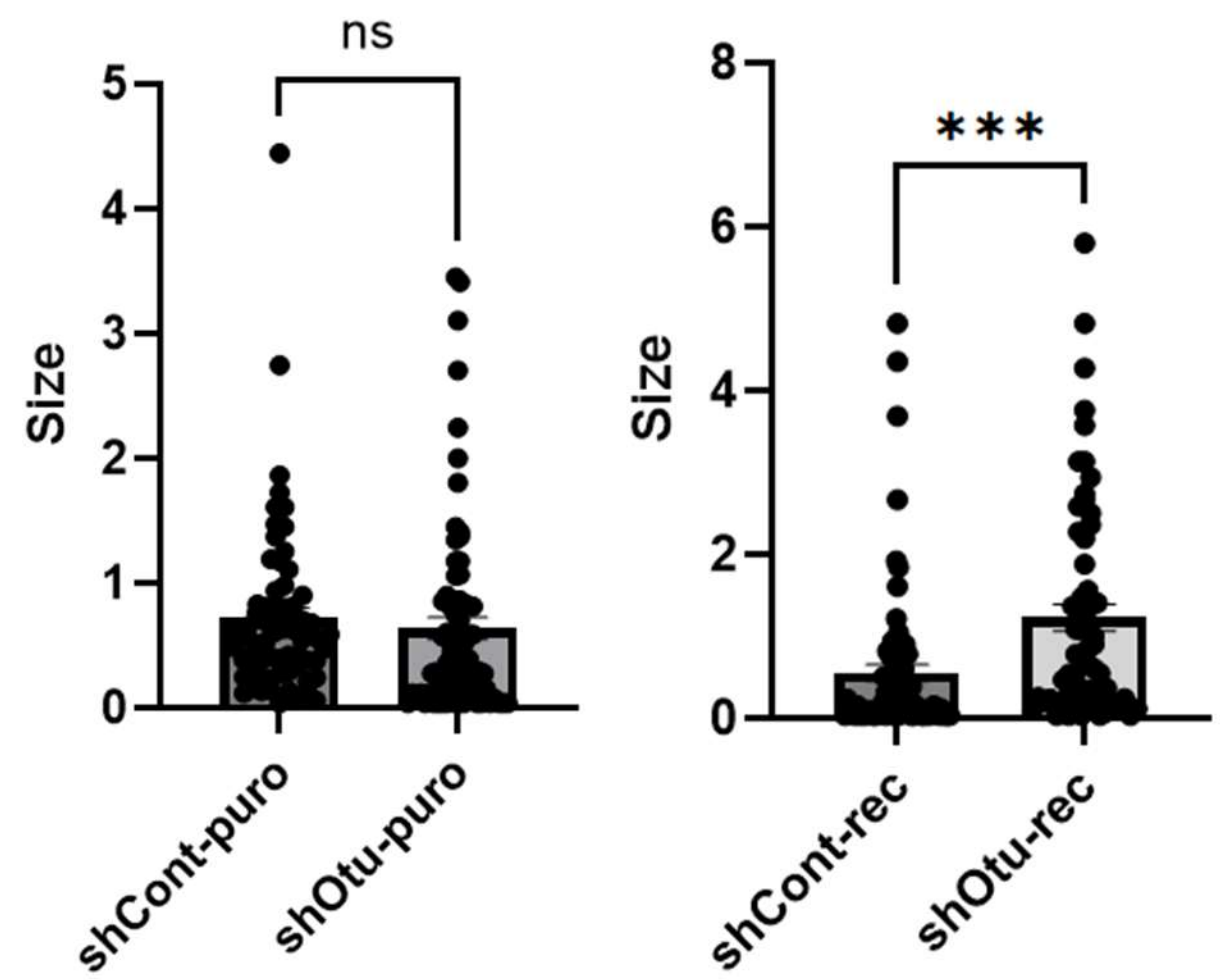

Reanalysis of aggregate size upon stress and after recovery

Minor comments:
Figure 1D should be quantified, for example using PCC, Pearson correlation coefficient.

Thank you for this suggestion. We agree that quantitative assessment would strengthen this result and will quantify the colocalization in Figure 1D using Pearson's correlation coefficient. The corresponding analysis and statistical reporting will be included in the revised manuscript.

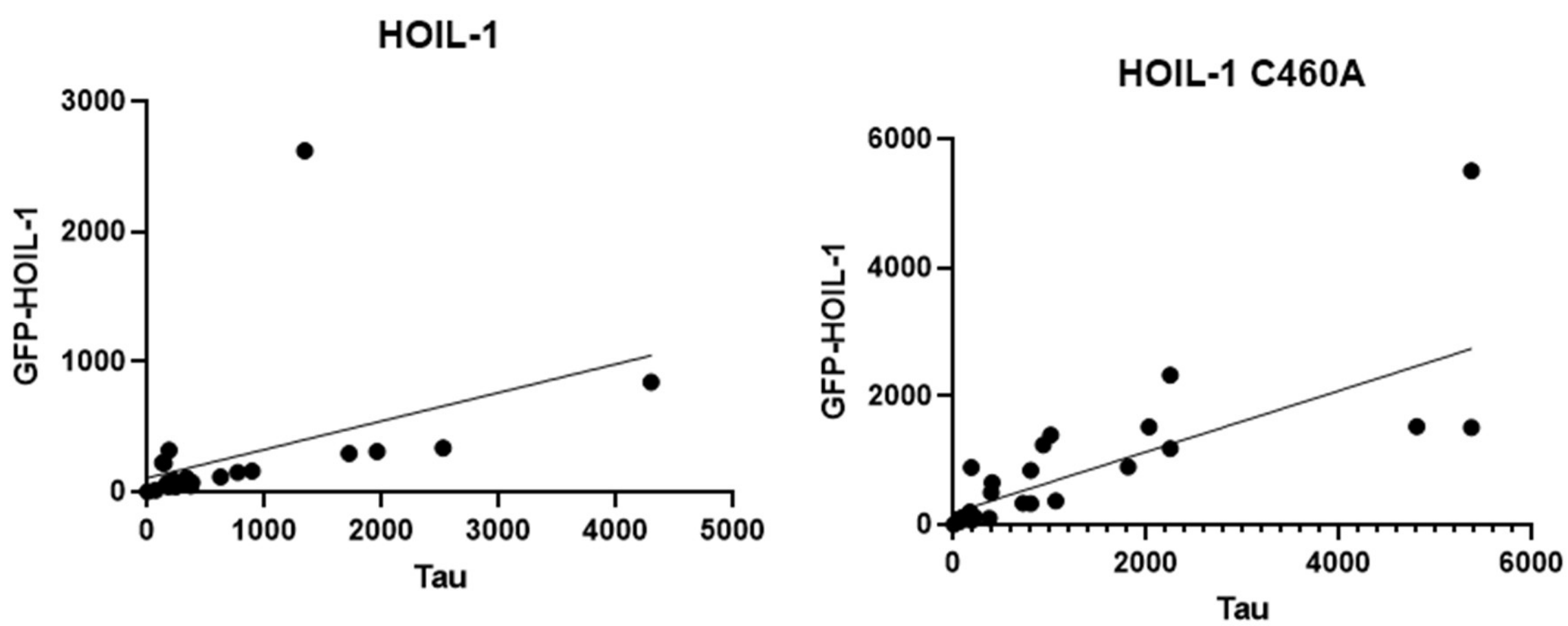

Pearson's correlation coefficient analysis of Tau aggregates with GFP–HOIL-1 or GFP–HOIL-1 C460A expression. Regions of interest (ROIs) were defined based on Tau aggregate fluorescence, and GFP fluorescence intensity was measured within the overlapping regions. Statistical significance was assessed using simple linear regression.
For HOIL-1, R squared = 0.1701, P= 0.0505, ns

For HOIL-1 C460A, R squared = 0.5505, P<0.0001, ****

Figure S1 should be quantified.

Figure S1A will be removed, as the data are not sufficiently convincing. The imaging data shown in Figure S1B have already been quantified and are presented in Figures S1C and S1D, and these will be retained in the revised manuscript.

Figure S3: It should be explained how the region for the profiles are shown were selected.

We will include the following clarification in the revised manuscript to address this point:

"In Figures S3A and S3B, representative p62 puncta from *Hoil-1*$^{+/+}$ and *Hoil-1*$^{C458A/C458A}$ MEFs during the recovery phase were selected, and line fluorescence profiles of p62 and LAMP2 were analyzed. These analyses show that, in wild-type cells, residual p62 puncta are typically enclosed within or surrounded by LAMP2-positive vesicles, whereas in catalytic mutant cells, p62 puncta fail to associate with LAMP2-positive compartments."

This added explanation will improve clarity and guide readers through the rationale and interpretation of the line profile analyses.

It is suggested to include a scheme of the LUBAC complex and its E3 ligase activities in Figure 1A. This will make it more accessible for readers, who are not so familiar with this complex, in particular as HOIP and HOIL can be easily confused.

Thank you for this helpful suggestion. We agree that including a schematic of the LUBAC complex and its distinct E3 ligase activities would improve accessibility for readers less familiar with the system. Accordingly, we will add a simplified schematic to Figure 1A highlighting the respective roles of HOIP and HOIL-1 to clarify their functions and avoid potential confusion as below.

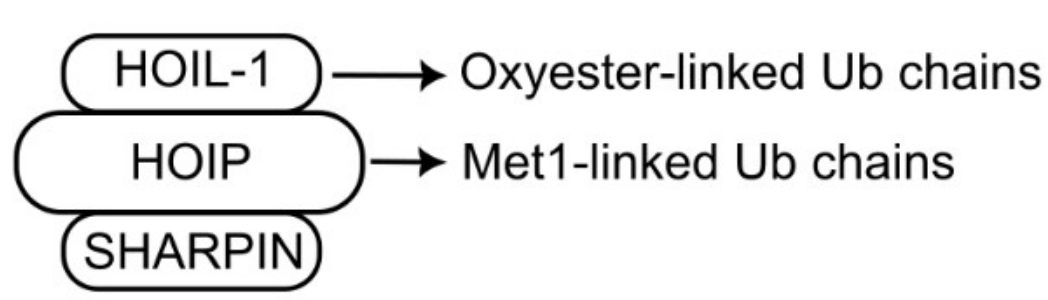

The authors may also want to clarify this in the abstract.

Thank you for the suggestion. We agree that this clarification would be helpful for readers, and we will revise the abstract accordingly to more clearly distinguish the roles of HOIP and HOIL-1 within the LUBAC complex.

Significance

As mentioned in the summary. The authors report the observation that an excess of linear ubiquitin chains produced by HOIP in the absence of HOIL-1 activity results in the solidification of p62 bodies and reduced clearance by autophagy. This observation is novel and will be interesting for the proteostasis field.

This reviewer is expert in autophagy and protein degradation, but less so in the LUBAC complex.

Reviewer #3 (Evidence, reproducibility and clarity (Required)):
Summary:
The study 'Excess Met1-ubiquitination leads to solid aggregate formation' by Kaypee et. al suggests a previously unrecognised role for the E3 ligase HOIL-1 in clearing protein aggregates via autophagy (e.g. aggrephagy). In their model, toxic protein aggregates in cells are modified with ubiquitin chains, including M1-linked Ub-chains catalysed by LUBAC (of which HOIL-1 is a component). The HOIL-1 ubiquitin signal is posited to induce trafficking of aggregates to lysosomes for subsequent clearance. However, when HOIL-1 is inactive (catalytic C460A mutation), the pathway is interrupted. As a result, protein aggregates fail to clear, they increase in size and shift their biophysical properties from liquid-like to more rigid, insoluble aggregates. The authors explain their observations by an increasing amount of M1-linked chains on protein aggregates, which occur as a result of 'unrestrained HOIP activity' due to HOIL-1 inactivity (based on previous work). Increasing amounts of M1-chains are posited to promote aggregate formation, aggregate growth, and prevent clearance.
The major claims made in this manuscript are the following:
1. Following the induction of protein aggregate formation (e.g. alpha-synuclein, tau, beta-amyloid, p62 bodies), cells that express catalytically inactive HOIL-1 fail to clear protein aggregates and end up with more, larger and more rigid protein aggregates compared to cells that express WT HOIL-1.
2. The observations made in 1. are due to disruptions in late-stage autophagic flux. While aggregates in cells that express WT HOIL-1 co-localise with autophagy and lysosomal markers, aggregates in cells that express mutant HOIL-1 show autophagic, but not lysosomal markers.
3. An increase in M1-chains (either due to HOIL-1 inactivity or due to OTULIN knockout) is believed to be the cause for claims 1. and 2.

Main methodologies used:
The authors use two cellular systems. The first one is SH-SY5Y cells in which either WT or mutant HOIL-1 are transiently overexpressed (via the pcDNA3.1 plasmid), and physiologically important aggregates (Tau, Abeta, asyn) are induced. The second cellular system is MEF cells in which either WT or mutant HOIL-1 are endogenously expressed; in these cells aggregates are formed crudely through disruption of ribosomal translation. It is questionable if both systems can be compared. Aggregate formation is mainly monitored and quantified via fluorescent microscopy in both fixed and live cells, or via sucrose gradient fractionation to separate soluble and insoluble (=aggregate) fractions. The rigidity of protein aggregates is analysed in cells via FRAP, size and circularity measurements and 1,6-HD treatment, or in vitro after aggregate formation assays via size and circularity measurements.

The observations are on the whole interesting, though the authors fail to discuss their data in light of previously published work. For example, HOIL-1 KO and KI animals were shown to feature polyglucososan bodies in brain, which is not mentioned. Also, McCrory et al on HOIL-1 chain types is not cited but seems relevant (Figure S4). Yet, the manuscript reports a number of interesting findings, more or less coherently, most useful for scientists embedded in current ubiquitin, autophagy, and LLPS fields. These reviewers believe that this manuscript will make a lot of sense in due course, and be well cited for a first description of the role of HOIL-1 in cellular quality control processes.

A number of improvements seem required to consolidate the findings, and improve readability and impact.

We thank the reviewer for recognizing the interest, coherence, and potential impact of our findings for the ubiquitin, autophagy, and LLPS communities. We appreciate the constructive literature suggestions and will incorporate the cited studies, including polyglucosan body in the brain in HOIL-1 animal models (Kelsall et al., 2022) and McCrory et al., to strengthen the manuscript.

Major:

1. Figure 1A-C: The authors transiently overexpress either WT HOIL-1L or catalytically inactive (C460A) HOIL-1 in SH-SY5Y cells, then induce and compare the formation of protein aggregates (alpha-synuclein, tau, amyloid-beta) in those cells over 72 h. More cells with aggregates were found in cells that overexpressed mutant HOIL-1L. While these findings are interesting, the cellular system used is artificial due to the transient overexpression of HOIL-1 (in presence of endogenous HOIL-1). Crucial controls are missing:

We thank the reviewer for these valuable insights. In response, we plan to carry out the following experiments to address points (a–d) and strengthen the corresponding conclusions.

a. Adding a condition in which no protein is overexpressed, for example via an empty pcDNA3.1 or GFP only vector. This would help ruling out secondary effects due to the transient overexpression. It would also allow to monitor whether the same amount of aggregates form in the empty ctr compared to when WT HOIL-1 is overexpressed.

Yes, we will include data from a GFP-only control in the revised manuscript. As expected, expression of the GFP-only vector shows little to no difference compared with HOIL-1 WT. A preliminary version of these data is shown below and will be fully incorporated in the revised figures.

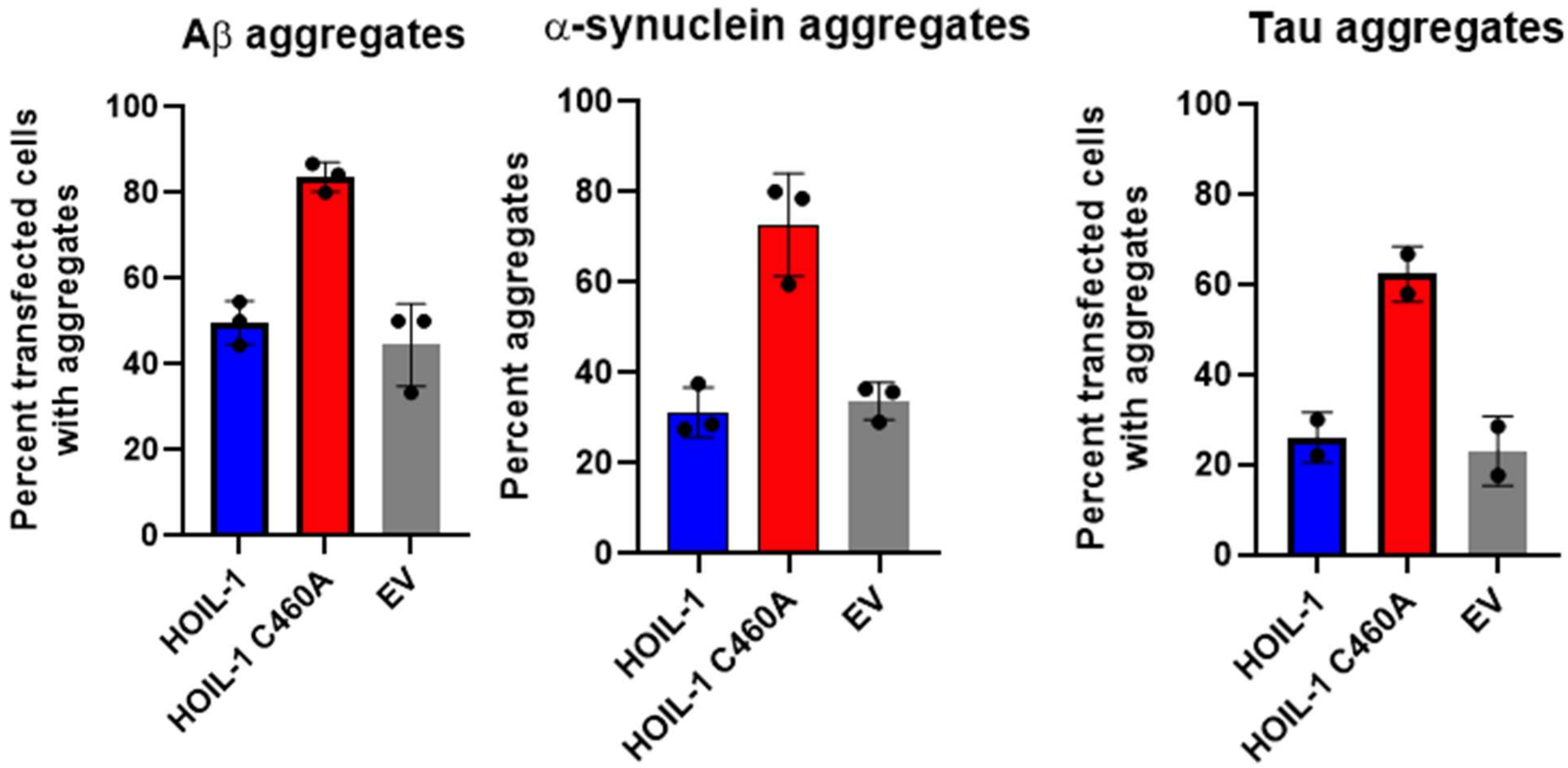

Preliminary data including GFP (empty vector/EV) control

b. Figure legends and raw data points (?) in graphs do not match. The graphs show dubious statistics from 2-3 grey dots, while the figure legend refers to n=100 cells etc. This needs to be fixed.

We apologize for the confusion. The cells were counted as originally described, based on three independent experiments. We will clarify this explicitly in the revised manuscript to ensure that the experimental design and statistical basis are clear to readers.

c. Showing Western Blots of HOIL-1, to better understand the levels of endogenous HOIL-1 vs overexpressed HOIL-1 in these cells, and to compare overexpression levels between WT and mutant HOIL-1.

We thank the reviewer for pointing out this issue. In response, we will include immunoblot data directly comparing endogenous HOIL1 levels with those of transfected untagged HOIL-1, allowing a clearer assessment of expression levels and strengthening the interpretation of the overexpression experiments.-

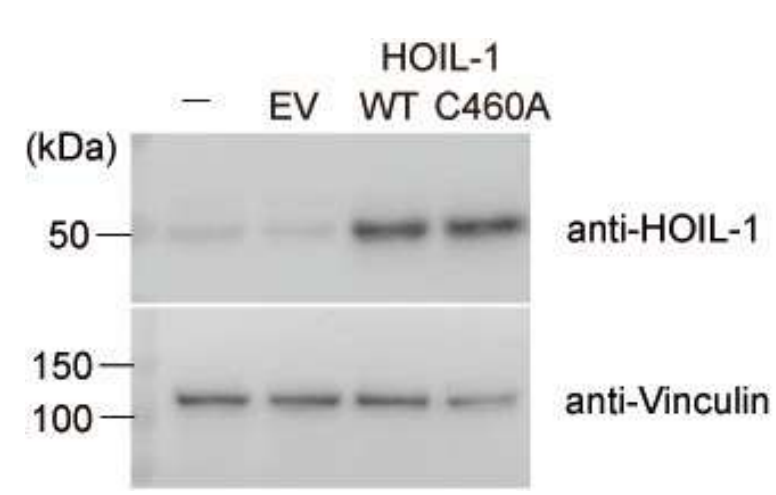

Expression levels of endogenous and transfected HOIL-1

d. The study would also improve by western blotting and IF staining for other LUBAC components such as HOIP and SHARPIN. Do alpha-synuclein aggregates in both WT and mutant conditions co-localize with the other LUBAC components, and are there any differences between WT and mutants. This would further help strengthening the claims made in Figure S1A: '...suggesting that LUBAC is recruited to or retained within α-Synuclein aggregates.' And in the discussion: 'we found that LUBAC components were sequestered in aggregates, as evidenced by microscopy and gradient fractionation of soluble and insoluble proteins, confirming the direct involvement of LUBAC in aggregate processing.'

This is indeed a critical point, and we acknowledge that we did not previously have direct evidence addressing it. To resolve this issue, we agree that the most appropriate approach is immunofluorescence/immunocytochemistry to directly assess the recruitment of LUBAC components to α-synuclein aggregates. As suitable antibodies for ICC detection of endogenous SHARPIN and HOIP are currently unavailable, we have elected to use epitope-tagged versions of SHARPIN and HOIP. Construction of these plasmids is currently being finalized, and these experiments are underway.

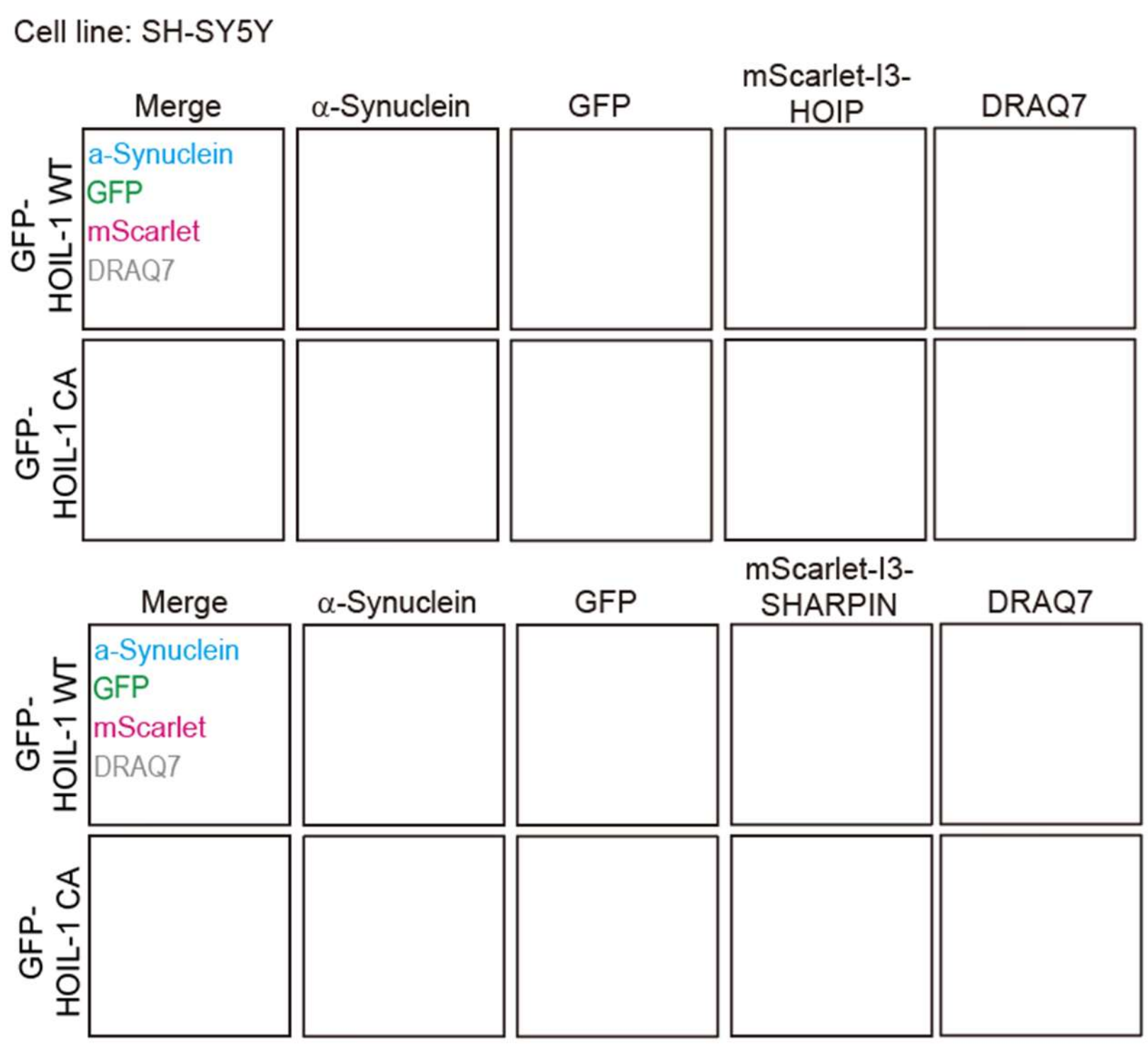

Planned experiments of ICC monitoring LUBAC components in aggregates

2. Figure 2A-F: The authors change to a genetic-derived system (comparing endogenously expressed HOIL-1 WT with mutant HOIL-1 based on MEF cells from their mouse models). However, they use puromycin to produce aggregates from random protein homeostasis defects, which yes leads to aggregates, but is not as nice as the induced generation of neiurodegeneration-relevant aggregates. It was observed that after 2 h of puromycin treatment, cells accumulate p62-positive protein aggregates, and in during recovery (2 h washout), the

aggregates in the HOIL1 mutant cells outgrow the aggregates in the WT HOIL1.

a. However, the authors claim that: 'While Hoil-1+/+ MEFs efficiently cleared puromycin-induced p62 bodies,...', which is not supported by the data shown here. When comparing WT in panel C with WT in panel E, it becomes evident that the average number of p62 puncta before and after recovery is the same (around 5 puncta/cell in both pre and post washout conditions). A similar observation can be made for the mutant (around 12 puncta/cell in both pre and post washout conditions). Can the authors please amend their claims, or comment and perform a direct statistical comparison between the pre and post recovery conditions to test for clearance of p62 puncta in the WT after puromycin washout.

We thank the reviewer for pointing this out. We agree that our previous description was not sufficiently precise and will revise the text to clarify that the **key difference lies in the size of the aggregates rather than their number**, with larger p62 bodies being more prominent in panel E compared with panel D. We will ensure that this distinction is clearly explained and not overstated. Consistent with this interpretation, in Figure S2 we observe differences in aggregate size but not number; these data will be added to the revised manuscript.

We will revise the text accordingly:
“After recovery from puromycin treatment, *Hoil-1*$^{+}$/$^{+}$ MEFs exhibited fewer large p62 bodies compared with *Hoil-1*$^{C485A/C485A}$ MEFs.”

b. The authors state that: 'These findings indicate that although HOIL-1 catalytic activity is dispensable for the initial formation of puromycin-induced aggregates, it is essential for their subsequent clearance.'
As long as clearance of p62 bodies in the WT is not clearly shown, the second part of this sentence should be amended/removed.

We agree with the reviewer and will rephrase this part to accurately reflect the data in the revised manuscript.

c. The experiments shown would improve by adding a t = 0 condition. How many p62 granules are present before puromycin treatment? Is there already a basal difference between WT and mutant HOIL-1L cells?

This is another important point. We will include a dataset obtained in the absence of puromycin treatment as a baseline control. Under these untreated conditions, we observe minimal to no p62 signal in either wild-type or HOIL-1 mutant MEFs, indicating that aggregate formation is specifically induced by proteotoxic stress.

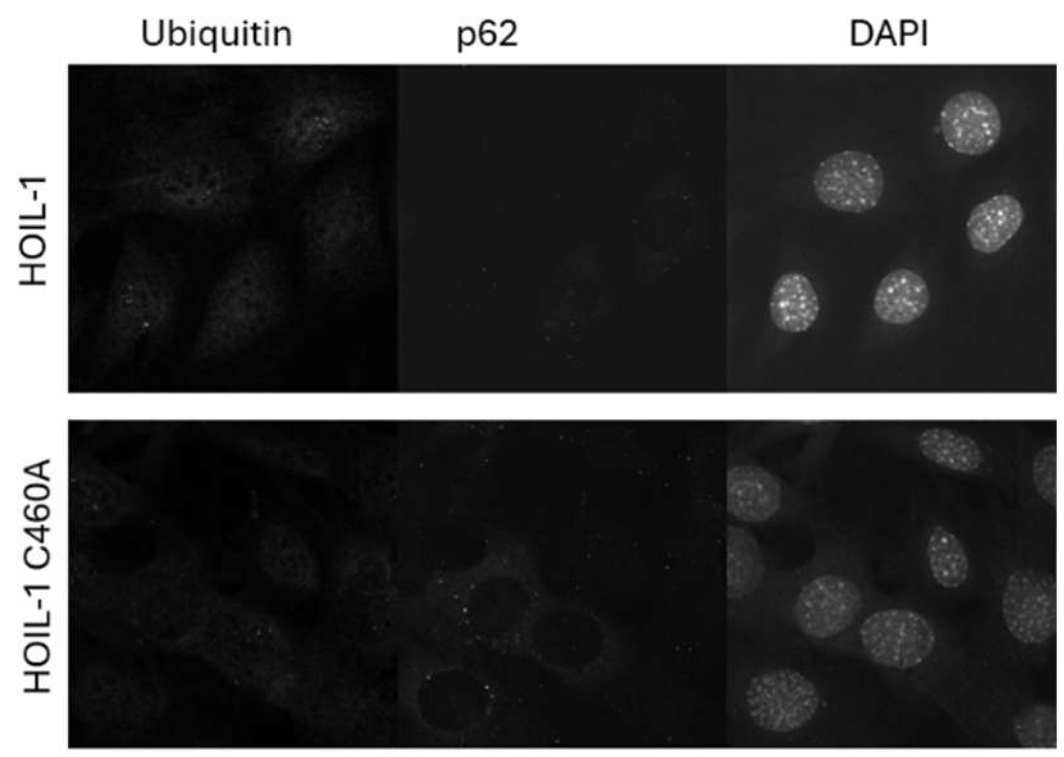

p62 body signals by ICC without puromycin

3. Figure S1A: The authors claim that other LUBAC components co-localise to protein aggregates, based on sucrose gradient fractionation and the presence of the respective proteins in the insoluble fractions. Could the authors perform IF and stain for other LUBAC components (SHARPIN and/or HOIP) in their MEF cell system to directly validate this claim?

This is a critical point, and we have carefully considered it. As also noted by Reviewer #1 (point 6), we have decided to remove this dataset and the accompanying text to avoid overinterpretation. However, as suggested in point 1(d), we plan to directly analyze the presence of LUBAC components within aggregates using immunocytochemistry in the revised study.

4. Figure 3G-H: The authors created a GFP-mCherry-p62 reporter system in both their WT and mutant HOIL-1 MEF cells and performed live cell imaging following puromycin treatment, which allows monitoring of both aggregate formation and loss of GFP signal due to the acidic lysosomal localisation. Excitingly, the ratio of GFP/mCherry in the later timepoints is reduced in the WT compared to mutant HOIL-1, indicating that HOIL-1 activity is required to traffic p62 bodies to lysosomes.

a. In panel G, a surprisingly large amount of p62 granules are present at t0, which (according to the relevant method section) is the time of puromycin treatment. This observation can be made for both WT and mutant cells. After 80 min of puromycin treatment in the WT, the majority of these puncta are cleared. Can the authors please comment on this high amount of p62 granules at t0 (before the effects of puromycin? And also on the observation that after 80 min there are now less granules than before puromycin? In case that t0 indicates the time of puromycin washout rather than puromycin addition, could this please be clarified in the methods or figure legend?

We apologize for the confusion. We will clarify the methodology in the revised manuscript to state that the time zero for live-cell imaging corresponds to **30 minutes after puromycin treatment**, at which point induced p62 bodies are already present. This time point does not represent an untreated condition and reflects technical constraints of the imaging setup.

b. Panel H would improve by adding the quantifications for t=0 (or ideally for all the time points).

We will add the corresponding data point and clarify that there are minimal to no p62 bodies at the true time-zero condition, consistent with the description in point 2(c). This clarification will be explicitly stated in the revised manuscript to avoid ambiguity.

c. Fig S3C-D: Same comments as before but for GFP-mCherry-LC3.

We will add data points and clarify for GFP-mCherry-LC3.

5. Fig. S4B and Fig 4A-B: The authors state that circular aggregates are more soluble and have more LLP characteristics, whereas non-circular aggregates are less soluble and have more aggregate-like characteristics. However, the aggregates shown in Fig. S4B are un-circular but easily dissolve in response to 1,6-HD treatment, which seems contradictory. On the other hand, the aggregates shown in Fig 4B in HOIL-1 mutant cells appear much rounder than the ones in S4B, but do not dissolve in response to 1,6-HD treatment. Can the authors please comment on these discrepancies?

Thank you for raising this important point. We agree that circularity alone is not a definitive proxy for material state, and that apparent discrepancies can arise depending on context. In our assays, **circularity reflects geometry**, whereas **1,6-hexanediol sensitivity reports material properties**, which are influenced not only by shape but also by underlying molecular interactions.
In Figure S4B, the non-circular condensates likely represent **irregular but still liquid-like assemblies**, consistent with their sensitivity to 1,6-hexanediol. In contrast, the rounder aggregates observed in Figure 4B in HOIL-1 mutant cells likely reflect **matured, compact structures** whose apparent circularity arises from surface tension during solidification, yet are resistant to 1,6-hexanediol due to stabilized intermolecular networks.
We will clarify in the revised manuscript that circularity and hexanediol sensitivity capture **distinct and complementary properties**, and that aggregate shape alone should not be interpreted as a direct measure of liquidity.

6. Fig. 4E-G. Here the authors suddenly switch to an in vitro aggregate-formation assay using mCherry-p62. In-vitro M1-chain reactions with either WT LUBAC or LUBAC with mutant HOIL-1L, together with the respective M1-chain reaction product, are added.
This is not clear from the figure, and a schematic, as well as a gel (Coomassie) should be included to show component purity and indicate the biochemical in vitro nature of the experiment. It is good to have this breadth of methods, but does not help in the presentation if all figures look alike.

We thank the reviewer for this constructive comment. In response, we will include Coomassie-stained gels to document component purity and will add schematic illustrations, as outlined below, to clearly convey the experimental setup.

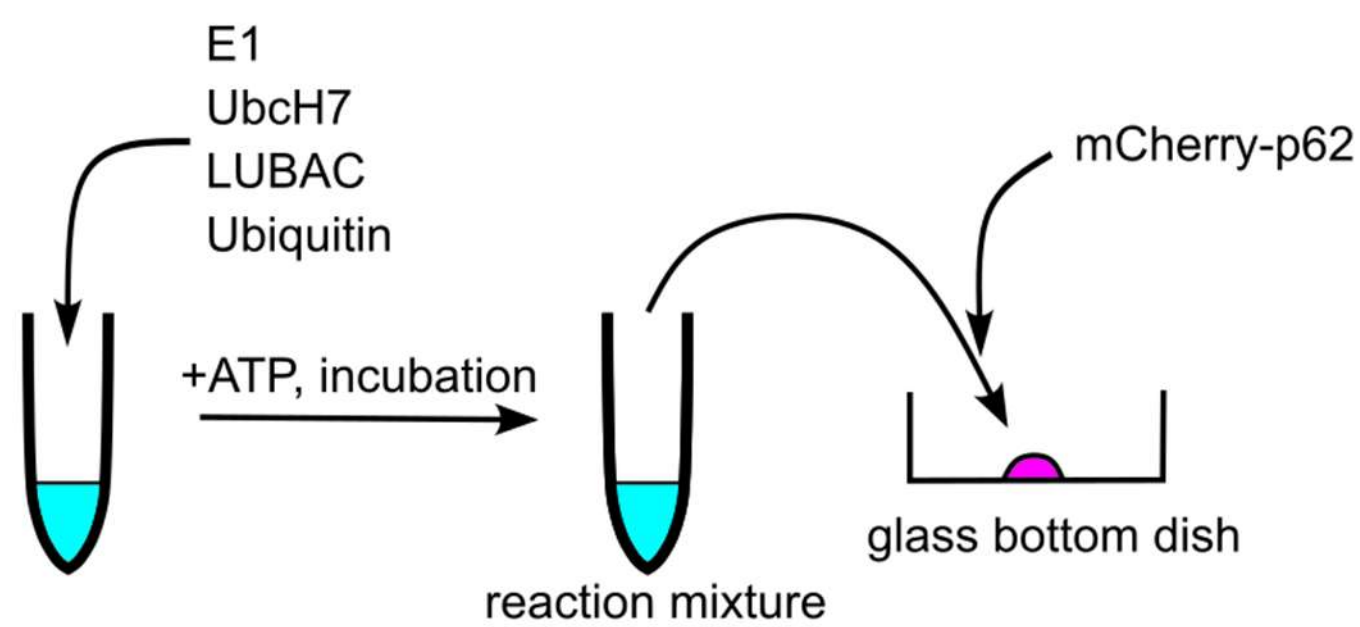

a. The key difference to the cellular situation is p62 aggregates are not directly ubiquitinated here, and instead ubiquitin chains are (non-covalently) added to samples. Can the authors please make this important difference clearer in their text? Why not directly ubiquitinate mCherry-p62 via LUBAC (WT vs mutant HOIL-1) and then perform an aggregation assay on the reaction product?

We thank the reviewer for raising this point. This is indeed a very interesting yet technically challenging issue—namely, identifying the relevant substrate(s) of LUBAC in the regulation of aggregate or condensate formation. In this study, we focused on ubiquitin itself as a primary substrate, reasoning that LUBAC-mediated formation of unconjugated ubiquitin chains can profoundly influence condensate properties. However, given the central role of p62 in this pathway, it is also plausible that p62 is directly ubiquitinated by LUBAC. To address this possibility, we will perform in vitro ubiquitination assays using p62 as a substrate, and if p62 is found to be ubiquitinated, we will subsequently assess the impact on LLPS using these reaction products.

b. Can the authors please clarify whether the reaction was inactivated prior to addition to the aggregate-formation assay? If not, the enzymes might still be active at the point of aggregate formation, and the observed effects might be influenced by enzymatic activities and not only the presence of different M1-chain architectures.

This is an interesting point. In our *in vitro* ubiquitination assays, samples are snap-frozen after incubation, indicating that residual enzymatic activity may persist in both wild-type LUBAC and LUBAC containing the HOIL-1 mutant. Fully inactivating LUBAC under these conditions is technically challenging, as additional inactivation steps lead to ubiquitin chain aggregation. Nevertheless, because clear differences are consistently observed between samples in which HOIP remains active in both conditions, we interpret the observed effects as specifically attributable to HOIL-1 ligase activity. We will make this rationale explicit in the revised manuscript.

7. Fig. 5B-C: The M1-specific DUB OTULIN is knocked down (again, cells) to increase the overall amount of M1-linked Ub-chains present in cells. P62 aggregate formation is induced and the authors claim that the increase in M1-chains influences aggregate size. This claim would be strengthened if it was directly shown that M1-chains form on p62 aggregates in this assay, for example via IF using an M1-antibody (and potentially a total ubiquitin antibody). This would also enable to directly compare the abundance of M1-chains between conditions (ctr vs Otulin, WT

vs HOIL-1L mutant).

We appreciate the reviewer for raising this important point. Having validated the specificity of the Met1-linked ubiquitin antibody for immunocytochemistry, we will perform the suggested experiments using this approach in the revised study.

Minor:

1. Figure 1 D: The authors state that 'Notably, HOIL-1 C460A was detected within these structures, as demonstrated by its colocalization with tau aggregates' and show a co-localisation comparison between WT and mutant HOIL-1L. This sentence implies that WT HOIL-1 was not detected in aggregates, however the chosen image of the WT cells does not show any obvious tau aggregate, even though aggregates were induced in this condition according to 1A-C. The better comparison would be to pick an image that includes a tau aggregate. Moreover, this experiment would benefit from quantification, calculating the percentage of total aggregates that co-localise with WT HOIL-1L vs with mutant HOIL-1L.

We thank the reviewer for raising this point. In response, we will analyze these data as outlined in our reply to Reviewer #2, minor comment 1, and incorporate the corresponding quantification and clarification in the revised manuscript.

2. Figure S1A: The authors state that 'Cells expressing HOIL-1 C460A displayed a pronounced accumulation of high-molecular-weight α-Synuclein species in the insoluble pellet fraction.' While the difference seen by Western Blot is apparent and seems to match Fig 1 A-C, it is not very strong. Moreover, the relevant comparison (WT vs mutant) is made between two different blots/membranes, and it is difficult to assess equal input solely based on the TCL lane. This experiment could be improved by normalising samples (for example via BCA) and by loading and imaging the two conditions (or at least the TCL and pellet samples) on the same membrane.

The authors also state that: 'LUBAC subunits, HOIP, HOIL-1, and SHARPIN, were also enriched in this fraction, suggesting that LUBAC is recruited to or retained within α-Synuclein aggregates.' Both Sharpin and HOIL-1 seem to be present in similar levels in the pellet fractions of WT and mutant HOIL-1. Overall HOIP levels seem to be significantly increased in the mutant over the WT (see TCL lane), and to a similar level in the pellet fraction. It would be great if the authors could include these observations in their interpretation.

Since the data shown in the original Figure S1A are not sufficiently convincing, we will remove this panel and the associated text from the revised manuscript. To address the underlying question more rigorously, we are instead performing immunocytochemistry to directly assess whether other LUBAC components, specifically SHARPIN and HOIP, localize to protein aggregates, as outlined in point 3(d). We will carefully analyze these new data and discuss their interpretation as suggested by the reviewer.

3. Figure 2G: Similar to before, this experiment would improve if the authors could find a way to normalise samples between conditions prior to sucrose gradient fractionation or have the most relevant samples on the same blot. It is challenging to properly interpret the results while the

bands in the total cell lysate (TCL) lane do not have similar intensities between samples. A blot in which only the TCL and the pellet samples of all conditions were loaded onto the same gel would solve this, allowing for a better comparison between conditions.
Based on what is shown in panel G, the authors should amend their claim: 'Consistent with microscopic observations, denser fractions from Hoil-1C458A/C458A MEFs contained increased signals of p62 specifically during the recovery phase (Figure 2G).' It is not apparent that more p62 is present in the insoluble fractions of mutant HOIL-1 cells after puromycin treatment. The band intensities look very similar (This is different for the recovery condition, which shows a strong difference, as stated).

We agree with the reviewer's interpretation of the current data. Based on the bands shown in panel G, it is not appropriate to conclude that p62 is increased in the insoluble fractions of *Hoil-1*$^{C458A/C458A}$ MEFs immediately after puromycin treatment, as the signal intensities appear comparable between genotypes. We will therefore amend the text accordingly and restrict this conclusion to the recovery condition, where a clear difference is observed. The revised manuscript will reflect this more precise and accurate interpretation..

4. Figure S2: Similar to before, the authors induce protein aggregate formation and compare cells endogenously expressing WT vs mutant HOIL-1L. The size of aggregates increases in mutant cells under proteotoxic stress. What happens to the number of aggregates per cell in these conditions? Does it also increase, or is it just the size?

Thank you for raising this point regarding aggregate number. We analyzed this parameter and found that the number of aggregates is comparable between conditions, whereas their size differs, as shown in Figure S2. Because this observation relates directly to issues raised for the main Figure 2, we will include these data in the revised manuscript and discuss their implications accordingly.

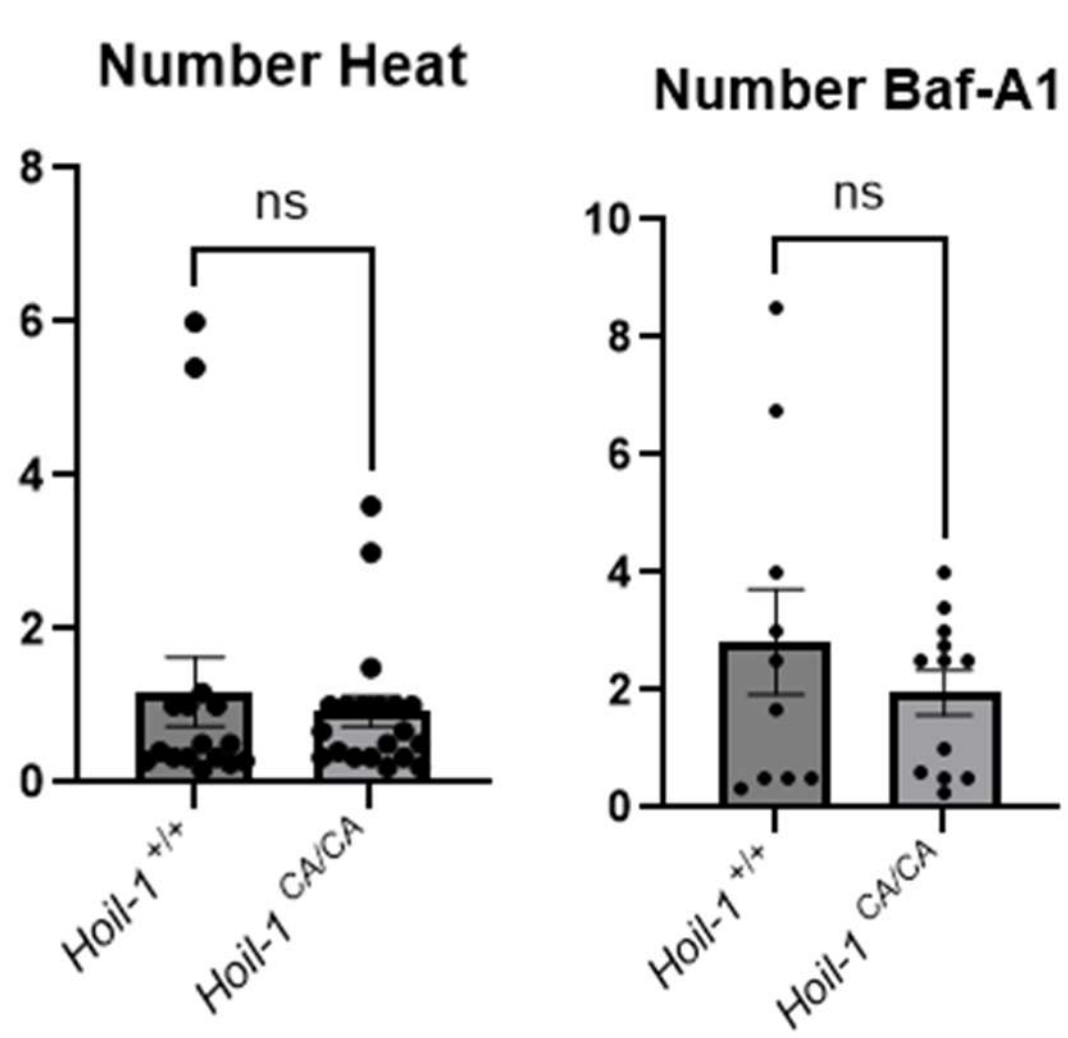

Analysis of the number of aggregates

5. Fig. S4A: Here the authors analyse the circularity of p62 aggregates in HOIL-1L mutant cells after recovery from puromycin treatment. This experiment would improve if the same analysis

could be performed for the WT cells and for the pre-recovery condition (under the condition that large enough granules are present), allowing to make a comparison between WT and mutant, as well as between pre- and post-recovery.

We will analyze data as suggested and will include in the revised manuscript.

6. Fig. 4H-J: The authors use a p62 mutant that is known for its enhanced ubiquitin affinity, repeat Fig. 4E-G and state: 'These aberrant condensates were similar to those observed in a reaction using wild-type p62.' Can the authors please comment on what they conclude from this similarity and why this experiment was performed? A quantitative comparison (condensate size and circularity) between WT p62 and mutant p62 may further be useful here.

Indeed, we did not initially include the necessary direct comparison between p62 WT–based and p62 SE mutant–based datasets to fully support this statement. Upon careful reanalysis, we identified an interesting and previously unappreciated observation: in reactions containing the HOIL-1 catalytic mutant, condensates formed with p62 show significantly reduced circularity. We will therefore include these comparative datasets in the revised manuscript and discuss their implications in the text. We thank the reviewer again for highlighting this important point.

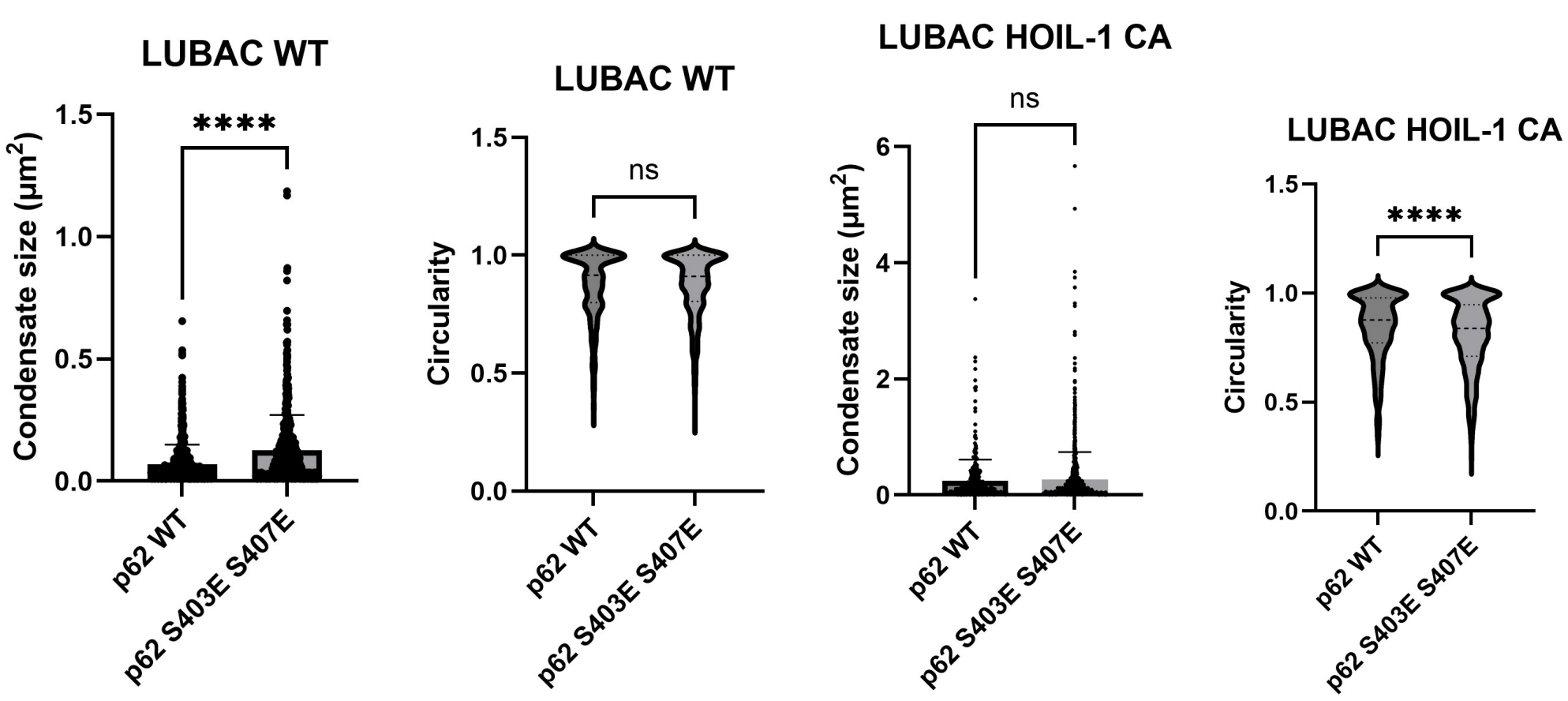

7. Fig. 5A: Here the authors pulldown M1-linked Ub-chains in WT vs mutant HOIL-1 cells, with or without puromycin-induced aggregate formation. More M1-chains are observed in mutant HOIL-1 cells under puromycin treatment, but the difference is very subtle. The conclusions drawn from this experiment could be strengthened by including alternative methods, for example (if available) Ub-AQUA to measure the abundance of M1-chains, or using the M1-antibody for IF analysis.

We fully agree with the reviewer that the immunoblot-based analyses reveal only very subtle differences, which may reflect the loss of spatial information when analyzing total cell lysates. As suggested, we will therefore perform IF/ICC-based analyses to better capture the localization and enrichment of Met1-linked ubiquitin chains and strengthen the overall conclusions.

8. Fig. S5B: The described differences between puromycin treatment and untreated conditions are extremely subtle on the anti-Ub blot, and absent in the anti-Met1 blot. I recommend that the authors remove this sentence, based on the shown data: 'we observed a modest increase in the signal of Met1-linked ubiquitin chains after puromycin treatment'.

We agree with the reviewer and will remove the sentence as suggested.

9. Fig. 5F-G: The authors went back to their more-artificial system from Fig 1, in which HOIL-1L was transiently overexpressed (WT or inactive mutant) in SH-SY5Y cells, alongside alpha-synuclein aggregate formation.

a. The claims made from this experiment would be stronger in the other cell system. Could OTULIN be transiently overexpressed in the MEF cells, to monitor the effect of aggregate formation and clearance. Again, staining aggregates with the M1-antibody would improve this experiment.

We thank the reviewer for this helpful suggestion and will carry out the experiments as outlined below. The plasmids required for these experiments are currently under construction, and we are finalizing them to proceed with the proposed analyses.

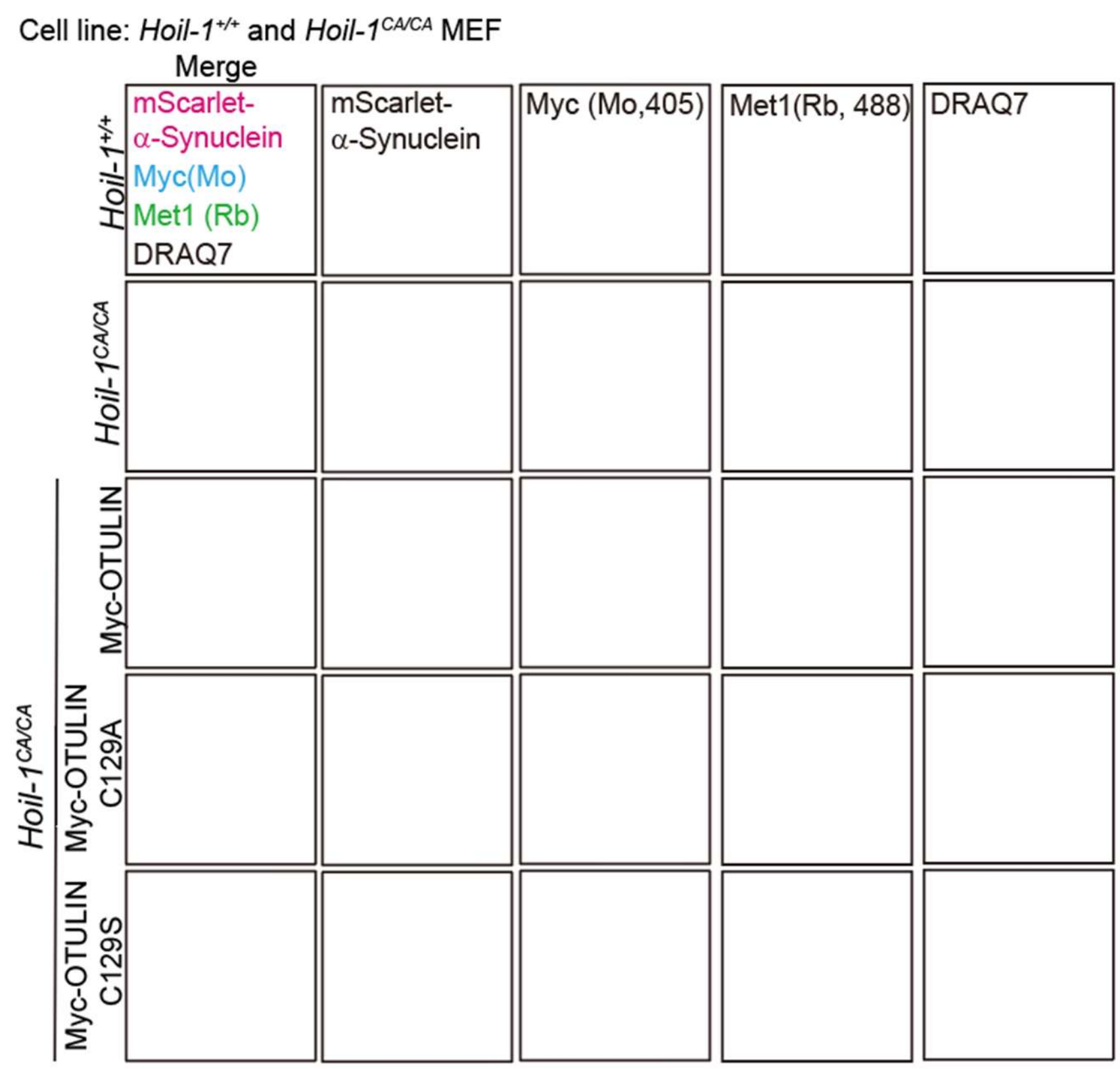

b. The authors claim that HOIL-1 activity fine-tunes the function of HOIP within LUBAC (from discussion: 'This regulatory mechanism ensures that in the presence of functional HOIL-1, the overall quantity and potentially the architecture of Met1-linked ubiquitin chains are tightly controlled'). What is the quantity and architecture of M1-chains catalysed by LUBAC when HOIL-1 is very highly abundant, as it would be the case in this cellular overexpression system?

This is a challenging issue, and we agree that there is no straightforward experimental approach to fully resolve it at present. Importantly, our observations depend on the coordinated activities of both HOIL-1 and HOIP in generating Met1-linked ubiquitin chains. We propose that excess HOIL-1 relative to HOIP may result in orphan HOIL-1 molecules that do not contribute to Met1-chain formation and therefore do not alter overall Met1-ubiquitin levels. Together with data derived from non-overexpression MEFs (Figures 2–5), we believe our findings collectively support our conclusions. Nevertheless, we acknowledge the limitations and aim to address this question in greater depth in a future, dedicated study.

10. Sentence structure: 'A comprehensive understanding of how Met1-linked ubiquitination, particularly through intricate regulation by Linear Ubiquitin Chain Assembly Complex (LUBAC) components, such as HOIL-1, influences aggregate dynamics and clearance; therefore, it is crucial to develop targeted therapeutic strategies against neurodegenerative proteinopathies'.

Thank you for pointing this out. We agree that the sentence structure is unclear. We will revise it for clarity and readability, as follows:
“A comprehensive understanding of how Met1-linked ubiquitination—particularly through its intricate regulation by Linear Ubiquitin Chain Assembly Complex (LUBAC) components such as HOIL-1—influences aggregate dynamics and clearance is crucial for the development of targeted therapeutic strategies against neurodegenerative proteinopathies.”
This revised wording will be incorporated into the manuscript.

Reviewer #3 (Significance (Required)):

Significance
The conclusions drawn from this study are very intriguing and give LUBAC (and HOIL-1) a so far unrecognised role in the clearance of protein aggregates, which are a hallmark of several neurodegenerative diseases for which there are currently no cures. Some of the findings described in this manuscript have the potential to be of very high impact and interest to a broader community, in particular researchers interested in protein homeostasis, autophagy, ubiquitin biology and neurodegeneration. In fact, those findings might even expand to autophagic pathways that target other cargo than protein aggregates. Both the novelty aspect and the potential for translational/therapeutic applications comprise the major strength of this manuscript. However, multiple of the presented experiments are currently lacking crucial controls, show weak effect sizes or were performed in artificial settings that likely do not represent relevant in vivo conditions, overall weakening or not fully supporting the claims made. Consequently, further experiments, data re-analyses and validations were recommended to fully support all the claims made here.
This review was written from the perspective of a researcher in the ubiquitin field.

3. Description of the revisions that have already been incorporated in the transferred manuscript

Please insert a point-by-point reply describing the revisions that were already carried out and included in the transferred manuscript. If no revisions have been carried out yet, please leave this section empty.

4. Description of analyses that authors prefer not to carry out

Please include a point-by-point response explaining why some of the requested data or additional analyses might not be necessary or cannot be provided within the scope of a revision. This can be due to time or resource limitations or in case of disagreement about the necessity of such additional data given the scope of the study. Please leave empty if not applicable.

The comments below raise thoughtful points regarding experimental interpretation and the boundaries of the current approaches. We have carefully considered these issues and address them through clarification and discussion. However, we believe that additional experimental analyses would be technically challenging or beyond the intended scope of this revision, and unlikely to substantially alter the overall conclusions.

Rev #3 point6
b. Can the authors please clarify whether the reaction was inactivated prior to addition to the aggregate-formation assay? If not, the enzymes might still be active at the point of aggregate formation, and the observed effects might be influenced by enzymatic activities and not only the presence of different M1-chain architectures.

This is an interesting point. In our *in vitro* ubiquitination assays, samples are snap-frozen after incubation, indicating that residual enzymatic activity may persist in both wild-type LUBAC and LUBAC containing the HOIL-1 mutant. Fully inactivating LUBAC under these conditions is technically challenging, as additional inactivation steps lead to ubiquitin chain aggregation. Nevertheless, because clear differences are consistently observed between samples in which HOIP remains active in both conditions, we interpret the observed effects as specifically attributable to HOIL-1 ligase activity. We will make this rationale explicit in the revised manuscript.

Rev #3 Minor point #9
b. The authors claim that HOIL-1 activity fine-tunes the function of HOIP within LUBAC (from discussion: 'This regulatory mechanism ensures that in the presence of functional HOIL-1, the overall quantity and potentially the architecture of Met1-linked ubiquitin chains are tightly controlled'). What is the quantity and architecture of M1-chains catalysed by LUBAC when

HOIL-1 is very highly abundant, as it would be the case in this cellular overexpression system?

This is a challenging issue, and we agree that there is no straightforward experimental approach to fully resolve it at present. Importantly, our observations depend on the coordinated activities of both HOIL-1 and HOIP in generating Met1-linked ubiquitin chains. We propose that excess HOIL-1 relative to HOIP may result in orphan HOIL-1 molecules that do not contribute to Met1-chain formation and therefore do not alter overall Met1-ubiquitin levels. Together with data derived from non-overexpression MEFs (Figures 2–5), we believe our findings collectively support our conclusions. Nevertheless, we acknowledge the limitations and aim to address this question in greater depth in a future, dedicated study.

Prof. Fumiyo Ikeda
The University of Osaka
Graduate School of Frontier Biosciences
Osaka
Japan

23rd Apr 2026

Re: EMBOJ-2026-124365-T
Excess Met1-ubiquitination leads to solid aggregate formation

Dear Fumiyo,

Thank you for transferring your manuscript on aggregate formation upon excess linear ubiquitination from Review Commons to The EMBO Journal. I have now had the chance to read your study, as well as to go through the referee reports and your responses to them. I appreciate the principle importance of the question as well as the potential interest highlighted by three expert referees, and I also realize that your plans for addressing their constructive comments appear reasonable. We would therefore be happy to pursue a revised manuscript further for EMBO Journal publication, assuming that the planned experiments will provide satisfactory answers to the reviewers' key queries. In this light, I am herewith inviting you to prepare and submit a new version revised along the lines suggested in your response letter.

When preparing a revised manuscript, please try to adhere to the guidelines listed below and in our Guide to Authors as closely as possible, as this should greatly facilitate our assessment at the time of resubmission - in particular regarding the completion of our author checklist, the inclusion of editable text files and individual figures files, and the conversion of "supplemental" material into Expanded View and/or Appendix content. Please also note that it is our policy to allow only a single round of (major) revision, making it important to comprehensively answer all criticisms at this point - if this should require more time than our standard three-months deadline, I would be happy to discuss an extension of the revision time, during which our 'scooping protection' (meaning that competing work appearing elsewhere in the meantime will not affect our considerations of your study) would of course remain valid. Please do not hesitate to contact me should you have any further questions at this stage.

Thank you again for the opportunity to consider this study for The EMBO Journal. I look forward to receiving your revision.

With kind regards,

Hartmut

Hartmut Vodermaier, PhD
Senior Editor, The EMBO Journal
h.vodermaier@embojournal.org

Instructions for preparing your revised manuscript:

Read our guidance for manuscript revisions and related editorial policies: https://link.springer.com/journal/44318/submission-guidelines#cms-Revised-submissions

Please make sure you upload a letter of response to the referees' comments together with the revised manuscript.

Please also check that the title and abstract of the manuscript are brief, yet explicit, even to non-specialists.

When assembling figures, please refer to our figure preparation guideline in order to ensure proper formatting and readability in print as well as on screen:
https://media.springernature.com/original/springer-cms/rest/v1/content/27825798/data/v1

At EMBO Press we ask authors to provide source data for the main manuscript figures. You will receive a separate email with instructions for providing source data with your revised manuscript, including how to upload and organize the files.

IMPORTANT: When you send the revision we will require

- a point-by-point response to the referees' comments, with a detailed description of the changes made (as a word file).
- a word file of the manuscript text.
- individual production quality figure files (one file per figure)
- a complete author checklist
- Expanded View files (replacing Supplementary Information)
- a Reagents and Tools Table as part of the Methods section

Please remember: Digital image enhancement is acceptable practice, as long as it accurately represents the original data and conforms to community standards. If a figure has been subjected to significant electronic manipulation, this must be noted in the figure legend or in the 'Materials and Methods' section. The editors reserve the right to request original versions of figures and the original images that were used to assemble the figure.

We realize that it is difficult to revise to a specific deadline. In the interest of protecting the conceptual advance provided by the work, we recommend a revision within 3 months (22nd Jul 2026). Please discuss the revision progress ahead of this time with the editor if you require more time to complete the revisions. Use the link below to submit your revision:

Link Not Available

***

Rev_Com_number: RC-2026-03404
New_manu_number: EMBOJ-2026-124365-T
Corr_author: Ikeda
Title: Excess Met1-ubiquitination leads to solid aggregate formation

Manuscript number: EMBOJ-2026-124365-T
Corresponding author(s): Fumiyo, Ikeda

We sincerely thank the reviewers for their thorough, constructive, and insightful evaluations of our manuscript. We are encouraged that all three reviewers recognized the novelty and significance of our central finding that HOIL-1 catalytic activity fine-tunes LUBAC-dependent Met1-linked ubiquitination to regulate p62-positive condensate material properties and promote late-stage aggrephagy.

The reviewers identified several areas where additional controls, quantification, mechanistic support, and clarification would strengthen the study. In response, we performed the additional experiments outlined in our revision plan, incorporated new analyses and controls, expanded discussion of relevant literature, and revised the text throughout to ensure that our conclusions are precise and fully supported by the data.

Detailed point-by-point responses to each reviewer comment are provided below.

Reviewer #1
Evidence, reproducibility and clarity

Background
GWAS analyses carried out several years ago identified over 40 genetic loci associated with increased risk of developing Alzheimers and other types of dementia. They included loci encoding the E3 ubiquitin ligase HOIL-1 and the protein SHARPIN, two of the three components of the Linear Ubiquitin Assembly Complex (LUBAC) (Bellenguez et al, 2022, cited in the paper). The third component of LUBAC, HOIP, is the only E3 ubiquitin ligase known to catalyse the formation of Met1-linked ubiquitin (also known as linear ubiquitin). HOIL-1 is one of the few E3 ubiquitin ligases that attaches ubiquitin to serine and threonine residues in proteins forming ester bonds (Kelsall et al. 2019) and has been reported to restrict the HOIP-catalysed formation of Met1-linked ubiquitin (Kelsall et al. 2019; Fuseya et al. 2020; Rodriguez Carvajal et al. 2021).

Summary of Paper's findings:
In this study the authors report that HOIL-1 catalytic activity prevents neurodegenerative protein aggregation (synuclein, tau, Aβ) in the human SH-SY5Y neuroblastoma cell line or in mouse embryonic fibroblasts (MEFs) expressing a catalytically inactive mutant of HOIL-1. They argue that this is achieved by maintaining the dynamic, liquid-like properties of protein condensates through regulation of Met1-linked ubiquitin chain levels, thereby facilitating efficient clearance via the aggrephagy pathway. They report that loss of HOIL-1 activity leads to excess Met1-ubiquitylation that drives the transition to rigid, solid-like aggregates resistant to autophagic degradation. In support of this conclusion, they also report that the siRNA knock-down of Otulin, a deubiquitylase that hydrolyses Met1-linked ubiquitin specifically, produces the same effect . The reframing of HOIL-1 as a key factor for fine-tuning ubiquitylation to maintain cellular protein homeostasis is an interesting development and the paper is generally well-written, focused and

concise. Further work is required however, to fully convince these reviewers that the effects observed are entirely attributable to excess Met1-linked ubiquitylation, as claimed.

We thank the reviewer for the careful assessment and positive evaluation of our study. We appreciate the recognition of the conceptual advance in reframing HOIL-1 as a regulator of protein homeostasis, as well as the clarity, focus, and concision of our presentation. Please find detailed responses as below.

Major comments:

1. The causal link between elevated Met1-linked chains and solid-like aggregates in cells is the central claim of the paper. Throughout the study the authors use inactive HOIL-1 to enhance aggregate formation, which they attribute to increased Met1-linked ubiquitylation, something observed by themselves and others previously (Kelsall et al. 2019; Fuseya et al. 2020; Rodriguez Carvajal et al. 2021). However, the immunoblot for Met1-linked ubiquitin (Fig 5A) is not very convincing.

We thank the reviewer for raising this important point. We repeated the experiments using larger amounts of starting material to improve the detection of Met1-linked ubiquitin by immunoblotting. As anticipated, a biochemical approach with repeated experiments did not address this issue clearly; because Met1-linked chains were expected to be enriched at specific cellular sites such as condensates and aggregates, total lysates may not optimally reflect their abundance. Therefore, we additionally assessed Met1-linked ubiquitin using immunocytochemistry with a chain-specific antibody, which are shown in new figures, Fig 2H, Fig 5F, and Fig EV5H. These new data supported that aggregates indeed include Met 1-linked ubiquitin chains.

In addition, the authors have not excluded the possibility that the loss of HOIL-1 enzyme activity has other effects on ubiquitylation, such as a change in the architecture of the ubiquitin chains caused by the absence of HOIL-1 catalysed formation of oxyester linkages.

We agree with the reviewer that HOIL-1-mediated oxyester-linked ubiquitination represents an important and interesting aspect of LUBAC biology. Indeed, HOIL-1 has been shown to generate hybrid Met1/oxyester ubiquitin chains, as demonstrated in our previous study (Rodriguez Carvajal et al., 2021) and by others (Kelsall et al., 2019; Fuseya et al., 2020). To begin addressing the potential contribution of this specific ubiquitin architecture, we took advantage of the deubiquitinase Cezanne, which we previously showed can hydrolyze oxyester linkages within Met1-linked chains (Rodriguez Carvajal et al., 2021). We therefore performed ICC analyses in neuroblastoma cells expressing either wild-type Cezanne or its catalytic mutant. Under the conditions tested, neither construct produced a clear effect on α-synuclein aggregate formation (new data shown below).

We acknowledge that this approach has limitations and that, at present, no specific experimental tool is available to selectively manipulate oxyester-linked ubiquitination in cells and thereby directly assess its functional contribution. Consequently, we feel that a definitive evaluation of the role of oxyester linkages falls beyond the scope of the present study and will require future investigation.

Importantly, our key conclusions do not rely on the exclusion of a contribution from oxyester linkages. Rather, they are supported by multiple independent lines of evidence, including the phenocopy produced by OTULIN depletion and our new ICC analyses demonstrating the presence of Met1-linked ubiquitin chains within p62-positive aggregates. Together, these findings continue to support our central conclusion that dysregulated Met1-linked ubiquitination contributes to altered aggregate properties and impaired clearance.

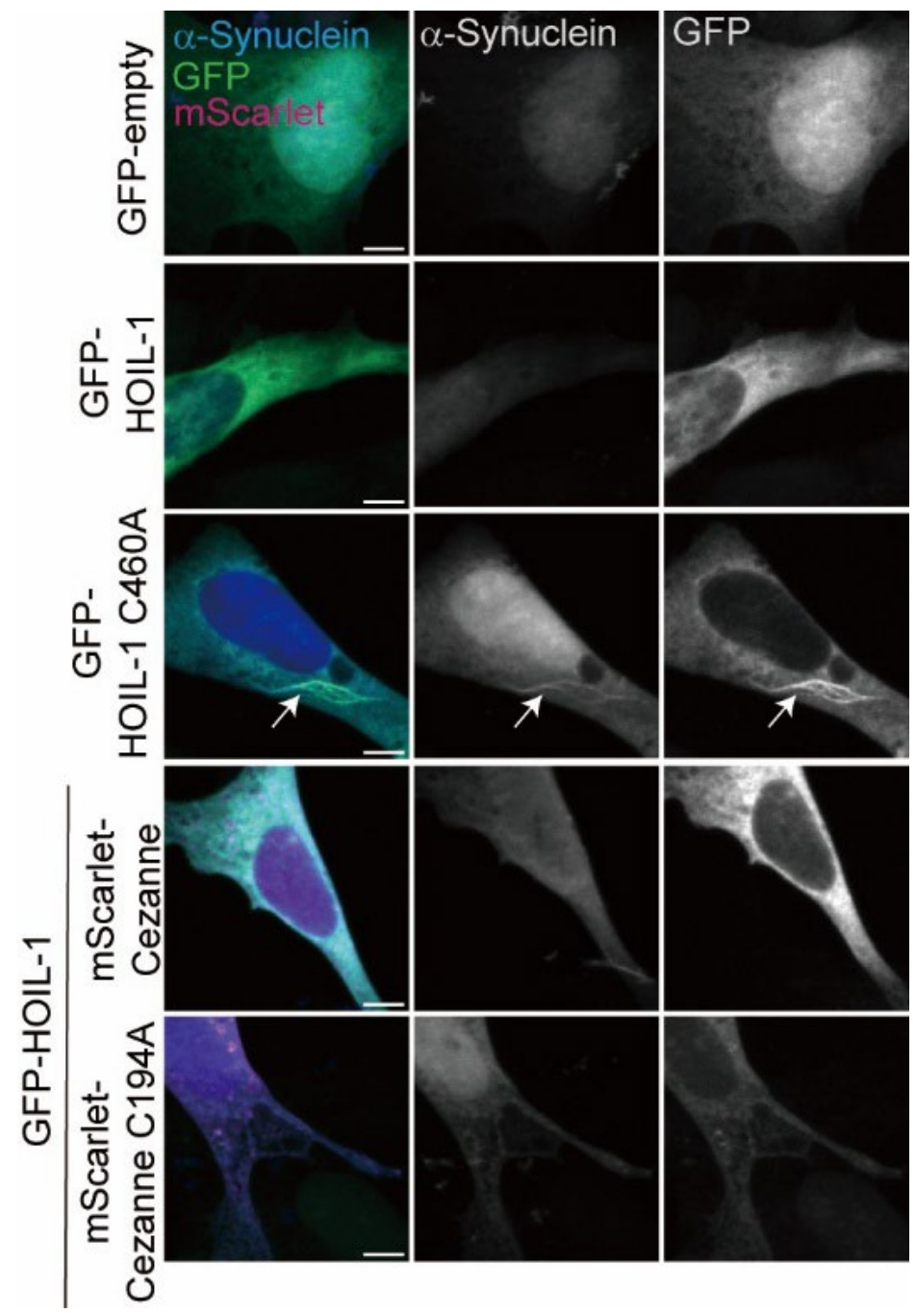

Cezanne-based ICC analysis. Representative immunocytochemistry (ICC) images showing α-synuclein aggregates in SH-SY5Y cells expressing GFP alone, GFP-HOIL-1 WT, GFP-HOIL-1 C460A, GFP-HOIL-1 WT plus mScarlet-I3-Cezanne, or GFP-HOIL-1 WT plus the catalytically inactive mScarlet-I3-Cezanne C194A mutant, used to assess the potential contribution of HOIL-1-dependent oxyester-linked ubiquitination.

Many/most ubiquitin chains formed in cells contain more than one ubiquitin linkage type. It is therefore important for the authors to perform immunoblots for other ubiquitin linkage types, such as Lys63-linked ubiquitin, and to include these results in Fig 5.

To assess whether Lys63-linked ubiquitin chains are involved, we analyzed the UBAN- or TUBE-enriched fractions for the presence of this linkage type. In this condition, we have not detected appreciable differences between the conditions examined in Fig 5. Our new data supporting this conclusion are shown in Fig EV5A and B.

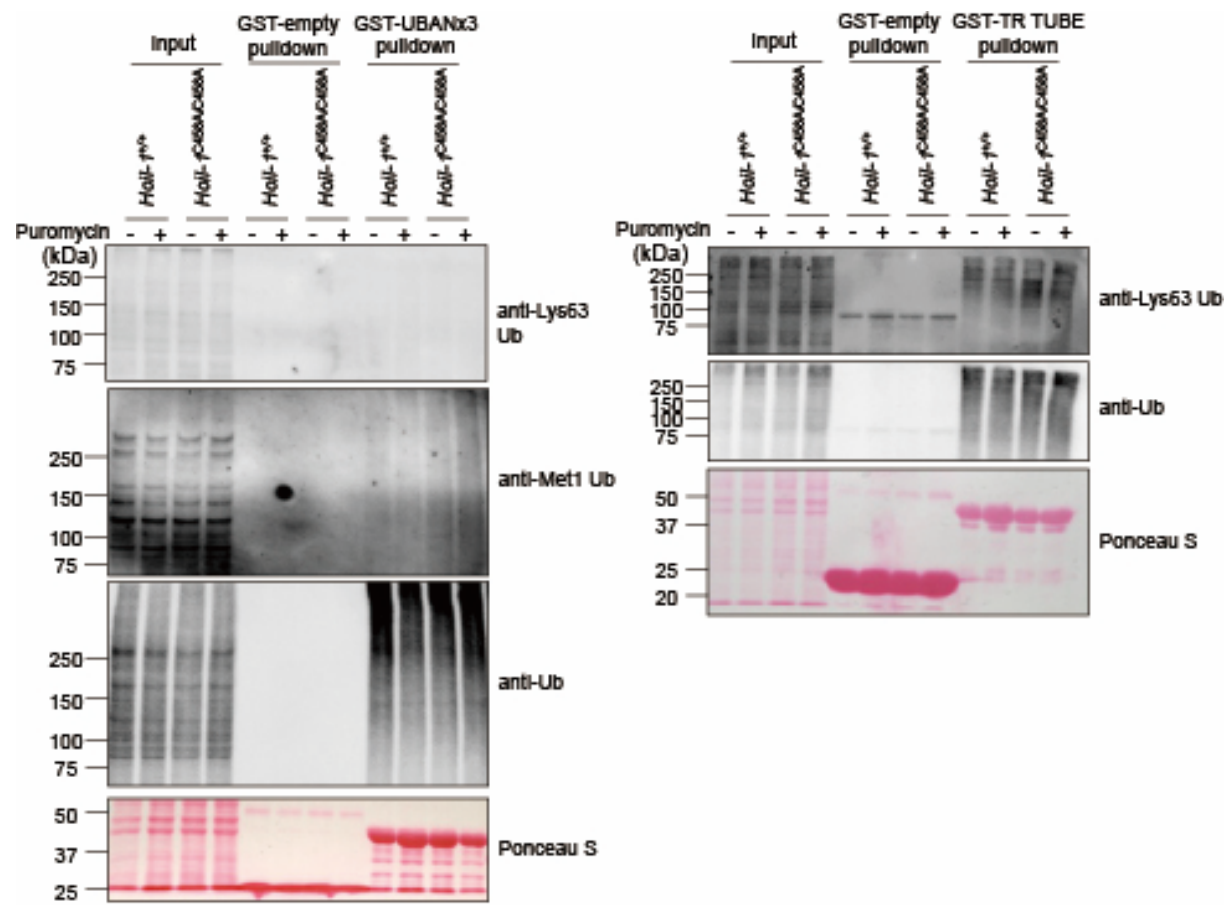

2. The reviewers also think that the authors' claims that the transition of condensate property is linked to elevated Met1-linked ubiquitin chains would be strengthened by performing the biophysical assays (FRAP and 1,6-hexanediol resistance) after Otulin knockdown/knockout (*and ideally also with Otulin rescue*). This will provide direct biophysical evidence linking Met-1 linked chain elevation to condensate liquidity and 1,6-HD sensitivity.

We thank the reviewer for this constructive suggestion. As requested, we performed both FRAP and 1,6-hexanediol sensitivity assays in OTULIN-knockdown cells. Importantly, the new FRAP analyses revealed that p62-positive condensates in sh*Otulin* cells exhibit reduced mobility compared with controls (new Fig. 5G), providing independent support for our central conclusion that elevated Met1-linked ubiquitination promotes the transition toward less dynamic condensates.

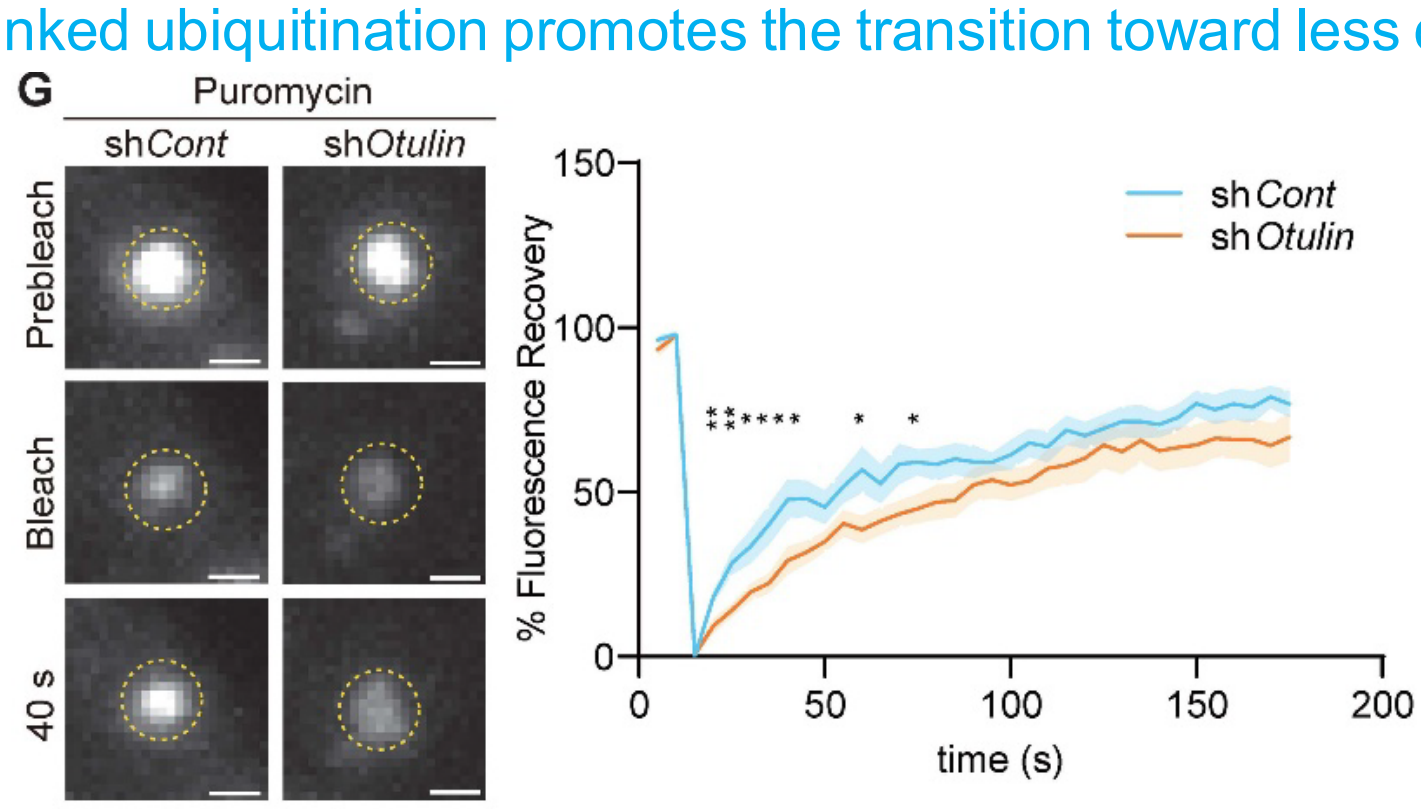

Interestingly, the 1,6-hexanediol assays in sh*Otulin* MEFs did not reveal a clear difference in the size of p62⁺/ubiquitin⁺ puncta compared with control cells. This suggests that partial OTULIN depletion may not be sufficient to generate the level or spatial enrichment of Met1-linked ubiquitin chains required to drive the formation of larger and more solid-like p62 bodies in this system. Consistent with this interpretation, the effects observed in the FRAP analyses of sh*Otulin* cells were more modest than those observed in Hoil-1 catalytic mutant MEFs (Fig. 4C), where Met1-linked ubiquitination is expected to be more profoundly dysregulated.

Nevertheless, the reduced condensate mobility observed following OTULIN depletion provides an independent line of evidence linking increased Met1-linked ubiquitination to altered condensate material properties and therefore further supports the central conclusions of our study.

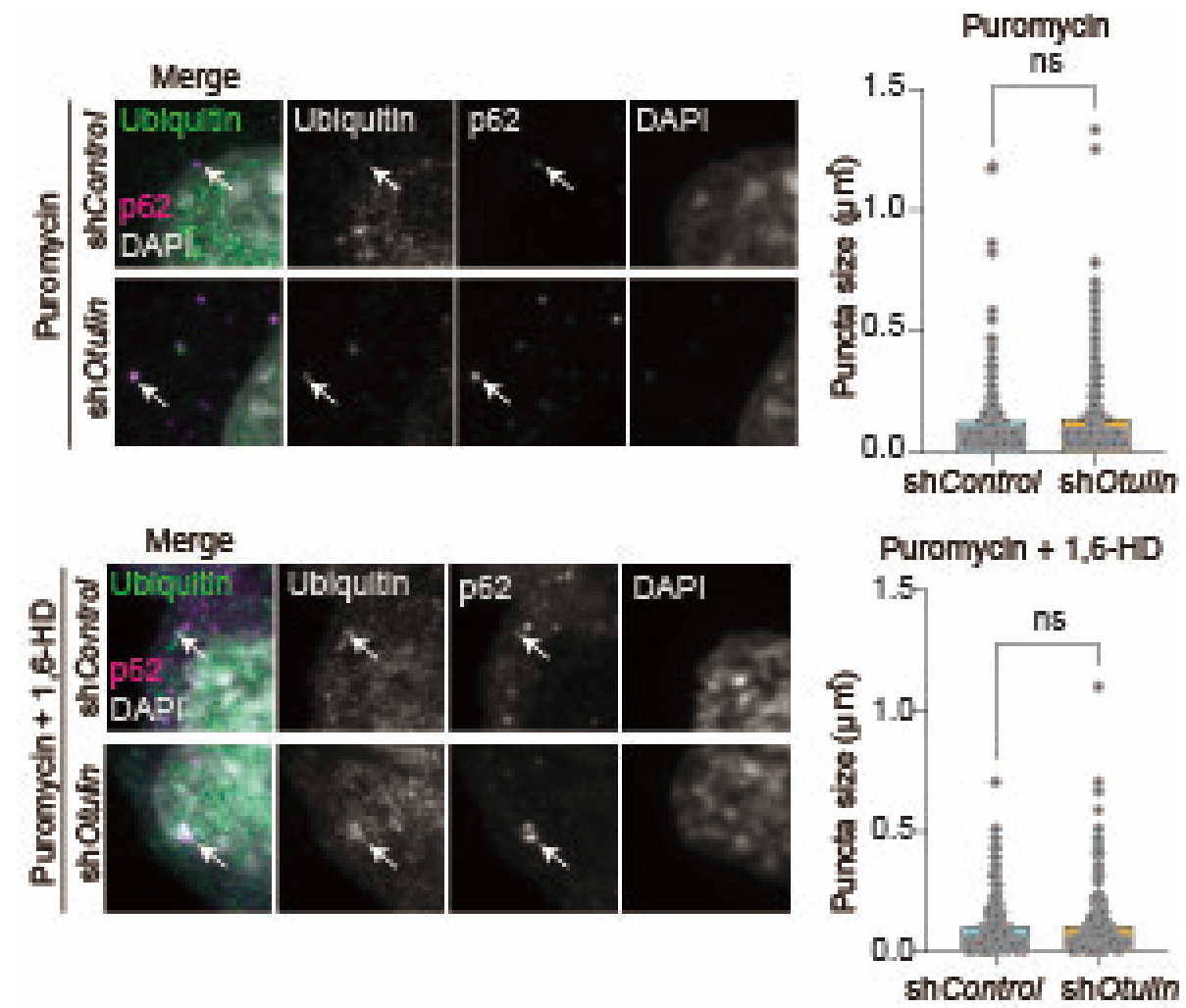

Representative images of immunofluorescence under puromycin treatment and puromycin+ 5% 1,6-Hexanediol treatment in sh*Control* and sh*Otulin* MEFs.

3. The authors have not shown any evidence that Met1-linked chains are more enriched at the sites of protein aggregation. Would the authors be able to demonstrate direct spatial colocalization of Met1-Ub with the analysed aggregates?

We appreciate this important suggestion. We first validated a Met1-linked ubiquitin–specific antibody in sh*Control* and sh*Otulin* MEFs, with and without the E1 inhibitor TAK-243, confirming a specific signal only in sh*Otulin* cells in the absence of E1 inhibition (Fig EV2E). Using this validated antibody, we performed the proposed analyses of Met1-linked ubiquitin localization. Our new data showed that Met1-linked ubiquitin chains are colocalized with the p62-positive aggregates (Fig 2H, Fig 5F, and Fig EV5H) supporting our main message.

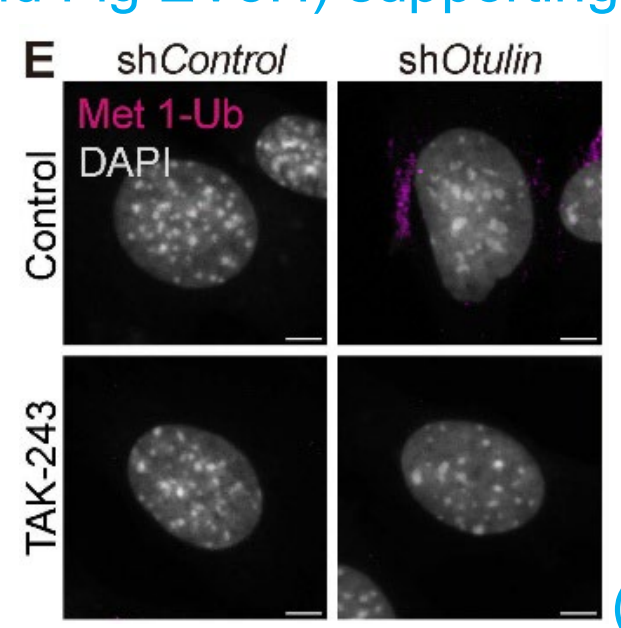

(Fig EV2E)

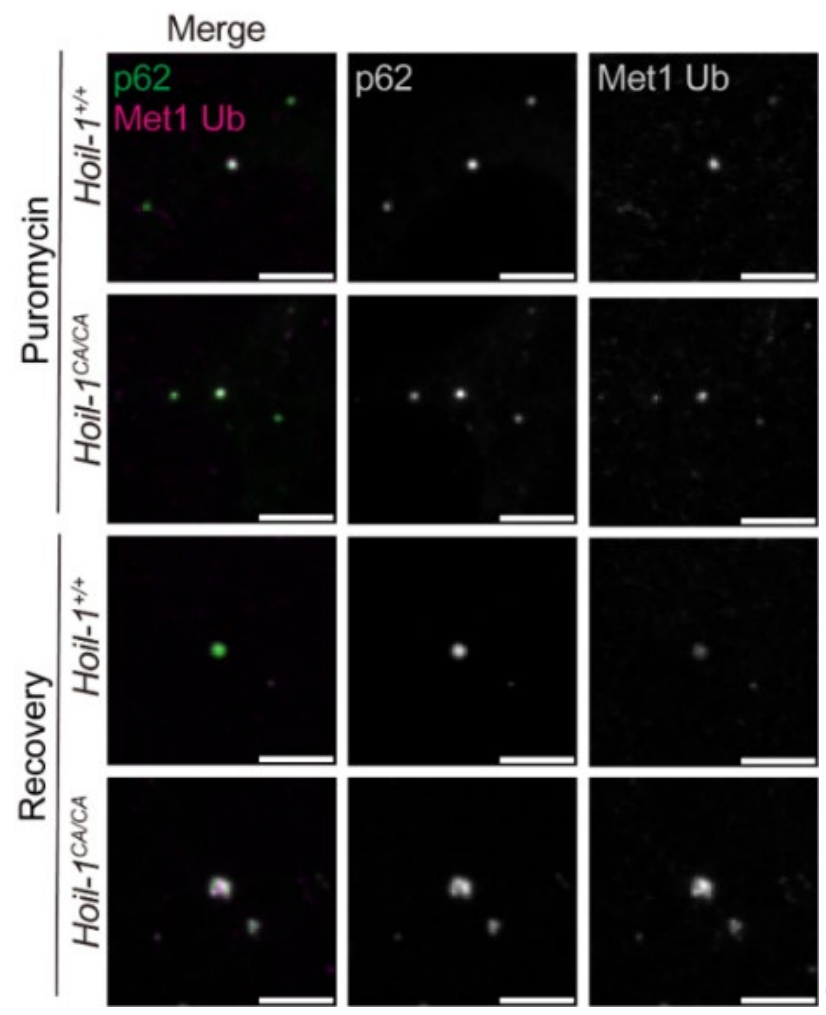

(Fig 2H)

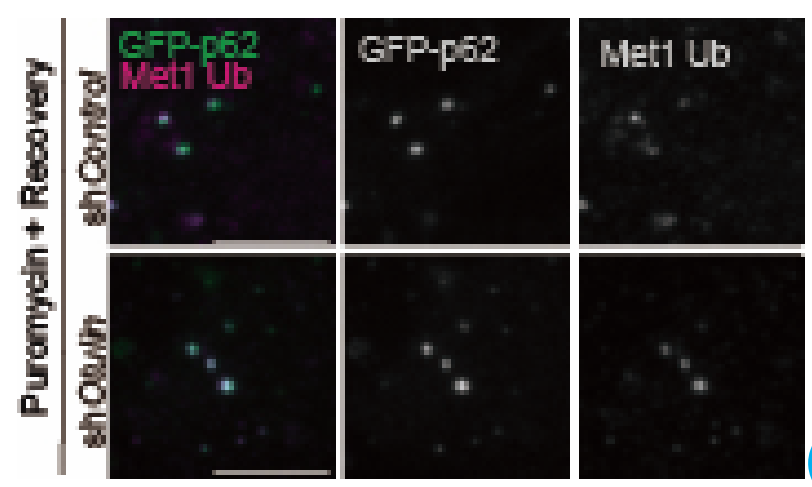

(Fig 5F)

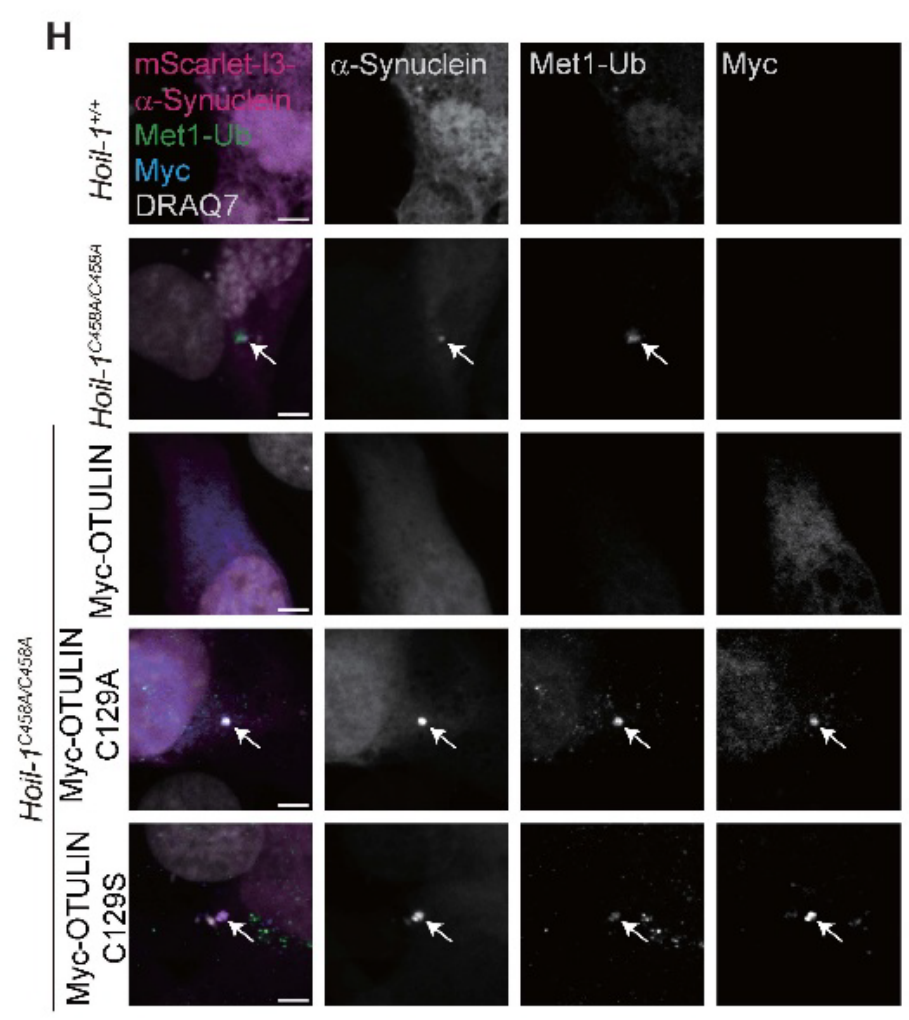

(Fig EV5H)

4. Do the authors know if the effects that they are seeing are general effects on autophagy? For example, is starvation-induced autophagy similarly impaired in the cells studied? A simple flux-style experiment looking at LC3-II levels and p62 with starvations vs puromycin (-/+ bafilomycin) would be informative here.

We examined autophagic flux under the suggested conditions. Our new data indicate that amino acid starvation-induced autophagy is largely comparable between wild-type and HOIL-1 mutant cells (new Fig 3J). Interestingly, puromycin-dependent induction of p62 was not clearly observed by immunoblotting in a conditions we tested, suggesting that "total" amount of p62 is not largely altered (new Fig EV3D).

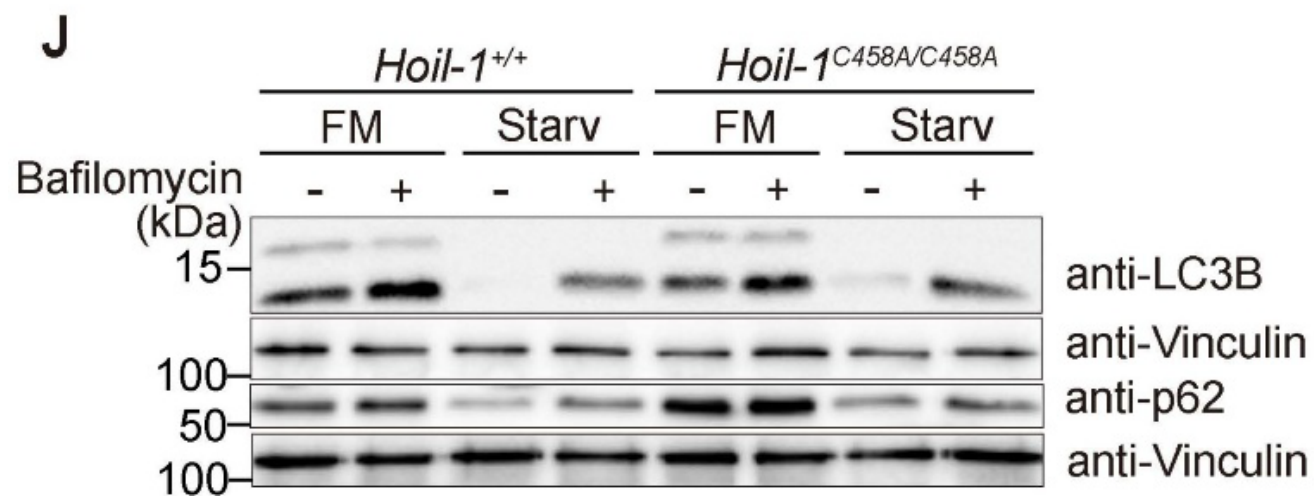

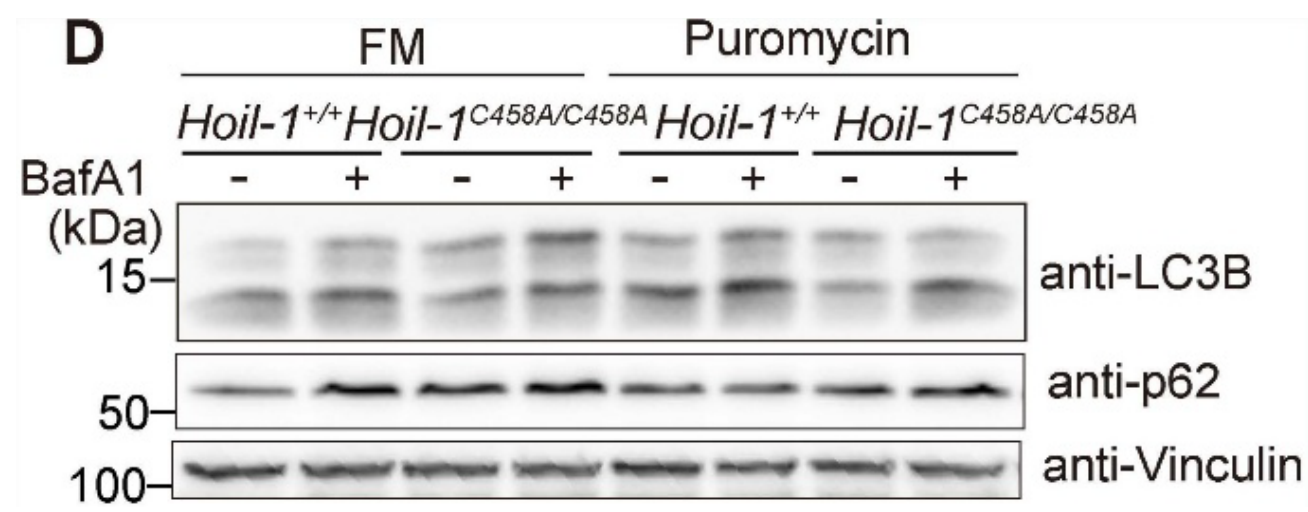

Minor comments:

5. The authors show that loss of HOIL-1 catalytic activity causes p62 bodies to transition from dynamic liquid-like states to rigid solid-like states and claim this as a more general effect on protein aggregates. But the study does not directly demonstrate a liquid-to-solid transition for the disease-relevant α-synuclein, tau, or Aβ aggregates, limiting the generalisation of the claim beyond p62 bodies. Perhaps the authors should modify the text to better reflect this (or, even better, consider treating α-synuclein/tau/Aβ aggregates with 1,6-hexanediol to measure the response). [optional]

We revised the manuscript to describe these observations more precisely and clearly, ensuring that the interpretation accurately reflects the data, as suggested (in the abstract, in the discussions p12).

Abstract: "In parallel, <u>p62 bodies</u> undergo a biophysical transition from dynamic, liquid-like condensates to rigid, solid-like structures."
P12: "Consistent with these cellular observations, our in vitro LLPS assay suggests that excessive Met1-linked chains may promote the formation of rigid, solid-like <u>p62-positive</u> aggregates, a phenomenon directly supported by our cellular biophysical analyses (e.g., 1,6-HD sensitivity and FRAP)."

6. Given that the blots presented in Fig S1A appear to come from different membranes, and high-molecular-weight species of α-synuclein seem to exist in the insoluble pellet fraction of both WT and C460A expressing cells, the reviewers would caution against concluding anything about differences, which can only be assessed if the samples are run side-by-side on the same gel.

We agree with the reviewer that this dataset is not the most appropriate for addressing the point raised. Accordingly, we have decided to omit these data from the revised manuscript to avoid overinterpretation and to maintain analytical rigor.

7. The Methods section says that two different total ubiquitin antibodies were purchased, but which one was used in Figure 5 and other figures are not stated. Please clarify.

We clarified the antibodies used as suggested in the revised manuscript (in the Method section, page 19 and 23).

8. On page 10 ABIN1 is mentioned but it is not mentioned that it is the protein product of the TNIP1 gene that is mentioned in the Introduction. This will confuse to many readers.

Thank you for pointing this out. We agree that this could be confusing for readers. We clarified in the revised manuscript that ABIN1 is the protein product of the *TNIP1* gene when it is first mentioned (in Introduction, p3), ensuring consistency and improving clarity between the Introduction and subsequent sections.

9. 1st paragraph of Discussion line 5 from bottom:- change "oof" to "of".

Thank you for pointing out this typographical error. We have corrected “oof” to “of” in the revised manuscript.

Significance

Prior research has established that the components of LUBAC are recruited to, and are components of, protein aggregates. A link between LUBAC and selective autophagy has also been established previously. The significance of this paper is that it identifies the catalytic function of HOIL-1 as a brake on the activity of LUBAC in proteostasis. The reviewer and co-reviewer are not experts in autophagy or aggregate formation in dementia but, if those reviewers who are find the data presented in these areas to be convincing, then this paper may be the first to suggest a molecular mechanism by which polymorphism/mutation of HOIL-1 leads to increased formation of the aggregates observed in Alzheimer's and other dementias. The results presented in the paper also suggested that initial autophagosome recruitment to aggregates is intact but subsequent late-stage autophagy is impaired. Hence, the study begins to identify the specific step that fails. However, as the authors themselves acknowledge, validation of these potentially exciting findings using in vivo models of neurodegeneration should be the aim of future studies.

The paper combines the molecular dissection of ubiquitin and autophagy pathways to understand the causes of neurodegenerative disease. The paper will therefore be of interest to a broad audience, encompassing both the basic research and clinical research communities.

Reviewers field of expertise: Biochemists and cell biologists with an interest in ubiquitin and cell signalling.

Reviewer #2

Evidence, reproducibility and clarity

Summary:

In this manuscript, the authors show that the branching activity of the E3 ligase HOIL-1, a component of the LUBAC complex, contributes to the autophagic clearance of p62 bodies and protein aggregates. This activity is attributed to enhanced linear, unbranched ubiquitin chain formation by the second E3 ligase of LUBAC, called HOIP. The model systems employed are cell lines including MEFs expressing a catalytically dead version of HOIL-1. In addition, the authors perform in vitro reconstitution experiments with purified ubiquitin chains, the LUBAC complex and p62. The main message is that solid p62 bodies are poor substrates for autophagy and that linear, non-branched ubiquitin chains promote solidification. The mechanism remains unclear and some of the effects sizes are rather modest.

We appreciate the positive assessment of our integrated cellular and in vitro approaches and the recognition of the key message that HOIL-1–dependent regulation of ubiquitin architecture critically influences p62 body properties and autophagic clearance.

Major comments:

The key observations mentioned above are convincingly shown. Since the authors don't claim any detailed molecular mechanisms, the number of conclusions in this study are limited.

Overall, the authors are quite careful regarding their conclusion, and therefore the ones that are made in this manuscript are generally well supported. The data regarding the clearance of the p62 bodies presented in Figure 3 should be backed up with additional data. The authors could add a macroautophagy inhibitors such as VPS34 IN1 and/or perform the clearance experiments in a ATG KO/KD cell line to corroborate the contribution of macroautophagy to the clearance.
In addition, a proteasome inhibitor should be used for comparison.
The expertise and resources for the experiments mentioned above are expected to be well within the authors' capacity and should be doable within a few weeks.

Thank you for raising this point. We performed p62-body clearance assays using either a VPS34 inhibitor or MG132, as suggested. We first validated the effects of a VPS34-inhibitor and MG132 and confirmed that both worked properly determined by immunoblotting using antibodies against p62 and ubiquitin (new Fig EV3H). As anticipated, total blockage of macroautophagy by a VPS34 inhibitor further affected the remains of larger aggregates in cells, suggesting that defects of aggrephagy in HOIL-1 mutant cells are partial. Interestingly, we did not observe clear differences between control and MG132-treated conditions at lease in the cells we tested. These new data are shown in a new Fig EV3H-K.

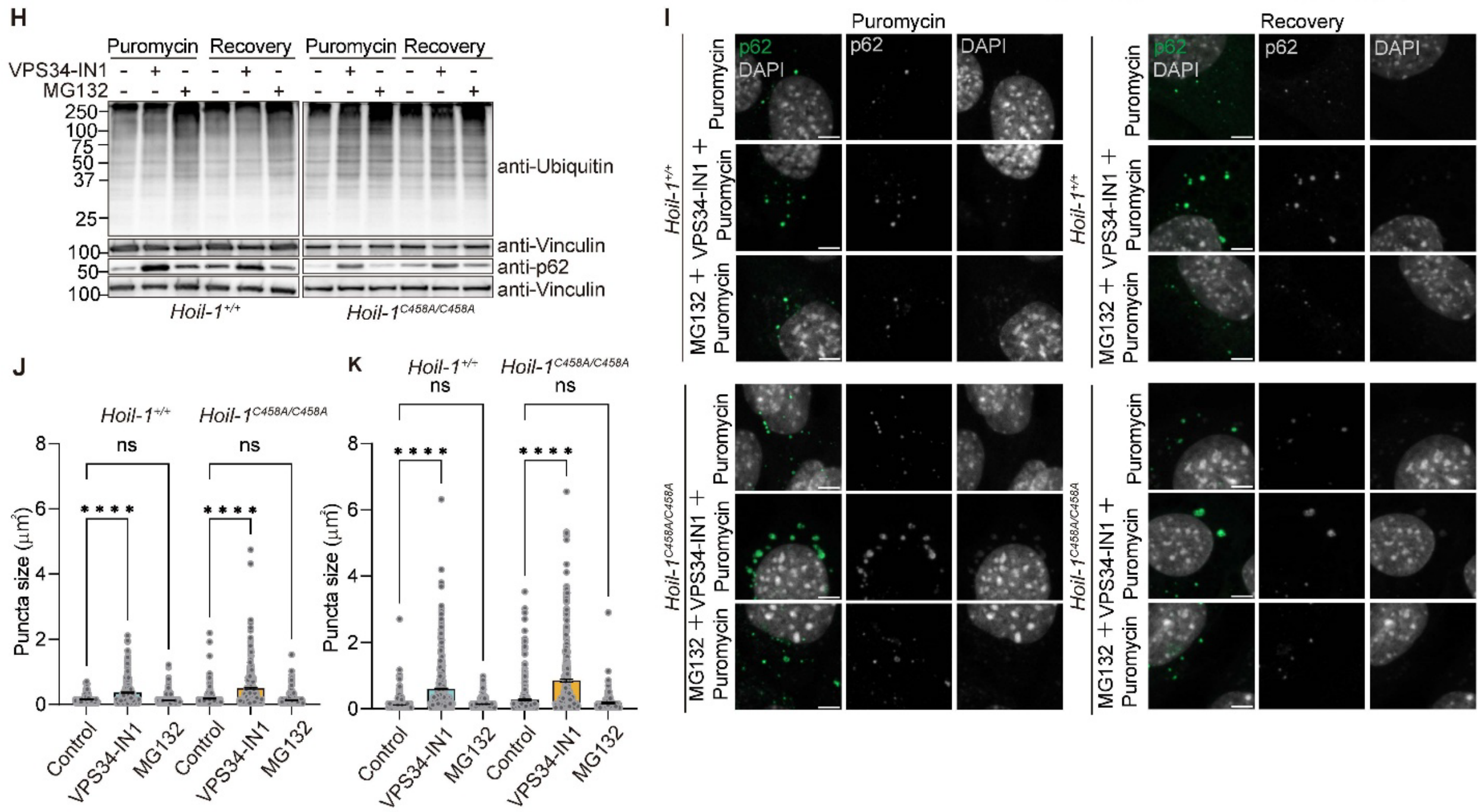

Some of the effects sizes (e.g. Fig. 5 and S5) are very small and it is possible that some of them are below statistical significance if the number of replicates are increased.

We reanalyzed the data, and with the inclusion of additional data points, we observed increased effect sizes during the recovery phase (updated Fig 5C and E), indicating more robust trends. These datasets appear promising and replaced the previous versions in the revised manuscript.

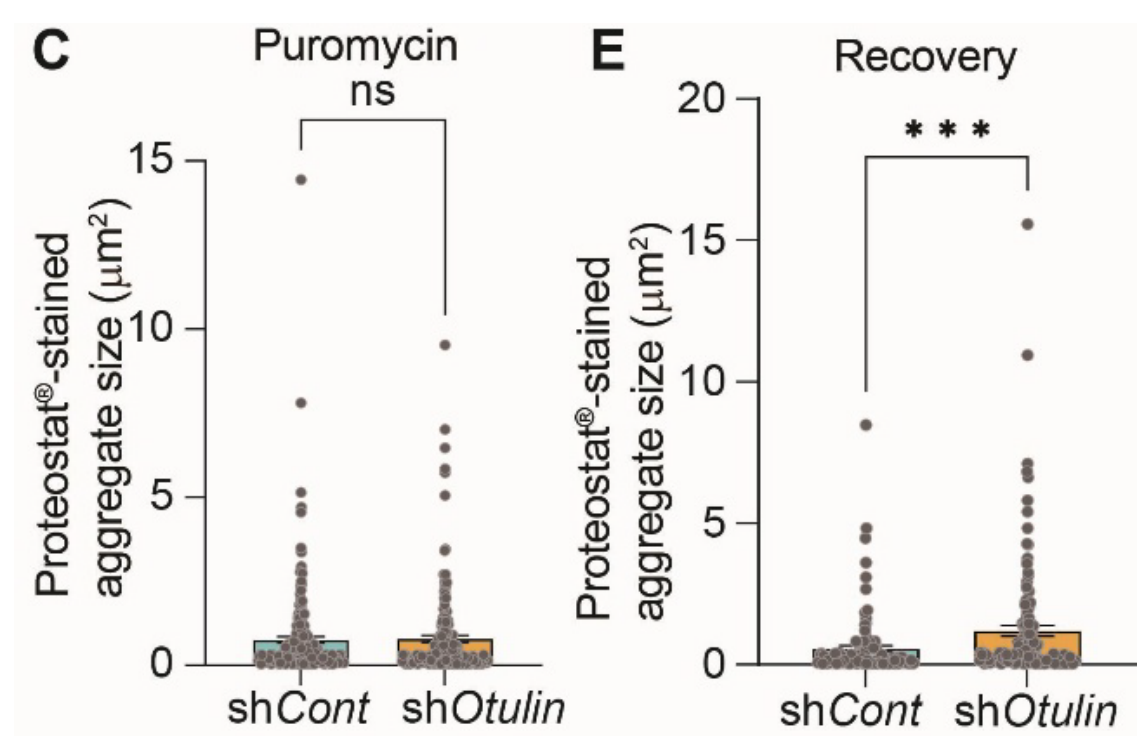

Minor comments:
Figure 1D should be quantified, for example using PCC, Pearson correlation coefficient.

Thank you for this suggestion. We agree that quantitative assessment would strengthen this result and quantified the colocalization in new Fig 1H. The corresponding analysis and statistical reporting have been included in the revised manuscript (in a new Fig 1F).

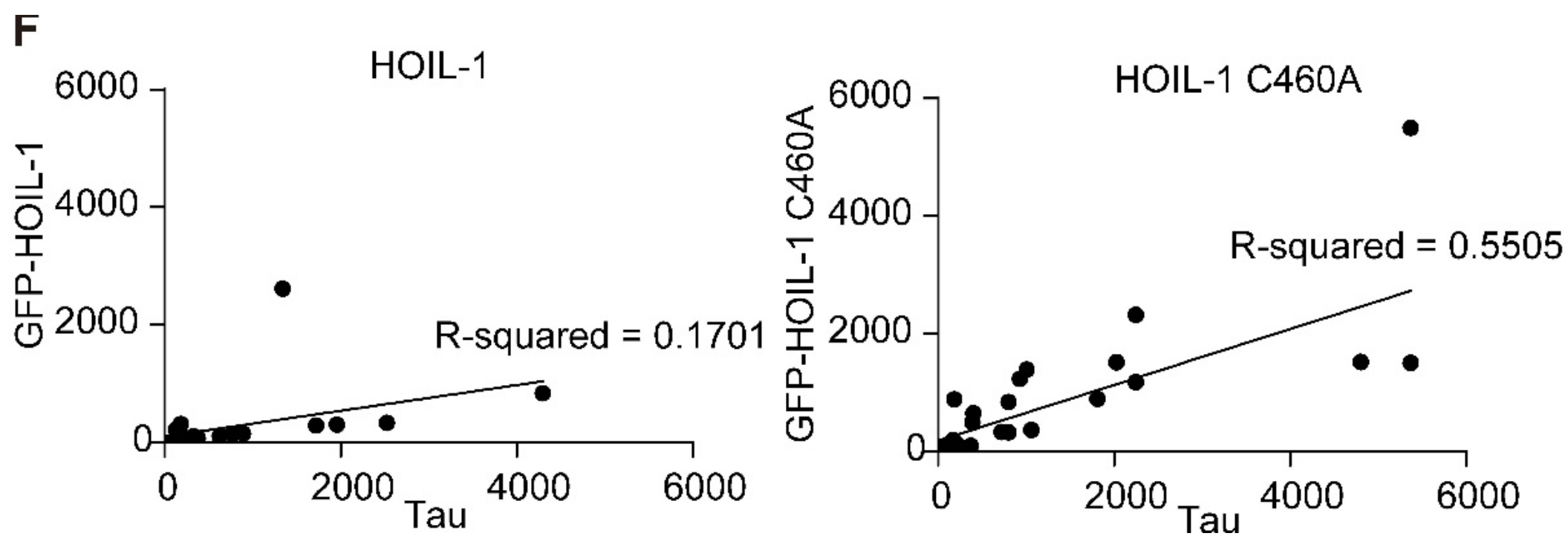

Figure S1 should be quantified.

Original Fig S1A was removed, as the data are not sufficiently convincing. The imaging data shown in the original Fig S1B (new Fig EV1D-F) have already been quantified and are presented.

Figure S3: It should be explained how the region for the profiles are shown were selected.

We included the following clarification in the revised manuscript to address this point (page 7):

"In Fig EV3A and EV3B, representative p62 puncta from *Hoil-1*$^{+/+}$ and *Hoil-1*$^{C458A/C458A}$ MEFs during the recovery phase were selected for line-scan fluorescence analysis of p62 and LAMP2 signals. These analyses show that, in *Hoil-1*$^{+/+}$ MEFs, the residual p62 puncta were typically enclosed within or surrounded by LAMP2-positive vesicle. In contrast, in *Hoil-1*$^{C458A/C458A}$ MEFs, p62 puncta rarely associated with LAMP2-positive compartments, suggesting impaired lysosomal targeting or degradation."

We believe that this explanation improved clarity and guide readers through the rationale and interpretation of the line profile analyses.

It is suggested to include a scheme of the LUBAC complex and its E3 ligase activities in Figure 1A. This will make it more accessible for readers, who are not so familiar with this complex, in particular as HOIP and HOIL can be easily confused.

Thank you for this helpful suggestion. We agree that including a schematic of the LUBAC complex and its distinct E3 ligase activities would improve accessibility for readers less familiar with the system. Accordingly, we added a simplified schematic to Fig 1A highlighting the respective roles of HOIP and HOIL-1 to clarify their functions and avoid potential confusion.

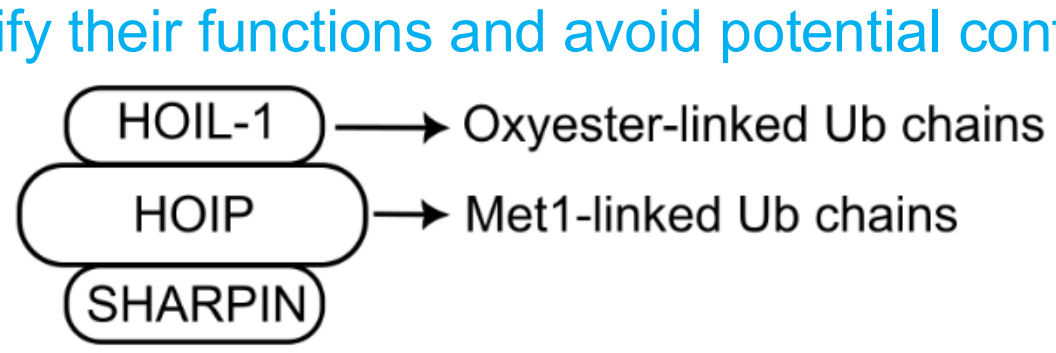

The authors may also want to clarify this in the abstract.

Thank you for the suggestion. We agree that this clarification would be helpful for readers, and we revised the abstract accordingly to more clearly distinguish the roles of HOIP and HOIL-1 within the LUBAC complex.

Significance

As mentioned in the summary. The authors report the observation that an excess of linear ubiquitin chains produced by HOIP in the absence of HOIL-1 activity results in the solidification of p62 bodies and reduced clearance by autophagy. This observation is novel and will be interesting for the proteostasis field.

This reviewer is expert in autophagy and protein degradation, but less so in the LUBAC complex.

Reviewer #3 (Evidence, reproducibility and clarity (Required)):

Summary:

The study 'Excess Met1-ubiquitination leads to solid aggregate formation' by Kaypee et. al suggests a previously unrecognised role for the E3 ligase HOIL-1 in clearing protein aggregates via autophagy (e.g. aggrephagy). In their model, toxic protein aggregates in cells are modified with ubiquitin chains, including M1-linked Ub-chains catalysed by LUBAC (of which HOIL-1 is a component). The HOIL-1 ubiquitin signal is posited to induce trafficking of aggregates to lysosomes for subsequent clearance. However, when HOIL-1 is inactive (catalytic C460A mutation), the pathway is interrupted. As a result, protein aggregates fail to clear, they increase in size and shift their biophysical properties from liquid-like to more rigid, insoluble aggregates. The authors explain their observations by an increasing amount of M1-linked chains on protein aggregates, which occur as a result of 'unrestrained HOIP activity' due to HOIL-1 inactivity (based on previous work). Increasing amounts of M1-chains are posited to promote aggregate formation, aggregate growth, and prevent clearance.

The major claims made in this manuscript are the following:

1. Following the induction of protein aggregate formation (e.g. alpha-synuclein, tau, beta-amyloid, p62 bodies), cells that express catalytically inactive HOIL-1 fail to clear protein aggregates and end up with more, larger and more rigid protein aggregates compared to cells that express WT HOIL-1.
2. The observations made in 1. are due to disruptions in late-stage autophagic flux. While aggregates in cells that express WT HOIL-1 co-localise with autophagy and lysosomal markers, aggregates in cells that express mutant HOIL-1 show autophagic, but not lysosomal markers.
3. An increase in M1-chains (either due to HOIL-1 inactivity or due to OTULIN knockout) is believed to be the cause for claims 1. and 2.

Main methodologies used:

The authors use two cellular systems. The first one is SH-SY5Y cells in which either WT or mutant HOIL-1 are transiently overexpressed (via the pcDNA3.1 plasmid), and physiologically important aggregates (Tau, Abeta, asyn) are induced. The second cellular system is MEF cells in which either WT or mutant HOIL-1 are endogenously expressed; in these cells aggregates are formed crudely through disruption of ribosomal translation. It is questionable if both systems can be compared. Aggregate formation is mainly monitored and quantified via fluorescent microscopy in both fixed and live cells, or via sucrose gradient fractionation to separate soluble

and insoluble (=aggregate) fractions. The rigidity of protein aggregates is analysed in cells via FRAP, size and circularity measurements and 1,6-HD treatment, or in vitro after aggregate formation assays via size and circularity measurements.

The observations are on the whole interesting, though the authors fail to discuss their data in light of previously published work. For example, HOIL-1 KO and KI animals were shown to feature polyglucososan bodies in brain, which is not mentioned. Also, McCrory et al on HOIL-1 chain types is not cited but seems relevant (Figure S4). Yet, the manuscript reports a number of interesting findings, more or less coherently, most useful for scientists embedded in current ubiquitin, autophagy, and LLPS fields. These reviewers believe that this manuscript will make a lot of sense in due course, and be well cited for a first description of the role of HOIL-1 in cellular quality control processes.

A number of improvements seem required to consolidate the findings, and improve readability and impact.

We thank the reviewer for recognizing the interest, coherence, and potential impact of our findings for the ubiquitin, autophagy, and LLPS communities. We appreciate the constructive literature suggestions and incorporated the cited studies, including polyglucosan body in the brain in HOIL-1 animal models (Kelsall et al., 2022) and (McCrory et al. 2022), to strengthen the manuscript (p13 and p9, respectively).

Major:

1. Figure 1A-C: The authors transiently overexpress either WT HOIL-1L or catalytically inactive (C460A) HOIL-1 in SH-SY5Y cells, then induce and compare the formation of protein aggregates (alpha-synuclein, tau, amyloid-beta) in those cells over 72 h. More cells with aggregates were found in cells that overexpressed mutant HOIL-1L. While these findings are interesting, the cellular system used is artificial due to the transient overexpression of HOIL-1 (in presence of endogenous HOIL-1). Crucial controls are missing:

We thank the reviewer for these valuable insights. In response, we carried out the following experiments to address points (a–d) and strengthen the corresponding conclusions.

a. Adding a condition in which no protein is overexpressed, for example via an empty pcDNA3.1 or GFP only vector. This would help ruling out secondary effects due to the transient overexpression. It would also allow to monitor whether the same amount of aggregates form in the empty ctr compared to when WT HOIL-1 is overexpressed.

Yes, we included data from a GFP-only control in the revised manuscript. As expected, expression of the GFP-only vector shows no significant difference compared with HOIL-1 WT. These control data have been fully incorporated in the revised figures (in new Fig 1B-D).

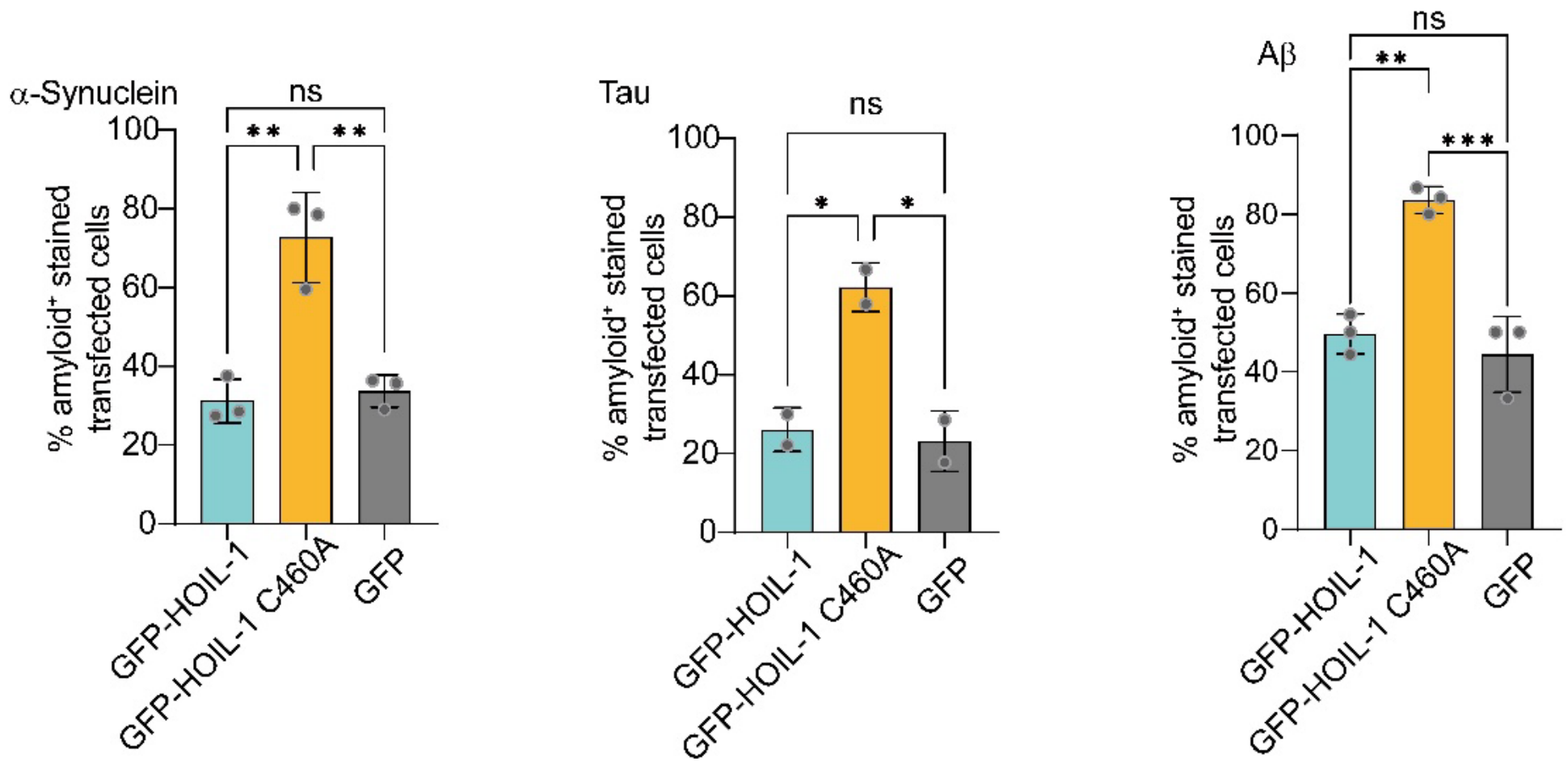

b. Figure legends and raw data points (?) in graphs do not match. The graphs show dubious statistics from 2-3 grey dots, while the figure legend refers to n=100 cells etc. This needs to be fixed.

We apologize for the confusion. The cells were counted as originally described, based on three independent experiments. We clarified this explicitly in the revised manuscript to ensure that the experimental design and statistical basis are clear to readers.

c. Showing Western Blots of HOIL-1, to better understand the levels of endogenous HOIL-1 vs overexpressed HOIL-1 in these cells, and to compare overexpression levels between WT and mutant HOIL-1.

We thank the reviewer for pointing out this issue. In response, we included immunoblot data directly comparing endogenous HOIL-1 levels with those of transfected HOIL-1 (in new Fig EV 1A), allowing a clearer assessment of expression levels and strengthening the interpretation of the overexpression experiments.

A

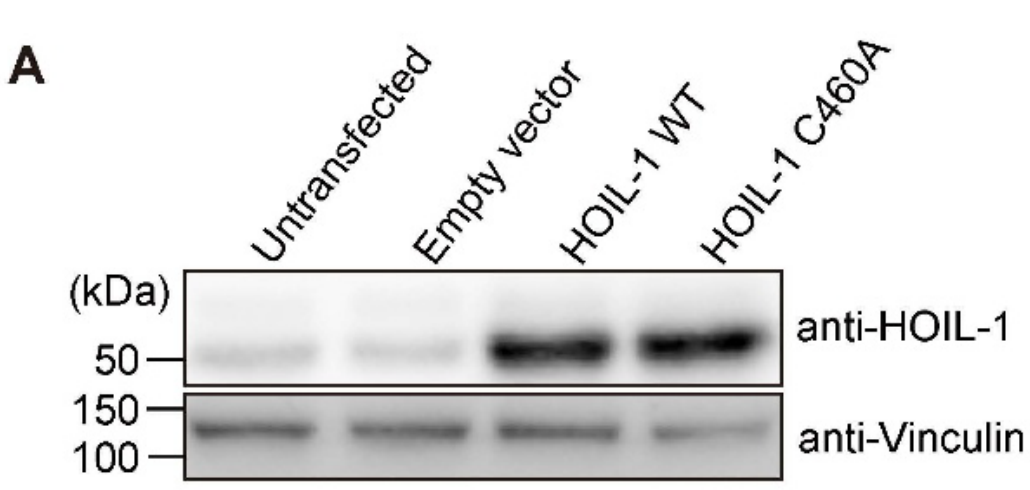

d. The study would also improve by western blotting and IF staining for other LUBAC components such as HOIP and SHARPIN. Do alpha-synuclein aggregates in both WT and mutant conditions co-localize with the other LUBAC components, and are there any differences between WT and mutants. This would further help strengthening the claims made in Figure S1A: '...suggesting that LUBAC is recruited to or retained within α-Synuclein aggregates.' And in the discussion: 'we found that LUBAC components were sequestered in aggregates, as evidenced by microscopy and gradient fractionation of soluble and insoluble proteins, confirming the direct involvement of LUBAC in aggregate processing.'

This is indeed a critical point, and we acknowledge that we did not previously have direct evidence addressing it. To resolve this issue, we agree that the most appropriate approach is immunofluorescence/immunocytochemistry to directly assess the recruitment of LUBAC components to α-synuclein aggregates. As suitable antibodies for ICC detection of endogenous SHARPIN and HOIP are currently unavailable, we have selected to use epitope-tagged versions of SHARPIN and HOIP and generated new plasmids (mScarlte-I3 tagged HOIP or SHARPIN). As expected, we observed colocalization of both HOIP and SHARPIN with aggregates. Interestingly, co expression with SHRAPIN lead to non-fibril like α-synuclein in comparison, HOIP co expression led to the fibril-like aggregate. These data are included in new Fig EV 1B and C.

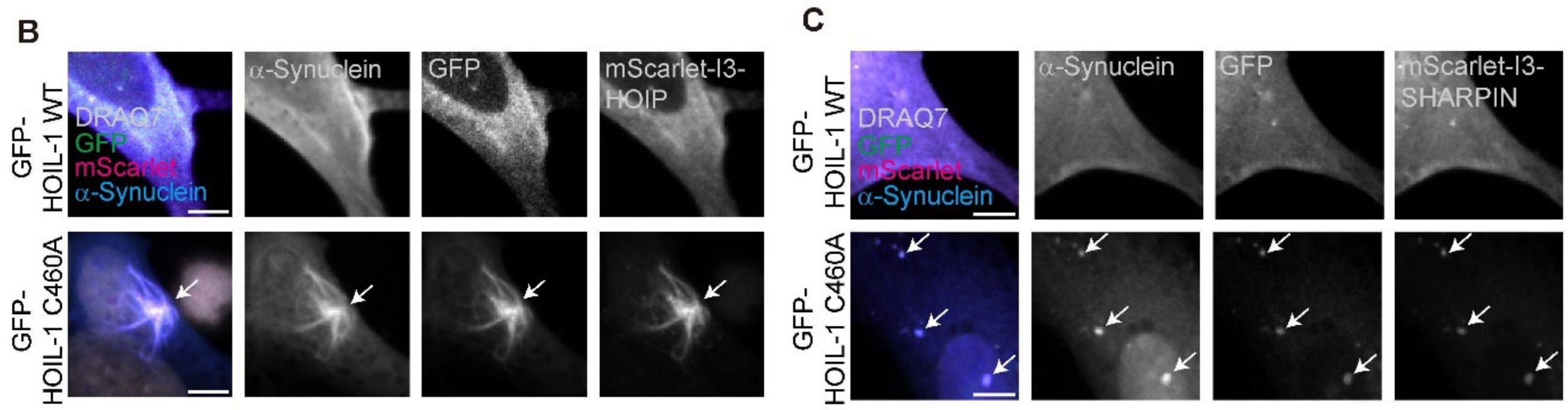

2. Figure 2A-F: The authors change to a genetic-derived system (comparing endogenously expressed HOIL-1 WT with mutant HOIL-1 based on MEF cells from their mouse models). However, they use puromycin to produce aggregates from random protein homeostasis defects, which yes leads to aggregates, but is not as nice as the induced generation of neiurodegeneration-relevant aggregates. It was observed that after 2 h of puromycin treatment, cells accumulate p62-positive protein aggregates, and in during recovery (2 h washout), the aggregates in the HOIL1 mutant cells outgrow the aggregates in the WT HOIL1.

a. However, the authors claim that: 'While Hoil-1+/+ MEFs efficiently cleared puromycin-induced p62 bodies,...', which is not supported by the data shown here. When comparing WT in panel C with WT in panel E, it becomes evident that the average number of p62 puncta before and after recovery is the same (around 5 puncta/cell in both pre and post washout conditions). A similar observation can be made for the mutant (around 12 puncta/cell in both pre and post washout conditions). Can the authors please amend their claims, or comment and perform a direct statistical comparison between the pre and post recovery conditions to test for clearance of p62 puncta in the WT after puromycin washout.

We thank the reviewer for pointing this out. We agree that our previous description was not sufficiently precise and revised the text to clarify that the key difference lies in the size of the aggregates rather than their number, with larger p62 bodies being more prominent. We ensured that this distinction is clearly explained and not overstated. Consistent with this interpretation, in Fig S2 we observed differences in aggregate size but not number; these data have been included in the revised manuscript (new Fig EV2A-C).

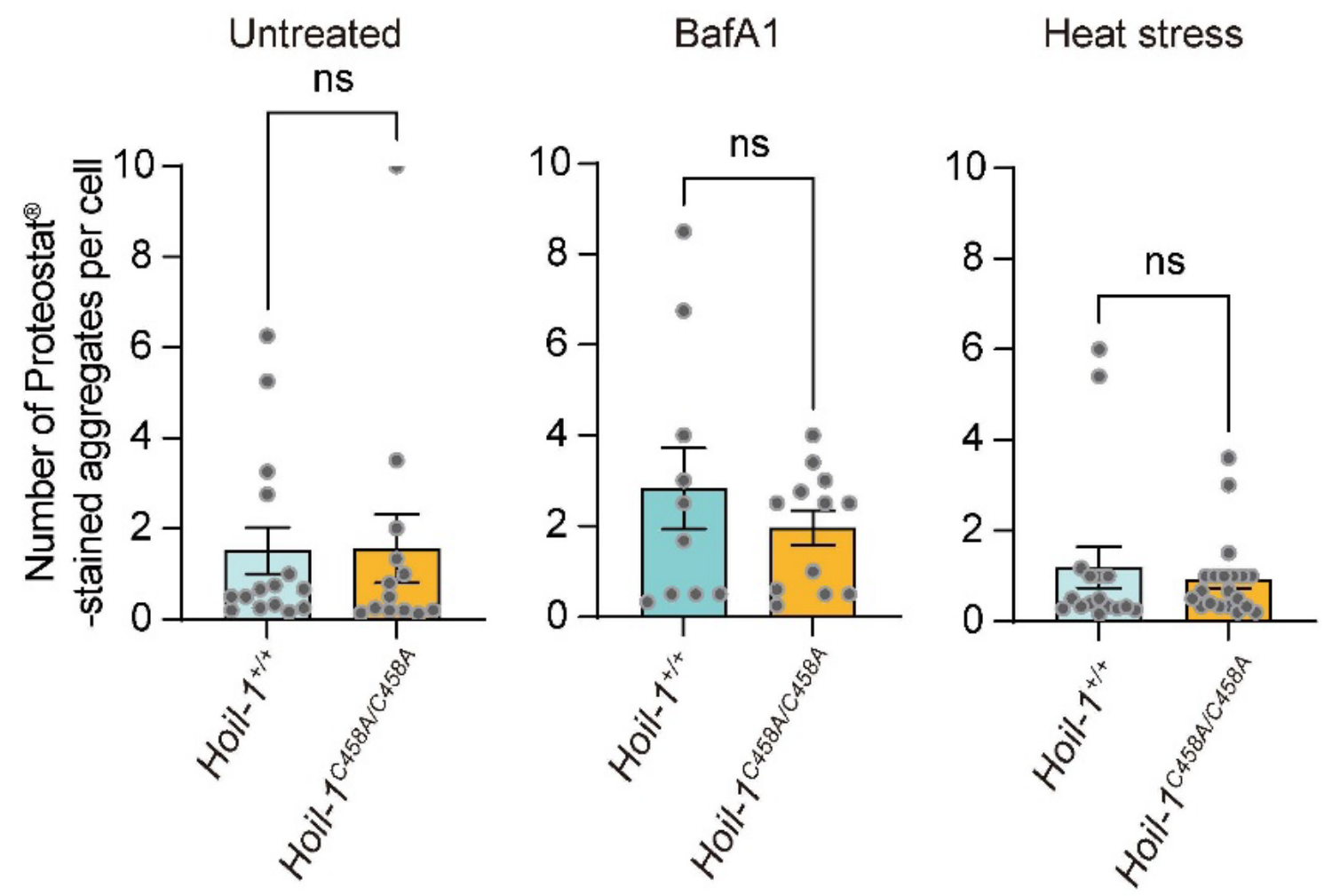

We revised the text accordingly (in page 6):
"Under both stress conditions, *Hoil-1*$^{C458A/C458A}$ MEFs accumulated similar in number yet significantly larger aggregates than *Hoil-1*$^{+/+}$ MEFs (Fig EV2A–C),"

b. The authors state that: 'These findings indicate that although HOIL-1 catalytic activity is dispensable for the initial formation of puromycin-induced aggregates, it is essential for their subsequent clearance.'
As long as clearance of p62 bodies in the WT is not clearly shown, the second part of this sentence should be amended/removed.

We agree with the reviewer and removed this sentence from page 6.

c. The experiments shown would improve by adding a t = 0 condition. How many p62 granules are present before puromycin treatment? Is there already a basal difference between WT and mutant HOIL-1L cells?

This is another important point. We included a dataset obtained in the absence of puromycin treatment as a baseline control (see a new Fig EV2D). Under these untreated conditions, we observed minimal to no p62 signal in either *Hoil-1*$^{+/+}$ or *Hoil-1*$^{C458A/C458A}$ MEFs, indicating that aggregate formation is specifically induced by proteotoxic stress.

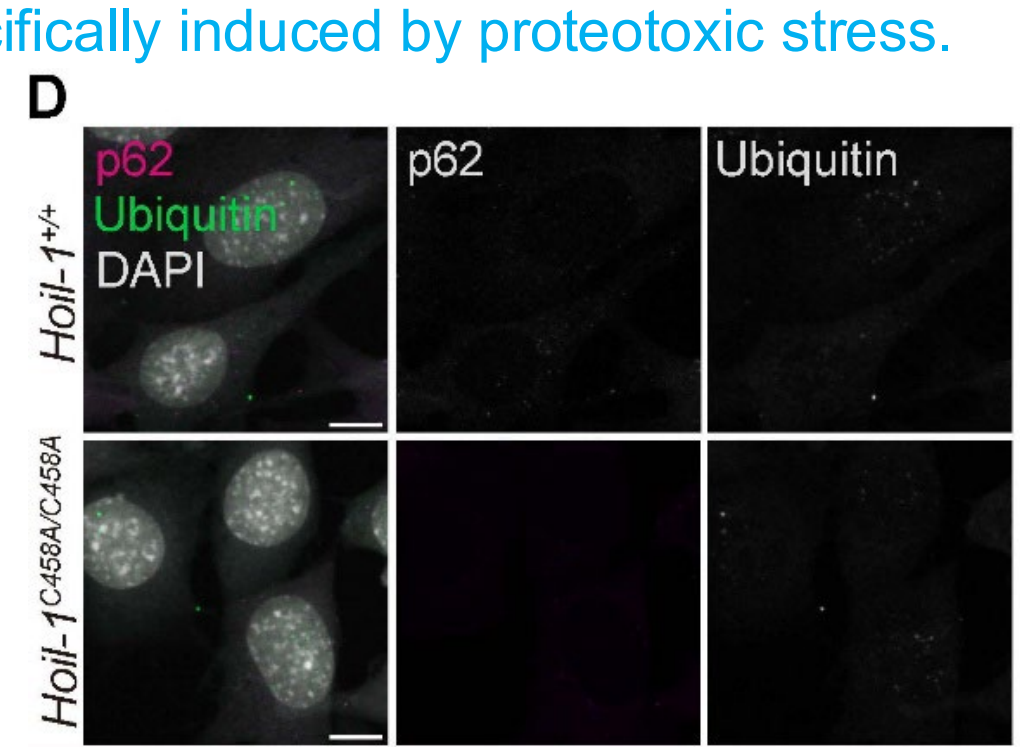

3. Figure S1A: The authors claim that other LUBAC components co-localise to protein aggregates, based on sucrose gradient fractionation and the presence of the respective proteins in the insoluble fractions. Could the authors perform IF and stain for other LUBAC components

(SHARPIN and/or HOIP) in their MEF cell system to directly validate this claim?

This is a critical point, and we have carefully considered it. As also noted by Reviewer #1 (point 6), we have decided to remove this dataset and the accompanying text to avoid overinterpretation. However, as suggested in point 1(d), we directly analyzed the presence of LUBAC components within aggregates using immunocytochemistry in the revised study (new Fig EV1B and C).

4. Figure 3G-H: The authors created a GFP-mCherry-p62 reporter system in both their WT and mutant HOIL-1 MEF cells and performed live cell imaging following puromycin treatment, which allows monitoring of both aggregate formation and loss of GFP signal due to the acidic lysosomal localisation. Excitingly, the ratio of GFP/mCherry in the later timepoints is reduced in the WT compared to mutant HOIL-1, indicating that HOIL-1 activity is required to traffic p62 bodies to lysosomes.

a. In panel G, a surprisingly large amount of p62 granules are present at t0, which (according to the relevant method section) is the time of puromycin treatment. This observation can be made for both WT and mutant cells. After 80 min of puromycin treatment in the WT, the majority of these puncta are cleared. Can the authors please comment on this high amount of p62 granules at t0 (before the effects of puromycin? And also on the observation that after 80 min there are now less granules than before puromycin? In case that t0 indicates the time of puromycin washout rather than puromycin addition, could this please be clarified in the methods or figure legend?

We apologize for the confusion and thank the reviewer for highlighting this point. We have clarified in the revised manuscript that the t = 0 time point for live-cell imaging corresponds to the start of image acquisition after puromycin pre-treatment, at which stage puromycin-induced p62 bodies have already formed. Therefore, this time point does not represent untreated cells, but rather reflects technical constraints of the live-imaging setup.
To make this clearer, we have added an experimental timeline to Fig. 3G and included ICC analyses of cells that were not exposed to puromycin (new Fig. EV3C). Consistent with our interpretation, we observed little to no p62-body formation in untreated cells, confirming that the puncta present at the imaging start point arise as a consequence of puromycin treatment.

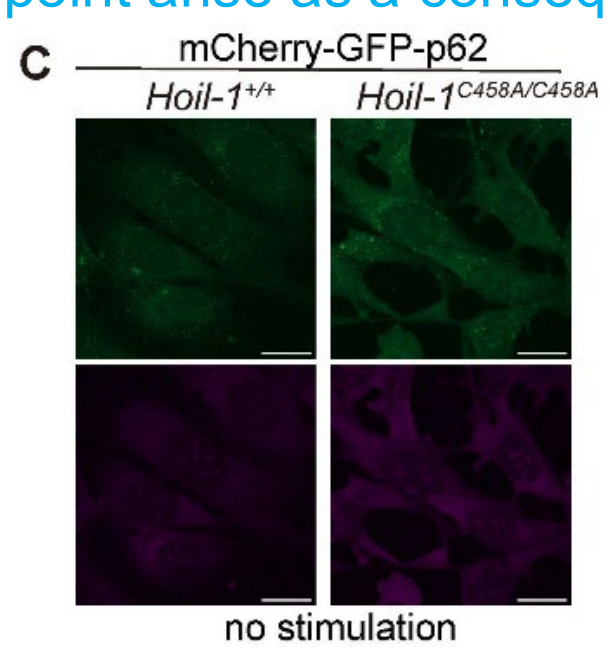

b. Panel H would improve by adding the quantifications for t=0 (or ideally for all the time points).

We added the corresponding data point (t=0), which is the starting time point of live imaging (see a new Fig 3H).

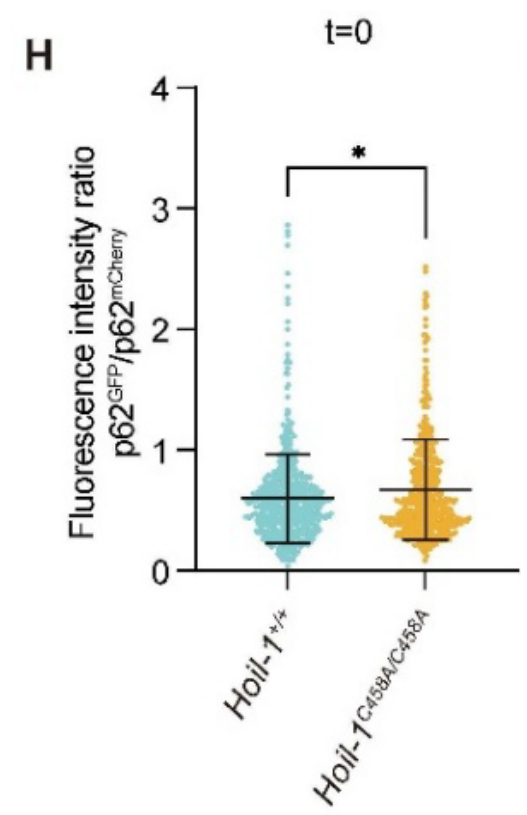

c. Fig S3C-D: Same comments as before but for GFP-mCherry-LC3.

Response corresponding to point 4(a) also applies here. We apologize for the confusion and have clarified in the revised manuscript that the t = 0 time point for live-cell imaging corresponds to the start of image acquisition after puromycin pre-treatment, at which point puromycin-induced LC3-positive puncta are already present. To improve clarity, we have added an experimental timeline to Fig EV3F. In addition, we included analyses of untreated cells (new Fig. EV3E). Under these basal conditions, we observed very few LC3-positive puncta and no appreciable differences between *Hoil-1*$^{+/+}$ and *Hoil-1*$^{C458A/C458A}$ MEFs, indicating that the observed changes arise in response to proteotoxic stress rather than under steady-state conditions.

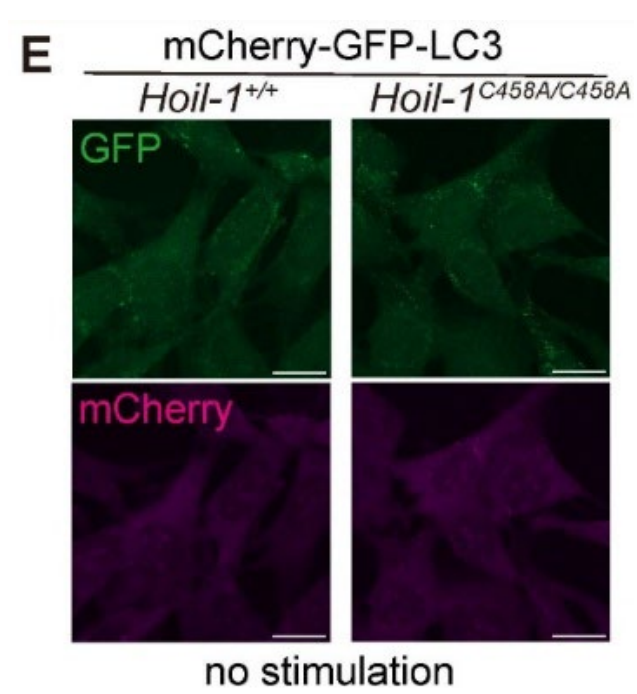

As suggested by the reviewer, we also included the t = 0 quantification in the revised analysis (new Fig. EV3G). At the start of imaging, the GFP/mCherry ratio was already elevated in *Hoil-1*$^{C458A/C458A}$ MEFs compared with *Hoil-1*$^{+/+}$ MEFs. This observation suggests that a proportion of LC3-positive structures had already reached acidic autolysosomal compartments before imaging began, particularly in wild-type cells, whereas this process was delayed in *Hoil-1*$^{C458A/C458A}$ MEFs. These findings are consistent with our overall model that HOIL-1 catalytic activity facilitates the efficient autophagic degradation of condensates/aggregates.

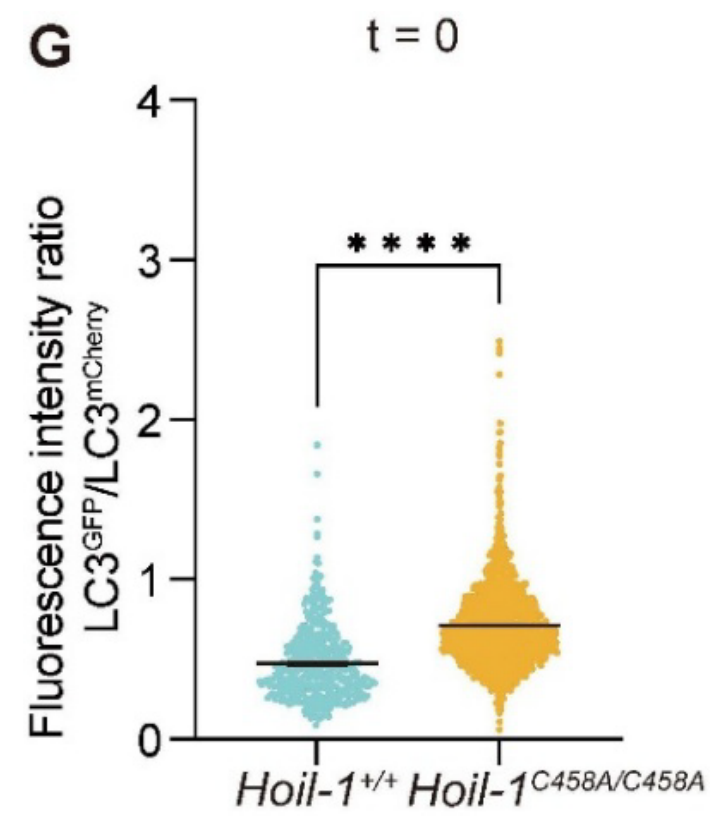

5. Fig. S4B and Fig 4A-B: The authors state that circular aggregates are more soluble and have more LLP characteristics, whereas non-circular aggregates are less soluble and have more aggregate-like characteristics. However, the aggregates shown in Fig. S4B are un-circular but easily dissolve in response to 1,6-HD treatment, which seems contradictory. On the other hand, the aggregates shown in Fig 4B in HOIL-1 mutant cells appear much rounder than the ones in S4B, but do not dissolve in response to 1,6-HD treatment. Can the authors please comment on these discrepancies?

Thank you for raising this important point. We agree that circularity alone is not a definitive proxy for material state, and that apparent discrepancies can arise depending on context. In our assays, circularity reflects geometry, whereas 1,6-hexanediol sensitivity reports material properties, which are influenced not only by shape but also by underlying molecular interactions.
In Fig EV4B, the non-circular condensates likely represent irregular but still liquid-like assemblies, consistent with their sensitivity to 1,6-hexanediol. In contrast, the rounder aggregates observed in Fig 4B in HOIL-1 mutant cells likely reflect matured, compact structures whose apparent circularity arises from surface tension during solidification yet are resistant to 1,6-hexanediol due to stabilized intermolecular networks.
We clarified in the revised manuscript that circularity and hexanediol sensitivity capture distinct and complementary properties, and that aggregate shape alone should not be interpreted as a direct measure of liquidity (see page 9).

"Circularity and 1,6-HD sensitivity capture distinct and complementary properties, and that aggregate shape alone should not be interpreted as a direct measure of liquidity."

6. Fig. 4E-G. Here the authors suddenly switch to an in vitro aggregate-formation assay using mCherry-p62. In-vitro M1-chain reactions with either WT LUBAC or LUBAC with mutant HOIL-1L, together with the respective M1-chain reaction product, are added.
This is not clear from the figure, and a schematic, as well as a gel (Coomassie) should be included to show component purity and indicate the biochemical in vitro nature of the experiment. It is good to have this breadth of methods, but does not help in the presentation if all figures look alike.

We thank the reviewer for this constructive comment. In response, we included Coomassie-stained gels to document component purity (a new Fig EV4C) and added schematic illustrations (a new Fig 4E) to clearly convey the experimental setup.

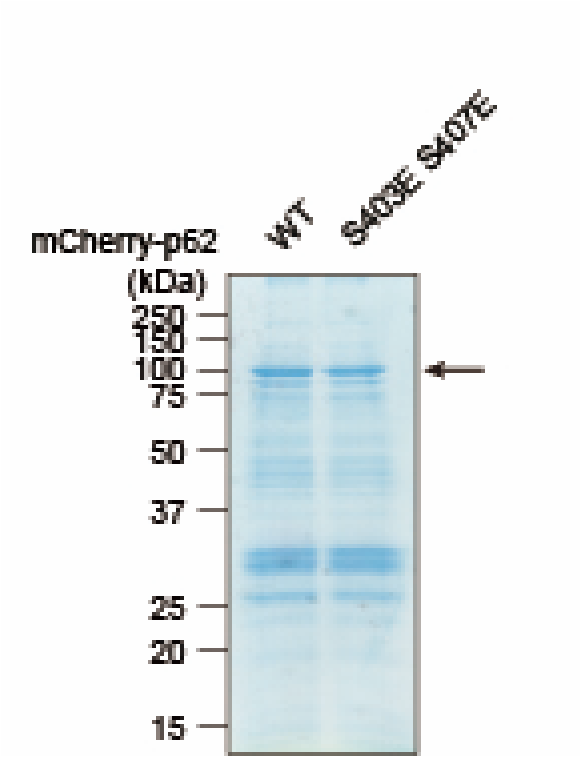

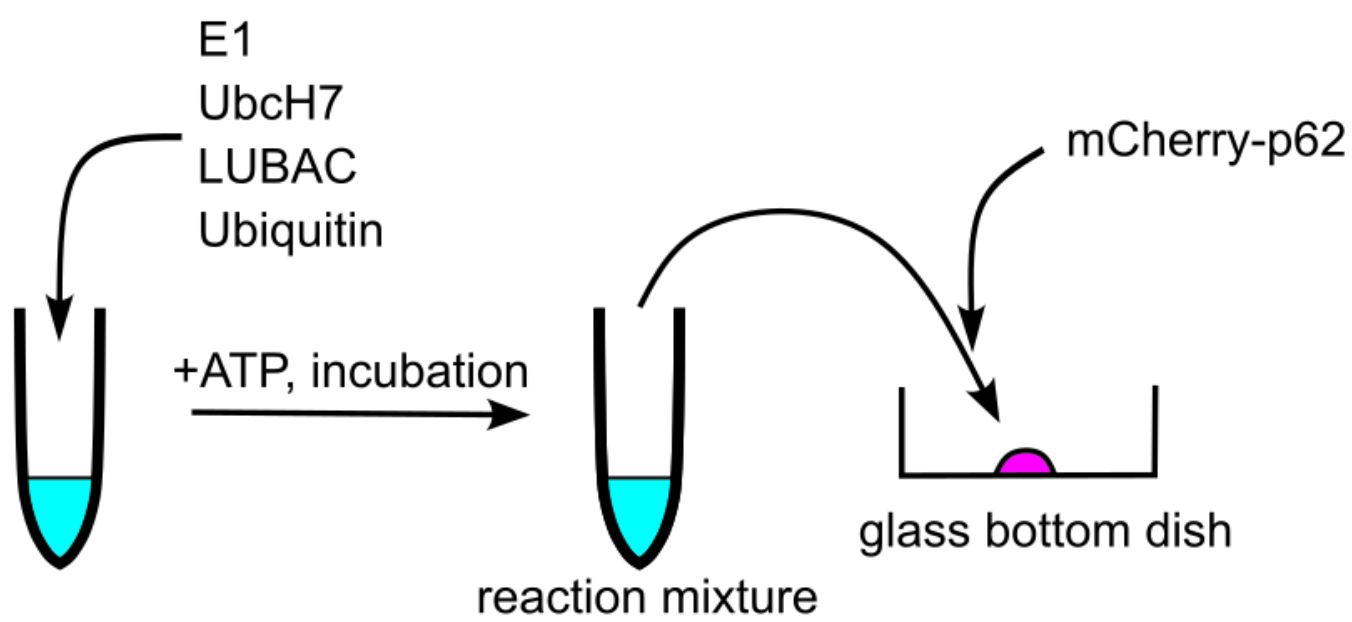

a. The key difference to the cellular situation is p62 aggregates are not directly ubiquitinated here, and instead ubiquitin chains are (non-covalently) added to samples. Can the authors please make this important difference clearer in their text? Why not directly ubiquitinate mCherry-p62 via LUBAC (WT vs mutant HOIL-1) and then perform an aggregation assay on the reaction product?

We thank the reviewer for raising this point. This is indeed a very interesting yet technically challenging issue—namely, identifying the relevant substrate(s) of LUBAC in the regulation of aggregate or condensate formation. In this study, we focused on ubiquitin itself as a primary substrate, reasoning that LUBAC-mediated formation of unconjugated ubiquitin chains can profoundly influence condensate properties. However, given the central role of p62 in this pathway, it is also plausible that p62 is directly ubiquitinated by LUBAC. To address this possibility, we performed in vitro ubiquitination assays using p62 as a substrate under the same condition of LLPS assays and found that p62 was not ubiquitinated, suggesting that direct ubiquitination of p62 by LUBAC is likely not involved in the process of LLPS at least under the condition we tested (Fig EV4E). As new data comparison analysis of p62 WT and p62 SE mutant may also support this idea that unconjugated Met 1 ubiquitin chains interacting with p62 makes larger sized condensates (new Fig EV4F), which is related to minor point #6.

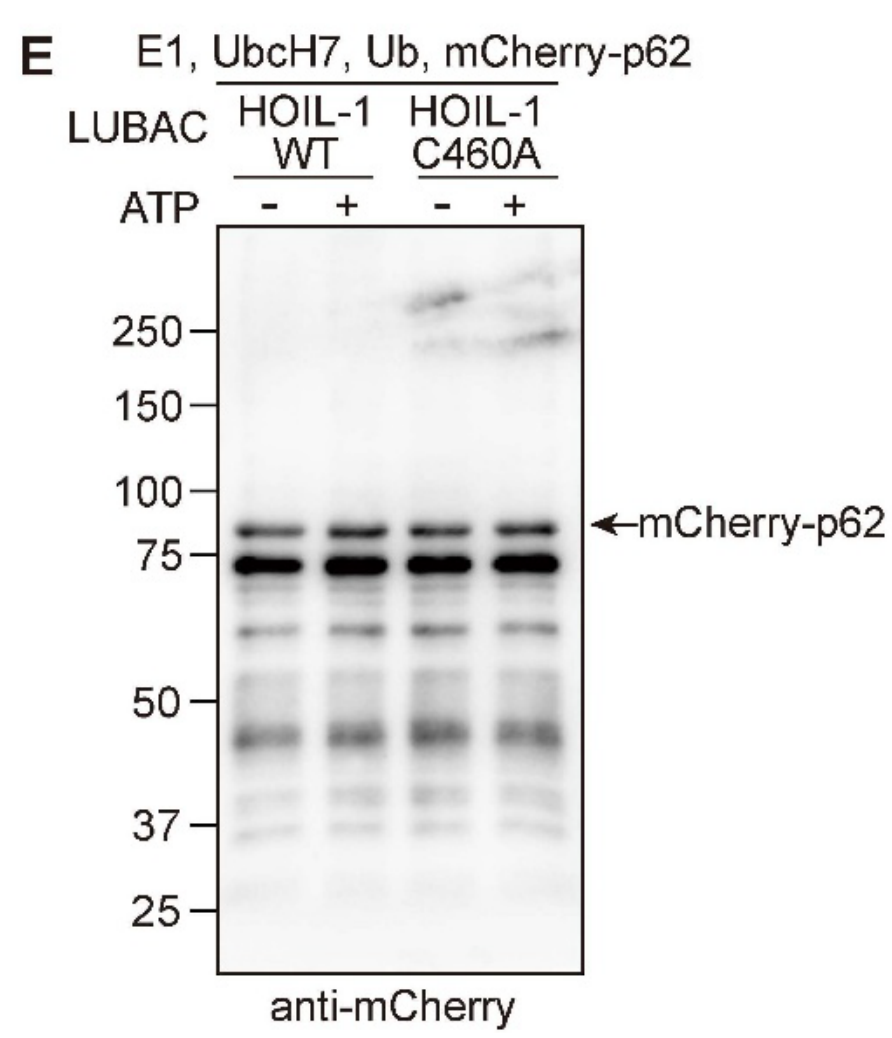

b. Can the authors please clarify whether the reaction was inactivated prior to addition to the aggregate-formation assay? If not, the enzymes might still be active at the point of aggregate formation, and the observed effects might be influenced by enzymatic activities and not only the presence of different M1-chain architectures.

This is an interesting point. In our *in vitro* ubiquitination assays, samples are snap-frozen after incubation, indicating that residual enzymatic activity may persist in both wild-type LUBAC and LUBAC containing the HOIL-1 mutant. Fully inactivating LUBAC under these conditions is technically challenging, as additional inactivation steps lead to ubiquitin chain aggregation. Nevertheless, because clear differences are consistently observed between samples in which HOIP remains active in both conditions, we interpret the observed effects as specifically attributable to HOIL-1 ligase activity.

7. Fig. 5B-C: The M1-specific DUB OTULIN is knocked down (again, cells) to increase the overall amount of M1-linked Ub-chains present in cells. P62 aggregate formation is induced and the authors claim that the increase in M1-chains influences aggregate size. This claim would be strengthened if it was directly shown that M1-chains form on p62 aggregates in this assay, for example via IF using an M1-antibody (and potentially a total ubiquitin antibody). This would also enable to directly compare the abundance of M1-chains between conditions (ctr vs Otulin, WT vs HOIL-1L mutant).

We appreciate the reviewer for raising this important point. Having validated the specificity of the Met1-linked ubiquitin antibody for immunocytochemistry, we performed the suggested experiments using this approach in the revised study (a new Fig 5F). As expected, we observed Met 1-linked ubiquitin signal with p62 bodies.

Minor:

1. Figure 1 D: The authors state that 'Notably, HOIL-1 C460A was detected within these structures, as demonstrated by its colocalization with tau aggregates' and show a co-localisation comparison between WT and mutant HOIL-1L. This sentence implies that WT HOIL-1 was not detected in aggregates, however the chosen image of the WT cells does not show any obvious tau aggregate, even though aggregates were induced in this condition according to 1A-C. The better comparison would be to pick an image that includes a tau aggregate. Moreover, this experiment would benefit from quantification, calculating the percentage of total aggregates that co-localise with WT HOIL-1L vs with mutant HOIL-1L.

We thank the reviewer for raising this point. In response, we analyzed these data as outlined in our reply to Reviewer #2, minor comment 1, and incorporated the corresponding quantification and clarification in the revised manuscript (new Fig 1F).

2. Figure S1A: The authors state that 'Cells expressing HOIL-1 C460A displayed a pronounced accumulation of high-molecular-weight α-Synuclein species in the insoluble pellet fraction.' While the difference seen by Western Blot is apparent and seems to match Fig 1 A-C, it is not very strong. Moreover, the relevant comparison (WT vs mutant) is made between two different blots/membranes, and it is difficult to assess equal input solely based on the TCL lane. This experiment could be improved by normalising samples (for example via BCA) and by loading

and imaging the two conditions (or at least the TCL and pellet samples) on the same membrane.

The authors also state that: 'LUBAC subunits, HOIP, HOIL-1, and SHARPIN, were also enriched in this fraction, suggesting that LUBAC is recruited to or retained within α-Synuclein aggregates.' Both Sharpin and HOIL-1 seem to be present in similar levels in the pellet fractions of WT and mutant HOIL-1. Overall HOIP levels seem to be significantly increased in the mutant over the WT (see TCL lane), and to a similar level in the pellet fraction. It would be great if the authors could include these observations in their interpretation.

Since the data shown in the original Fig S1A were not sufficiently convincing, we removed this panel and the associated text from the revised manuscript. To address the underlying question more rigorously, we instead performed immunocytochemistry to directly assess whether other LUBAC components, specifically SHARPIN and HOIP, localize to protein aggregates, as outlined in point 3(d) (new Fig EV1B and C).

3. Figure 2G: Similar to before, this experiment would improve if the authors could find a way to normalise samples between conditions prior to sucrose gradient fractionation or have the most relevant samples on the same blot. It is challenging to properly interpret the results while the bands in the total cell lysate (TCL) lane do not have similar intensities between samples. A blot in which only the TCL and the pellet samples of all conditions were loaded onto the same gel would solve this, allowing for a better comparison between conditions.

Based on what is shown in panel G, the authors should amend their claim: 'Consistent with microscopic observations, denser fractions from Hoil-1C458A/C458A MEFs contained increased signals of p62 specifically during the recovery phase (Figure 2G).' It is not apparent that more p62 is present in the insoluble fractions of mutant HOIL-1 cells after puromycin treatment. The band intensities look very similar (This is different for the recovery condition, which shows a strong difference, as stated).

We agree with the reviewer's interpretation of the current data. Based on the bands shown in Fig 2I, it is not appropriate to conclude that p62 is increased in the insoluble fractions of *Hoil-1*$^{C458A/C458A}$ MEFs immediately after puromycin treatment, as the signal intensities appear comparable between genotypes. We therefore amended the text accordingly and restrict this conclusion to the recovery condition, where a clear difference is observed. The revised manuscript reflected this more precise and accurate interpretation (page 6).

"Consistent with microscopic observations, denser fractions from Hoil-1C458A/C458A MEFs contained increased signals of p62 <u>specifically during the recovery phase</u>."

4. Figure S2: Similar to before, the authors induce protein aggregate formation and compare cells endogenously expressing WT vs mutant HOIL-1L. The size of aggregates increases in mutant cells under proteotoxic stress. What happens to the number of aggregates per cell in these conditions? Does it also increase, or is it just the size?

Thank you for raising this point regarding aggregate number. We analyzed this parameter and found that the number of aggregates is comparable between conditions, whereas their size differs, as shown in the original Fig S2 (new Fig EV2). Because this observation relates directly to issues raised for the main Fig 2, we included these data in the revised manuscript (new Fig EV2A-C).

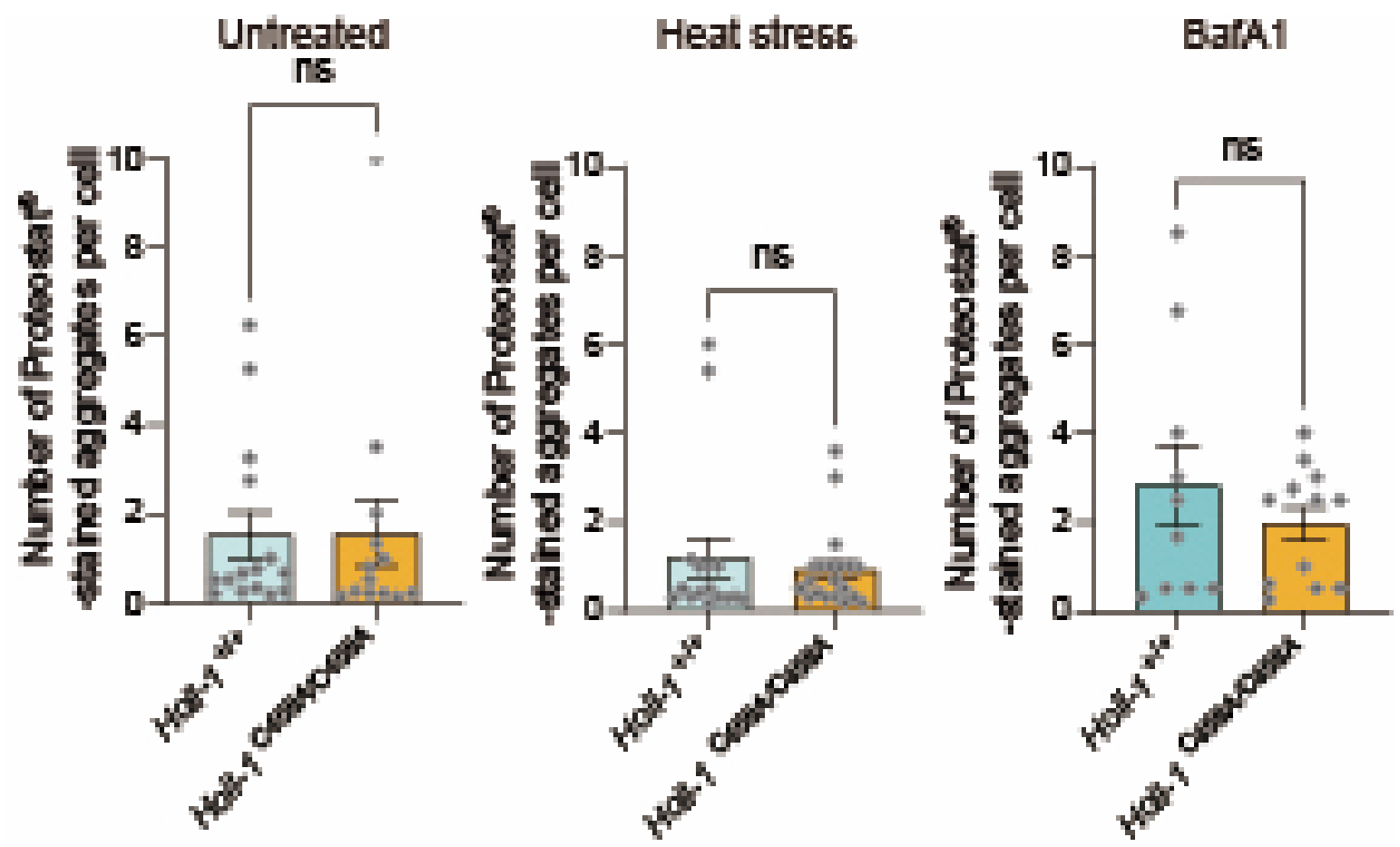

5. Fig. S4A: Here the authors analyse the circularity of p62 aggregates in HOIL-1L mutant cells after recovery from puromycin treatment. This experiment would improve if the same analysis could be performed for the WT cells and for the pre-recovery condition (under the condition that large enough granules are present), allowing to make a comparison between WT and mutant, as well as between pre- and post-recovery.

We analyzed data as suggested and included in the revised manuscript (new Fig EV4A).

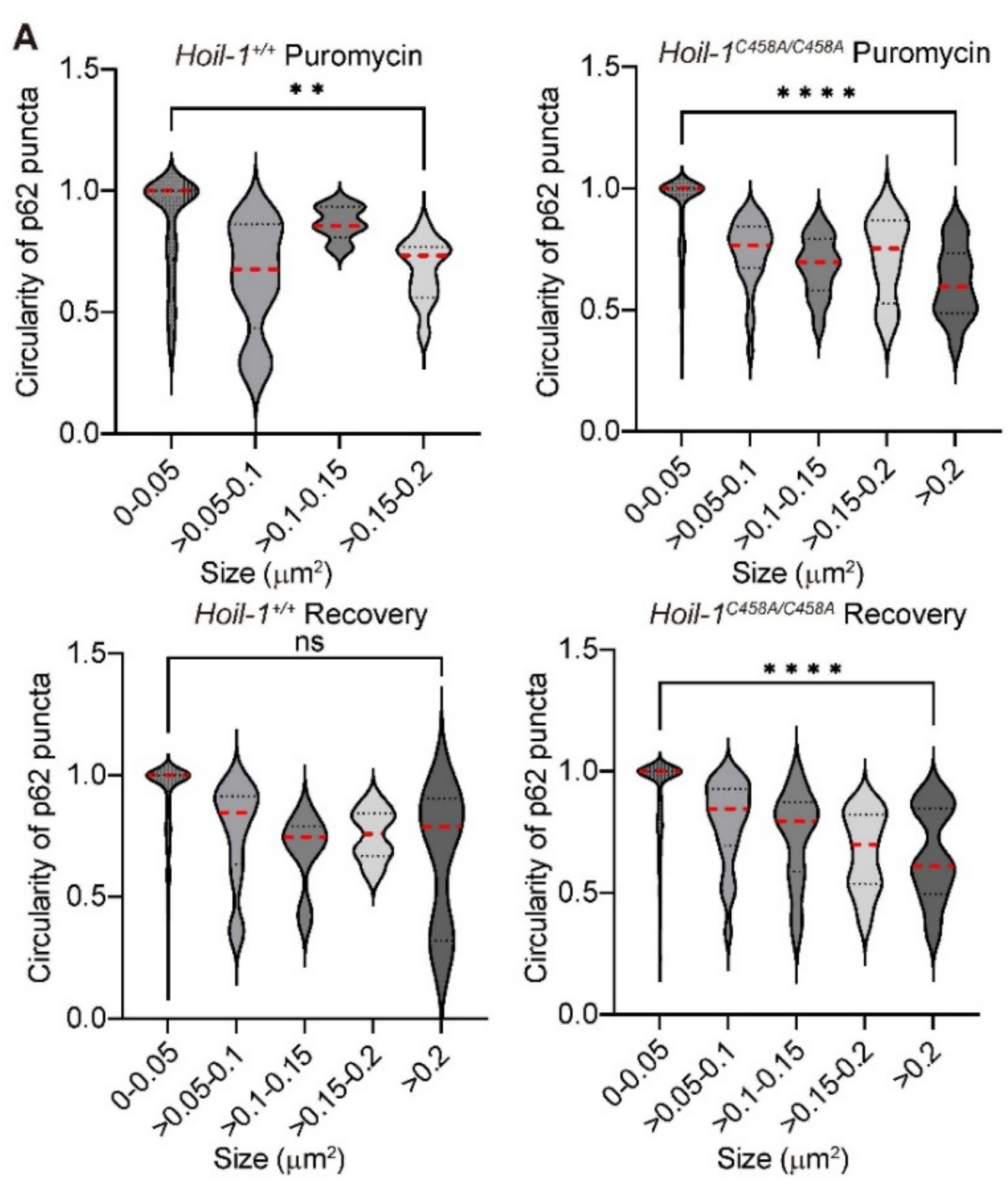

6. Fig. 4H-J: The authors use a p62 mutant that is known for its enhanced ubiquitin affinity, repeat Fig. 4E-G and state: 'These aberrant condensates were similar to those observed in a

reaction using wild-type p62.' Can the authors please comment on what they conclude from this similarity and why this experiment was performed? A quantitative comparison (condensate size and circularity) between WT p62 and mutant p62 may further be useful here.

Indeed, we did not initially include the necessary direct comparison between p62 WT–based and p62 SE mutant–based datasets to fully support this statement. Upon careful reanalysis (using the same original datasets), we identified an interesting and previously unappreciated observation: in reactions containing the HOIL-1 catalytic mutant, condensates formed with p62 SE mutant show significantly reduced circularity. We therefore included these comparative datasets in the revised manuscript (new Fig EV4F) and described in the text (page 10). We thank the reviewer again for highlighting this important point.

“Comparison of condensate size and circularity between wild-type p62 and the phospho mimietic p62 mutant using the same dataset revealed that p62 condensates formed in the presence of HOIL-1 C460A-generated ubiquitin chains were less circular (Fig EV4F). These findings suggest that interactions between p62 and ubiquitin chains contribute to the regulation of condensate morphology.”

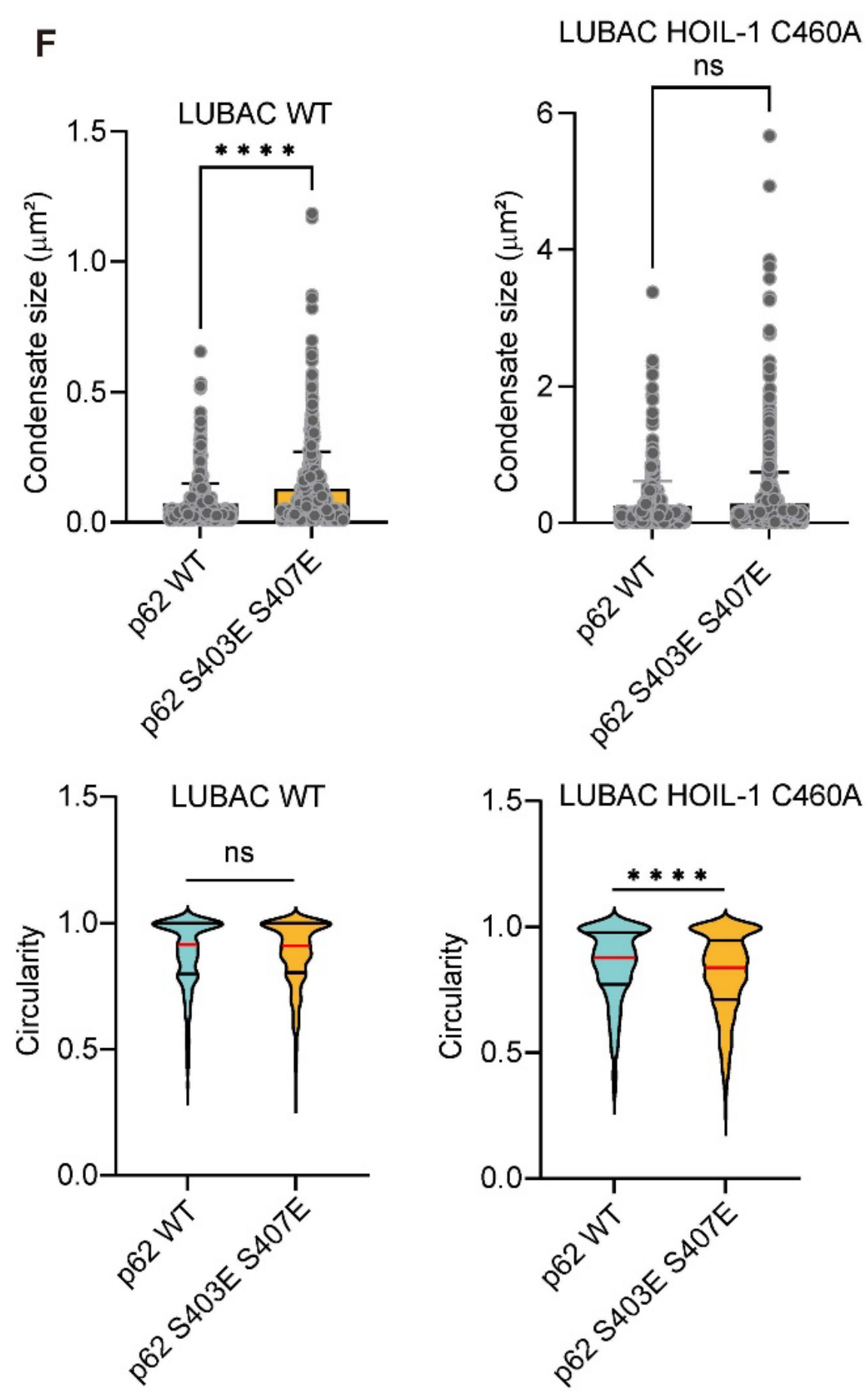

7. Fig. 5A: Here the authors pulldown M1-linked Ub-chains in WT vs mutant HOIL-1 cells, with or without puromycin-induced aggregate formation. More M1-chains are observed in mutant HOIL-1 cells under puromycin treatment, but the difference is very subtle. The conclusions

drawn from this experiment could be strengthened by including alternative methods, for example (if available) Ub-AQUA to measure the abundance of M1-chains, or using the M1-antibody for IF analysis.

We fully agree with the reviewer that the immunoblot-based analyses reveal only very subtle differences, which may reflect the loss of spatial information when analyzing total cell lysates. As suggested, we therefore performed IF/ICC-based analyses to better capture the localization and enrichment of Met1-linked ubiquitin chains and strengthen the overall conclusions (new Fig 2H and Fig EV5H).

8. Fig. S5B: The described differences between puromycin treatment and untreated conditions are extremely subtle on the anti-Ub blot, and absent in the anti-Met1 blot. I recommend that the authors remove this sentence, based on the shown data: 'we observed a modest increase in the signal of Met1-linked ubiquitin chains after puromycin treatment'.

We agree with the reviewer and removed the part of the sentence as suggested.

9. Fig. 5F-G: The authors went back to their more-artificial system from Fig 1, in which HOIL-1L was transiently overexpressed (WT or inactive mutant) in SH-SY5Y cells, alongside alpha-synuclein aggregate formation.

a. The claims made from this experiment would be stronger in the other cell system. Could OTULIN be transiently overexpressed in the MEF cells, to monitor the effect of aggregate formation and clearance. Again, staining aggregates with the M1-antibody would improve this experiment.

We thank the reviewer for this helpful suggestion. We performed the recommended experiments and included the results in the revised manuscript (new Fig. EV5H). Since MEFs are inherently difficult to transfect efficiently, establishing conditions for transient expression required substantial optimization. Nevertheless, despite variability associated with transfection efficiency, the results showed a trend consistent with our model, although the differences did not reach statistical significance (please see below the statistical analysis). Importantly, the observed direction of the effect supports our overall conclusions.

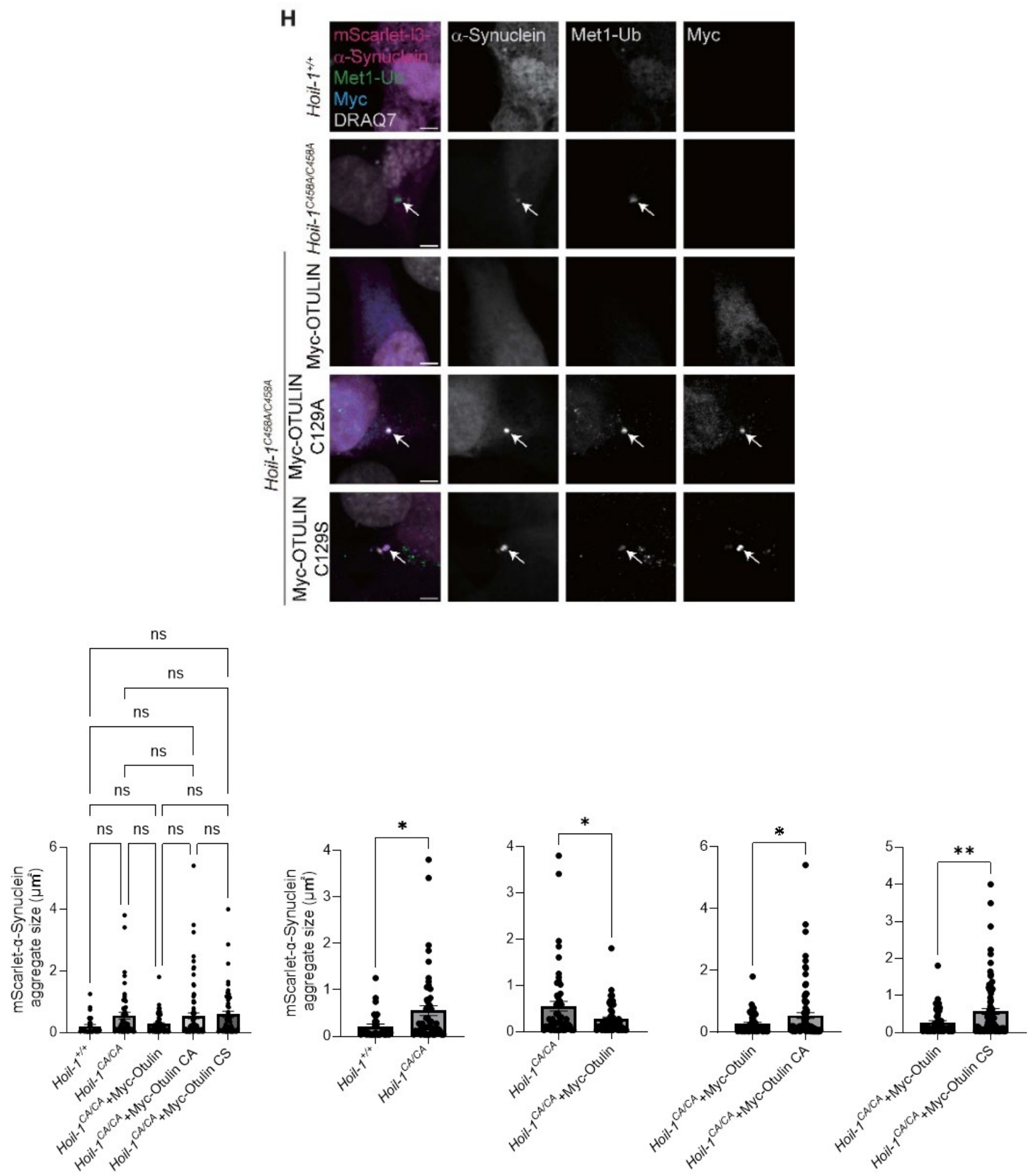

Kruskal-Wallis test

Unpaired t-test

b. The authors claim that HOIL-1 activity fine-tunes the function of HOIP within LUBAC (from discussion: 'This regulatory mechanism ensures that in the presence of functional HOIL-1, the overall quantity and potentially the architecture of Met1-linked ubiquitin chains are tightly controlled'). What is the quantity and architecture of M1-chains catalysed by LUBAC when HOIL-1 is very highly abundant, as it would be the case in this cellular overexpression system?

This is a challenging issue, and we agree that there is no straightforward experimental approach to fully resolve it at present. Importantly, our observations depend on the coordinated activities of both HOIL-1 and HOIP in generating Met1-linked ubiquitin chains. We propose that excess HOIL-1 relative to HOIP may result in orphan HOIL-1 molecules that do not contribute to Met1-chain formation and therefore do not alter overall Met1-ubiquitin levels. Together with data derived from non-overexpression MEFs (Fig 2– Fig 5), we believe our findings collectively support our conclusions. Nevertheless, we acknowledge the limitations and aim to address this question in greater depth in a future, dedicated study.

10. Sentence structure: 'A comprehensive understanding of how Met1-linked ubiquitination, particularly through intricate regulation by Linear Ubiquitin Chain Assembly Complex (LUBAC) components, such as HOIL-1, influences aggregate dynamics and clearance; therefore, it is crucial to develop targeted therapeutic strategies against neurodegenerative proteinopathies'.

Thank you for pointing this out. We agree that the sentence structure is unclear. We revised it for clarity and readability, as follows:

“A comprehensive understanding of how Met1 linked ubiquitination—particularly through its intricate regulation by Linear Ubiquitin Chain Assembly Complex (LUBAC) components such as HOIL 1—influences aggregate dynamics and clearance is crucial for the development of targeted therapeutic strategies against neurodegenerative proteinopathies.”
This revised wording has been incorporated into the manuscript (in pages 3-4).

Reviewer #3 (Significance (Required)):

Significance
The conclusions drawn from this study are very intriguing and give LUBAC (and HOIL-1) a so far unrecognised role in the clearance of protein aggregates, which are a hallmark of several neurodegenerative diseases for which there are currently no cures. Some of the findings described in this manuscript have the potential to be of very high impact and interest to a broader community, in particular researchers interested in protein homeostasis, autophagy, ubiquitin biology and neurodegeneration. In fact, those findings might even expand to autophagic pathways that target other cargo than protein aggregates. Both the novelty aspect and the potential for translational/therapeutic applications comprise the major strength of this manuscript. However, multiple of the presented experiments are currently lacking crucial controls, show weak effect sizes or were performed in artificial settings that likely do not represent relevant in vivo conditions, overall weakening or not fully supporting the claims made. Consequently, further experiments, data re-analyses and validations were recommended to fully support all the claims made here.
This review was written from the perspective of a researcher in the ubiquitin field.

Prof. Fumiyo Ikeda
The University of Osaka
Graduate School of Frontier Biosciences
Osaka
Japan

27th Aug 2026

Re: EMBOJ-2026-124365R
Excess Met1-ubiquitination leads to solid aggregate formation

Dear Fumiyo,

Thank you for submitting your revised manuscript on linear ubiquitination and aggregate formation to The EMBO Journal. I sent it back to two of the original Review Commons referees, who have now returned the comments copied below. Both of them consider the work significantly strengthened and most of the earlier concerns addressed, except for a few minor points still mentioned by referee 1. We shall therefore be happy to accept the manuscript for publication, as soon as these presentational points as well as the following editorial requests have been incorporated:

- Please carefully go through the reference list and make sure that all references have their complete citation information - including year, journal name & volume, and page/elocator numbers - some of this information is currently missing for several of them.

- Please remove the Reagents and Tools table from the manuscript text, and upload it separately.

- As we have switched from a free-text author contribution statement towards a more formal statement based on Contributor Role Taxonomy (CRediT) terms, please remove the present Author Contribution section and instead specify each author's contribution(s) directly in the Author Information page of our submission system during upload of the final manuscript. See https://casrai.org/credit/ for more information.

- Please provide suggestions for a short 'blurb' text prefacing and summing up the conceptual aspect of the study in two sentences (max. 250 characters), followed by 3-5 one-sentence 'bullet points' with brief factual statements of key results of the paper; they will form the basis of an editor-written 'Synopsis' accompanying the online version of the article. Please also upload a synopsis image, which can be used as a "visual title" for the synopsis section of your paper. The image should be in JPG format and exactly 550 pixels wide and 300 pixels high, and please make sure that it remains clear enough even in these modest dimensions.

- Finally, during routine pre-acceptance checks, our data editors have raised the following queries regarding figures, data, and legends; I would appreciate if you briefly answered to them in the cover letter of your final submission, and made the requested text modifications with changes/additions highlighted via the "Track changes" option, to facilitate our final checking:
1) Please define the annotated p values ****/***/**/* as well as provide the exact p-values for the same in the legend of figure 2C, D, F, G; 3H, EV1 F as appropriate.
2) Please provide the exact p values in the legends of figures 3I, 4D, G, H, J, K; 5I, EV1 E, EV2 B, EV3 G, J, K; EV4 A, F.
3) Please indicate the statistical test used for data analysis in the legend of figure 2D
4) Please provide information related to 'n' in the legends of figures 2D, 5C.
5) Please define the error bars in the legends of figures 5C, 7C, D, E, F.
6) Please define the scale bar for figures 4F, I
7) Please define the white arrow in the legend of figureEV1 B-D, EV5 H.
8) Please define the yellow arrow in the legends of figures 3A, D.
9) Please define the orange arrow in the legend of figure 5D.
10) Please define the white dashed borders in the legend of figures 1D, E; 2B, F; 5B.
11) Please define the yellow dashed borders in the legend of figures 4C, 5G.

I am returning the manuscript to you for minor revision solely to allow you to make these last changes, hoping you will be able to return the final modified files within the course of one week. Once we will have received them, we should be ready to swiftly proceed with formal acceptance and production of the manuscript.

With kind regards,

Hartmut

Hartmut Vodermaier, PhD
Senior Editor, The EMBO Journal
h.vodermaier@embojournal.org

*** PLEASE NOTE: All revised manuscript are subject to initial checks for completeness and adherence to our formatting guidelines. Revisions may be returned to the authors and delayed in their editorial re-evaluation if they fail to comply to the following requirements. As a first step please read our guidelines for revised submissions: https://link.springer.com/journal/44318/submission-guidelines#cms-Revised-submissions

1) Every manuscript requires a Data Availability section (even if only stating that no deposited datasets are included). Primary datasets or computer code produced in the current study have to be deposited in appropriate public repositories prior to resubmission, and reviewer access details provided in case that public access is not yet allowed.

2) Each figure legend must specify
- size of the scale bars that are mandatory for all micrograph panels
- the statistical test used to generate error bars and P-values
- the type error bars (e.g., S.E.M., S.D.)
- the number (n) and nature (biological or technical replicate) of independent experiments underlying each data point
- Figures may not include error bars for experiments with n<3; scatter plots showing individual data points should be used instead.

3) Revised manuscript text (including main tables, and figure legends for main and EV figures) has to be submitted as editable text file (e.g., .docx format). We encourage highlighting of changes (e.g., via text color) for the referees' reference.

4) Each main and each Expanded View (EV) figure should be uploaded as individual production-quality files (preferably in .eps, .tif, .jpg formats). For suggestions on figure preparation/layout, please refer to our Figure Preparation Guidelines: https://media.springernature.com/original/springer-cms/rest/v1/content/27825798/data/v1

5) Point-by-point response letters should include the original referee comments in full together with your detailed responses to them (and to specific editor requests if applicable), and also be uploaded as editable (e.g., .docx) text files.

6) Please complete our Author Checklist, and make sure that information entered into the checklist is also reflected in the manuscript; the checklist will be available to readers as part of the Review Process File.

7) All authors listed as (co-)corresponding need to deposit, in their respective author profiles in our submission system, a unique ORCiD identifier linked to their name. Please see our Guide to Authors for detailed instructions.

8) Please note that supplementary information at EMBO Press has been superseded by the 'Expanded View' for inclusion of additional figures, tables, movies or datasets; with up to five EV Figures being typeset and directly accessible in the HTML version of the article.

9) To facilitate reproducibilty and cross-laboratory adoption of methodologies, please structure the Materials & Methods section as outlined in our guide to authors, including a completed Reagents and Tools Table.

10) Digital image enhancement is acceptable practice, as long as it accurately represents the original data and conforms to community standards. If a figure has been subjected to significant electronic manipulation, this must be clearly noted in the figure legend and/or the 'Materials and Methods' section. The editors reserve the right to request original versions of figures and the original images that were used to assemble the figure. Finally, we generally encourage uploading of numerical as well as gel/blot image source data.

At EMBO Press we ask authors to provide source data for the main manuscript figures. You will receive a separate email with instructions for providing source data with your revised manuscript, including how to upload and organize the files.

In the interest of ensuring the conceptual advance provided by the work, we recommend submitting a revision within 3 months (25th Nov 2026). Please discuss the revision progress ahead of this time with the editor if you require more time to complete the revisions. Use the link below to submit your revision:

Link Not Available

Referee #1:

The authors have addressed all the major points made in our original review. The inclusion of results showing co-localisation of Met1 chains with p62-positive aggregates is particularly important, as this was a major omission in the original manuscript.

The authors still need to address the minor issues with the manuscript listed below
Page 8 - "bulk autophagic flux induced by amino acid starvation was not significantly ltered in Hoil-1C458A/C458A MEFS" should read "bulk autophagic flux induced by amino acid starvation was not significantly altered in Hoil-1C458A/C458A MEFS"
Page 9 - I would recommend removing the word "that" from the sentence "Circularity and 1,6-HD sensitivity capture distinct and complementary properties, and that aggregate shape alone should not be interpreted as a direct measure of liquidity."
Page 9- At reviewer 3's suggestion, the authors include McCrory et al (2022) as a reference. The issue is where Kaypee et al choose to use it - it is provided as a reference that the HOIL-1 C460A catalytically inactive mutant produces Met1-linked ubiquitin chains more efficiently than wildtype LUBAC in vitro, something that does not seem relevant to the McCrory study, which uses a C458S mutation in murine BMDMs and does not directly address the issue Kaypee et al are discussing. Furthermore, the authors do not cite work from the Iwai group (Fuseya et al 2020) that does, in fact, back up the statement that they make about the increased activity of LUBAC complexes containing a HOIL-1 C460A mutant. Therefore, the referencing of this section still needs addressing.
Page 14 - The reference Gomez-Diaz et al (2021) appears several times in the reagents table as a source of the human HOIL-1 cDNA clones. This reference is absent from the reference list.
Page 24 - I think that the opening sentence of the "Sucrose gradient fractionation" section should read "MEFs were treated with 5 µg/ml puromycin..." rather than "synucleinMEFs were treated with 5 µg/ml puromycin..."
Page 29 - Reference Zheng et al (2011) is missing volume and page numbers (50; e2568)
Page 31 - The figure legend for Fig 3A contains a repetition of "Scale bar, 5", which should be removed.

Referee #3:

The authors have addressed all comments raised by this reviewer.
The manuscript remains very compelling. New data, especially p7 EV3A/B nicely support the model. An additional possibility is that the branches generated by HOIL1 represent a specific signal to the p62/autophagy machinery that an M1 chain alone cannot produce.
The newly incorporated text introduced a few typos and grammatical errors that should be revised.

Manuscript number: EMBOJ-2026-124365R
Corresponding author(s): Fumiyo, Ikeda

We sincerely thank both of the reviewers for their thorough evaluations of our revised manuscript. We corrected all the points raised in the text.

Detailed point-by-point responses to each reviewer comment are provided below.

Referee #1:

The authors have addressed all the major points made in our original review. The inclusion of results showing co-localisation of Met1 chains with p62-positive aggregates is particularly important, as this was a major omission in the original manuscript.

The authors still need to address the minor issues with the manuscript listed below
Page 8 - "bulk autophagic flux induced by amino acid starvation was not significantly ltered in Hoil-1C458A/C458A MEFS" should read "bulk autophagic flux induced by amino acid starvation was not significantly altered in Hoil-1C458A/C458A MEFS"

This typo was fixed.

Page 9 - I would recommend removing the word "that" from the sentence "Circularity and 1,6-HD sensitivity capture distinct and complementary properties, and that aggregate shape alone should not be interpreted as a direct measure of liquidity."

We removed “that” from the sentence.

Page 9- At reviewer 3's suggestion, the authors include McCrory et al (2022) as a reference. The issue is where Kaypee et al choose to use it - it is provided as a reference that the HOIL-1 C460A catalytically inactive mutant produces Met1-linked ubiquitin chains more efficiently than wildtype LUBAC in vitro, something that does not seem relevant to the McCrory study, which uses a C458S mutation in murine BMDMs and does not directly address the issue Kaypee et al are discussing. Furthermore, the authors do not cite work from the Iwai group (Fuseya et al 2020) that does, in fact, back up the statement that they make about the increased activity of LUBAC complexes containing a HOIL-1 C460A mutant. Therefore, the referencing of this section still needs addressing.

We rephrased the text and cited both papers, Fuseya et al and McCrory et al.

“We and others demonstrated that, *in vitro*, LUBAC containing a point mutation at HOIL-1 C460 catalytic site (Fuseya *et al*, 2020; McCrory *et al*, 2022) produces Met1-linked ubiquitin chains more efficiently than wild-type LUBAC (Rodriguez Carvajal *et al.*, 2021).”

Page 14 - The reference Gomez-Diaz et al (2021) appears several times in the reagents table as a source of the human HOIL-1 cDNA clones. This reference is absent from the reference list.

We corrected the citation list by adding this paper.

Page 24 - I think that the opening sentence of the "Sucrose gradient fractionation" section should read "MEFs were treated with 5 µg/ml puromycin..." rather than "synucleinMEFs were treated with 5 µg/ml puromycin..."

We corrected the sentence by removing “synuclein”.

Page 29 - Reference Zheng et al (2011) is missing volume and page numbers (50; e2568)

We reviewed the entire reference list and added page numbers to all references in which they were previously missing.

Page 31 - The figure legend for Fig 3A contains a repetition of "Scale bar, 5", which should be removed.

We removed these repeated words.

Referee #3:

The authors have addressed all comments raised by this reviewer.
The manuscript remains very compelling. New data, especially p7 EV3A/B nicely support the model. An additional possibility is that the branches generated by HOIL1 represent a specific signal to the p62/autophagy machinery that an M1 chain alone cannot produce.

The newly incorporated text introduced a few typos and grammatical errors that should be revised.

We corrected typing errors in the text.

EMBO Press Author Checklist

Corresponding Author Name: Fumiyo Ikeda	
Journal Submitted to: The EMBO Journal	
Manuscript Number: EMBOJ-2026-124365-T	

USEFUL LINKS FOR COMPLETING THIS FORM
The EMBO Journal - Author Guidelines
EMBO Reports - Author Guidelines
Molecular Systems Biology - Author Guidelines
EMBO Molecular Medicine - Author Guidelines

Reporting Checklist for Life Science Articles (updated January
This checklist is adapted from Materials Design Analysis Reporting (MDAR) Checklist for Authors. MDAR establishes a minimum set of requirements in transparent reporting in the life sciences (see Statement of Task: 10.31222/osf.io/9sm4x). Please follow the journal's guidelines in preparing your
Please note that a copy of this checklist will be published alongside your article.

Abridged guidelines for figures
1. Data
The data shown in figures should satisfy the following conditions:
- ➞ the data were obtained and processed according to the field's best practice and are presented to reflect the results of the experiments in an accurate and unbiased manner.
- ➞ ideally, figure panels should include only measurements that are directly comparable to each other and obtained with the same assay.
- ➞ plots include clearly labeled error bars for independent experiments and sample sizes. Unless justified, error bars should not be shown for technical
- ➞ if n<5, the individual data points from each experiment should be plotted. Any statistical test employed should be justified.
- ➞ Source Data should be included to report the data underlying figures according to the guidelines set out in the authorship guidelines on Data

2. Captions
Each figure caption should contain the following information, for each panel where they are relevant:
- ➞ a specification of the experimental system investigated (eg cell line, species name).
- ➞ the assay(s) and method(s) used to carry out the reported observations and measurements.
- ➞ an explicit mention of the biological and chemical entity(ies) that are being measured.
- ➞ an explicit mention of the biological and chemical entity(ies) that are altered/varied/perturbed in a controlled manner.
- ➞ the exact sample size (n) for each experimental group/condition, given as a number, not a range;
- ➞ a description of the sample collection allowing the reader to understand whether the samples represent technical or biological replicates (including how many animals, litters, cultures, etc.).
- ➞ a statement of how many times the experiment shown was independently replicated in the laboratory.
- ➞ definitions of statistical methods and measures:
  - common tests, such as t-test (please specify whether paired vs. unpaired), simple χ2 tests, Wilcoxon and Mann-Whitney tests, can be unambiguously identified by name only, but more complex techniques should be described in the methods section;
  - are tests one-sided or two-sided?
  - are there adjustments for multiple comparisons?
  - exact statistical test results, e.g., P values = x but not P values < x;
  - definition of 'center values' as median or average;
  - definition of error bars as s.d. or s.e.m.

Please complete ALL of the questions below.
Select "Not Applicable" only when the requested information is not relevant for your study.

Materials

Newly Created Materials	Information included in the manuscript?	In which section is the information available? (Reagents and Tools Table, Materials and Methods, Figures, Data Availability Section)
New materials and reagents need to be available; do any restrictions apply?	Yes	Reagents and Tools Table and Method section

Antibodies	Information included in the manuscript?	In which section is the information available? (Reagents and Tools Table, Materials and Methods, Figures, Data Availability Section)
For **antibodies** provide the following information:<br>- Commercial antibodies: RRID (if possible) or supplier name, catalogue number and or/clone number<br>- Non-commercial: RRID or citation	Yes	Reagents and Tools Table and Method section

DNA and RNA sequences	Information included in the manuscript?	In which section is the information available? (Reagents and Tools Table, Materials and Methods, Figures, Data Availability Section)
**Short novel DNA or RNA including primers, probes**: provide the sequences.	Yes	Reagents and Tools Table

Cell materials	Information included in the manuscript?	In which section is the information available? (Reagents and Tools Table, Materials and Methods, Figures, Data Availability Section)
**Cell lines:** Provide species information, strain. Provide accession number in repository **OR** supplier name, catalog number, clone number, and/**OR** RRID.	Yes	Reagents and Tools Table
**Primary cultures:** Provide species, strain, sex of origin, genetic modification status.	Not Applicable	
Report if the cell lines were recently **authenticated** (e.g., by STR profiling) and tested for mycoplasma contamination.	Yes	Method section

Experimental animals	Information included in the manuscript?	In which section is the information available? (Reagents and Tools Table, Materials and Methods, Figures, Data Availability Section)
**Laboratory animals or Model organisms:** Provide species, strain, sex, age, genetic modification status. Provide accession number in repository **OR** supplier name, catalog number, clone number, **OR** RRID.	Not Applicable	
**Animal observed in or captured from the field:** Provide species, sex, and age where possible.	Not Applicable	
Please detail **housing and husbandry conditions**.	Not Applicable	

Plants and microbes	Information included in the manuscript?	In which section is the information available? (Reagents and Tools Table, Materials and Methods, Figures, Data Availability Section)
**Plants:** provide species and strain, ecotype and cultivar where relevant, unique accession number if available, and source (including location for collected wild specimens).	Not Applicable	
**Microbes:** provide species and strain, unique accession number if available, and source.	Not Applicable	

Human research participants	Information included in the manuscript?	In which section is the information available? (Reagents and Tools Table, Materials and Methods, Figures, Data Availability Section)
If collected and within the bounds of privacy constraints report on age, sex and gender or ethnicity for all study participants.	Not Applicable	

Core facilities	Information included in the manuscript?	In which section is the information available? (Reagents and Tools Table, Materials and Methods, Figures, Data Availability Section)
If your work benefited from core facilities, was their service mentioned in the acknowledgments section?	Yes	Acknowledgements section

Design

Study protocol	Information included in the manuscript?	In which section is the information available? (Reagents and Tools Table, Materials and Methods, Figures, Data Availability Section)
If study protocol has been **pre-registered, provide DOI in the manuscript**. For clinical trials, provide the trial registration number **OR** cite DOI.	Not Applicable	
Report the **clinical trial registration number** (at ClinicalTrials.gov or equivalent), where applicable.	Not Applicable	

Laboratory protocol	Information included in the manuscript?	In which section is the information available? (Reagents and Tools Table, Materials and Methods, Figures, Data Availability Section)
Provide DOI OR other citation details if **external detailed step-by-step protocols** are available.	Not Applicable	

Experimental study design and statistics	Information included in the manuscript?	In which section is the information available? (Reagents and Tools Table, Materials and Methods, Figures, Data Availability Section)
Include a statement about **sample size** estimate even if no statistical methods were used.	Yes	Figure legends
Were any steps taken to minimize the effects of subjective bias when allocating animals/samples to treatment (e.g. **randomization procedure**)? If yes, have they been described?	Not Applicable	
Include a statement about **blinding** even if no blinding was done.	Not Applicable	
Describe **inclusion/exclusion criteria** if samples or animals were excluded from the analysis. Were the criteria pre-established?<br><br>If sample or data points were omitted from analysis, report if this was due to attrition or intentional exclusion and provide justification.	Not Applicable	
For every figure, are **statistical tests** justified as appropriate? Do the data meet the assumptions of the tests (e.g., normal distribution)? Describe any methods used to assess it. Is there an estimate of variation within each group of data? Is the variance similar between the groups that are being statistically compared?	Yes	Methods section

Sample definition and in-laboratory replication	Information included in the manuscript?	In which section is the information available? (Reagents and Tools Table, Materials and Methods, Figures, Data Availability Section)
In the figure legends: state number of times the experiment was **replicated** in laboratory.	Yes	Figure legends
In the figure legends: define whether data describe **technical or biological replicates**.	Yes	Figure legends

Ethics

Ethics	Information included in the manuscript?	In which section is the information available? (Reagents and Tools Table, Materials and Methods, Figures, Data Availability Section)
Studies involving **human participants**: State details of **authority granting ethics approval** (IRB or equivalent committee(s), provide reference number for approval.	Not Applicable	
Studies involving **human participants**: Include a statement confirming that **informed consent** was obtained from all subjects and that the experiments conformed to the principles set out in the WMA Declaration of Helsinki and the Department of Health and Human Services Belmont Report.	Not Applicable	
Studies involving **human participants:** For publication of **patient photos**, include a statement confirming that consent to publish was obtained.	Not Applicable	
Studies involving experimental **animals**: State details of **authority granting ethics approval** (IRB or equivalent committee(s), provide reference number for approval. Include a statement of compliance with ethical regulations.	Not Applicable	
Studies involving **specimen and field samples:** State if relevant **permits** obtained, provide details of authority approving study; if none were required, explain why.	Not Applicable	

Dual Use Research of Concern (DURC)	Information included in the manuscript?	In which section is the information available? (Reagents and Tools Table, Materials and Methods, Figures, Data Availability Section)
Could your study fall under dual use research restrictions? Please check biosecurity documents and list of **select agents and toxins** (CDC): https://www.selectagents.gov/sat/list.htm	Not Applicable	
If you used a select agent, is the security level of the lab appropriate and reported in the manuscript?	Not Applicable	
If a study is subject to dual use research of concern regulations, is the name of the **authority granting approval and reference number** for the regulatory approval provided in the manuscript?	Not Applicable	

Reporting

The MDAR framework recommends adoption of discipline-specific guidelines, established and endorsed through community initiatives. Journals have their own policy about requiring specific guidelines and recommendations to complement MDAR.

Adherence to community standards	Information included in the manuscript?	In which section is the information available? (Reagents and Tools Table, Materials and Methods, Figures, Data Availability Section)
State if relevant guidelines or checklists (e.g., **ICMJE, MIBBI, ARRIVE, PRISMA**) have been followed or provided.	Not Applicable	
For **tumor marker prognostic studies,** we recommend that you follow the **REMARK** reporting guidelines (see link list at top right). See author guidelines, under 'Reporting Guidelines'. Please confirm you have followed these guidelines.	Not Applicable	
For **phase II and III randomized controlled trials**, please refer to the **CONSORT** flow diagram (see link list at top right) and submit the CONSORT checklist (see link list at top right) with your submission. See author guidelines, under 'Reporting Guidelines'. Please confirm you have submitted this list.	Not Applicable	

Data Availability

Data availability	Information included in the manuscript?	In which section is the information available? (Reagents and Tools Table, Materials and Methods, Figures, Data Availability Section)
Have **primary datasets** been deposited according to the journal's guidelines (see 'Data Deposition' section) and the respective accession numbers provided in the Data Availability Section?	Yes	Data availability section
Were **human clinical and genomic datasets** deposited in a public access-controlled repository in accordance to ethical obligations to the patients and to the applicable consent agreement?	Not Applicable	
Are **computational models** that are central and integral to a study available without restrictions in a machine-readable form? Were the relevant accession numbers or links provided?	Not Applicable	
If publicly available data were reused, provide the respective **data citations in the reference list**.	Not Applicable	
