# Supplementary material for: Excess Met1-linked ubiquitination leads to solid aggregate formation
Source: EMBO J. 2026 Sep 28;45(20):7246–73. doi: 10.1038/s44318-026-00909-7 (PMC13672799; doi:10.1038/s44318-026-00909-7)
Supplement: Supplementary file 2 [file 44318_2026_909_MOESM7_ESM.pdf]

Expanded View Figures

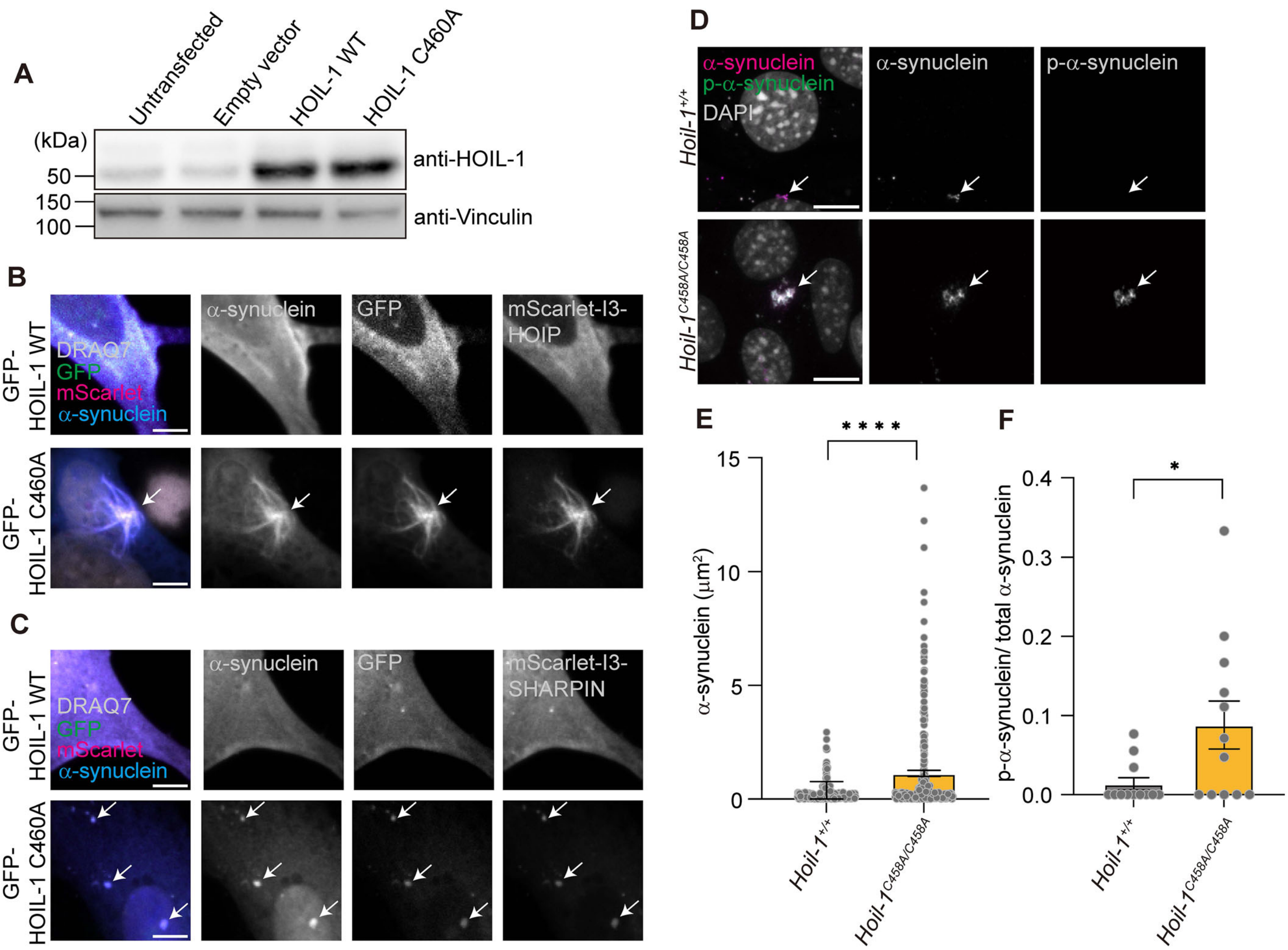

Figure EV1. LUBAC components localize to protein aggregates, which accumulate in cells expressing catalytically inactive HOIL-1.

(**A**) Immunoblot analysis of HOIL-1 expression in SH-SY5Y cells 48 h after transfection with HOIL-1 or HOIL-1 C460A. Untransfected and empty vector (EV)-transfected cells served as controls. Vinculin was used as a loading control. Representative data from one experiment. (**B, C**) Representative fluorescence microscopy images showing colocalization of α-synuclein aggregates with GFP-HOIL-1 or GFP-HOIL-1 C460A and either (**B**) mScarlet-I3-HOIP or (**C**) mScarlet-I3-SHARPIN in SH-SY5Y cells. Representative images from three independent biological experiments are shown. Scale bar, 5 µm. Arrows indicate the position of α-synuclein-positive puncta across each channel. (**D**) Representative immunofluorescence images showing colocalization of α-synuclein aggregates and phosphorylated α-synuclein (p-α-synuclein) in *Hoil-1*$^{+/+}$ and *Hoil-1*$^{C458A/C458A}$ MEFs. Representative images from three independent biological experiments are shown. Scale bar, 10 µm. Arrows indicate the position of α-synuclein-positive puncta across each channel. (**E**) Quantification of α-synuclein aggregate size from (**D**). Data were presented as mean ± SEM from more than 100 cells per condition across three independent biological experiments. Statistical significance was determined using a Mann–Whitney test (****$P = 8.9 \times 10^{-5}$). (**F**) Quantification of the ratio of p-α-synuclein-positive puncta to total α-synuclein puncta from (**D**). Data were presented as mean ± SEM from $n = 12$ fields across three independent biological experiments. Statistical significance was determined using an unpaired *t*-test (*$P = 0.0268$).

 © The Author(s)

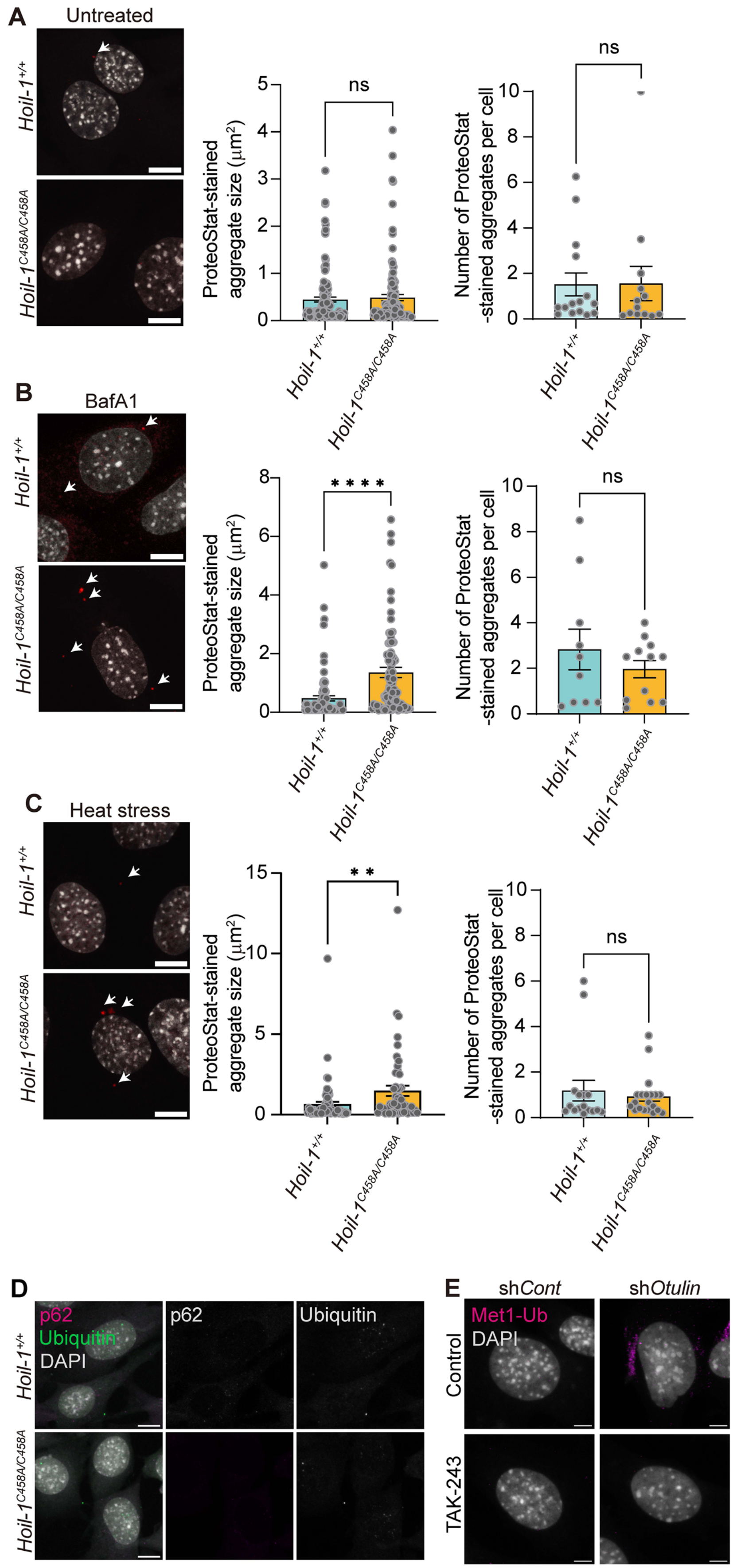
A
Untreated
Hoil-1+/+
Hoil-1C458A/C458A
ProteoStat-stained aggregate size (μm2)
ns
Number of ProteoStat -stained aggregates per cell
ns
B
BafA1
Hoil-1+/+
Hoil-1C458A/C458A
ProteoStat-stained aggregate size (μm2)
****
Number of ProteoStat -stained aggregates per cell
ns
C
Heat stress
Hoil-1+/+
Hoil-1C458A/C458A
ProteoStat-stained aggregate size (μm2)
**
Number of ProteoStat -stained aggregates per cell
ns
D
p62
Ubiquitin
DAPI
p62
Ubiquitin
Hoil-1+/+
Hoil-1C458A/C458A
E
shCont
shOtulin
Met1-Ub
DAPI
Control
TAK-243

Figure EV2. Characterization of proteotoxic stress-induced aggregates in *Hoil-1*$^{C458A/C458A}$ cells.

(**A–C**) Representative images of ProteoStat-stained aggregates (arrows) in *Hoil-1*$^{+/+}$ and *Hoil-1*$^{C458A/C458A}$ MEFs under (**A**) untreated and proteotoxic stress conditions, (**B**) treatment with 100 nM bafilomycin A1 (BafA1), and (**C**) heat stress (42 °C). Scale bar, 5 µm. Quantification of aggregate size and aggregate number per cell is shown alongside the representative images. Data were presented as mean ± SEM from two independent biological experiments (untreated, $n > 75$ cells; BafA1, $n > 30$ cells; heat stress, $n > 55$ cells per condition). Statistical significance was determined using a Mann–Whitney test ((**A**) size: ns, $P = 0.5399$; number: ns, $P = 0.0146$; (**B**) size: ****$P = 2.8 \times 10^{-8}$; number: ns, $P = 0.5343$; (**C**) size: ** $P = 0.0029$; number: ns, $P = 0.0280$). (**D**) Representative immunofluorescence images showing p62 and ubiquitin in untreated *Hoil-1*$^{+/+}$ and *Hoil-1*$^{C458A/C458A}$ MEFs. Representative images from two independent biological experiments are shown. Scale bar, 5 µm. (**E**) Representative fluorescence microscopy images showing Met1-linked ubiquitin chains in sh*Control* and sh*Otulin* MEFs treated with or without the E1 ubiquitin-activating enzyme inhibitor TAK243 (1 µM, 4 h). Scale bar, 5 µm.

© The Author(s)

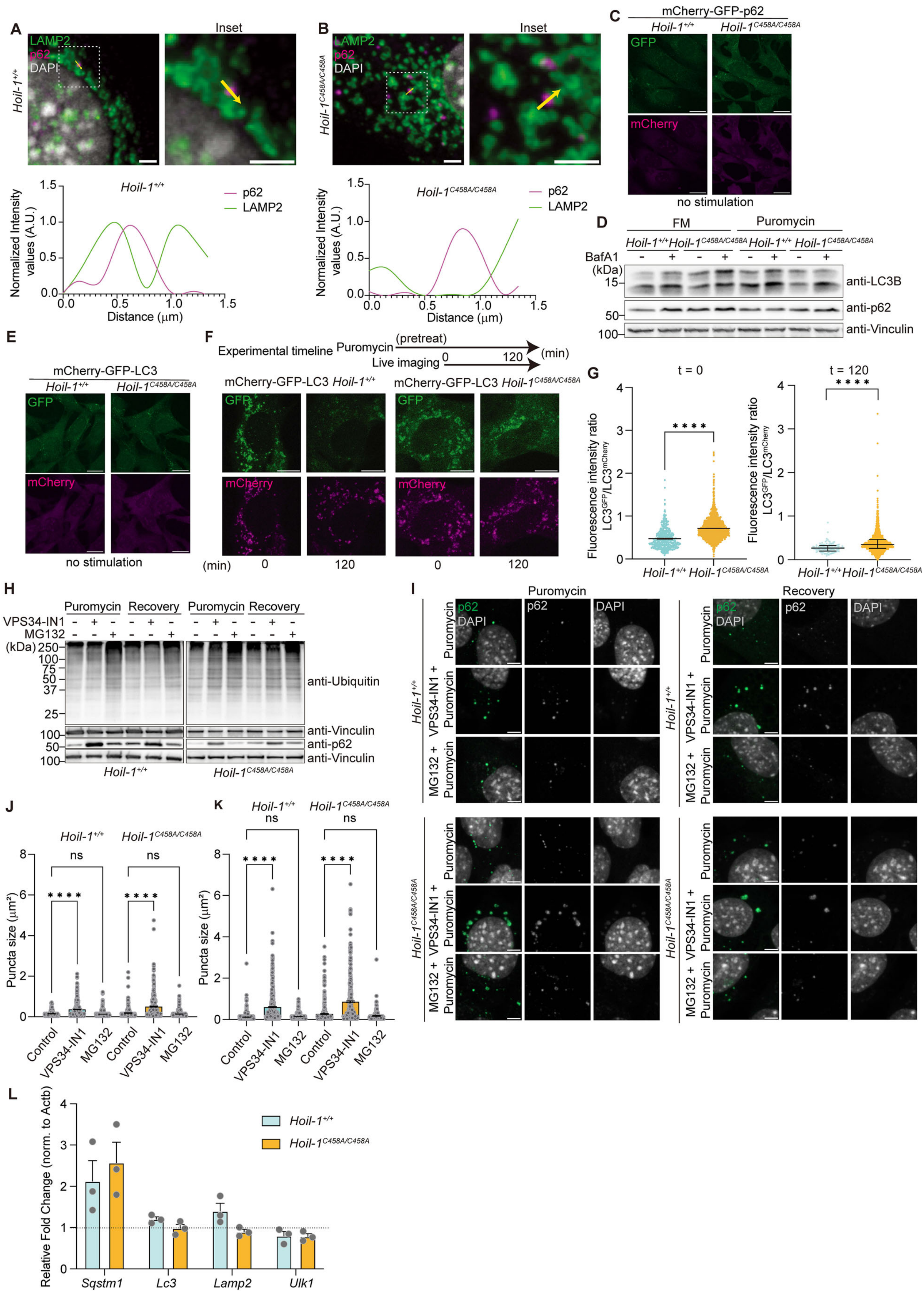
A
LAMP2
p62
DAPI
Hoil-1+/+
Inset
Normalized Intensity values (A.U.)
Distance (μm)
p62
LAMP2
B
Hoil-1C458A/C458A
Inset
C
mCherry-GFP-p62
Hoil-1+/+
Hoil-1C458A/C458A
GFP
mCherry
no stimulation
D
FM
Puromycin
BafA1
(kDa)
anti-LC3B
anti-p62
anti-Vinculin
E
mCherry-GFP-LC3
no stimulation
F
Experimental timeline
Puromycin (pretreat)
Live imaging
(min)
mCherry-GFP-LC3 Hoil-1+/+
mCherry-GFP-LC3 Hoil-1C458A/C458A
G
t = 0
t = 120
Fluorescence intensity ratio LC3GFP/LC3mCherry
H
Puromycin
Recovery
VPS34-IN1
MG132
anti-Ubiquitin
anti-Vinculin
anti-p62
I
p62
DAPI
Puromycin
VPS34-IN1 + Puromycin
MG132 + Puromycin
Recovery
J
K
Puncta size (μm²)
ns
Control
VPS34-IN1
MG132
L
Relative Fold Change (norm. to Actb)
Sqstm1
Lc3
Lamp2
Ulk1

Figure EV3. Aggrephagy is selectively impaired in *Hoil-1*$^{C458A/C458A}$ MEFs.

(**A**, **B**) Fluorescence intensity profiles of p62 and LAMP2 along the line indicated by the arrow in (A) *Hoil-1*$^{+/+}$ and (B) *Hoil-1*$^{C458A/C458A}$ MEFs, illustrating the spatial relationship between p62 and LAMP2 signals. Representative images from three independent biological experiments are shown. Scale bar, 2 µm. White dashed boxes indicate the region magnified in the inset. (**C**) Representative fluorescence microscopy images showing mCherry-EGFP-p62 in untreated *Hoil-1*$^{+/+}$ and *Hoil-1*$^{C458A/C458A}$ MEFs. Representative images from two independent biological experiments are shown. Scale bar, 20 µm. (**D**) Immunoblot analysis of autophagy markers in *Hoil-1*$^{+/+}$ and *Hoil-1*$^{C458A/C458A}$ MEFs cultured in full medium (FM) or following puromycin treatment (2 h), in the presence or absence of bafilomycin A1 (BafA1). Representative data from two independent biological experiments. (**E**) Representative fluorescence microscopy images of *Hoil-1*$^{+/+}$ and *Hoil-1*$^{C458A/C458A}$ MEFs expressing mCherry-EGFP-LC3B under basal (unstimulated) conditions. Scale bar, 20 µm. (**F**) Representative time-lapse imaging of the mCherry-EGFP-LC3 reporter in *Hoil-1*$^{+/+}$ and *Hoil-1*$^{C458A/C458A}$ MEFs following puromycin treatment (5 µg/ml). Cells were pretreated with puromycin prior to live-cell imaging to ensure the formation of LC3-positive puncta before image acquisition ($n = 40$ cells from three independent biological experiments). Scale bar, 10 µm. (**G**) Quantification of the GFP/mCherry fluorescence intensity ratio of the mCherry-EGFP-LC3 reporter shown in (**F**). Ratios were measured at the start of imaging ($t = 0$ min) and at the final time point ($t = 120$ min). Data were presented as mean ± SEM from three independent biological experiments. Statistical significance was determined using a Mann–Whitney test ($t = 0$, ****$P < 1.0\times10^{-15}$, $t = 120$, ****$P = 1.5 \times 10^{-12}$). (**H**) *Hoil-1*$^{+/+}$ and *Hoil-1*$^{C458A/C458A}$ MEFs were pretreated with VPS34-IN1 (5 µM, 16 h) or MG132 (5 µM, 2 h), followed by puromycin treatment (5 µg/ml, 2 h) and a 2 h recovery period in puromycin-free medium. VPS34-IN1 or MG132 was maintained throughout both the puromycin treatment and recovery phases. Cell lysates were subsequently analyzed by immunoblotting using antibodies against p62 and ubiquitin. Vinculin was used as a loading control. Representative data from two independent biological experiments. (**I**) *Hoil-1*$^{+/+}$ and *Hoil-1*$^{C458A/C458A}$ MEFs were pretreated with VPS34-IN1 (5 µM, 16 h) or MG132 (5 µM, 2 h), followed by puromycin treatment (5 µg/ml, 2 h) and a 2 h recovery period in puromycin-free medium. VPS34-IN1 or MG132 was maintained throughout both the puromycin treatment and recovery phases. Cells were subsequently analyzed by immunofluorescence staining for p62. Representative data from two independent biological experiments. Scale bar, 5 µm. (**J**, **K**). Quantification of p62 body size in puromycin-treated (**J**) and recovery (**K**) conditions in *Hoil-1*$^{+/+}$ and *Hoil-1*$^{C458A/C458A}$ MEFs. Analyses were performed using samples treated under the same conditions as those shown in (**I**). Data were presented as mean ± SEM. Statistical significance was assessed using a Kruskal–Wallis test followed by Dunn's multiple-comparison test ((**J**) ****$P = 4.0 \times 10^{-8}$, (**K**) ****$P < 1.0 \times 10^{-15}$). Approximately 30 cells per condition were analyzed across two independent biological experiments.
(**L**) qRT-PCR analysis of autophagy-related gene expression in *Hoil-1*$^{+/+}$ and *Hoil-1*$^{C458A/C458A}$ MEFs following puromycin treatment (5 µg/ml). Gene expression levels were normalized to *Actb* and are shown relative to untreated controls. Data were presented as mean relative fold change ± SEM from three independent biological experiments. No significant differences were observed between genotypes.

 © The Author(s)

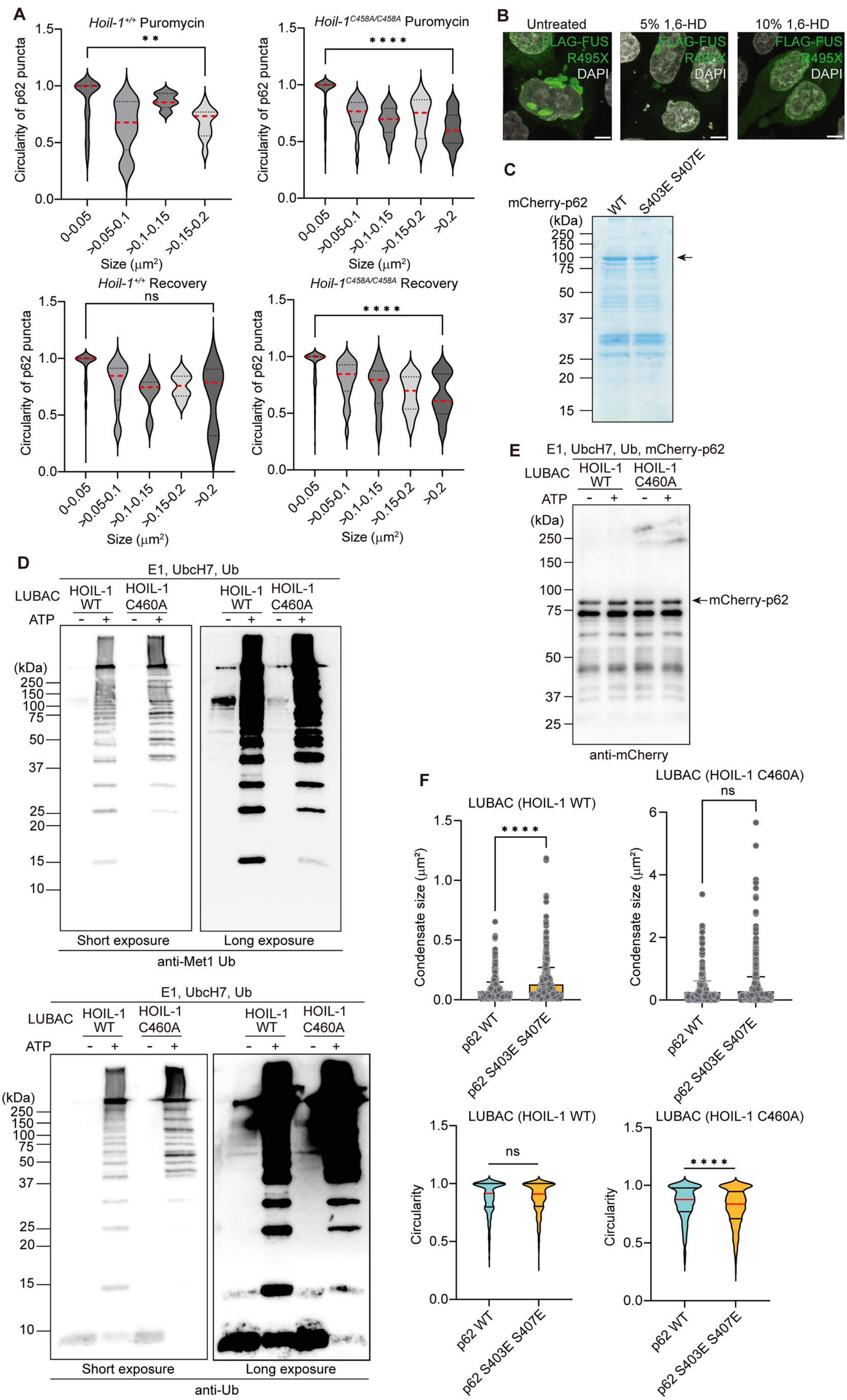
A
Hoil-1+/+ Puromycin
Hoil-1C458A/C458A Puromycin
Hoil-1+/+ Recovery
Hoil-1C458A/C458A Recovery
Circularity of p62 puncta
Size (μm²)
0-0.05
>0.05-0.1
>0.1-0.15
>0.15-0.2
>0.2
ns
B
Untreated
5% 1,6-HD
10% 1,6-HD
FLAG-FUS R495X
DAPI
C
mCherry-p62
WT
S403E S407E
(kDa)
D
E1, UbcH7, Ub
LUBAC
HOIL-1 WT
HOIL-1 C460A
ATP
Short exposure
Long exposure
anti-Met1 Ub
anti-Ub
E
E1, UbcH7, Ub, mCherry-p62
mCherry-p62
anti-mCherry
F
LUBAC (HOIL-1 WT)
LUBAC (HOIL-1 C460A)
Condensate size (μm²)
Circularity
p62 WT
p62 S403E S407E

Figure EV4. Met1-linked ubiquitin chains alter the material properties of p62 condensates.

(**A**) Quantification of p62 body circularity in *Hoil-1*$^{+/+}$ and *Hoil-1*$^{C458A/C458A}$ MEFs following puromycin treatment and after the recovery period. Data were presented from three independent biological experiments. Statistical significance was assessed using a Kruskal–Wallis test (*Hoil-1*$^{+/+}$ Puromycin: **, $P = 0.0097$, *Hoil-1*$^{C458A/C458A}$ Puromycin: ****$P = 6.9 \times 10^{-7}$, *Hoil-1*$^{+/+}$ Recovery: ns, $P = 0.1467$, *Hoil-1*$^{C458A/C458A}$ Recovery: ****$P = 2.3 \times 10^{-7}$). (**B**) Representative images demonstrating the dissolution of FUS R495X condensates in HEK293T cells following treatment with 5% or 10% 1,6-hexanediol (1,6-HD). Scale bar, 10 µm. (**C**) SDS-PAGE analysis of purified recombinant mCherry-p62 proteins visualized by InstantBlue staining. Arrows indicate mCherry-p62 WT and the phosphomimetic mCherry-p62 S403E/S407E mutant. (**D**) Immunoblot analysis of in vitro ubiquitination reactions comparing ubiquitin chains assembled by wild-type LUBAC and LUBAC containing HOIL-1 C460A. Met1-linked and total ubiquitin chains were detected using anti-Met1 ubiquitin and anti-ubiquitin antibodies, respectively. (**E**) Immunoblot analysis of mCherry-p62 in parallel reaction mixtures corresponding to the in vitro LLPS assays shown in Fig. 4F, demonstrating the absence of detectable p62 ubiquitination under the assay conditions used. (**F**) Direct comparison of condensate size and circularity between mCherry-p62 WT and the phosphomimetic mCherry-p62 S403E/S407E mutant using the datasets shown in Fig. 4F–K. Condensate size is presented as mean ± SEM, whereas circularity is presented as median (red line) with interquartile range. Statistical significance was assessed using a Mann–Whitney test (HOIL-1 WT Condensate size: ****$P < 1.0 \times 10^{-15}$; HOIL-1 C460A Condensate size: ns, $P = 0.6951$; HOIL-1 WT Circularity: ns, $P = 0.1619$; HOIL-1 C460A Circularity: ****$P = 5.1 \times 10^{-5}$).

 © The Author(s)

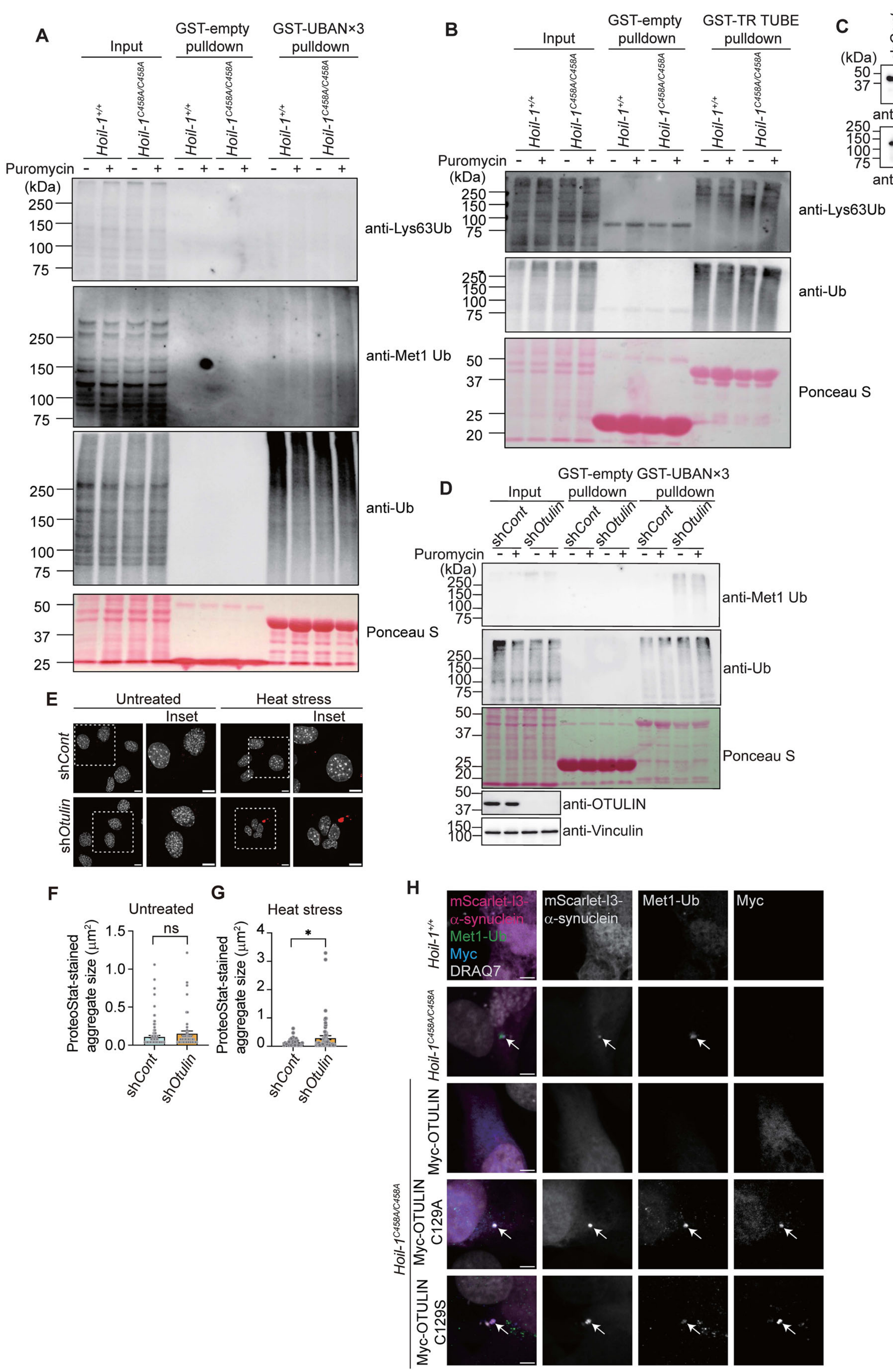
A
Input
GST-empty pulldown
GST-UBAN×3 pulldown
Hoil-1+/+
Hoil-1C458A/C458A
Puromycin
(kDa)
anti-Lys63Ub
anti-Met1 Ub
anti-Ub
Ponceau S
B
GST-TR TUBE pulldown
C
shCont
shOtulin
anti-OTULIN
anti-Vinculin
D
E
Untreated
Heat stress
Inset
F
G
ProteoStat-stained aggregate size (µm²)
ns
*
H
mScarlet-I3-α-synuclein
Met1-Ub
Myc
DRAQ7
Myc-OTULIN
Myc-OTULIN C129A
Myc-OTULIN C129S

◀ **Figure EV5. Elevated Met1-linked ubiquitination promotes persistent aggregate accumulation.**

(**A**) GST and GST-UBAN×3 pull-down assays were performed using lysates from *Hoil-1*$^{+/+}$ and *Hoil-1*$^{C458A/C458A}$ MEFs with or without puromycin treatment, followed by immunoblot analysis. Lys63-linked ubiquitin chains, Met1-linked ubiquitin chains, and total ubiquitin were detected using linkage-specific or pan-ubiquitin antibodies, as indicated. Ponceau S staining (bottom panel) is shown as a loading control. Representative data from two independent biological experiments. (**B**) GST and GST-TR-TUBE pull-down assays were performed using lysates from *Hoil-1*$^{+/+}$ and *Hoil-1*$^{C458A/C458A}$ MEFs, followed by immunoblot analysis of the indicated proteins. Ponceau S staining (bottom panel) is shown as a loading control. Representative data from three independent biological experiments. (**C**) Immunoblot analysis confirming OTULIN knockdown in sh*Otulin* MEFs compared with sh*Control* MEFs. Vinculin was used as a loading control. (**D**) GST and GST-UBAN×3 pull-down assays were performed using lysates from sh*Control* and sh*Otulin* MEFs, followed by immunoblot analysis. Met1-linked ubiquitin chains (top panel) and total ubiquitin (middle panel) were examined in the presence or absence of puromycin treatment (5 µg/ml, 2 h). Ponceau S staining (bottom panel) is shown as a loading control for pull-down samples. OTULIN knockdown was confirmed using an anti-OTULIN antibody, and vinculin was used as a loading control for input samples. Representative data from three independent biological experiments. (**E**) Representative images of ProteoStat-stained aggregates in sh*Control* and sh*Otulin* MEFs subjected to heat stress (42 °C). Representative images from three independent biological experiments are shown. Scale bar, 5 µm. White dashed boxes indicate the region magnified in the inset. (**F**, **G**) Quantification of aggregate size in sh*Control* and sh*Otulin* MEFs under (**F**) untreated and (**G**) heat stress conditions. Data were presented as mean ± SEM from three independent biological experiments ($N = 3$). Statistical significance was assessed using the Mann–Whitney test (ns, $P > 0.05$; *$P = 0.0354$). (**H**) Representative fluorescence microscopy images showing α-synuclein aggregates and Met1-linked ubiquitin chains in *Hoil-1*$^{+/+}$ and *Hoil-1*$^{C458A/C458A}$ MEFs transiently expressing Myc-tagged OTULIN, OTULIN C129A, or OTULIN C129S, as indicated. Representative images from three independent biological experiments are shown. Scale bar, 5 µm. Arrows indicate the position of α-synuclein-positive puncta across each channel.

 © The Author(s)
