# Supplementary material for: Excess Met1-linked ubiquitination leads to solid aggregate formation
Source: EMBO J. 2026 Sep 28;45(20):7246–73. doi: 10.1038/s44318-026-00909-7 (PMC13672799; doi:10.1038/s44318-026-00909-7)
Supplement: Supplementary file 4 [file 44318_2026_909_MOESM3_ESM.zip › Figure 2/2I/WesternBlotting_p62_Uncropped Figure 2I.pdf]

Uncropped Figure 2I

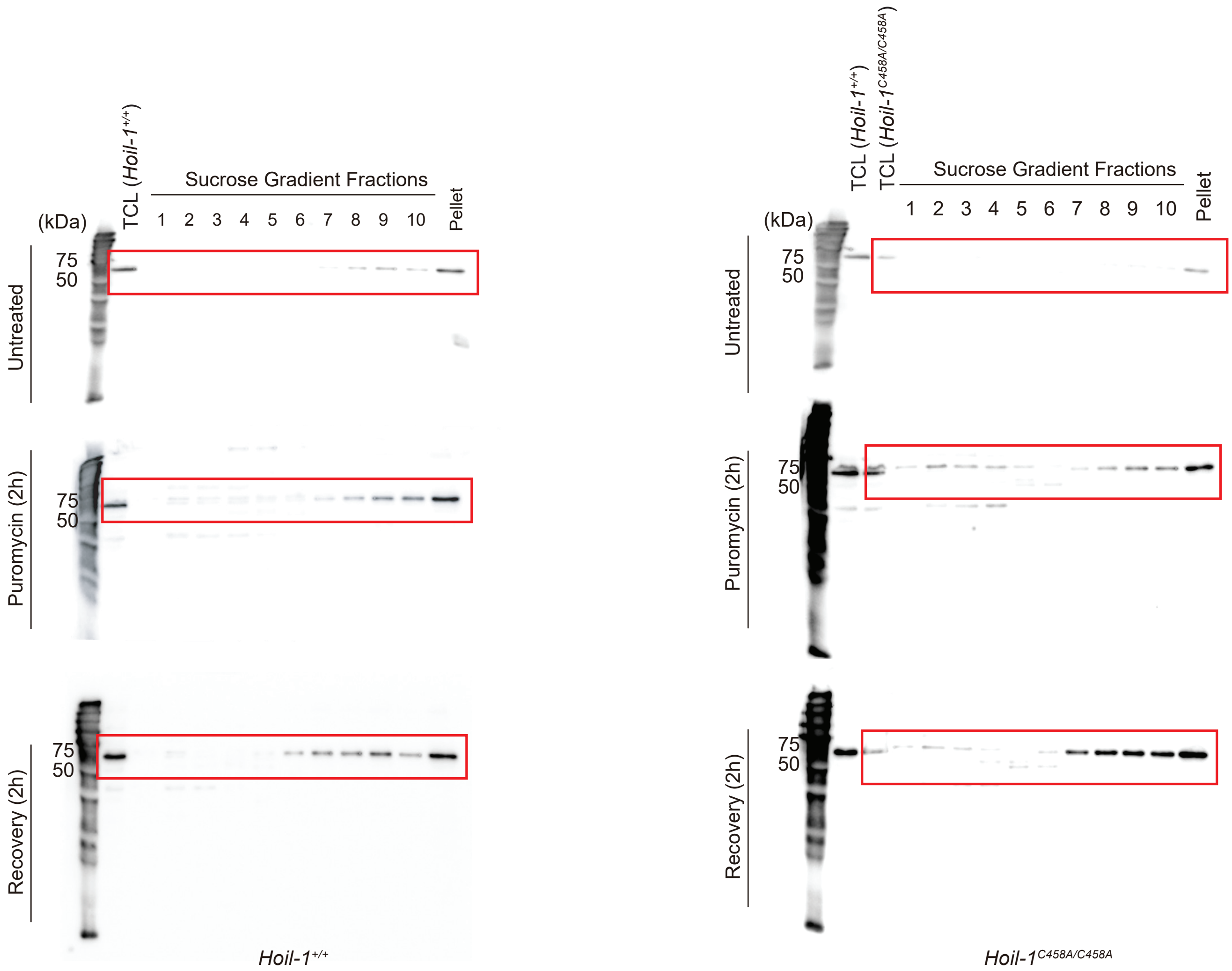

anti-p62
