# Supplementary material for: Excess Met1-linked ubiquitination leads to solid aggregate formation
Source: EMBO J. 2026 Sep 28;45(20):7246–73. doi: 10.1038/s44318-026-00909-7 (PMC13672799; doi:10.1038/s44318-026-00909-7)

Uncropped Figure 3J

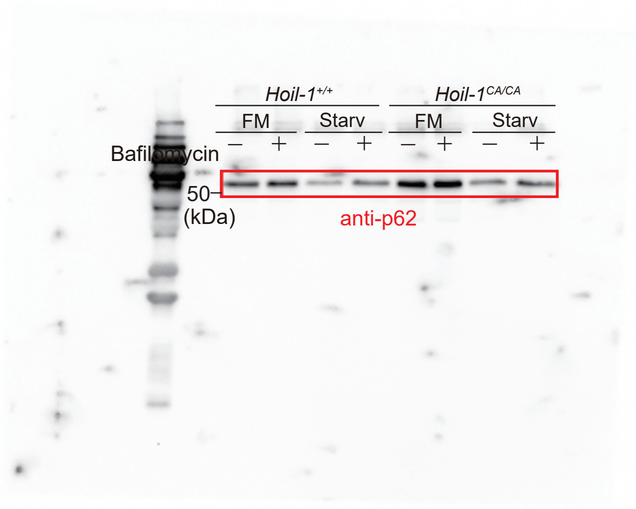

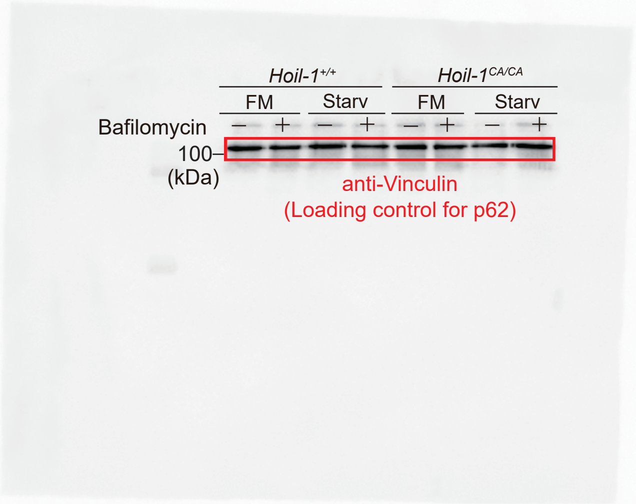

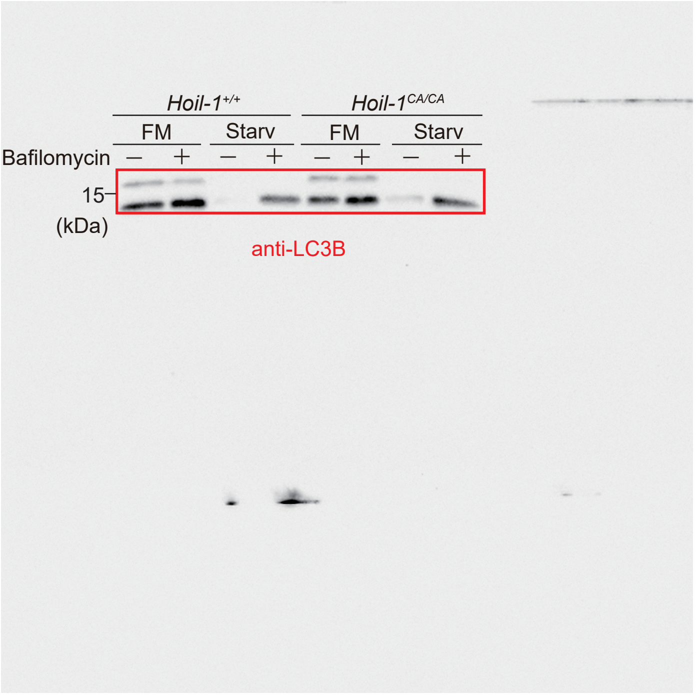

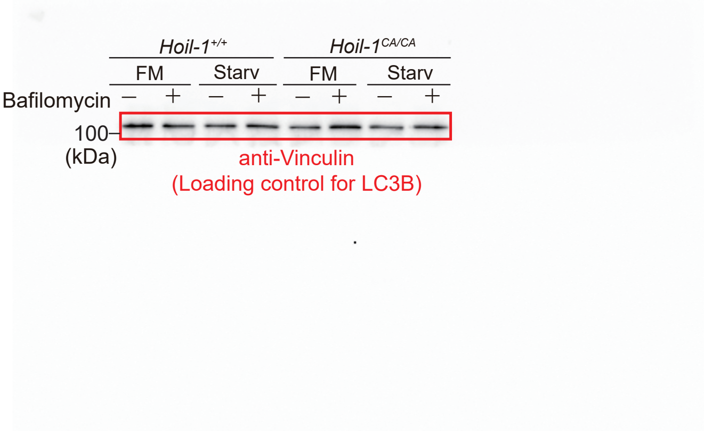

Supplement: Supplementary file 5 [file 44318_2026_909_MOESM4_ESM.zip › Figure 3/3J/WesternBlotting_LC3B_p62_Vinculin_Uncropped Figure 3J.pdf]
