# Supplementary material for: Excess Met1-linked ubiquitination leads to solid aggregate formation
Source: EMBO J. 2026 Sep 28;45(20):7246–73. doi: 10.1038/s44318-026-00909-7 (PMC13672799; doi:10.1038/s44318-026-00909-7)

Uncropped Figure 5A

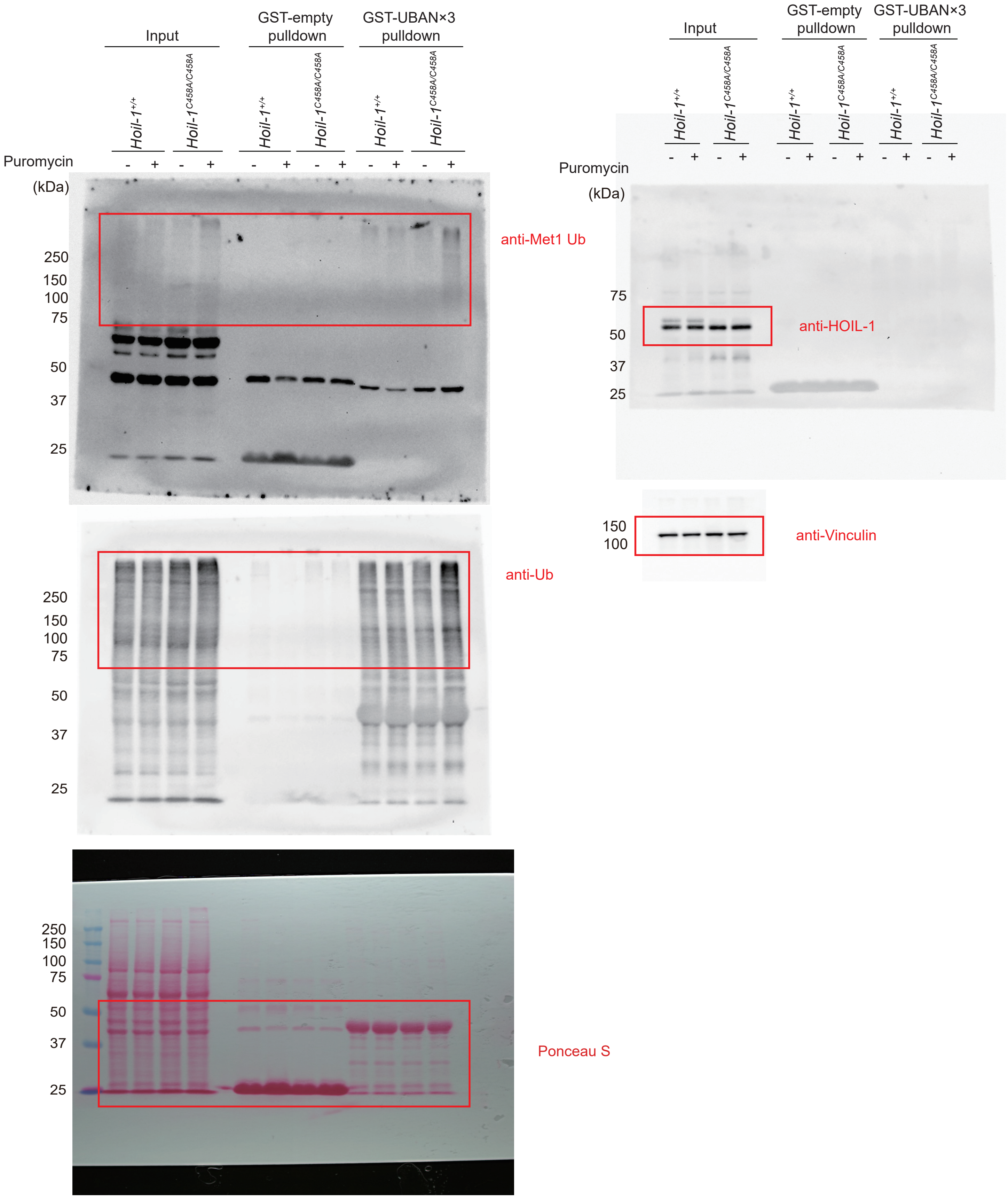

Supplement: Supplementary file 7 [file 44318_2026_909_MOESM6_ESM.zip › Figure 5/5A/Western Blotting_Met1_Ub_Ponceau_HOIL-1_Vinculin_Uncropped Figure 5A.pdf]
